# Elucidation of protein biomarkers for verification of selected biological warfare agents using tandem mass spectrometry

**DOI:** 10.1038/s41598-020-59156-3

**Published:** 2020-02-10

**Authors:** Sakshi Rajoria, Sasikumar Sabna, Prabhakar Babele, Ravi Bhushan Kumar, Dev Vrat Kamboj, Subodh Kumar, Syed Imteyaz Alam

**Affiliations:** 1 Biotechnology Division, Defence Research & Development Establishment, 474002 Gwalior, India; 2 Microbiology Division, Defence Research & Development Establishment, Gwalior, India

**Keywords:** Bioinformatics, Environmental impact

## Abstract

Some pathogens and toxins have the potential to be used as weapons of mass destruction and instigate population-based fear. Efforts to mitigate biothreat require development of efficient countermeasures which in turn relies on fast and accurate methods to detect the biological agents in a range of complex matrices including environmental and clinical samples. We report here an mass spectrometry (MS) based methodology, employing both targeted and shot-gun approaches for the verification of biological agents from the environmental samples. Our shot-gun methodology relied on tandem MS analysis of abundant peptides from the spiked samples, whereas, the targeted method was based on an extensive elucidation of marker proteins and unique peptides resulting in the generation of an inclusion list of masses reflecting relevant peptides for the unambiguous identification of nine bacterial species [listed as priority agents of bioterrorism by Centre for Disease Control and Prevention (CDC)] belonging to phylogenetically diverse genera. The marker peptides were elucidated by extensive literature mining, *in silico* analysis, and tandem MS (MS/MS) analysis of abundant proteins of the cultivated bacterial species in our laboratory. A combination of shot-gun MS/MS analysis and the targeted search using a panel of unique peptides is likely to provide unambiguous verification of biological agents at sub-species level, even with limited fractionation of crude protein extracts from environmental samples. The comprehensive list of peptides reflected in the inclusion list, makes a valuable resource for the multiplex analysis of select biothreat agents and further development of targeted MS/MS assays.

## Introduction

The realization that that some pathogenic microbes and toxins of biological origin can be used as weapons of mass destruction has gained prominence especially in the backdrop of growing concerns for bioterrorism. Biological select agents are derived from biological sources, are capable of causing significant damage to human health and safety, and can be overtly or covertly used for ideological, political, or financial gain^1^. U.S. Department of Health and Human Services (HHS) and the U.S. Department of Agriculture (USDA) have prioritized these select agents^2^ and CDC has classified the agents of bioterrorism under the categories A through C^3^. It includes several toxins, bacteria, and viruses that are prioritized based on their high morbidity and mortality, dose of infections, environmental stability, production ease, and lack of an established countermeasure. Our vulnerability to biological warfare agents was realized after the anthrax letter attack of 2001^4^; the mortality rate of inhalational anthrax is 100% when left untreated. The high-consequence bacterial biowarfare (BW) agents encompass wide phylogenetic lineages including the causative agents of plague (*Yersinia pestis*), anthrax (*Bacillus anthracis*), brucellosis (*Brucella* spp), and tularaemia (*Francisella tularensis*). Missile warheads, manned or unmanned spray-tanks coupled to the aircraft, and aircraft capable of delivering biological agents are some of the possible means of delivery of these biological agents for clandestine applications intended to damage human, animal, and plant resources. Aerosol is the most likely and destructive dissemination mode in this regard^5^.

Efforts to mitigate biothreat require development of efficient countermeasures including effective treatment and decontamination. The medical countermeasures in such a scenario will be heavily dependent on rapid methods to detect the biological agents in a range of complex matrices including environmental and clinical samples. In this regard, complexity of matrices, such as environmental soil samples, is a major confounding element resulting in prohibitively significant background signals and false-positive/negative results^6,7^. In the clinical context and with additional concerns (e.g. resistance to antibiotics, virulence, and emerging pathogens), early detection of the agent and disease diagnosis greatly helps prognosis and selection of a potential therapeutic measure^8,9^.

Various analytical methods have been used in the recent past to identify agents of bioterrorism; including nucleic acid based assays, such as polymerase chain reaction and protein based immunological assays, for example enzyme-linked immunosorbent assays, the latter applicable for both protein toxins and microorganisms^10–17^. Although, of immense value for the rapid screening of samples, these methods suffer limitations pertaining to depth of analysis, suitability for unknown and advanced biological agents, and lack of specificity (for example due to cross-reactivity with similar molecules), posing a risk of the false positive and ambiguous results. Recent developments in the MS technology promise un-equivocal identification of microorganisms with remarkable specificity, speed, and sensitivity. The multiplexing capability and suitability for analyzing complex samples such as air, water, culture medium, bodily fluids, and food are of immense value for the utility of MS based techniques in biothreat scenario. MS platforms such as Matrix-assisted laser desorption-ionization time-of-flight mass spectrometry (MALDI-TOF-MS) or liquid chromatography tandem mass spectrometry (LC-MS/MS) are gaining popularity in microbiological research, largely for diagnostic applications, using mass fingerprinting or peptide sequencing approaches^18^.

MALDI-TOF MS is emerging as a rapid, robust, and cost-effective technology for a routine bacterial identification method, largely in clinical diagnostic laboratories^19–22^. For example, MALDI Biotyper and VITEK MS have been approved by the U.S. Food and Drug Administration for bacterial identification in clinical diagnostic laboratories^23–26^. Both these MALDI-TOF-MS-based platforms ionize intact proteins extracted from whole cell culture without specific protease treatment^19,27^. However, this approach not only requires pure cultures to produce stable and consistent result^23,24^, culture conditions may still affect the quality of the spectra and the final identification^25^. Moreover, most of the MS based detection methodologies reported till now, rely on an in-house database and analysis of MS spectra of intact proteins with very limited or no peptide sequence information; the strength of MS/MS analysis that provides sequence information at the highest resolution is missing in these approaches. Utility of MALDI-TOF-MS method for the species level identification of high-consequence bacterial pathogens was found to be challenging due to mass spectral profile database content and quality^28^. The single MS approaches also suffer serious limitations for analysis of complex microbial cultures, detection of microorganisms in food and environmental samples, in situations where the organism is non-cultivable or fastidious, and for identifying rare pathogenic bacteria or novel-emerging pathogens^29^. MS/MS is capable of generating sequence information and therefore can significantly improve the specificity for the identification of biological agents based on the detection of unique tryptic peptides^30–36^. For example, a combined bottom-up and top-down proteomics methodology was used, employing a high-resolution/high mass accuracy LTQ-Orbitrap instrument to identify specific markers of *B. anthracis* spores, capable of discriminating closely related *Bacillus cereus* biovar anthracis CI, CA, and *Bacillus thuringiensis* 9727^37^. Proteotypic peptides are classically defined as “the peptides that uniquely identify each protein and are consistently observed when a sample mixture is interrogated by a (tandem) mass spectrometer”^38^. There are a few software applications available for the in silico digestion and predictive elucidation of proteotypic peptides for the targeted proteomic analysis^39–42^. For example, Skyline software aids selection of proteotypic peptides for the MRM assay development for the quantitative analysis of target proteins (https://sciex.com/products/software/skyline-software). Similarly, PeptideManager is a tool for the selection of peptide for the targeted proteomic analysis for mixed samples originating from different species^40^. However, these methodologies select peptides for proteins from a target proteome (e.g., human) and ensure specificity against a background of host proteome (e.g., mouse, rat)^43^. Mesuere *et al*.^43^ have recently reported a web application, The Unique Peptide Finder (http://unipept.ugent.be/peptidefinder), to screen for taxon-specific tryptic peptides.

Though beginning to be recognized, the reports on application of MS-based methodology in biothreat scenario are scanty: among the first of the applications, Dworzanski *et al*.^44^ demonstrated discrimination and classification of the closely related strains of *B. anthracis*, *B. cereus*, and *B. thuringiensis*. Using tandem MS approach, we have previously reported recovery and analysis of biological agent after bioaerosol exposure in a simulated scenario of biothreat^6^. Using a poly-disperse disseminator in the experimental set-up, the importance of the choice of matrix was highlighted for a post-attack identification after recovery of the biological agent. We recommended to use matrices with minimal interfering substances (e.g. foliage, smooth surfaces, sand) for verification of the bio-agent in a biothreat scenario, retrospectively. Complex environmental matrices (e.g. soil) are challenging for the said purpose as the high background signal hinders assays and lead to false- positive/negative results^7^. We report here a comprehensive elucidation of marker proteins and species specific unique peptides for the targeted tandem MS analysis of bacterial select agents. The targeted approach described here is distinct from the well-known multiple reaction monitoring (MRM) assays and involves selection of several unique peptide masses for the tandem MS analysis after the first MS scan. The MS based methodology employing both targeted and shot-gun approaches for the verification of biological agents from the environmental samples is demonstrated here. The targeted approach is intended to screen for the bacterial agent from a pre-defined list of pathogens using selected unique marker peptides, whereas, the shot-gun methodology aims at generating sequence information for as many peptides as possible and to identify even the unknown or advanced biothreat agents. Our shot-gun methodology relied on tandem MS analysis of abundant peptides from the spiked samples, whereas, the targeted method was based on an extensive elucidation of marker proteins, unique peptides, strain coverage analysis, and generation of an inclusion list of masses reflecting relevant peptides for the unambiguous identification of selected bacterial biowarfare agents. The comprehensive list of peptides reflected in the inclusion list, makes a valuable resource for the multiplex analysis of select biothreat agents and further development of targeted tandem MS assays. The species-specific marker peptides for the nine bacterial select agents were elucidated by extensive literature mining, *in silico* analysis, and MS/MS analysis of abundant proteins from axenically grown bacterial species in our laboratory. As a proof of concept, the methodology was validated using blind samples spiked with one or two select agents spiked in soil and sand.

## Results

### Identification of abundant proteins from select bacterial agents

Abundant proteins from *B. anthracis* Sterne strain, *Clostridium perfringens* ATCC13124, *Burkholderia pseudomallei*, *Brucella abortus* strain NCTC 10093, and *Clostridium tetani* strain drde were identified by MS/MS analysis of whole cell lysate. Cells were axenically grown on rich medium and total protein was extracted as described in the methods. The proteins were separated on SDS-PAGE and abundant protein bands (20–40) were excised from each lane. After tryptic digestion of excised bands, proteins were identified by MALDI-TOF-TOF analysis and subsequent MASCOT search against the taxonomic group of bacteria as described in the methods sections. Results obtained from two such independent analysis, were combined together and the proteins identified in the corresponding species with significant MS/MS ion score of peptides are listed in Supplementary Table S1 A–F. On several occasions, more than one proteins from the same species were identified from a single spot, as there could be multiple co-migrating proteins on single dimension of SDS-PAGE. Only for *C. botulinum*, MS/MS data for different botulinum neurotoxin (BoNT) serotypes were compiled from reported literature. This way, 60 non-redundant proteins from *B. anthracis* Sterne strain (184 peptides), 30 from *B. abortus* (176 peptides), 12 from *B. pseudomallei* (73 peptides), 48 *C. perfringens* (211 peptides), and 59 proteins from *C. tetani* (227 peptides), were identified with significant MASCOT score. The peptides identified with significant MS/MS ion score from crude lysate of cells with minimal fractionation indicated their abundance and amenability for MS analysis, in turn their suitability as markers. All peptides observed here were subjected to global protein BLAST against the GeneBank non-redundant protein database with search parameters adjusted for short input sequence and those showing less than 100% sequence identity with any species other than the target species were noted. Representative MS and MS/MS spectra for three proteins have been shown in Supplementary Fig. S1.

### Elucidation of putative protein markers for detection

The following parameters were considered for qualifying a protein as putative marker for a pathogenic species: (1) an evidence of expression indicating abundance, supported by the number of scientific reports; (2) identification of the protein in crude cell lysate using MALD-MS/MS analysis with significant ion score for peptides; and (3) a low percent identity of the protein with its closest homolog in any other bacterial species. From the bacterial select agents listed by HHS and USDA as priority agents of bioterrorism, nine bacterial species belonging to diverse genera were selected for the analysis. This included *Brucella suis* [BSU], *B. abortus* [BAB], *Brucella melitensis* [BMT], *B. pseudomallei* [BP], *Burkholderia mallei* [BM], *C. perfringens* [CP], *Clostridium botulinum* [CB], *C. tetani* [CT], and *B. anthracis* [BA].

Evidences for the abundance and expression of different proteins in a given species were screened by extensive literature mining at PUBMED (www.ncbi.nlm.nih.gov) using set of appropriate keywords, one after the other. Any protein for a given species reported in at least three independent publications was selected and its attributes with respect to immunogenicity, immuno-dominance in naturally infected clinical sera, immuno-protection, and role in virulence were noted. The short-listed proteins were subjected to global protein BLAST against the non-redundant protein database at National Center for Biotechnology Information (NCBI) using the FASTA sequences for each of the proteins retrieved from the reference strain of the selected bacterial species (Supplementary Table S2 A–F). Localization of the protein was noted from the experimental reports during literature survey and also predicted by PSORT algorithm at ExPASy Proteomics tools (http://www.expasy.ch).

**Figure 1:**
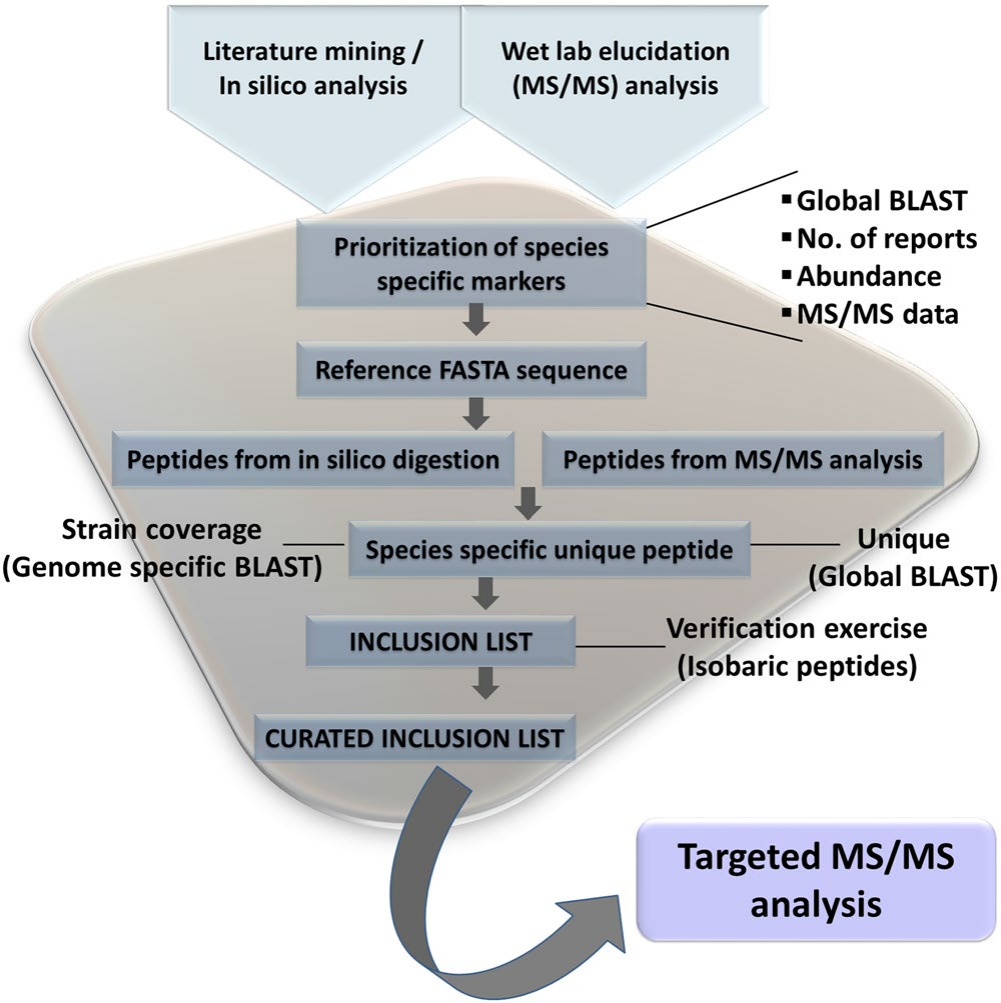
Schematic representation of the workflow for elucidation of marker peptides for targeted analysis of selected bacterial biowarfare agents.

**Figure 2:**
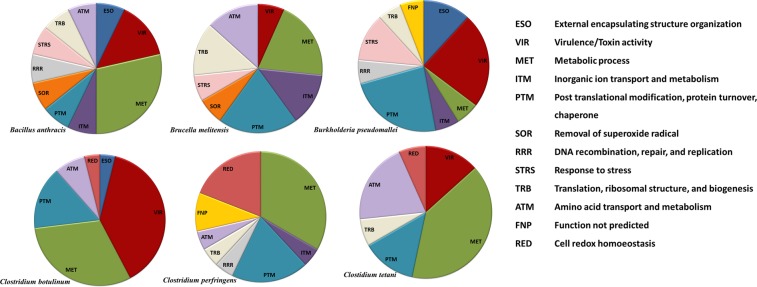
Functional classification of proteins short-listed as putative marker for targeted detection of selected bacterial biowarfare agents.

As described in the methods, we used our own ranking scheme to prioritize candidate protein markers for each of the selected species by taking into consideration the three parameters mentioned above: the number of reports for the expression of a given protein and th–e number of peptides with significant MS/MS ion score in the experimental identification of abundant proteins in the laboratory were assigned 25 points each, whereas the percent identity with the nearest homolog was given 50 points. Low percent identity with the nearest homolog in any other species was given maximum weightage as it entails a better chance of scoring unique peptides for the given species for the targeted MS based assay development. For instance, a sequence showing 100% identity with the nearest homolog in any other species was assigned 0 point (100–100), while 50 points for the identity parameter was given to a sequence with 50% identity (100–50). Abundant proteins identified from the whole cell lysate of selected bacterial species were assigned 25 points: 5 points for every peptide with significant MS/MS ion score, 1 peptide was given 5 points and 5 additional points were given to every additional peptide up to a maximum of 5 peptides (≥5 peptides given complete 25 points). These peptides from the tryptic digest of minimally fractionated crude lysates indicated their abundance and amenability for tandem MS analysis, further augmenting their potential as markers for unambiguous detection. The number of times a given protein was reported for a particular species as expressed, in independent studies, was considered a proof of its abundance and putative role in virulence was given priority to improve specificity to discriminate closely related species or to differentiate between pathogenic and saprophytic strains of the species. We assigned 5 points for 3 reports and 5 additional points for every additional report up to 7 (≥7 reports for the given protein assigned complete 25 points). This priority protein list was appended with pathogen specific virulence associated proteins from the reported literature, if not selected at this stage. Supplementary Table S2 A–F, lists 15–27 putative proteins markers for each of the selected bacterial species in the decreasing order of the weightage assigned according to the scheme described above. At this stage, the closely related species of *Burkholderia* (*B. pseudomallei* and *B. mallei*) and *Brucella* (*B. melitensis*, *B. suis*, and *B. abortus*) were considered together. Supplementary Table S1 (A–F) also reflects: maximum number of times any peptide of the protein was observed during MS/MS analysis in the present investigation using crude whole cell lysate of the organism; the number of reports for expression; predicted localization (PSORT); percent identity with the nearest homolog; and its role with respect to virulence, immunogenicity, vaccine potential and experimentally determined localization. The number of proteins shortlisted at each of the steps mentioned above for the selected taxa is summarized in Table 1 and the schematic workflow is reflected in Fig. 1. The predicted molecular function/biological process for the putative marker proteins for the selected bacterial agents indicated a predominance of proteins involved in: post-translational modification, protein turnover and chaperone function; metabolism; and those related to virulence (Fig. 2). Supplementary Table S3 summarizes the selected putative marker proteins for specific detection of target bacterial agents of BW significance with brief molecular function/biological process, predicted localization, and other functional attributes experimentally demonstrated by different groups. These putative markers (19 for *B. anthracis*, 20 for the three closely related species of *Brucella*, 16 for *B. pseudomallei* and *B. mallei*, 27 for *C. botulinum*, and 21 for *C. perfringens*, and 15 for *C. tetani*) are arguably expressed by the selected pathogenic species and are distantly related to their homologs in other bacterial species. The details of the in silico methods for selection of the marker proteins is presented in Supplementary Material S6.

**Table 1:** Elucidation of protein markers for unambiguous detection of selected bacterial biowarfare agents. BMT represents all three closely related species of *Brucella* (*B. melitensis, B. abortus*, and *B. suis*) recognized as agents of bioterrorism by CDC, Atlanta. BM = *B. mallei* and *B. pseudomallei*; BA = *B. anthracis*; CP = *C. perfringens*; CT = *C. tetani*; CB = *C. botulinum*.

	BMT	BM	BA	CP	CT	CB
Literature mining for evidence of expression	263	524	566	184	56	105
Proteins with ≥3 reports of expression	67	24	48	31	6	51
Proteins identified in whole cell lysate (MS/MS)	30	12	60	48	58	6
Overlapping proteins (wet-lab/data mining)	10	7	26	24	6	6
Appended with virulence associated proteins	10	10	10	10	10	10
Priority proteins for targeted analysis	16	16	15	21	15	27

### Elucidation of unique peptides and generation of inclusion list

The FASTA sequences of the selected putative marker proteins were subjected to *in silico* tryptic digestion and the peptides in the mass range of 1000–3000 Da were subjected to global protein BLAST against the GeneBank non-redundant protein database with search parameters adjusted for short input sequence. Peptides showing less than 100% sequence identity with any other species, indicating a difference of at least one amino acid residue and as consequence unique precursor mass, were listed as unique. Apart from the peptides selected as unique for the target species, peptides were also selected that were specific to the very closely related species within the *B. cereus* sensu lato group, including eleven other species (*B. cereus*, *Bacillus mycoides*, *Bacillus pseudomycoides*, *B. thuringiensis*, *Bacillus weihenstephanensis*, *Bacillus cytotoxicus*, *Bacillus wiedmannii*, *Bacillus toyonensis*, *Bacillus bingmayongensis*, *Bacillus gaemokensis*, and *Bacillus manliponensis*) with a tolerance for one or two species. This way, the final inclusion list for *B. anthracis* included those unique to the species, specific to the *B. cereus* sensu lato group without or with one or two exception, and those abundant in the species to aid a comprehensive identification. Only a few peptides could be scored specific for the closely related species of *B. mallei* and *B. pseudomallei*; the two species were considered together for the peptide BLAST search and scoring unique peptides. In order to aid a comprehensive identification, the final inclusion list for *B.mallei* and *B. pseudomallei* included those unique to the species, specific to both the target species without or with one or two exception, and those abundant in the species.

All the peptides selected for the three closely related species of *Brucella* (*B. melitensis, B. abortus*, and *B. suis*) were further screened for species specific peptides after downloading all the available sequences of putative marker proteins using species specific genome BLAST (keeping specific species as target organism). The FASTA sequences were sorted into three groups, corresponding to each of the taxa (*B. melitensis, B. abortus*, and *B. suis*). This resulted in the list of peptides for the three species of *Brucella* including those unique to one, two or all the three species (*B. melintensis*, *B. abortus*, and *B. suis*, as indicated in superscript as M, A, and S) without or with one or two exception, and those abundant in the species to aid a comprehensive identification (Supplementary Table S4 A–G).

In order to avoid false positives for the intended analysis of environmental samples, we deducted isobaric peptides obtained from tryptic digest of bacterial consortium on MS scan using the inclusion list for all the peptides for the selected agents (0.5 Da window) and MS/MS analysis of selected precursors. The curated list of marker peptides and their corresponding masses are shown in Supplementary Table S4 A–G. The unique peptides in the inclusion list were further screened for strain coverage using all the available sequences retrieved by species specific BLAST search and counting the number of strains from the alignment data for the conservation pattern of the selected peptides in diverse strains in the genome data base (Supplementary Table S4 A–G). The details of the in silico methods used for the elucidation of species specific peptides is described in Supplementary Material S6.

### Proof-of-concept studies

The shot gun and targeted search methodologies using the inclusion list of peptides described here were used in the proof-of-concept experiment using number coded blind samples. The results of a representative analysis using eight number-coded blind soil and sand samples spiked with either or both of *B. anthracis* Sterne strain and *C. perfringens* ATCC13124 is shown in Supplementary Table S5. Garden soil and sand were either blank (not spiked with any agent) or spiked with cell suspension [10^6^ *C. perfringens* ATCC13124 or 10^7^ *B. anthracis* Sterne colony forming units per gram (cfu.g^−1^) of matrix] of one or two bacterial agents and dried overnight at 37 ^o^C in order to simulate field conditions under which the agents are likely to bind to the matrix particles as a result of desiccation or other environmental factors. When both the agents were spiked, *C. perfringens* was added at a concentration one log lower (10^6^ cfu.g^−1^) than *B. anthracis*. The analyst was provided the number coded blind samples to carry out the identification of a pathogenic bacterial species. Total protein was extracted as described in the methods and subjected to minimal resolution on SDS-PAGE. Ten abundant protein bands from each sample (except for the two blank samples) were excised from the SDS-PAGE gel (Supplementary Fig. S2). The gel pieces were subjected to tryptic digestion before loading onto MALDI target plates for tandem MS analysis by MALDI-TOF-TOF using both shot-gun and targeted acquisition from replicate spots. Agents were correctly identified in all the samples, except for the mixture of two bacterial agents where *C. perfringens* (at one log lower concentration), was missed, perhaps due to masking of the proteins on 1D gel and for only a limited fraction of proteins (10 bands) being analyzed (Supplementary Table S5). For *B. anthracis*, other species within the *B. cereus* sensu lato group also exhibited significant match in the MASCOT search. However, the decision regarding *B. anthracis* could easily be taken for the number of places it ranked at the top of the matched species. Limited number of peptides from the inclusion list was seen in the targeted approach due to the limited number of protein bands sampled. The number of peptides matched (MP) to the identified protein during MASCOT search using MS data and the MASCOT scores are also shown in the Supplementary Table S5. MSCOT ion score for the MS/MS analysis of the fragmentation data of peptides showing significant ion score and the RMS error indicate ability of the proposed methodology to correctly identify the biological agent even with the limited sampling of the protein bands from the extracted proteins. MS and MS/MS spectra of the tryptic digest of three representative proteins from this study are shown in Supplementary Fig. S4.

## Discussion

Mitigation of bio-threat requires rapid and accurate methods to detect the biological agents in a range of complex environmental and clinical matrices. The study reported here describes a comprehensive MS based methodology employing both targeted and shot-gun approaches for the verification of biological agents from the environmental samples in a biothreat scenario. The shot gun approach aims at generating sequence information for as many peptides as possible and attempting to identify even the unknown or advanced pathogens, taking advantage of the ever expanding protein database and search engines for MS and MS/MS data interrogation. Our shot-gun methodology relies on tandem MS analysis of abundant peptides from the spiked samples, whereas, the targeted method is based on an extensive elucidation of marker proteins and unique peptides. This resulted in the generation of an inclusion list of masses reflecting relevant peptides for the unambiguous identification of bacterial select agents belonging to diverse genera including nine bacterial species. The comprehensive list of peptides reflected in the inclusion list (Supplementary Table S4), makes a valuable resource for the multiplex analysis of select biothreat agents. This marker peptide list was elucidated by extensive literature mining, *in silico* analysis, and MS/MS analysis of abundant proteins of the cultivated select bacterial species in our laboratory. The methodology was validated using blind soil and sand samples spiked with one or two select agents.

MALDI-TOF MS has been used for the detection of microorganisms and toxins in environmental and food samples using both bottom-up and top down approaches after cultivation of microorganisms for enrichment and separation^45–47^. Shotgun proteomics has shown promising results in describing a large panel of virulence factors in several pathogens including the analysis of exoproteome from *B. cereus*^48^. Lasch *et al*.^49^ analyzed cultivated strains of *Bacillus* and related genera including those of *B. anthracis* and *B. cereus* by MALDI-TOF MS revealing mass spectral peaks specifically belonging to *B. anthracis* species. A rapid MALDI-TOF MS method was developed for identification of *B. anthracis* spores and its differentiation from closely related *B. cereus* in powder samples, with a limit of detection of 2.5 × 10^6^ spores^46^. Ten distinct pathogenic bacterial samples including *B. anthracis* and *Y. pestis* were used in an inter-laboratory ring trial from nine European countries: accurate microbial identification relied on the comprehensive spectral databases used^50^. There are reports where intact protein biomarkers have been defined by proteogenomics in order to differentiate microorganisms and discriminate pathogens of a given genus or clade^51–53^. However, the single MALDI-MS approach suffers several limitations due to lack of peptide sequence information, need of pure cultures to produce consistent results, and effect of culture conditions on spectral quality and ensuing identifications^23–25^. The inability to differentiate high-consequence pathogens from related bacterial species can be a serious issue for clinical diagnostic laboratories and more so in a biothreat perspective^28,50,54^. Incorrect identifications with single MALDI-TOF MS methods have resulted in laboratory exposures with *Francisella tularensis*^55^ and *B. pseudomallei*^56^.

We describe here the use of tandem mass spectrometry (MALDI-TOF-TOF) to seek sequence information for multiple target proteins for the detection of bacterial pathogens of BW significance from diverse phylogenetic clades. The putative marker proteins were judiciously selected to impart selectivity by employing a ranking scheme to prioritize candidate protein markers for each of the selected species by taking into consideration, the three parameters described in the results. Extensive literature mining provided strong evidence of expression indicating abundance and role in virulence at the experimental level as indicated by the number of scientific reports (Supplementary Table S2 A–F). The identification of the abundant proteins, from the selected bacterial species using MALDI-MS/MS analysis in the minimally fractionated crude cell lysate, with significant ion score for peptides indicated abundance and amenability of the peptides for the tandem MS analysis (Supplementary Table S1 A–F). A low percent identity of the protein with its closest homolog in any other bacterial species was given maximum weightage for an increased likely hood of getting species specific unique peptides (Supplementary Table S5). Taking advantage of the sequence information and to improve the specificity, bottom-up analysis using MALDI-TOF(/TOF) MS and electrospray ionization ESI-MS(/MS) methods have been reported for the identification of toxins and microorganisms, based on the detection of unique tryptic peptides^30–37^. Tandem MS approaches, providing peptide amino acid sequence information, are more often obtained with LC-MS/MS systems for crude proteins: the tryptic digest of whole proteome is separated by HPLC and the ionized peptides are detected and further fragmented to obtain peptide sequences by comparing the tandem spectra to the theoretical peptide fragmentation spectra. For example, a combined bottom-up and top-down proteomics methodology was used, employing a HR/MS LTQ-Orbitrap instrument, to elucidate specific markers of *B. anthracis* spores capable of discriminating closely related *B. cereus* biovar anthracis CI, CA, and *B. thuringiensis* 9727^37^. Employing a statistical scoring algorithm against a fully sequenced database of 170 bacterial genomes, identification of bacteria has been reported using tandem MS data obtained with bacterial proteins extracts digested with trypsin^57^. Although restricted to the 170 sequenced organisms, the method was shown to be reliable in strain level identification of bacteria when enough peptide information was collected.

Our study represents perhaps the broadest and most comprehensive list of unique peptides for the targeted verification of bacterial select agents including *B. suis*, *B. abortus*, *B. melitensis*, *B. pseudomallei*, *B. mallei*, *C. perfringens*, *C. botulinum*, *C. tetani*, and *B. anthracis*. Using *C. botulinum* as a model, Al-Shahib *et al*.^58^ reported biomarker discovery and validation using mass spectrometry and bioinformatics. However, the validation studies were carried out on the peptides unique to *C. botulinum* strains by searching with phylogenetically related *Clostridium* species and not using a global BLAST analysis. Although, there are a few software applications recently developed for the in silico digestion and elucidation of proteotypic peptides for the targeted MS based analysis, our definition of species specific proteotypic peptide is broader in specificity and relies on global identity with all known sequences in the protein database at NCBI (www.ncbi.nlm.nih.gov)^44–49^. This approach, employing global BLAST search for each peptide from the list of *in silico* digested putative markers is likely to provide subtle advantage in imparting specificity of identification of biowarfare agents in a possible biological emergency. Sensitivity of detection in a biothreat scenario is of lesser importance than the accuracy of agent verification as ambiguity of identification has got serious implications for the execution of medical countermeasures; selection of a potential therapeutic and prophylactic agent relies on unambiguous agent identification. Mathematical models were used for estimating dosage and casualty that is likely to result from the release of aerosol near ground level from a single point by an aircraft. Notably, it is estimated that in an area of 100 km^2^, several kilograms of bio-agent need to be released for a substantial effect on a population^5,59^. In the recent past, selective and sensitive quantitation of protein candidates by targeted analysis has been accomplished by selected reaction monitoring (SRM) methods coupled to isotope dilution^60^. The MRM assays on LC-MS/MS are rapid and provide multiplexing capabilities for several target proteins: ion selection and scoring for daughter ions after fragmentation (MRM transition) is used to identify and quantify molecules of interest per sample run. There are reports for the specific and sensitive detection and quantification of toxins and pathogens from environmental and food matrices, including *B. anthracis* spores and *Y. pestis*^36,37^. Immunocapture of intact cell has often been combined with LC/SRM, to improve sensitivity^36,46^. However, the SRM acquisition has an intrinsic limitation of selectivity due to low resolution of the mass analyzers (quadrupole) used for mass selection resulting in bias with multiplex targets. Recent developments in high-resolution accurate-mass acquisition (HR/AM) instruments have been used to overcome this limitation with improved selectivity due to enhanced capability for the separation of ions of interest from interfering moieties, especially in complex biological samples^61^. A multiplexed and targeted analysis 109 peptides from 27 *Staphylococcus aureus* proteins has been reported using LC–SRM approach for the species level identification and additional information pertaining to antibiotic resistance, virulence factor detection (e.g. toxins involved in toxic shock syndrome), and epidemiological typing (e.g. Protein A peptides)^62^. It is important to note that this SRM assay requires ≥10^7^ cfu for identification and a preliminary culture step is required.

The inclusion list peptides described here for the nine bacterial select agents, were derived from rationally selected protein markers. The tryptic peptides of these putative marker proteins were subjected to rigorous screening by global BLAST search against the huge protein data base to ensure maximum representation of unique peptides from multiple target proteins of a given species. The peptides were further screened for the strain coverage and curated to reduce background signal by deducting isobaric peptides from the environmental bacterial consortium. Although reflected together in Supplementary Tables S2 and S3, the putative marker proteins were searched independently for the closely related species of *B. pseudomallei*, *B. mallei* and for the very related three species of *Brucella* (*B. melitensis*, *B. suis* and *B. abortus*). Although, discrimination of these very closely related species is challenging, a combination of shot-gun tandem MS analysis and a targeted search using a panel of unique peptides is likely to provide unambiguous verification of biological agents even at sub-species level, even with limited fractionation of crude protein extracts from the environmental samples. However, the approach based on the species specific unique peptides should be continuously monitored and updated, as sequencing of novel species or strains whose protein sequences differ from those in public databases can potentially erode some of the signatures. As several proteins and peptides are being considered for the targeted analysis here, the signature erosion by discovery of novel taxa are less likely to grossly influence the identification. Although, only unique peptides showing less than 100% sequence identity with any other bacterial species (except the query species) were selected for the inclusion list of unique peptides; it was based on the assumption that a difference of even one single amino acid is most likely to ensure that the selected mass will not come from any other species except the target taxon. Nevertheless, a very low possibility of getting a non-specific isobaric peptide with same amino acid composition but differing in sequence does exist. The proof-of-concept study reported here shows the applicability of the method for the verification of the biological agent. Further studies are needed before the characteristics of the assay reported here are known; rigorous validation study would include many more samples and replicates, include all the agents for which the targeted assay is intended with positive and negative controls and, a limit of detection analysis, and false negative and false positive rates. A detailed validation exercise, when carried out with the state of art LC interface (e.g. two dimensional nano-LC), would likely provide deeper analysis and high resolution data. The data-independent acquisition (DIA) methods, such as SWATH-MS, are emerging technologies that enables deep proteome coverage and consistent quantitation, and accuracy^63^. The inclusion list of peptides reported here can be of immense use in other platforms of high resolution mass spectrometry (e.g. SWATH-MS) for targeted analysis of bio-threat agents.

## Conclusions

The realization that that some pathogenic microbes and toxins of biological origin can be used as weapons of mass destruction has gained prominence especially in the backdrop of growing concerns for bioterrorism. Efforts to mitigate biothreat require development of efficient countermeasures which in turn relies on fast and accurate methods to detect the biological agents in diverse complex matrices including environmental and clinical samples. We report here an MS based methodology, employing both targeted and shot-gun approaches for the verification of biological agents from the environmental samples. Our shot-gun methodology relied on tandem MS analysis of abundant peptides from the spiked samples, whereas, the targeted method was based on an extensive elucidation of marker proteins, unique peptides, strain coverage analysis and generation of an inclusion list of masses reflecting relevant peptides for the unambiguous identification of bacterial select agents belonging to the diverse taxonomic groups. The comprehensive list of peptides reflected in the inclusion list, makes a valuable resource for the multiplex analysis of select biothreat agents, and was elucidated by extensive literature mining, *in silico* analysis, and MS/MS analysis of abundant proteins of the selected cultivated bacterial species in our laboratory. A combination of shot-gun tandem MS analysis and a targeted search using a panel of unique peptides is likely to provide unambiguous verification of biological agents even at sub-species level, even with limited fractionation of crude protein extracts from environmental samples. The validation study reported here is just a proof of concept to show the applicability of the method, as deeper analysis can be carried out using the current HR-MS platforms with high end LC interface (e.g. two dimensional nano-LC). The inclusion list of peptides reported here can be of immense use in other recent platforms of high resolution mass spectrometry (e.g. SWATH-MS) for targeted analysis of bio-threat agents.

## Materials and Methods

### Bacterial strains and growth conditions

The following strains of bacteria were used for the elucidation of abundant proteins and their peptides by MS/MS analysis of tryptic digest of proteins in the laboratory: *B. anthracis* Sterne strain, *Clostridium perfringens* ATCC13124, *Burkholderia pseudomallei* strain B50, *Brucella abortus* strain NCTC 10093, and *Clostridium tetani* strain drde. A high containment facility (biosafety level 3) was used for the cultivation of bacterial cultures and preparation of whole cell protein lysate and the protocols were approved by institutional biosafety committee of Defence Research and Development Establishment, Gwalior, India. Non-pathogenic *B. anthracis* Sterne strain was obtained from Institute of Veterinary and Preventive Medicine, Ranipet, Vellore, India and grown at 37 ^o^C on Brain Heart Infusion (BHI) broth containing calf brain infusion, 12.5 g; beef heart, 5 g; peptone, 10 g; NaCl, 5 g; D-glucose, 29 g; Na_2_HPO_4_, 2.5 g; and distilled water 1 L. *C. perfringens* ATCC13124 was obtained from Becton Dickinson India Pvt. Ltd., India and grown anaerobically in modified reinforced clostridial medium (RCM) broth containing 10 g of peptone, 10 g of beef extract, 3 g of yeast extract, 5 g of dextrose, 5 g of NaCl, 3 g of cysteine HCl, 0.5 g of sodium acetate, and 1000 ml distilled water. *C. tetani* drde strain was isolated previously from decaying fish sample at DRDE, Gwalior, India and was anaerobically grown in trypticase peptone yeast extract glucose (TPYG) broth containing pancreatic digest of casein, 50 g; peptone, 5 g; yeast extract, 20 g; glucose, 4 g; sodium thioglycollate, 1 g; NaCl, 2 g; soluble starch, 1 g; cycloserine, 250 mg; sulphamethoxazole, 76 mg and trimethoprim, 4 mg per litre. *B. pseudomallei* strain B50 is a clinical isolate retrieved from our own culture collection at DRDE, Gwalior, India and the bacterium was axenically grown in LB broth at 37 °C. *B. abortus* strain NCTC 10093 was grown in Brucella broth with 5% CO_2_ at 37 °C.

### Reagents and chemicals

Analytical grade chemicals were procured from Sigma-Aldrich (Sigma-Aldrich Chemicals Pvt. Ltd., New Delhi, India), unless specified otherwise. Tryptic digestion was carried out using sequencing grade modified trypsin from Promega (Promega, Madison, WI, USA). Mass standards for MALDI-TOF-TOF analysis were obtained from Applied Biosystem, USA^34^. The culture media were procured from; Oxoid Ltd., England, or Difco Laboratories, France, or HiMedia, India.

### Extraction of total cellular protein

Bacterial strains were axenically grown in rich media, till the exponential phase, for the preparation of whole cell proteome as described above. Cells were harvested by centrifugation at 8,000 × g for 30 min at 4 °C, followed by a washing step with 20 mM Tris.HCl buffer (pH 7.5). Extraction buffer (2% SDS, 100 mM DTT, 20 mM Tris-HCl pH 8.8) was added to the cell pellets and incubated for 20 min at at 95 °C. After centrifugation at 20,000 × g for 10 min at 10 °C, the supernatant was collected and the protein in solution was precipitated with trichloroacetic acid (TCA) at a final concentration of 10% (w/v) in the presence of β-mercaptoethanol (0.07%). After an incubation of 2 h on ice, the protein pellet was collected by centrifugation (10,000 × g, 4 °C, 20 min), washed twice with 500 μL of acetone, and air-dried for 5–10 min, and stored at −80 °C till use^34^. Using the method described earlier, the protein pellet was solubilised with 8 M urea and 2% w/v CHAPS and loaded onto a discontinuous denaturing SDS PAGE (12%) after an additional heating at 95 °C for 5 min with SDS lysis buffer [12.5 mM Tris-HCl (pH 6.8), 4% glycerol (w/v), 0.4% SDS (w/v), and 1% β-mercaptoethanol (v/v)]^34^. Quick Start Bradford Protein Assay kit (Bio-Rad) was used for the determination of protein concentration as per manufacturer’s instructions using BSA as standard.

For the viualisation of protein bands, gels were stained with Coomassie Brilliant Blue G-250 and gel pieces were excised from the abundant protein bands (20–40) using thin walled microfuge tubes, cut at the tip with an approximate 2 mm diameter.

### In-gel protein digestion

The excised gel pieces were destained with three washes of 200 µL 50% ACN/50 mM NH_4_HCO_3_ for 30 min each, at room temperature. After reduction with 10 mM DTT, the proteins were alkylated using 50 mM iodoacetamide, the gel pieces were dried. Trypsin (100 ng) in 50 mM NH_4_HCO_3_ was added to the gel pieces and digestion was carried out overnight at 37 ^o^C. Using 60% ACN and 0.1% TFA, the peptides were extracted and dried. MALDI-MS/MS analysis was carried out after resuspending the peptides in 0.5% TFA.

### MALDI-TOF-TOF analysis of tryptic protein digests

Abundant proteins from total cellular protein fraction from five bacterial select agents were digested with trypsin and identified by Applied Biosystem 4800 plus MALDI TOF/TOF Analyzer (AB Sciex, USA). As described earlier, the trypsin digested peptides were spotted onto the 384-well MALDI target plate after mixing with equal volume of the CHCA matrix solution (10 mg/mL) and MS mass spectra were recoded in the reflector positive mode using a laser (200 Hz repetition rate with a wavelength of 355 nm) with accelerated voltage of 2 kV^34^. A six component peptide standard (mass range of 905–3660 Da) was used for the default calibration at 13 callibration points. For the identification of abundant proteins, the MS/MS mass spectra were acquired by the data dependent acquisition method. MS/MS fragment ions were generated by collision induced dissociation (CID) at 1 kV voltage for 20 strongest precursors selected with a signal-to-noise ratio >20, between the m/Z range of 850–4000 Da. MS/MS spectra were acquired as described previously^64^. CID was carried out for the fragmentation of precursor ions, sequentially selected by timed ion selector, using air as collision gas at 1 kV energy and 1.5 × 10^−6^ recharge pressure threshold. All MS and MS/MS spectra were obtained by accumulation of at least 1200 and 1600 laser shots, respectively and peak lists were generated using the 4000 Series Explorer Software v. 3.5 (Applied Biosystems). MS/MS peaks were selected on the basis of a signal-to-noise ratio greater than 10 over a mass range of 60–20 Da below the precursor mass. The data was analyzed using Protein Pilot version 4.0 (Applied Biosystem) employing the MASCOT 2.0 search engine (Matrix Science, London, UK). The peak list was searched against all entries at non-redundant protein sequence database of NCBI with 16338050 sequences. The following search parameters were used: 50 ppm precursor ion mass tolerance; ±0.6 Da fragment ion mass tolerance with +1 charge state, one missed cleavage for trypsin digestion, and variable modifications (oxidation of methionine and carbamidomethylation of cysteine). For the successful identification of protein by MS/MS, MASCOT score greater than 62 was accepted as significant (*p*-value < 0.05).

For targeted search approach, precursor ions on a ±0.5 Da window, with a signal-to-noise ratio greater than 20, were selected for MS/MS analysis from the inclusion list defined in the interpretation method of the instrument containing *m/z* for all unique specific peptides for the selected pathogenic bacterial species^34^.

### Bioinformatic analysis

Literature mining for the evidence of expression of proteins for a given pathogen was carried out using PUBMED search engine at NCBI (www.ncbi.nlm.nih.gov) using appropriate keywords. All the proteins experimentally shown to be expressed by the selected species (9) were initially ranked in the decreasing order of the number of reports for a given protein with a cut-off of at least three reports for each. Literature mining was also used for the attribution of functional role of expressed proteins with respect to immunogenicity, immuno-dominance in naturally infected clinical sera, immuno-protective nature, role in virulence, and surface localization. ExPASy Proteomics tools (http://www.expasy.ch) were also used for the prediction of molecular function/biological process and protein localization sites in cells (PSORT).

Candidate protein markers for each of the selected species were prioritized according to an arbitrarily designed scoring scheme that was aimed at selecting proteins/peptides unique to the species and expressed by the microorganism in abundance, in turn increasing the probability of getting better signal on MS analysis. We have taken three parameters for the prioritization of the proteins: the number of reports for the expression of a given protein (25 points), number of peptides with significant MS/MS ion score in the experimental identification of abundant proteins in the laboratory (25 points), and percent identity with the nearest homolog (50 points). FASTA sequences for each of the short-listed proteins were retrieved from the reference strain of the selected bacterial species and subjected to global protein BLAST against the non-redundant protein database at NCBI. Low percent identity with the nearest homolog in any other species was given maximum weightage for a better probability of getting unique marker peptides for targeted MS based assay development. For example, on the two extremes, 100% sequence identity fetched 0 point (100–100) while ≤50% identity gave complete 50 points for the identity parameter. Experimental identification of abundant proteins in the laboratory were assigned 25 points; significant MS/MS ion score for 1 peptide given 5 points and 5 additional points for every additional peptide up to 5 (≥5 peptides given complete 25 points). Number of independent reports was assigned 25 points with 5 points for 3 reports and 5 additional points for every additional report up to 7 (≥7 reports for the given protein assigned complete 25 points). This way, 15–27 proteins were short-listed as putative marker proteins for each of the selected species.

The FASTA sequences of the selected putative marker proteins were subjected to *in silico* tryptic digestion using the Peptide Mass algorithm at ExPASy Proteomics tools (http://www.expasy.org). All the peptides in the mass range of 1000–3000 Da obtained from the in silico digestion of putative marker proteins were subjected to global protein BLAST against the GeneBank non-redundant protein database (http://wwww.ncbi.nlm.nih.gov) with search parameters adjusted for short input sequence. Peptides showing less than 100% sequence identity with any other bacterial species (except the query species) were selected for the inclusion list of unique peptides. This selection was based on the assumption that a difference of even one single amino acid is most likely to ensure that the selected mass will not come from any other species except the target taxon. Peptide BLAST was given a tolerance for very closely species within the *Bacillus cereus* sensu lato group, including six other species (*B. cereus*, *B. mycoides*, *B. pseudomycoides*, *B. thuringiensis*, *B. weihenstephanensis*, and *B. cytotoxicus*). The tolerance within the *Bacillus cereus* sensu lato group meant that the peptide queried did not show 100% sequence identity with any other species but a complete match with one or more of the six species within the group was allowed. Similarly, the closely related species of *Burkholderia* (*B. mallei* and *B. pseudomallei*) and *Brucella* (*B. melitensis, B. abortus*, and *B. suis*) were considered together for the peptide BLAST search and scoring unique peptides. In order to discriminate between these closely related pathogens, the list of putative markers was further appended with peptides unique to these related species using reported literature. The unique peptides in the inclusion list were further screened for strain coverage using all the available sequences retrieved by taxon specific BLAST search and counting for the presence of a given peptide from the alignment data of these sequences from diverse strains in the genome data base. The details of *in silico* analysis and a summary of results are presented in the Supplementary Material S6.

### Proof-of-concept studies

Number coded, eight blind samples of soil or sand were spiked with none, one or two bacterial select agents and subjected to targeted and shotgun analysis using tandem mass spectrometry after extraction of proteins. *B. anthracis* Sterne strain and *C. perfringens* ATCC13124 were used for the spiking experiment. Exponentially growing cultures were harvested and washed with phosphate buffer saline and resuspended in 50 mM Tris.HCl (pH 7.2). Cells were added to garden soil and sand (sieved into particle size range of 1-2 mm) at a concentration of 10^6^ or 10^7^ cfu.g^−1^. After addition of the cell suspension (1.2 mL.g^−1^ soil or sand), the matrix was dried overnight at 37 °C to simulate the possible binding of the cells to matrices in coarse of drying under field conditions. Total protein was extracted by the method modified from Chourey *et al*.^65^. Briefly, 2 g of spiked sand or soil was resuspended in 1 ml distilled water and 1.5 ml Alkaline-SDS Buffer (5% SDS; 50 mM Tris.HCl; 0.15 M NaCl; 0.1 mM EDTA; 1 mM MgCl_2_; 50 mM DTT). After intermittent vortexing for 10 min at room temperature, the mixture was heated at 95^o^C for 10 min and allowed to cool at room temperature. Supernatant was collected by centrifugation at 10,000 × g for 30 min at room temperature and protein was precipitated by trichloro-acetic acid (TCA) at a final concentration of 10% (w/v) in the presence of β-mercaptoethanol (0.07%). The protein pellet collected by centrifugation (10,000 × g, 4 °C, 10 min) after 2 h of incubation on ice and washed twice with 500 μL of acetone. The pellet was air-dried for 5 min and the protein pellet was solubilized in buffer containing 8 M Urea and 2% CHAPS followed by a heating at 95 °C for 5 min in SDS lysis buffer before subjecting it to discontinuous denaturing SDS-PAGE. After staining with Coomassie Brilliant Blue G-250, abundant proteins from Coomassie-stained SDS-PAGE were excised and subjected to in gel digestion and identification by tandem mass spectrometry using shot-gun and targeted approach as described in the text.

## Supplementary information

Supplementary Information.
