# Supplementary material for: Elucidation of protein biomarkers for verification of selected biological warfare agents using tandem mass spectrometry
Source: Sci Rep. 2020 Feb 10;10:2205. doi: 10.1038/s41598-020-59156-3 (PMC7010682; doi:10.1038/s41598-020-59156-3)

Title: **Elucidation of protein biomarkers for verification of selected biological warfare agents using tandem mass spectrometry**

Authors: **Sakshi Rajoria, Sasikumar Sabna, Prabhakar Babele, Ravi Bhushan Kumar, Dev Vrat Kamboj, Subodh Kumar, Syed Imteyaz Alam**

Contents of supplementary material

Supplementary Table S1 (A to F): Protein identified by tandem MS analysis in the cell lysates of selected species.

Supplementary Table S2 (A to F): Summary of putative marker proteins for the specific detection of selected species.

Supplementary Table S3: Summary of selected marker proteins for specific detection of target bacterial agents of BW significance.

Supplementary Table S4 A to I: Inclusion list of unique peptides from selected protein markers of selected species.

Supplementary Table S5: Results from proof-of-concept studies.

Supplementary material S6: Methods for *in silico* analysis

Supplementary Figure S1: MS and MS/MS spectra of tryptic peptides obtained from three representative marker proteins.

Supplementary Figure S2: Gel image for the proof-of-concept study using blind samples

Supplementary Figure S3: MS and MS/MS spectra of tryptic peptides obtained from representative proteins identified in proof-of-concept study.

Supplementary Table S1 (A to F): Proteins identified by tandem MS analysis in the cell lysates of selected species.

Supplementary Table S1-A: Proteins identified by tandem MS analysis in the cell lysate of *Bacillus anthracis.*

S.No	Protein[accession no]	Observed m/z (Da)	Error (Da)	Peptide	Ion score	Unique[b]
1	50S ribosomal protein L31 [gi|30265354]	1594.68	0.066	VEISSDSHPFYTGR	62	N
2	Ribosomal protein L17 [gi|2760186]	1272.60	0.073	DLATDLIINER	76	N
3	30S ribosomal protein S4 [gi|30264729]	2063.96	0.096	AELPAEINEALIVEFYSR	113	N
4	30S ribosomal protein S10 [gi|30260300]	1884.93	0.076	LIDIVSPTPQTVDSLMR	120	N
		1411.75	0.053	LDLPSGVDIEIKL	59	N
5	Ribosomal protein L11 [gi|30260288]	2086.99	0.111	TADQAGLIIPVEITVFEDR	121	N
6	30S ribosomal protein S13 [gi|30018404]	1288.61	0.058	ILAEAGISEETR	46	N
		1423.76	0.068	VVISLTYVFGIGR	42	N
7	30S ribosomal protein S9 [gi|30260334]	1142.51	0.044	AQVQYYGTGR	32	N
		1455.66	0.058	DFENYIPFAALR	58	N
8	30S ribosomal protein S8 [gi|30018394]	1691.78	0.070	DVEYIEDNKQGILR	76	N
9	50S ribosomal protein S7 [gi|30260320]	1321.63	0.053	LGFEGGQTPLFR	41	N
		1376.70	0.051	EFAIVNLSTLNR	21	N
10	30S ribosomal protein S7 [gi|30260297]	1606.75	0.060	TGKEPMEVFEQALK	42	N
		1755.81	0.073	SQTILYNAFDIVSER	54	**Y***
		1769.84	0.079	VGGANYQVPVEVRPER	28	N
11	50S ribosomal protein L13 [gi|65317588]	1493.68	0.053	WYVVDAEGQTLGR	72	N
		1621.75	0.074	KWYVVDAEGQTLGR	52	N
12	50S ribosomal protein L16 [gi|254686616]	1550.74	0.046	IMFEIAGVSEEVAR	66	N
		1745.91	0.077	IFPSKPYTAKPLEVR	60	N
		2424.10	0.085	GGTEIAFGEFGLQAQAASWITNR	119	N
13	SSU ribosomal protein S30P [gi|30023223]	1159.54	0.025	YFDTFPEIK	47	N
		1515.84	0.036	VEVTIPFTDLLLR	82	N
		2382.07	0.070	AEETNSDMYAAIDLVVDKIER	80	**Y***
14	Ribosomal subunit interface protein [gi|30265214]	1159.54	0.025	YFDTFPEIK	47	N
		1515.84	0.036	VEVTIPFTDLLLR	82	N
		2382.07	0.070	AEETNSDMYAAIDLVVDKLER	80	N
15	50S ribosomal protein L5 [gi|30260313]	2312.18	0.064	TLDNAVEELTQITGQKPVVTR	121	**Y***

16	50S ribosomal protein L6 [gi|30018395]	1062.57	0.019	GLELVGVGYR	43	N
		1663.80	0.040	ALIGNMVEGVTEGFAR	49	N
17	Aconitate hydratase [gi|30263563]	1021.50	0.028	AVIAESFER	34	N
		1123.60	0.033	HGGILQMVLR	52	N
		1286.61	0.042	EPGEVETLSGLR	76	N
		1435.59	0.041	FDSEVEIDYYR	60	N
		1515.78	0.002	DVQDIDVPFKPSR	68	N
		1869.85	0.059	AGTADALAFNMDLEFKR	36	N
		2013.06	0.075	VILQDFTGVPAVVDLASLR	32	N
18	Phosphopyruvatehydratase [gi|30265161]	1822.86	0.059	AIVPSGASTGEHEAVELR	71	N
		1911.87	0.038	GNPTVEVEVYTESGAFGR	48	N
		1945.88	0.054	GLNTAVGDEGGFAPNLGSNR	96	N
19	(3R)-hydroxymyristoyl-ACP dehydratase [gi|30265290]	1283.57	0.046	LAFFAGIDNCR	46	N
20	S-layer protein EA1 [gi|30261021]	2302.03	0.079	SFPDVPAGHWAEGSINYLVDK	130	**Y***
		1140.54	0.032	ADLYDTLTTK	58	**Y***
		1684.83	0.059	ILNLPVDENAQPSFK	78	N
		1765.85	0.057	SVNFKPVQTENFVEK	81	N
		1934.88	0.077	GAITGKPDGTYGPTESIDR	143	**Y***
		2527.21	0.053	TLPVTFVTTDQYGDPFGANTAAIK	143	**Y***
		1179.53	0.033	VGQYGQSPDTK	75	**Y***
		1223.55	0.032	DNAQAYVTDVK	44	**Y***
		1340.56	0.042	GDGKENFYPEGK	60	**Y***
		1405.72	0.047	AEAAQFIALTDKK	73	N
		1340.57	0.040	GDGKENFYPEGK	53	**Y***
		1405.72	0.047	AEAAQFIALTDKK	61	N
		1414.76	0.045	ATVEIVQETIAIK	58	**Y***
		1501.72	0.047	ASFASMLVSAYNLK	109	N
		1533.74	0.045	DFALNSQNLVVGEK	100	N
		1684.83	0.052	ILNLPVDENAQPSFK	86	N
		1857.78	0.057	VYSDPENLEGYEVESK	79	N
		1878.89	0.051	LGDVTVSQTSDSALPNFK	113	N
		1996.90	0.053	NVVFALDADNDGVVNYGSK	125	N

		2497.12	0.063	FVANNLDGSPANIFEGGEATSTTGK	142	**Y***
		2773.35	0.081	SSNHGIISVVNNYITAEAAGEATLTIK	143	N
		1772.83	0.010	GNADVEYLNLANHDVK	54	N
		1416.67	0.029	GHWAYEAVNTLR	71	N
		1889.83	0.031	GSYSLYFSFSDGYIQR	77	N
21	DNA-dependent RNA polymerase [gi|126654238]	1471.69	0.066	VNKFGFIETPYR	45	N
22	Elongation factor G [gi|30260298]	885.46	0.019	ILYYTGR	39	N
		1093.50	0.013	LSEEDPTFR	35	N
		1373.68	0.032	VEANVGAPQVAYR	27	N
		1411.68	0.009	SSDEEPFAALAFK	30	N
		1663.80	0.038	IGADFLYSVGTIHDR	54	N
		1870.87	0.038	VEIPEEHKELAEEYR	73	N
		2039.98	0.053	VNIIDTPGHVDFTVEVER	44	N
		2239.02	0.039	VEVVIPEEYMGDIMGDVTSR	107	N
		2390.95	0.041	IGETHEGASQMDWMEQEQER	102	N
		2540.23	0.065	VLDGAVAVLDAQSGVEPQTETVWR	105	N
23	Elongation factor Tu [gi|254739504]	1667.85	0.047	LLDQAQAGDNIGALLR	140	N
		1217.55	0.028	ALQGEADWEAK	55	N
		1795.93	0.022	SKPHVNIGTIGHVDHGK	65	N
		1806.83	0.047	GITISTAHVEYETETR	73	N
		1959.95	0.058	IIELMAEVDAYIPTPER	75	N
		2181.02	0.069	ETDKPFLMPVEDVFSITGR	101	N
		2736.28	0.096	VGDVVEIIGLAEENASTTVTGVEMFR	77	N
		1475.82	0.038	QVGVPYIVVFLNK	57	N
		2223.90	0.074	CDMVDDEELLELVEMEVR	91	N
		2701.20	0.102	NMITGAAQMDGGILVVSAADGPMPQT R	109	N
		2736.26	0.106	VGDVVEIIGLAEENASTTVTGVEMFR	79	N
		2237.93	0.059	CDMVEDEELLELVEMEVR	76	N
24	Isocitrate dehydrogenase [gi|30022668]	970.47	0.028	VVTYDFAR	37	N
		1186.52	0.024	VFTWAEYDR	33	N
		2131.01	0.066	AFNQTGEWLPEETLNLIR	55	N

25	Isocitrate Dehydrogenase [gi|15826081]	970.47	0.028	VVTYDFAR	37	N
		1186.52	0.029	VFTWAQYDR	32	N
26	Alcohol dehydrogenase [gi|30262278]	1441.64	0.027	VSIAWMFQSCGR	39	N
		1626.90	0.037	LVLDGIEVVGSLVGTR	76	N
		1822.96	0.044	VVAVGLPVETMDLNIPR	77	N
27	Glyceraldehyde-3-phosphate dehydrogenase [gi|30265165]	1703.72	0.036	VLSWYDNETGYSNR	123	N
		2304.06	0.050	DPAQLPWSDYGVEVVVESTGR	48	N
		2872.34	0.105	VIAERDPAQLPWSDYGVEVVVESTGR	19	N
28	NADH dehydrogenase [gi|30265112]	1865.89	0.043	AFEEFAPVNSGTLASLGR	56	N
29	3-hydroxybutyryl-CoA dehydrogenase [gi|30265367]	1035.51	0.020	YVNAGWLGR	27	N
		1675.81	0.032	VPVEVNDFPGFVSNR	68	N
30	Electron transfer flavoprotein [gi|30264589]	2338.13	0.066	DKDADLILGGNVAIDGASGQVGPR	138	N
		2895.37	0.087	DVEGDTEVVETSLPVLVTAQQGLNEPR	69	N
31	Histidinol-phosphate aminotransferase [gi|30261613]	1902.98	0.045	IGYAVGNTELIGQLEVAR	67	N
32	Cysteine synthase [gi|30260259]	1114.49	0.022	TEQAFEYAR	54	N
		1197.66	0.021	VLVIIPSNGER	36	N
		1393.66	0.027	EHGYFIPQQFK	66	N
		1958.95	0.045	AYGAELVLTPGPEGMGGAIR	54	N
33	Enoyl-(acyl carrier protein) reductase [gi|30019369]	1221.61	0.024	TFVVMGVANQR	36	N
		2200.01	0.058	TTTQEEVGDTAVFLFSDLAR	82	N
34	Flavinreductase [gi|52140242]	1300.69	0.027	LGEQFELVRPI	42	N
35	Reductase protein [gi|4584102]	2034.96	0.045	SIDTAAIYQNEEGVGQAIR	63	N
36	2,5-didehydrogluconate reductase [gi|229015594]	2034.96	0.082	SIDTAAIYKNEEGVGQAIR	63	N
37	Alkyl hydroperoxidereductase [gi|30260515]	1307.63	0.027	IEYIMIGDPTR	47	N
		1375.77	0.027	MLLIGTEVKPFK	79	N
		1942.01	0.052	WQEGSATLKPSLDLVGKI	136	N
		2008.94	0.058	TITTNFNVLMEEEGLAAR	154	N
		2024.95	0.039	TITTNFNVLMEEEGLAAR	40	N
		2036.91	0.044	ANAYHNGEFIQVTDESLK	36	N
		2048.94	0.048	ELGVEVYSVSTDTHFTHK	111	N
		2418.12	0.072	GTFIIDPDGVIQSMEINADGIGR	146	N
38	Hypothetical protein BA_5565 [gi|30265345]	1299.69	0.027	LGEQFELVRPL	42	N

39	Hypothetical protein BC4088 [gi|30022175]	1944.92	0.030	TFETDGVYHIIAHTNAR	59	N
40	Hypothetical protein bcere0006_38520 [gi|229174765]	1944.92	0.030	TFQTDGVYHIIAHTNAR	59	**Y***
41	Hypothetical protein bthur0009_10850 [gi|254726266]	1554.79	0.039	VGSFAPVNNVIYFK	71	N
		1604.70	0.032	MKDFYYEQPIDR	66	N
		2391.11	0.072	FHLLYQLENGTWTVHETFR	35	N
		2413.04	0.066	TPELTFLNEEMHSGFFTQER	43	**Y***
		2695.25	0.089	EYAFVPHLTIGQGLSDAEHADVLGR	149	N
42	Hypothetical protein BA_4148 [gi|30264009]	1634.70	0.068	NHFVLAGQWDYER	59	N
43	Hypothetical protein BC1692 [gi|30261802]	1483.67	0.035	ELLQEFQEYER	42	N
44	Hypothetical protein bthur0005_11990 [gi|75765154]	1317.66	0.030	QFIEQQQLQR	30	N
45	Single-stranded DNA-binding protein [gi|47531059]	1422.65	0.031	EADFINCVIWR	40	N
		1794.89	0.041	YTPNGVAVATFTLAVNR	31	N
		1978.99	0.049	VYVTEVLAESVQFLEPR	27	N
46	PTS system, glucose-specific IIA component [gi|30265343]	1442.70	0.025	NIEEVPDPVFAGR	58	N
		1542.80	0.027	TNEETIVAPLTGAVK	38	N
47	Universal stress protein [gi|30022700]	1246.63	0.035	IETVLEFGNPK	85	N
		1370.73	0.040	FLIGSVSEHIIR	81	N
		1668.81	0.030	ANLFAEDLLEDYKK	51	N
		1116.49	0.041	AYSAVEAYSR	47	N
		1718.78	0.070	VDLIMCGATGLNAVER	85	N
48	Chain A, Dlp-1 [gi|21730368]	2152.92	0.081	FEELYTESATHIDEIAER	68	N
49	Cytoplasmic protein [gi|30022265]	1699.80	0.091	EVNVHIVGVTFETQK	81	N
50	Regulatory protein SpoVG [gi|30018319]	1180.54	0.063	DIAHPINSGTR	43	N
		1344.63	0.056	IQDAVLTEYHR	76	N
		1935.94	0.083	AIASITLDHEFVVHDIR	55	N
51	Anti-sigma F factor antagonist [gi|30264152]	1590.72	0.064	LAGELDHHTAEELR	86	N
52	Major cold-shock protein [gi|254752895]	1964.83	0.080	TLEEGQEVTFEVEQGNR	121	N
53	10 kDa chaperonin GROES [gi|30260442]	1242.58	0.052	YEGTDYLILR	66	N
		1256.65	0.054	VVIELVQAEEK	57	N
		1545.82	0.068	VALEVAAGDLIIFSK	103	N
		1990.91	0.097	YAGTEVKYEGTDYLILR	76	N
54	Formate-acetyltransferase [gi|30260668]	984.43	0.040	QMQFFGAR	20	N

		1305.62	0.054	SGVITGLPDAYGR	42	N
		1538.65	0.061	TGEPFAPGANPMHGR	27	N
		1555.64	0.060	TSAIQYENDDIMR	45	N
		1837.80	0.075	KADLNLTGGVMSEDTMR	36	N
55	Unnamed protein product [gi|580888]	1254.63	0.053	FQLTDIPPAPR	66	N
		1565.73	0.065	AVITVPAYFNDAER	35	N
56	Molecular chaperone DnaK [gi|30022393]	1254.63	0.053	FQLTDLPPAPR	66	N
		1565.73	0.065	AVITVPAYFNDAER	35	N
57	Formate-acetyltransferase [gi|251799218]	984.43	0.039	QMQFFGAR	24	N
		1357.64	0.050	TSTFLDIYIER	66	N
		1765.76	0.032	THNQGVFDAYTPEMR	24	N
58	Molecular chaperone GroEL [gi|30260443]	1200.59	0.019	NVTAGANPMGLR	44	N
		1595.67	0.050	EIELEDAFENMGAK	69	N
		1654.84	0.063	QIAINAGLEGSVVVER	50	N
		1682.72	0.062	AQLEETTSEFDREK	51	N
		1843.82	0.026	GFTTELDVVEGMQFDR	134	N
		1927.02	0.028	VASIVAEGDEATGINIVLR	103	N
		2106.02	0.020	TNDVAGDGTTTATVLAQAMIR	106	N
		2230.06	0.096	AMLEDIAILTGGEVITEELGR	102	N
		2659.28	0.043	SSIAQVAAISAADEEVGQLIAEAMER	189	N
		1425.62	0.022	AQLEETTSEFDR	54	N
		2230.13	0.033	AMLEDIAILTGGEVITEELGR	138	N
59	Chaperonin [gi|319654650]	1200.59	0.019	NVTAGANPMGIR	44	N
		1595.72	0.005	EIELEDAFENMGAK	56	N
		1843.82	0.026	GFTTELDVVEGMQFDR	134	N
		2106.02	0.020	TNDVAGDGTTTATVLAQAMIR	106	N
		2230.13	0.033	AMLEDIAILTGGEVITEELGR	138	N
60	Cpn60 [gi|325169661]	1200.59	0.019	VTAGANPMGIR	44	N
		1842.81	0.026	GFTTELDVVEGMQFDR	134	N

[b]N = Not a unique peptide; Y= peptide unique to the genus in global BLAST; **Y** (bold) = peptide unique to the species in global blast; Y* = peptide unique to the species with one exception.

Supplementary Table S1-B: Protein identified by tandem MS analysis in the cell lysate of *Brucella abortus*.

S.No	Protein [accession no]	Observed m/z (Da)	Error (Da)	Pepide	Ion score	Unique
1	Aldehyde dehydrogenase [gi|17988030]	1698.86	0.017	ETTNADLPLAVDHFR	82	N
		2084.02	0.052	YFENTSPVNGQVLCEVAR	85	N
		1653.82	0.044	SDAADVEAALDAAHAAR	57	N
2	Glucose-6-phosphate 1-dehydrogenase [gi|62317385]	1842.00	0.028	AFYLAVSPTLFGDIATR	35	N
3	Glyceraldehyde-3-phosphate dehydrogenase	2388.26	0.021	TDIQVVAINDLGPVETNAHLLR	67	N
	[gi|4165122]	1759.81	0.009	ILSWYDNEWGFSSR	63	N
		2388.28	0.007	TDIQVVAINDLGPVETNAHLLR	65	N
4	Alcohol dehydrogenase [gi|62290600]	1759.82	0.017	ILSWYDNEWGFSSR	58	N
		2388.27	0.014	TDIQVVAINDLGPVETNAHLLR	79	N
5	Malate dehydrogenase [gi|62290781]	2406.21	0.018	FTGANDYAAIEGADVVIVTAGVPR	59	N
6	3-hydroxyisobutyrate dehydrogenase [gi|62290218]	2124.95	0.008	FDDEGHGGEDFSGIINFLR	113	Y
7	Chaperonin GroEL [gi|144111]	1583.87	0.004	AAVEEGIVAGGGTALLR	67	N
		1853.88	0.026	TAETELEVVEGMQFDR	63	N
		1455.73	0.023	GVNADQEAGINIVR	57	N
		1233.62	0.026	LESVTLDMLGR	28	N
		2039.01	0.059	EVELEDKFENMGAQMLR	44	N
8	Molecular chaperone DnaK [gi|62290965]	1545.78	0.009	AGEIDEVVLVGGMTR	55	N
		1592.90	0.007	LLGQFDLVGIPPAPR	37	N
		1522.71	0.025	TTPSIIAFTDGDER	32	N
		1744.77	0.007	STNGDTFLGGEDFDIR	55	N
9	Elongation factor Tu [gi|237815669]	1377.63	0.008	AYDQIDAAPEER	78	N
		1653.89	0.005	LLDQGQAGDNIGALIR	108	N
		1781.98	0.001	KLLDQGQAGDNIGALIR	22	N
		2145.03	0.002	CDQVDDAELLELVELEVR	73	N
		1761.86	0.012	GITISTAHVEYETANR	27	N
		2020.97	0.005	HTPFFTNYRPQFYFR	15	N
10	Immunogenic 39-kDa protein [gi|1911181]	1173.52	0.005	TYVDDNFSGR	39	N
		1860.96	0.002	VTAGNAPTAVQMLGFDIR	55	N
		2206.07	0.016	GISWTDMPVAGGGGTEAMTVLR	40	N

		1709.98	0.005	LLATLYPETIHIVAR	40	Y*
		1820.94	0.003	LDSATSSLGIPLHPGAER	57	Y*
11	Aminotransferase [gi|62317484]	1853.99	0.001	ALGLEPTLSANDIEALAR	90	N
		1977.00	0.001	GGEVFTPVPNGTFLNGITR	39	N
		2033.08	0.001	ILGAASQATWLGSLVFDGAR	76	N
		1144.63	0.000	VVPIIGFDER	27	N
12	L-amino acid-binding periplasmic protein AAPJ	1581.83	0.007	FTAFQSGEVDVLIR	73	Y*
	[gi|260757721]					
13	Universal stress protein [gi|17987221]	1026.51	0.014	QGINYEFR	68	Y
		1492.81	0.004	HGVGVEVLALASQGR	37	**Y***
14	30S ribosomal protein S2 [gi|17982767]	1071.53	0.010	SAQYYVNAR	36	N
		1278.69	0.008	AIALYCDLIAR	47	N
15	30S ribosomal protein S8 [gi|62290130]	1095.53	0.052	YYEGVPVIR	33	N
		1274.55	0.056	EQNVGGELLCR	36	N
		1375.69	0.063	VLDVLQAEGYIR	53	N
16	50S ribosomal protein L9 [gi|62289437]	1671.86	0.009	DIAEIITANGFTLHR	77	Y*
17	ATP-dependent clp protease [gi|62290024]	871.43	0.006	AYDIFSR	38	N
		911.41	0.009	DYDTIER	39	N
		2056.99	0.009	IMVHQPSGGFQGQASDIER	62	N
18	Putative translaldolase [gi|260755493]	1206.55	0.012	TIYDNYDFR	53	N
		1760.89	0.021	LDDTGINGMELIAEIR	69	N
19	Putative translaldolase [gi|260755493]	1206.52	0.013	TIYDNYDFR	46	N
		1759.86	0.028	LDDTGINGMELIAEIR	32	N
20	Ribosome recycling factor [gi|17987109]	1659.85	0.017	DSGLGLNPITDGMTLR	75	N
21	Fe/Mn family superoxide dismutase [gi|62289534]	1490.67	0.043	AFDSDLGGYDKFR	58	N
		2139.03	0.033	ADFIAAGAGQFGSGWAWLSVK	116	N
		2287.06	0.043	AFELPALPYDYDALAPFMSR	90	N
22	Bacterioferritin [gi|62317577]	1137.56	0.003	LLNDWGYTR	32	Y
		1944.06	0.035	IIFLEGFPNLQTVSPLR	88	Y*
		1607.77	0.016	EVLEADLKGEYDAR	98	N
23	Inorganic pyrophosphatase [gi|17986360]	1296.53	0.015	IGDWGDEDYAR	63	N
24	Biotin carboxyl carrier protein [gi|13487161]	2496.17	0.036	DLADILNETDLTDIEVEHGDLR	124	N

25	DNA starvation/stationary phase protection protein	1006.49	0.038	YGDVANLVR	68	N
	Dps [gi|62317577]	1483.68	0.017	AELDDHVDTIAER	72	Y*
		1716.79	0.056	GPQFIAVHEMLDGFR	83	N
		2399.01	0.030	ESIKDADDAGDDDTADIFTAASR	153	**Y***
		2463.25	0.085	LKPYPTDIYAVHDHLVALIER	114	Y
		1543.70	0.052	ALWFLEAHVQESN	80	Y
26	Invasion protein B [gi|17987867]	1189.51	0.044	QEQSSAQAGQR	63	**Y***
27	Peptidoglycan-associated lipoprotein [gi|260755394]	1063.51	0.038	EYNLALGQR	38	N
		1325.66	0.043	IFFDLDSSLIR	85	N
		1778.76	0.067	YPQYSITIEGHADER	39	N
28	Small heat shock protein HspA [gi|17988067]	1367.56	0.061	SGDGYPPYNIER	20	N
29	Putative cytoplasmic protein [gi|62317375]	1078.54	0.018	LPVFQGFDR	31	Y
30	Hypothetical protein BMEI1214 [gi|62289693]	1465.82	0.057	GALIGAGAGALGGLLVR	38	N
		1698.84	0.069	DVSIGTAGGAIIGGIAGGGR	84	Y*
		1706.76	0.052	NYAGFDLASDDIHIR	59	Y

[b]N = Not a unique peptide; Y= peptide unique to the genus in global BLAST; **Y** (bold) = peptide unique to the species in global blast; Y* = peptide unique to the species with one exception.

Supplementary Table S1-D: Protein identified by tandem MS analysis in the cell lysate of *Clostridium botulinum*.

S.No	Protein [accession no]	Observed m/z (Da)	Peptide	MS or MS/MS	Unique	Reference
1	**BoNT/A**	1272.66	SFGHEVLNLTR	MS	**Y**	1
		1936.90	GANLSTNFNGQNTEINSR	-do-	**Y**	1
		1130.60	YTMFHYLR*	-do-	**Y**	1
		1148.60	YVDVNNVGIR	-do-	**Y**	1
		1122.50	LVASNWYNR**	-do-	**Y**	1
		1181.60	IPNAGQMQPVK*	MS/MS	**Y**	2
		911.60	IWVIPER*	-do-	**Y**	2
		923.50	IYSTDLGR*	-do-	N	2
		1619.80	GIPFWGGSTIDTELK**	-do-	**Y**	2
		1271.70	SFGHEVLNLTR	-do-	**Y**	2
		2055.10	FATDPAVTLAHELIHAGHR	-do-	**Y**	2
		1129.60	LYGIAINPNR**	-do-	**Y**	2
		1396.70	FIDSLQENEFR*	-do-	**Y**	2
		1473.80	VNYTIYDGFNLR	-do-	**Y**	2
		1264.60	NFTGLFEFYK**	-do-	**Y**	2
		1070.60	VNTQIDLIR**	-do-	**Y**	2
		1202.60	EALENQAEATK	-do-	**Y**	2
		1492.70	NNINFNIDDLSSK*	-do-	**Y**	2
		1192.60	RLEDFDASLK	-do-	**Y**	2
		957.50	GTLIGQVDR	-do-	**Y**	2
		1675.90	VNNTLSTDIPFQLSK	-do-	**Y**	2
		946.50	VNFDPIDK	-do-	**Y**	2
		1465.70	DLYDNQSNSGILK	-do-	**Y**	2
		1147.60	YVDVNNVGIR	-do-	**Y**	2
2	**BoNT/B**	776.37	VLQNFR**	MS	N	1
		1097.50	EGFGGIMQMK**	-do-	**Y**	1
		1262.60	LGCNWQFIPK*	-do-	**Y**	1
		2077.00	YFSIFNTELSQSNIEER	-do-	**Y**	1
		1112.60	LISNPGEVER*	MS/MS	**Y**	2
		1237.70	VDDLPIVPNEK	-do-	**Y**	2
		1201.60	YSIDVESFDK*	-do-	**Y**	2
		1309.70	ASYFSDSLPPVK**	-do-	**Y**	2
		2226.10	NLLDNEIYTIEEGFNISDK**	-do-	**Y**	2
		858.50	EHLAVYK**	-do-	N	2
		2723.40	IFTDENTIFQYLYSQTFPLDIR*	-do-	**Y**	2
		1388.70	QIVNDFVIEANK	-do-	**Y**	2
		874.50	TIDNALTK	-do-	**Y**	2

		1603.80	ALNYQAQALEEIIK**	-do-	**Y**	2
		1492.70	SNINIDFNDINSK	-do-	**Y**	2
		1077.60	LLDFDNTLK	-do-	**Y**	2
		1234.60	NLLNYIDENK**	-do-	**Y**	2
		1284.70	LYLIGSAEYEK*	-do-	**Y**	2
		1687.90	YNSEILNNIILNLR	-do-	**Y**	2
		1379.70	DNNLIDLSGYGAK	-do-	**Y**	2
		1378.70	VEVYDGVELNDK	-do-	**Y**	2
		1137.50	EDISEYINR	-do-	**Y**	2
		1129.60	IQSYSEYLK*	-do-	N	2
		1085.60	DSPVGEILTR	-do-	**Y**	2
		1346.60	SNSQSINDDIVR*	-do-	**Y**	2
		2292.10	KEDYIYLDFFNLNQEWR	-do-	**Y**	2
		2213.10	LFLAPISDSDEFYNTIQIK	-do-	**Y**	2
		1810.90	KDEESTDEIGLIGIHR*	-do-	**Y**	2
3	**BoNT/C**	1849.90	PITINNFNYSDPVDNK**	MS/MS	**Y**	2
		2097.00	NILYLDTHLNTLANEPEK**	-do-	**Y**	2
		12872.70	ITGNIWVIPDR	-do-	**Y**	2
		1249.70	NSNPNLNKPPR	-do-	**Y**	2
		2328.00	SGYYDPNYLSTDSDKDTFLK	-do-	**Y**	2
		1120.60	EIGEELIYR**	-do-	**Y**	2
		1414.80	TGSINPSVIITGPR**	-do-	**Y**	2
		1395.70	ENIIDPETSTFK**	-do-	**Y**	2
		1492.70	LNSITTANPSSFNK**	-do-	**Y**	2
		1421.60	FVVESSGEVTVNR**	-do-	**Y**	2
		2610.30	NKFVELYNELTQIFTEFNYAK	-do-	**Y**	2
		905.50	IYNVQNR**	-do-	N	2
		1591.80	SNLNVLFMGQNLSR**	-do-	**Y**	2
		1532.80	NTDLPFIGDISDVK	-do-	**Y**	2
		2909.50	KDINEETEVIYYPDNVSVDQVILSK	-do-	**Y**	2
		2781.30	DINEETEVIYYPDNVSVDQVILSK	-do-	**Y**	2
		3778.80	NTSEHGQLDLLYPSIDSESEILPGENQVFYDNR	-do-	**Y**	2
		2127.00	TQNVDYLNSYYYLESQK**	-do-	**Y**	2
		1442.70	LSDNVEDFTFTR	-do-	**Y**	2
		1175.60	SIEEALDNSAK	-do-	**Y**	2
		1315.70	VYTYFPTLANK	-do-	**Y**	2
		2978.50	VNAGVQGGLFLMWANDVVEDFTTNILR	-do-	**Y**	2
		2794.30	IITQFNNISYQMYDSLNYQAGAIK	-do-	**Y**	2
		1139.60	YSGSDKENIK**	-do-	**Y**	2
		1132.60	ISEAMNNINK**	-do-	**Y**	2
		1248.60	VIDELNEFDR	-do-	**Y**	2
		2004.10	LINLIDSHNIILVGEVDK	-do-	**Y**	2
		2674.30	VNNSFQNTIPFNIFSYTNNSLLK	-do-	**Y**	2

		1697.80	DIINEYFNNINDSK	-do-	**Y**	2
4	**BoNT/D**	2072.10	DFNYSDPVNDNDILYLR**	MS/MS	**Y**	2
		1717.10	AFMITQNIWVIPER**	-do-	**Y**	2
		2786.60	FNSLYSDLTNVMSEVVYSSQYNVK**	-do-	**Y**	2
		1323.80	LSSESVVDLFTK**	-do-	**Y**	2
		1280.80	IYTFLPSLAEK**	-do-	**Y**	2
		2695.60	GVQAGLFLNWANEVVEDFTTNIMK**	-do-	**Y**	2
		1161.60	TIENCLEQR**	-do-	**Y**	2
		2409.40	VAPEFLLTFSDVTSNQSSAVLGK**	-do-	**Y**	2
		2491.60	TKTELINLIDSHNIILVGEVDR**	-do-	**Y**	2
5	**BoNT/E**	1566.70	INSFNYNDPVNDR*	MS/MS	**Y**	2
		1039.60	NIWIIPER*	-do-	**Y**	2
		1850.90	NVIGTTPQDFHPPTSLK*	-do-	**Y**	2
		1712.90	INNNLSGGILLEELSK*	-do-	**Y**	2
		2799.30	ANPYLGNDNTPDNQFHIGDASAVEIK*	-do-	**Y**	2
		2834.30	NNYMPSNHGFGSIAIVTFSPEYSFR*	-do-	**Y**	2
		1279.60	DASGIYSVNINK*	-do-	**Y**	2
		1433.70	LYSFTEFDLATK*	-do-	**Y**	2
		2510.30	LSNLLNDSIYNISEGYNINNLK*	-do-	**Y**	2
		982.50	GQNANLNPR*	-do-	**Y**	2
		3898.80	EIDDTVTSNNNYENDLDQVILNFNSESAPGLSDEK**	-do-	**Y**	2
		1501.80	LNLTIQNDAYIPK**	-do-	**Y**	2
		2467.20	VPEGENNVNLTSSIDTALLEQPK*	-do-	**Y**	2
		2410.10	IADISIVVPYIGLALNIGNEAQK**	-do-	**Y**	2
		3179.80	DALELLGAGILLEFEPELLIPTILVFTIK**	-do-	**Y**	2
		1602.70	EVYSFIVSNWMTK**	-do-	**Y**	2
		1776.90	EQMYQALQNQVNAIK	-do-	**Y**	2
		1145.50	YNSYTLEEK**	-do-	**Y**	2
		114.60	QIENELNQK**	-do-	**Y**	2
		1131.60	VSIAMNNIDR*	-do-	**Y**	2
		1417.70	FLTESSISYLMK**	-do-	**Y**	2
		927.40	LSSYTDDK**	-do-	**Y**	2
		892.40	SSVLNMR**	-do-	N	2
		1154.60	NFSISFWVR**	-do-	N	2
		1851.90	IVNVNNEYTIINCMR**	-do-	**Y**	2
		2406.20	VSLNHNEIIWTLQDNAGINQK	-do-	**Y**	2
		1263.70	WIFVTITNDR**	-do-	**Y**	2
		1289.60	LYINGNLIDQK	-do-	**Y**	2
		1896.00	SILNLGNIHVSDNILFK**	-do-	**Y**	2
		3390.70	YFNIFDKELDETEIQTLYSNEPNTNILK	-do-	**Y**	2
		1432.70	DFWGNYLLYDK**	-do-	**Y**	2
		1131.60	DSTLSINNIR**	-do-	**Y**	2
		886.50	STILLANR**	-do-	N	2

		1524.80	KNDQVYINFVASK*	-do-	**Y**	2
		1691.80	THLFPLYADTATTNK	-do-	**Y**	2
6	**BoNT/F**	2673.40	SSIILNLLVLGAGPDIFENSSYPVR	MS/MS	**Y**	2
		2226.10	LEEFLTFGGQDLNIITSAMK	-do-	**Y**	2
		2505.10	VNSAPPEYDINEYK	-do-	**Y**	2
		1412.70	LYSFTEIDLANK	-do-	**Y**	2
		2861.40	VPNLLDDDIYTVSEGFNIGNLAVNNR	-do-	**Y**	2
		1039.50	DFTTEATQK	-do-	**Y**	2
		1046.50	AINNSLMER	-do-	**Y**	2
		1665.80	EIYSWIVSNWLTR	-do-	**Y**	2
		1977.00	LESEYNINNIREELNK	-do-	**Y**	2
		1160.60	VSLAMENIER	-do-	**Y**	2
		1477.70	FITESSIFYLMK	-do-	**Y**	2
		2257.00	EYDEGVKEYLLDYISEHR	-do-	**Y**	2
		3611.80	SILGNSVQELNDLVTSTLNNSIPFELSSYTNDK	-do-	**Y**	2
		1445.70	YQNFSISFWVR**	-do-	**Y**	2
		1735.80	VNLNNEYTIIDCIR	-do-	**Y**	2
		1600.80	IIWTLQDTAGNNQK	-do-	**Y**	2
		2048.00	LVFNYTQMISISDYINK	-do-	**Y**	2
		1262.70	WIFVTITNNR**	-do-	**Y**	2
		1948.90	TEIETLYSDEPDPSILK	-do-	**Y**	2
		1431.70	DFWGNYLLYNK	-do-	**Y**	2
		1066.60	YYLLNLLR	-do-	**N**	2
		1538.10	NGSTDISNTDNFVR	-do-	**Y**	2
		1418.70	KNDLAYINVVDR	-do-	**Y**	2
7	**BoNT/G**	2140.00	FTYGFQPDQFNASTGVFSK**	MS/MS	**Y**	2
		1567.70	DVYEYYDPTYLK**	-do-	**Y**	2
		1246.70	FAANVANVSINK**	-do-	**Y**	2
		1968.90	FCPSCLNVFNNVQENK**	-do-	**Y**	2
		1288.70	ALQNFQDIANR**	-do-	**Y**	2
		1864.00	LNIVSSAQGSGIDISLYK**	-do-	**Y**	2
		1961.00	ALMFGFTETNLAGEYGIK**	-do-	**Y**	2
		1505.70	YSYFSEYLPPIK**	-do-	**Y**	2
		2040.00	LLDNTIYTQNEGFNIASK**	-do-	**Y**	2
		2275.20	AVNKEAYEEISLEHLVIYR**	-do-	**Y**	2
		1425.70	GVIDDFTSESTWK**	-do-	**Y**	2
		284.70	IIWTLIDVNAK**	-do-	**Y**	2
		1540.80	FVWIKDFNIFGR**	-do-	**Y**	2
		2098.10	TNFNNAAINYQNLYLGLR**	-do-	**Y**	2

[b]N = Not a unique peptide; Y= peptide unique to the genus in global BLAST; **Y** (bold) = peptide unique to the species in global blast.

1. Kull, S.; Pauly, D.; Sto¨rmann, B.; Kirchner, S.; Sta¨mmler, M.; Dorner, M. B.; Lasch, P.; Naumann, D.; Dorner, B. G. Multiplex Detection of Microbial and Plant Toxins by Immunoaffinity Enrichment and Matrix-Assisted Laser Desorption/Ionization Mass Spectrometry. Anal. Chem. 2010, 82, 2916–2924.

2. Hines, H. B.; Lebeda, F.; Hale, M.; Brueggemann, E. E. Characterization of Botulinum Progenitor Toxins by Mass Spectrometry. Appl. Environ. Microbiol. 2005, 71(8), 4478–4486.

Supplementary Table S1-E: Protein identified by tandem MS analysis in the cell lysate of *Clostridium perfringens*.

S.No	Protein[accession no]	Observed m/z (Da)	Error (Da)	Peptide	Ion score	Unique
1	Pyruvate-flavodoxin oxidoreductase [gi|18311043]	1358.64	0.039	IPFVNFFDGFR	57	N
		1676.81	0.056	NAFYGLENAPVIVGGR	40	**Y**
		1843.66	0.062	EYNCFDYYGAADAER	33	**Y**
		1859.81	0.061	EALEAWIAEVDNGEGTR	30	**Y**
2	Acetaldehyde-CoA/alcohol dehydrogenase	1171.54	0.057	NAVFFSPHPR	55	**Y**
	[gi|18311513]	1397.60	0.066	FSQEQVDEIFR	54	N
		2169.97	0.115	VLIGEVESVELEEPFSHEK	93	N
		2366.94	0.147	IMWVMYEHPEVAFEDLAMR	41	N
3	Hypothetical protein CLOSPO_00599 [gi|187777054]	2169.97	0.115	ILVGEVQSVELEEPFSHEK	93	N
4	Hypothetical protein CLOSCI_00827 [gi|167758487]	1152.58	0.022	TVVTGIEMFR	45	N
		2137.04	0.022	ATDQPFLMPVEDVFTITGR	28	N
5	Hypothetical protein CATMIT_02801	1030.42	0.036	MFDGIEFR	40	N
		1279.54	0.043	NSVVFDEAENR	78	N
6	Hypothetical protein CPE0268 [gi|18309250]	1106.57	0.022	TQFGYHLIK	55	N
		1792.82	0.035	YPAQQQAMLASDEGKR	111	**Y**
		2047.99	0.049	AFEDVKEQVVNMLIQER	102	**Y**
		2293.14	0.050	VLATVGNTEITSDYIDEIIAR	102	**Y**
7	Hypothetical protein CLOL250_01067	1796.84	0.047	YAVGQFNINNLEWTK	62	N
	[gi|160893516]					
8	Hypothetical protein CLOBAR_01364	1796.83	0.058	YAVGQFNINNLEWTK	134	N
	[gi|164687722]					
9	Hypothetical protein CPE1535 [gi|18310517]	1193.57	0.018	SYWGVELSPR	62	**Y**
		1280.66	0.018	SIGTSVQSFLNK	59	**Y**
		1662.84	0.021	LCIILPGIGYCLDR	56	**Y***
		2073.97	0.037	EIGYDVLEIEYGYQINR	155	**Y**
		2997.50	0.052	TLDEFNIESNSLVITGDKDPLLSIAHR	102	**Y**
10	Hypothetical protein CPF_0363 [gi|110800590]	1119.58	0.004	FTPAFYVFK	42	**Y**
		1757.86	0.019	THASPHLFLFTEETK	101	**Y***

		1928.86	0.023	ENFWEGGSVSNFSPIMK	75	**Y***
		1965.86	0.020	SFNYENMNLIDYSDLK	59	**Y***
11	Hypothetical protein CPE0689 [gi|18309671]	933.53	0.016	AFEGLLKR	29	N
		1215.57	0.026	IAFEEAEHAAK	80	N
		1395.66	0.030	EGFPEVAEAYKR	103	N
		1538.70	0.038	FAEMLGEVVEADTK	128	N
		1709.77	0.040	QADREGFPEVAEAYK	71	N
		1720.75	0.041	FVEQSGDLAFADEHR	112	N
		2395.04	0.066	QLNLDAIHDTVHEMCKDEAR	65	N
		2690.18	0.077	IIEGLQANFTGECTEVGMYLAMSR	138	N
		2750.15	0.073	CPICGAGADKFVEQSGDLAFADEHR	99	**Y***
12	Hypothetical protein CPE0689 [gi|18309671]	1395.66	0.027	EGFPEVAEAYKR	102	N
		1720.75	0.035	FVEQSGDLAFADEHR	112	N
13	Hypothetical protein CPE0082 [gi|18309064]	1395.66	0.009	EGFPEVAEAYQR	102	**Y***
14	Hypothetical protein CPF_1179 [gi|110799882]	1515.80	0.051	IVPFGGGFPIIVDGK	68	**Y***
15	Molecular chaperone DnaK [gi|110802609]	986.46	0.039	HIDMTLTR	65	N
		1022.41	0.039	LGGDDFDQR	68	N
		1129.59	0.044	FTLSGIAPAPR	63	N
		1226.58	0.048	IIDYIAEDFK	81	N
		1316.56	0.050	AMEDLTQAFYK	80	**Y***
		1372.63	0.055	FNELTHDLVER	72	N
		1493.66	0.054	TTPSVVSFQANGER	67	N
		1636.76	0.063	DYTPQEISAMILQK	86	N
		1675.82	0.068	IINEPTAASLAYGLDK	88	N
		1894.87	0.081	VTEAVITVPAYFNDAER	101	N
		2044.00	0.095	SGNVSLNDIDKVILVGGSTR	119	**Y**
		2094.97	0.095	IIDYIAEDFKAQNGIDLR	69	N
		2516.11	0.121	SQIFSTAADNQTSVEIHVVQGER	67	N
		2644.20	0.130	KSQIFSTAADNQTSVEIHVVQGER	144	N
		2818.47	0.142	DVLLLDVTPLTLGIETLGGVATPLIER	152	N
		1571.75	0.062	AKFNELTHDLVER	76	N
		2263.05	0.108	VNIDGKDYTPQEISAMILQK	130	N

16	Phosphoenolpyruvate-protein phosphotransferase	1659.80	0.057	VSYLYNPMHPAVLR	28	N
	[gi|18311339]	2150.01	0.081	TLPYLPLPEEMNPFLGYR	40	N
17	Mal A [gi|1149672]	1277.63	0.038	FGLLLQDEASGK	52	**Y***
		1433.71	0.044	LSFNVGVIDLNDK	75	**Y***
		1469.59	0.049	TLDDMMTPFNER	42	**Y***
		1745.89	0.061	LSFNVGVIDLNDKVGR	65	**Y***
		2909.35	0.124	GTGIEIENFEHQKPLYFSPDHPLIK	116	**Y***
18	Nitrite/sulfite reductase-like protein [gi|18309529]	1039.52	0.035	IYTYNLPR	47	**Y**
		1209.55	0.036	LGMDENLYVR	68	**Y***
		1446.57	0.040	MGEEEFKNCFR	59	**Y**
19	Dipeptidase PepV [gi|110799340]	1088.57	0.035	GINAIMQTIK	60	**Y***
		1277.63	0.038	FGILLQDEASGK	52	**Y***
		1315.62	0.041	VFGVSAHGSTPEK	53	**Y**
		1338.58	0.039	DIEYYLEHEK	71	**Y**
		1433.71	0.044	LSFNVGVIDLNDK	75	**Y***
		1469.59	0.049	TLDDMMTPFNER	42	**Y***
		1587.75	0.045	FEVSHKNDEIELK	82	**Y**
		1612.69	0.051	FLNDNIGEDVYGEK	94	**Y**
		1745.89	0.061	LSFNVGVIDLNDKVGR	65	**Y***
		1766.83	0.064	GTTDDKGPIMASLYGLK	87	**Y***
		1973.92	0.061	APVAGFTPDAEFPIINGEK	65	**Y***
		2030.96	0.075	IDEMKDDLIESVQNIIR	134	**Y***
		2909.35	0.124	GTGIEIENFEHQKPLYFSPDHPLIK	116	**Y***
20	Peptidase T [gi|110800422]	1424.73	0.031	QCGIEPIKNPIR	51	**Y***
		1655.76	0.023	IGELEYENFNAASAK	96	N
		1712.90	0.033	ILAEELKEIGVDEVR	53	**Y***
		2292.07	0.043	LSFMGLPTPNLFTGGENFHGR	66	**Y***
21	Metallopeptidase, family M24 [gi|18311479]	1225.65	0.047	AHFLIQLLDR	51	**Y**
		1647.72	0.062	IEDLVLVTEDGCER	50	N
22	Glucose-6-phosphate isomerase [gi|18311249]	945.47	0.030	LVYFFEK	63	N
		1039.51	0.038	ILKDYMEK	38	N
		1250.59	0.046	SGTTTEPAIAFR	105	N

		1446.73	0.026	DIFETVINVGSPR	73	**Y***
		1602.79	0.064	RDIFETVINVGSPR	78	**Y***
		1679.81	0.064	NMFALLGKPGFEDLK	73	N
		1999.84	0.088	SHEVAYMQETINQAHNK	123	**Y**
		2124.96	0.042	TLADAEGYETFVIPDDVGGR	67	N
		2303.02	0.112	EEIVIEANDENIDGLNFLAGK	187	**Y***
		2583.14	0.143	ACGISGYVLGINPFDQPGVEAYKK	88	**Y**
		2639.12	0.133	GLFPAAVDFSTDLHSMGQYIQEGR	144	N
		3070.40	0.158	GTLLAHNDGEVPNVVVNVPELTPYYFGR	145	**Y**
		3121.30	0.164	LHNGTGAGNDFLGWVDLPVNYDKDEFAR	107	**Y**
23	Triosephosphate isomerase [gi|18310284]	999.53	0.032	TPIIAGNWK	81	**N**
		1018.50	0.029	GAFTGEIAPR	47	N
		1289.55	0.037	VGAQNMHFEEK	61	N
		1561.73	0.044	TATSDQANETIAAIR	55	N
		1616.85	0.048	VVIAYEPIWAIGTGK	90	N
		1780.81	0.053	AMVAEMFGQEVADKVR	107	**Y**
		1934.94	0.058	IQYGGSVKPNTIAEQMAK	114	**Y**
		1990.90	0.064	MLEAMNIDYVIIGHSER	96	N
24	Peptidyl-prolyl cis-trans isomerase [gi|18311548]	1755.97	0.027	IKIELYPEIAPNTVR	58	**Y***
25	Glutamate decarboxylase [gi|18311040]	1514.74	0.054	ADLSHNLAELYLR	60	**Y***
		1659.77	0.069	SASQIIGQYYNFLR	53	N
26	Elongation factor Tu [gi|18311389]	1152.58	0.022	TVVTGIEMFR	45	N
		1668.86	0.026	LLDEAQAGDNIGALLR	90	N
		1735.79	0.023	ELMDAVDSYIPTPER	68	N
		1788.85	0.035	GITINTAHVEYETANR	56	N
		1950.97	0.041	GVLHVGDEVEVIGLTEER	36	**Y**
		2137.04	0.059	ATDKPFLMPVEDVFTITGR	28	N
27	Flavoprotein [gi|18309270]	1259.58	0.021	AEFFDQYIAR	49	**Y**
		1724.80	0.035	IAGAFGSYGWSGEAVPR	69	N
		1759.91	0.034	GLDIEIICPGHGPILR	47	**Y***
28	Ornithine carbamoyl transferase [gi|1321787]	948.44	0.034	DFTPAEIR	56	N
		1022.44	0.036	EVEDEVFR	43	N

		1030.42	0.036	MYDGIEFR	40	N
		1279.54	0.043	NSVVFDEAENR	78	N
29	Phosphopyruvate hydratase [gi|18310281]	1318.68	0.054	QYIEIIDVVAR	30	**Y**
		1421.63	0.052	INEELGEVAEYR	38	**Y**
30	ABC transporter [gi|15595885]	1229.53	0.057	FATNSTFTNAR	50	N
		1746.81	0.091	TTWGQLLGTTDSTPIR	30	N
		2606.15	0.163	VNGNLPTQANVSTALGSVAPPANAADR	147	N
31	Maltose ABC transporter [gi|18311325]	1324.62	0.060	GYQFVQDLVQK	35	**Y**
		1788.77	0.120	KSGFYISGPWDVSAFK	64	**Y***
32	Choloylglycine hydrolase family protein	988.59	0.019	GLAGGLLFLK	90	**Y***
	[gi|110800845]	1402.78	0.026	LFSILNSVWVPK	85	N
		1464.68	0.025	GNPITAHYTFTDK	78	N
		1478.73	0.026	NFTLETQSKPWK	92	**Y***
		1513.72	0.027	NLIGDITDEEAPAR	139	**Y***
		1541.72	0.025	TLDTSFNPFEVGSK	83	**Y***
		1809.84	0.023	MGESVVLEPTNHGHFR	82	**Y***
		2013.95	0.046	EFSLENVKENELVTYSI	79	**Y***
		2086.98	0.036	NMDIEYSFNQSIIFIPR	134	**Y***
		2628.32	0.040	LGDQSLTALGQGTGLVGLPGDFTPASR	132	**Y***
		2070.94	0.046	CTHIHISSVNNNLYWGR	79	**Y***
		2417.21	0.045	TVITPISNGTGLLGLPGDYTSPSR	112	**Y***
		914.46	0.019	DGLHLFGR	295	N
33	Di-hydrodipicolinate synthase [gi|110798880]	938.51	0.027	LLEWHIK	32	N
		1212.59	0.032	YFLGEVIESR	69	**Y**
		1638.73	0.020	TAMNLMNFNCGPLR	23	**Y**
		2019.06	0.047	YLSLANSLFIETNPIPVK	76	N
		2498.14	0.081	GSCVALITPFTEDGVNYEELRK	60	**Y**
		2816.33	0.118	GLYAHFNAINDAINIPIMLYNVPSR	75	**Y***
34	Fructose-1,6-bisphosphate aldolase [gi|18310332]	1796.84	0.047	YAVGQFNINNLEWTK	62	N
		1852.84	0.043	INVNTECQLSFAEATR	45	N
		1891.91	0.052	YPANWAGLNFEALANIK	126	**Y**
		1993.97	0.099	QIAELGVTMLAAGIGNIHGK	81	N

35	Fructose-1,6-bisphosphate aldolase [gi|18310332]	1052.48	0.030	MELFGSVNR	71	N
		1101.59	0.032	LLNPGFEAIK	67	N
		1123.52	0.030	MELFGSVNRA	61	N
		1229.69	0.033	KLLNPGFEAIK	65	N
		1309.61	0.037	EKMELFGSVNR	108	N
		1534.74	0.036	TIVGMVNGMLEELK	100	N
		1796.83	0.058	YAVGQFNINNLEWTK	134	N
		1852.82	0.058	INVNTECQLSFAEATR	137	N
		1891.89	0.071	YPANWAGLNFEALANIK	149	**Y**
		1993.02	0.065	QIAELGVTMLAAGIGNIHGK	154	N
		2295.18	0.074	AILLTAQENNSPVILGVSEGAAK	140	N
		3008.43	0.110	AATGDMPLVLHGGTGIPSDMIAEAISLGVSK	121	**Y**
36	Deoxyribose-phosphate aldolase [gi|18311034]	1388.66	0.016	GADEVDMVINIGR	102	N
		1568.72	0.017	TSTGFSTGGATPEDIK	81	N
		1576.79	0.019	VIIETCLLTEEEK	59	N
37	Glutaminase [gi|110798927]	1470.76	0.036	NKPSNPFINAGAIK	66	**Y**
38	Purine nucleoside phosphorylase [gi|18311477]	2103.89	0.075	EGVYAMFSGPTYETPAEVR	78	**Y**
39	Tetra-hydro-dipicolinatesuccinylase [gi|18310891]	868.49	0.029	IEPGAIIR	35	N
		1475.64	0.039	SYNFTDPYEIAR	58	**Y**
		1654.84	0.050	NSAIPMLDLLNINAR	83	**Y***
40	Rubrerythrin [gi|1200049]	961.42	0.028	AETAHYNR	53	**Y**
		1095.47	0.035	YTYFSSTAR	70	**Y**
		1808.74	0.066	CGNCGFIWEGAEAPLK	116	**Y**
		1866.85	0.016	CPACLHPQAYFEVFK	84	**Y***
		2212.01	0.099	KEGYVQISNIFLETAENEK	140	**N**
		2259.99	0.026	CPACLHPQAYFEVFKETY	104	**Y***
		3317.63	0.078	FLKDDLQGEAVEINAAYPVELPTDTLTNLK	156	**Y**
		3686.55	0.097	FAADGEHDELSNLYPSFADVADEEGFPEVAA AFR	181	**Y***
		1215.57	0.026	IAFEEAEHAAK	80	**N**
		1395.66	0.030	EGFPEVAEAYKR	103	**N**
		1538.70	0.038	FAEMLGEVVEADTK	128	**N**

		1709.77	0.040	QADREGFPEVAEAYK	71	**N**
		1776.77	0.038	FIEQGEDLAFADEHR	97	**Y***
		2395.04	0.066	QLNLDAIHDTVHEMCKDEAR	65	**N**
		1865.86	0.009	QADREGFPEVAEAYQR	52	**Y***
41	Flavodoxin [gi|18311044]	1787.77	0.054	IEQYDMEPFVNQFK	101	**Y**
		2202.03	0.003	VEDVLEADAVALGSPSMDNNR	136	**Y**
42	Thioredoxin [gi|18311337]	1230.70	0.017	VVDTLVGFRPK	60	**N**
43	Glutaredoxin [gi|18311419]	1879.85	0.030	NVEFEDLNVQTDMVAR	111	**N**
		1500.63	0.029	VYSTNSCPWCVK	75	**N**
		1879.85	0.034	NVEFEDLNVQTDMVAR	98	**N**
		2016.05	0.029	QMGVPVLDINGTIIVGFDK	92	**N**
		1757.93	0.033	SVPVAVIGEEVIVGFDK	82	**Y**
44	Heme oxygenase [gi|18309196]	1011.49	0.033	YLADLFGGR	34	**N**
		1151.57	0.033	DFVLPEIYR	53	**Y**
45	Putative PTS system [gi|110798827]	1577.84	0.022	LHTPVLITNPDMVK	89	**Y**
		1689.84	0.026	TIDLSQVPDQVFAEK	83	**Y**
46	Putative endoribonuclease L-PSP [gi|18309994]	1544.69	0.042	TTVFLSDMNDFVR	32	**Y**
		1933.82	0.054	MNEVYATYFTENQPAR	52	**Y**
47	Putative endoribonuclease L-PSP [gi|18309994]	1128.66	0.027	IEIEVIATLK	60	**N**
		1258.66	0.017	APGAIGPYSQGIK	99	**N**
		1544.71	0.023	TTVFLSDMNDFVR	118	**N**
		1933.83	0.042	MNEVYATYFTENQPAR	127	**Y**
48	30S ribosomal protein S8 [gi|18311373]	1344.69	0.026	VMTDPIADLLTR	33	**N**
		1595.67	0.040	GVEEYNDGVVPMMR	54	**Y**

[b]N = Not a unique peptide; Y= peptide unique to the genus in global BLAST; **Y** (bold) = peptide unique to the species in global blast; Y* = peptide unique to the species with one exception.

Supplementary Table S1-F: Protein identified by tandem MS analysis in the cell lysate of *Clostridium tetani*.

S.No	Protein [accession no]	Observed m/z (Da)	Error (Da)	Peptide	Ion score	Unique
1	Putative S-layer protein [gi|28210215]	976.56	0.021	VVVYVGNVK	77	**Y**
		1235.62	0.041	GVPVPTFEYVK	42	**Y**
		1447.72	0.052	DSLLVVFSEPVSR	68	**Y**
		1491.68	0.060	SVNDYVYILYSR	82	**Y**
		1686.80	0.068	GGYAFTALIELPGYSK	74	**Y**
		1779.80	0.069	FEAINDGVDTTVETLR	101	**Y***
		1862.77	0.056	YNVFYGENGNKDQWK	38	**Y**
		1935.89	0.074	QVTQDADITAEDVVLYR	122	**Y**
		2077.98	0.081	NILIDNQYNYYHVLAPK	82	**Y**
		2089.06	0.093	NVYIVGGEGVVKPSIESTLK	100	**Y***
2	Putative S-layer protein [gi|28210213]	940.45	0.038	IYINFDR	47	N
		1136.52	0.041	GADRFDTNLK	41	N
		1294.57	0.054	FTSADQDLIER	86	Y
		1740.77	0.056	DNTIIYTLTDEGTER	71	**Y***
		1793.80	0.047	VVFDGKVDEDTAEDVR	76	**Y***
		1149.58	0.051	IAILFGQEMK	58	**Y**
		1257.54	0.052	IGFEADYSEVK	90	**Y**
		1422.63	0.054	DAYGNKIEDELR	72	**Y***
		1468.70	0.067	VSHVLTVDDNTIR	70	**Y***
		1537.70	0.047	DGEYLADASGWIIK	104	**Y***
		1655.85	0.039	ADVQSIEALNLNQIK	48	**Y***
		2702.23	0.092	KVTIEFSEAIFVDENTQYDALNR	111	**Y***
		1149.57	0.055	IAILFGQEMK	62	**Y**
		2181.98	0.068	VEDVIDLAGNVMDDYTATIK	131	**Y***
		2307.07	0.094	DKFQGIYHNEIAPELLYEK	104	**Y***
		2574.13	0.105	VTIEFSEAIFVDENTQYDALNR	43	**Y***
		2702.20	0.125	KVTIEFSEAIFVDENTQYDALNR	124	**Y***
3	Hypothetical protein CTC00463	1155.53	0.020	NGYIEYGAIR	45	**Y**

	[gi|28210214]	1862.81	0.043	AYNDDKNGYIEYGAIR	23	**Y**
4	Hypothetical protein CLOL250_01393	1923.90	0.067	IGVFEDENLLFEETLR	80	N
	[gi|160893839]					
5	Hypothetical protein CTC00681	2044.98	0.077	ILEPGWHFTIPFADFVR	64	N
	[gi|28210405]	2123.02	0.082	QQILDIEPQNVITQDNVR	93	N
6	Hypothetical protein CA_C1717	1487.76	0.066	LINIGFGNIVSANR	56	N
	[gi|15894994]	2471.20	0.121	AVIITDSDHVILSAVQPETVAHR	77	N
7	Pyruvate-flavodoxin oxidoreductase	1241.55	0.045	FQDFLMGEVR	60	N
	[gi|28211392]	1345.66	0.025	IPFLSFFDGFR	46	N
		1430.62	0.032	AVDCGYWQLYR	53	N
		1565.81	0.067	IAGELLPGVFHVSAR	68	N
8	Rubredoxin/rubrerythrin [gi|28210543]	1215.55	0.065	GVDEEVIEGLR	41	N
9	Rubrerythrin [gi|28212206]	1107.52	0.021	YTYYASIAR	50	N
		1651.79	0.037	VADEEGFPTIATVFR	120	**Y***
		2275.90	0.076	YAADGENEEWSELYPEFAR	129	**Y**
10	Molecular chaperone DnaK [gi|28211653]	2445.08	0.115	SQVFSTAADGQTSVEIHVVQGER	126	N
11	60 kDa chaperonin [gi|161486837]	1425.59	0.054	AQIEETTSEFDR	51	N
		1544.77	0.066	FGAPLITNDGVTIAR	59	N
		2102.01	0.097	TNDVAGDGTTTATLLAQAIIR	45	N
12	Cobyric acid synthase [gi|28210441]	1668.80	0.051	FQLEDEDGAIDFNR	34	**Y**
		1899.96	0.051	FDIVVIEGAGSPAEINLR	74	N
		2198.02	0.073	ISNFTDFDALKVEEDVSIR	46	**Y***
13	Elongation factor Tu [gi|28212176]	2073.94	0.058	ADQVDDAELIELVEMEVR	162	**Y***
		2151.05	0.063	ATDKPFLMPVEDIFTITGR	117	N
		1141.60	0.024	SFTGQVYVLK	52	N
		1152.58	0.026	SVITGIEMFR	53	N
		1353.75	0.031	VGVEHIVVFLNK	80	N
		1668.84	0.043	LLDEAQAGDNIGALLR	80	N
		1796.93	0.051	KLLDEAQAGDNIGALLR	33	N
		1835.82	0.047	GITISTSHVEYETENR	77	N
		1977.89	0.045	HTPFFNGYRPQFYFR	29	N
		2089.94	0.049	ADQVDDAELIELVEMEVR	37	**Y***

		2167.05	0.059	ATDKPFLMPVEDIFTITGR	46	N
		2308.05	0.057	ELMNEYGFPGDDAPVVVGSALK	106	**Y**
		2715.24	0.078	NMITGAAQMDGAILVVSAADGPMPQTR	89	N
		890.40	0.033	VAMDEGLR	34	N
14	Methylaspartate ammonia-lyase [gi|28212141]	1162.62	0.038	LLAYVEWLR	56	N
		2249.96	0.092	IVDVLCTPGFTGFYFDDQR	133	N
		2339.99	0.093	GAGHDGFTYTGEPVTEGFTQIR	93	**Y**
15	Methionine gamma-lyase [gi|28212109]	1867.88	0.080	DMTGSVLSPFDAFLVIR	73	N
16	Phosphopyruvate hydratase [gi|28210138]	1292.63	0.046	NYIEIIDVSAR	58	N
		1774.87	0.062	AAVPSGASTGIFEAVELR	47	N
17	3-hydroxybutyryl-CoA dehydratase	1776.89	0.042	ELIYTADIIGAEEALR	76	**Y**
	[gi|28212012]	1917.92	0.054	FGQPEVGLGITPGFGGTQR	85	N
		2278.22	0.090	IAVLTINRPDALNALNSELLK	29	**Y**
18	Fumarate hydratase [gi|28212139]	849.35	0.039	MDPYAPR	28	N
		1537.75	0.065	AGDSVLISGVIYTSR	83	N
		1836.82	0.091	KAEIVCYEDLGAEALR	69	N
		2927.37	0.162	RLEVEDLPVVVVIDSEGNNLYEIGQK	63	N
19	Thiolase [gi|168183516]	1538.69	0.056	KGEVVFDTDEFPR	104	N
		1888.91	0.078	GLATLCIGGGMGTAIIVER	86	N
		1920.89	0.074	VNVNGGAIALGHPIGCSGAR	33	N
20	Acetyl-CoA acetyltransferase [gi|28210073]	1538.69	0.056	KGEVVFDTDEFPR	104	N
		1455.76	0.054	AGIPVEVPAFTLNK	74	N
		1596.71	0.058	GLDPAYMGYGPVGATK	84	N
		1906.88	0.069	VNVNGGAIALGHPVGCSGAR	57	N
		2589.13	0.104	AGDADVVIAGGMENMSAAPYVLPNAR	112	**Y**
		2762.35	0.131	AGIKPEIIDEVIMGNVIQAGLGQSPGR	152	N
		1557.57	0.054	EMQDEFACASQNK	60	N
		1888.91	0.078	GLATLCIGGGMGTALLVER	86	N
		2017.00	0.089	KGLATLCIGGGMGTALLVER	82	N
		2333.09	0.093	VEDLDLIEANEAFASQSLAVAK	141	N
		2557.97	0.117	DGLWESFNDYHMGMTAENIAEK	109	N
		2778.36	0.115	AGIKPEIIDEVIMGNVIQAGLGQSPGR	45	N

21	Phosphate butyryltransferase [gi|28212125]	1794.81	0.065	LMSHVAVFETDKFDR	31	N
		1105.56	0.033	GLVDTANFLR	53	N
		1794.82	0.061	LMSHVAVFETDKFDR	23	N
22	Adenosyltransferase [gi|28210464]	1731.81	0.091	APEILPNFTLEQFGR	38	N
23	Butyrate kinase [gi|28212124]	1449.63	0.044	YSNVYEQFPFR	47	N
		1923.90	0.067	IGVFEDENLIFEETLR	80	Y*
		1943.89	0.042	IIDVNNALDGEGPFSPER	52	N
		2471.23	0.115	FIAPVVLYPGEDELLALTQGGLR	87	Y
24	Ribose-phosphate pyro phosphokinase	1386.70	0.039	ITAVIPYYGYAR	20	N
	[gi|28209957]					
25	Adenylate kinase [gi|28212159]	1908.98	0.082	TDAALLIDVPQELILER	40	N
26	Aspartate-semialdehyde dehydrogenase	1640.83	0.047	KFPVDNLYLFASAR	92	**Y**
	[gi|28211890]					
27	Glyceraldehyde 3-phosphate dehydrogenase	1796.85	0.056	GILGYSEEPLVSVDYR	54	N
28	3-hydroxybutyryl-CoA dehydrogenase	1150.54	0.027	DIKDEFVER	45	N
	[gi|28212008]	2382.15	0.116	EVSVAIGKDPVEVAEAPGFVVNR	120	N
		1365.56	0.045	MAQEDMDSILGR	58	**Y**
		1384.59	0.048	GMATSQETFDAVK	26	N
29	Putative electron-transfer flavoprotein	1091.53	0.029	GVWVFAEQR	57	N
	[gi|1055221]					
30	Electron transfer flavoprotein [gi|28210434]	1593.76	0.039	IDSIAEELVAYGADK	93	N
		1677.77	0.049	NVMDIGEAEFLVAGGR	113	**Y**
		1693.77	0.046	NVMDIGEAEFLVAGGR	70	**Y**
		1758.85	0.053	MLEELAELLGGNVAGSR	124	**Y***
		2184.02	0.072	VIYAQNPLLSHYTTDGYTK	117	**Y**
		2558.28	0.104	VICDLVAEEKPEIILVGATYIGR	151	N
		1091.53	0.031	GVWVFAEQR	52	N
		1296.65	0.044	KLDYDIVFAGR	89	N
		1730.84	0.063	VTVITMGPQQAESTLR	36	**Y**
		1168.56	0.034	LDYDIVFAGR	55	N
		1252.51	0.041	AWEDGYENIR	77	N
		1296.65	0.042	KLDYDIVFAGR	95	N

		1337.59	0.039	TWNADFLEVDK	65	**Y**
		1408.61	0.047	RAWEDGYENIR	41	N
		1535.69	0.047	YMHVAHIFGAFDK	97	N
		1690.77	0.058	EALSMGADEAILISDR	108	N
		1730.84	0.062	VTVITMGPQQAESTLR	119	**Y**
		1763.88	0.067	AFAGADTLATATALSAAIK	102	**Y**
		1905.89	0.070	YMHVAHIFGAFDKEIK	74	N
		2082.06	0.079	QVPDTTEVKIDPVTGTLIR	74	N
31	Electron transfer flavoprotein [gi|28210433]	1252.54	0.017	AWEDGYENIR	61	N
		1296.66	0.032	KLDYDIVFAGR	56	N
32	Membrane lipoprotein tmpC [gi|28210125]	1258.54	0.014	YVDSFEDQQK	39	**Y**
		1275.59	0.033	QQEQYQPNLK	43	N
		1811.83	0.031	FETGFAAGVMSVNPEAGK	76	**Y**
		2412.09	0.087	MLYNSGCDIVFHAAGGVGLGVFK	121	N
		2509.17	0.146	TPNTLSITFKEEEGSFLAGVIAGK	35	**Y**
33	30S ribosomal protein S2 [gi|28210947]	1127.51	0.043	MAPYIFTER	56	N
		1267.63	0.047	KVEQAYNFIR	61	**Y**
		1730.81	0.045	NLNANNVGAMFVVDPR	31	**Y**
		1371.63	0.052	QAQEAIQEEAVR	70	N
		1592.76	0.061	QLLEAGVHFGHQTR	39	N
		1730.81	0.054	NLNANNVGAMFVVDPR	60	**Y**
34	30S ribosomal protein S4 [gi|28212155]	1063.54	0.043	ISNYGLQLR	45	N
		1157.50	0.043	GYAPGQHGQSR	19	N
		1313.59	0.053	RGYAPGQHGQSR	25	N
		1557.80	0.061	GITGENLLVLLEMR	48	**Y***
		1860.84	0.069	TLPNWLEGNLENFEGK	87	N
35	30S ribosomal protein S3 [gi|28212171]	1549.69	0.030	NFSDNLVEDNNIR	71	N
		1804.87	0.050	TEFYHEGTIPLQTLR	62	N
36	SSU ribosomal protein S30P [gi|28210085]	1712.91	0.074	QIIEVTIPFNGVILR	40	N
		1852.86	0.101	YFKPDVEAQVTLSVEK	80	**Y**
37	Ribosomal protein S5 [gi|341904759]	1112.55	0.053	EGTGVIAGGPVR	45	N
		1290.59	0.055	NMVNATINGLSR	29	N

		2514.12	0.141	NLVSVQMVGTTVPHNIEGEFGTGK	96	**Y**
38	30S ribosomal protein S7 [gi|126697636]	1784.87	0.056	VGGANYQVPIEVRPER	63	N
39	30S ribosomal protein S9 [gi|28212147]	1656.71	0.072	RDEEEYFGLETLR	86	**Y**
		2250.07	0.109	DKFDVLVNVYGGGLTGQAGAIR	184	**Y**
		943.42	0.041	VQYYGTGR	45	N
		1381.78	0.060	VIVNQPLVLTGTK	66	N
		1500.62	0.065	DEEEYFGLETLR	85	**Y**
40	30S ribosomal protein S12 [gi|28212179]	2966.38	0.155	LTNGYEVSAYIPGVGHNLQEHSVVLIR	139	N
41	30S ribosomal protein S11 [gi|28212156]	1342.73	0.058	SLQAAGLEVTLIK	98	N
		2928.32	0.126	STFNNSIVTLTDTAGNALSWASAGGLGFR	156	N
42	30S ribosomal protein S13 [gi|28212157]	1557.78	0.066	IKDLSEEEVNLLR	102	**Y**
		1897.95	0.081	RVEVGLTYIFGIGTSSAK	74	**Y**
43	30S ribosomal protein S8 [gi|28212165]	1344.67	0.040	VMTDPIADLLTR	61	N
		1537.75	0.051	KLSIGGEVLCYIW	43	N
44	50S ribosomal protein S10 [gi|15896384]	1395.74	0.067	LDLPAGVDIEIKL	75	N
		1474.64	0.070	AFDHTILDQSAEK	92	N
		1904.02	0.097	VAGPVPLPTEKDVVTILR	112	N
45	30S ribosomal protein S10 [gi|15896384]	1395.74	0.063	LDLPAGVDIEIKL	72	N
		1474.65	0.062	AFDHTILDQSAEK	87	N
46	Ribosomal protein S15 [gi|341904758]	1900.88	0.080	SEGDTGSPEVQIALLTQR	107	N
47	50S ribosomal protein L1 [gi|28212183]	1363.63	0.051	ANFDETIDLAVR	111	N
		1834.80	0.064	EAEAAGADFVGAEELVEK	113	N
48	50S ribosomal protein L3 [gi|28212175]	1437.64	0.030	LGMTQIFDENNR	55	N
		1569.71	0.038	DGYEAIQVGYGEIR	72	N
		1635.76	0.062	LENVSEYQVGQEIK	87	**Y**
49	50S ribosomal protein L4 [gi|28212174]	875.47	0.022	PTVGLFNK	39	N
		1717.96	0.046	EVLHQVIVAQLANKR	59	N
		1996.98	0.056	VQENEIVILESLEADAPK	121	**Y**
		2255.16	0.074	NIEGVTVLPVNNINVYDILR	117	N
50	50S ribosomal protein L25 [gi|28210591]	1064.56	0.051	GFIPGIIYGK	49	N
		1079.46	0.047	EGQSINFER	47	N
		1265.61	0.057	WTIPIFFEGR	64	**Y**

		2168.04	0.102	DAVTSSIIHIDIQEVSALEK	105	**Y**
51	50S ribosomal protein L5 [gi|28212166]	919.39	0.050	FLGMPFAH	56	**Y**
		1011.55	0.055	EVIPALIEK	56	N
		1195.57	0.067	GMDIIFVTTAK	47	N
		2200.10	0.121	MLESAVSDLSIIAGQKPIVTK	136	**Y**
52	50S ribosomal protein L6 [gi|28212164]	1133.60	0.060	KLELVGVGYR	56	N
		1336.56	0.070	EGVEFQLDGTNK	80	**Y**
		1542.76	0.081	GIDKELVGAVASDIR	116	**Y**
		1738.77	0.096	SLVNNMVVGVTEGYEK	113	**Y**
		1765.88	0.098	LVLNLGFSHPVEIEAK	115	N
		1893.97	0.104	KLVLNLGFSHPVEIEAK	132	N
		2151.98	0.119	TMHPNIDIAIEENSVIVTR	101	**Y**
		2248.16	0.112	LPIAIPNGVTVTVSPENVVTVK	114	N
53	50S ribosomal protein L13 [gi|28212148]	1069.62	0.049	VASQVALILR	52	N
		1319.65	0.036	SYIAKPEEVQR	49	N
		1350.65	0.051	KPEFVFEEAVR	84	N
		1489.72	0.058	WYVVDAEGKPLGR	42	N
		1935.84	0.069	GSEHNNEAQNPEVLELR	65	**Y**
		2098.87	0.106	GSEHNNEAQNPEVLELRY	67	**Y**
		2474.10	0.148	NKPTYTPHVDTGDYVVIINAEK	102	N
		1617.80	0.064	KWYVVDAEGKPLGR	44	N
54	50S ribosomal protein L16 [gi|28212169]	1558.77	0.061	GTPEYWVAVVKPGR	67	N
		1578.75	0.049	VLFELTGVDEETAR	92	**Y**
55	50S ribosomal protein L10 [gi|61215282]	1520.76	0.078	APVSNFAYLINAIK	123	N
		2380.03	0.135	AECIVLADYQGLTVEEATELR	136	N
56	50S ribosomal protein L15 [gi|28212161]	1053.53	0.045	RGFTNIFAK	41	N
		1264.62	0.053	LHELRPAEGSR	48	N
		1864.82	0.083	SGGGVRPGFEGGQMPLYR	43	N
		2088.01	0.097	LNIFEDDTVVTPELLIEK	83	Y
57	LSU ribosomal protein L21P [gi|28204122]	1644.80	0.053	DNGEFVVGKPVVEGAK	62	N
58	50S ribosomal protein L18 [gi|28212163]	922.42	0.028	GGYIYHGR	42	N
		1161.53	0.033	NIFGTPEMPR	93	N

		1289.63	0.036	KNIFGTPEMPR	66	N
59	50S ribosomal protein L22 [gi|28212172]	1807.88	0.052	GKDVNEAFAILNYTPR	69	N
		956.42	0.041	YQPHAQGR	40	N
		1622.74	0.075	DVNEAFAILNYTPR	78	N
		1683.75	0.083	LYVAEAYACQGPTLK	103	N
		1699.75	0.073	SAVANAENNLELDPSR	54	N
		1807.84	0.088	GKDVNEAFAILNYTPR	93	N

[b]N = Not a unique peptide; Y= peptide unique to the genus in global BLAST; **Y** (bold) = peptide unique to the species in global blast; Y* = peptide unique to the species with one exception.

Supplementary Table S2 (A to F): Summary of putative marker proteins for the specific detection of selected species.

Supplementary Table S2-A: Summary of putative marker proteins for the specific detection of *B. anthracis*.

Score	Protein (accession no.)	Observed in crude lysate[a]	No. of times reported[b]	Localization (PSORT)[c]	Nearest homolog (% identity, Coverage, E-value)[d]	Remarks[e]	References
100	S-layer protein EA1	4	11	CW (10)	-	I,CE,EC	1-11
70	Protective antigen, PA	ND	6	EC (9.9)	*Brevibacillus sp. SKDU10** (52, 79, 0.0) *Paenibacillus forsythia* (29, 19, 9e-06)	I/EC/Vrl	12, 5-7,13,15
65	Lethal factor	ND	5	EC (9.9)	*Brevibacillu slaterosporus** (34, 40, 2e-39) *Anaerostipeshadrus* (35, 11, 4e-05)	EC/Vrl/I	2,6,7,13,15
60	Polysaccharide deacetylase, YxkH, YjeA	ND	5	M (3.3)	*Streptococcus pneumonia* (90, 100, 0.0) *Polysaccharide deacetylase* (55, 100, 8e-135)	C/I/W	1,2,7,9,15
60	ABC transporter, substrate binding protein	ND	4	C (7.5)	*Bacillus subtilis** (99, 100, 0.0) *Paenibacillus ehimensis* (61, 97, 9e-149)	I/CM	1,4,10, 12
60	Sulfatase, YvgJ, YflE	ND	4	CM (9.3)	-	I	2,4,5,7
53	60 kDa molecular chaperone GroEL	6	7	C (9.9)	*Streptococcus pneumonia** (99%, 100%, 0.0) *Bacillus sp. UNC322MFChir4.1* (97%, 96%, 0.0)	I,Vrl,SP,EC	2,3,5,7,9-11
50	Manganese superoxide dismutase, SodA-2	ND	9	EC (9.7)	*Bacillus wiedmannii** (97, 100, 6e-151) *Bacillus decisifrondis* (75, 98, 2e-117)	I/SP/EC	2-5, 7, 9-11, 15
45	Single-stranded DNA-binding protein	1	2	C (9.6)	*Bacillus gaemokensis** (98, 100, 4e-118) *Geobacillus sp. WSUCF1* (70, 100, 4e-77)	I	10,11
44	Universal stress protein	3	2	C (7.5)	*Bacillus gaemokensis** (97, 100, 3e-103) *Bacillus panaciterrae* (84, 100, 5e-90)	I	11,13
40	Alcohol dehydrogenase	1	2	C (9.9)	*Bacillus cytotoxicus** (94, 98, 0.0) *Enterococcus haemoperoxidus* (75, 98, 0.0)	SP	12
37	Elongation factor Tu, EF-Tu	3	3	C (9.9)	*Bacillus manliponensis**	I,SP	3,10,11

					(96, 99, 0.0)<br>*Bacillus panaciterrae*<br>(93, 100, 0.0)		
36	Histidinol-phosphate aminotransferase	1	1	C (7.5)	*Bacillus wiedmannii**<br>(96, 100, 0.0)<br>*Bacillus panaciterrae*<br>(69, 99,0.0)	SP	1
30	Enolase, Eno	ND	8	C (10)	*Streptococcus pneumonia**<br>(99, 99, 0.0)<br>*Bacillus sp. 491mf*<br>(95, 100, 0.0)	I/SP/C	2,3,5-7,9-12
31	Alkyl hydroperoxide reductase	3	6	C (9.6)	*Streptococcus pneumonia*<br>(99, 95, 2e-131)	I,Vrl,SP	2,3,5,7,11,12
30	Aconitate hydratase	1	3	C (9.9)	*Bacillus wiedmannii*<br>(99, 100, 0.0)<br>*Bacillus cytotoxicus*<br>(95, 100, 0.0)	I,SP	3,4,10
28	Elongation factor G, EF-G	1	3	C (9.9)	*Bacillus sp.*<br>(97, 100, 0.0)	I,C,SP	3,9,12
25	Cysteine synthase	1	4	C (7.5)	*Bacillus wiedmannii*<br>(98, 100, 0.0)<br>*Bacillus cytotoxicus*<br>(95, 100, 0.0)	SP	3,6
24	10 kDa chaperonin GroES	2	1	C (9.97)	*Bacillus gaemokensis**<br>(98, 100, 3e-55)<br>*Bacillus panaciterrae*<br>(96, 100, 4e-53)	SP	10
20	Formate acetyltransferase	2	1	C (9.9)	*Bacillus bingmayongensis*<br>(95, 100, 0.0)	I	11
20	Trigger factor, TIG	ND	5	C (7.5)	*Bacillus wiedmannii*<br>(97, 100, 0.0)<br>*Bacillus sp. UNC437CL72CviS29*<br>(95, 99, 0.0)	SP/I/C	3,7,9,11,12
19	Regulatory protein SpoVG	2	1	C (7.5)	*Bacillus manliponensis**<br>(99, 100, 1e-63)<br>*Bacillus oryziterrae*<br>(96, 100, 1e-61)	C	9
18	Isocitrate dehydrogenase	1	1	C (9.9)	*Bacillus wiedmannii**<br>(99, 100, 0.0)<br>*Bacillus cytotoxicus*<br>(97, 100, 0.0)	C	9
18	Pyruvate dehydrogenase E1, PdhA, PdhB, PdhD	ND	5	C (9.9)	*Streptococcus pneumonia*<br>(99, 95, 0.0)	I/SP/C	3,9-12
17	3-hydroxybutyryl-CoA dehydrogenase	1	1	C (7.5)	*Bacillus wiedmannii**	I	11

					(99, 100, 0.0)<br>*Bacillus sp. 123MFChir2*<br>(93, 100, 0.0)		
16	Glyceraldehyde-3-phosphate dehydrogenase	1	3	C (10)	*Bacillus gaemokensis**<br>(99, 100, 0.0)	I,EC	3,6,11
15	Molecular chaperone DnaK	1	7	C (9.97)	*Bacillus gaemokensis*<br>(98, 100, 0.0)<br>*Bacillus cytotoxicus*<br>(95, 100, 0.0)	I,SP,EC	2,3,7,9-12,15
15	Electron transfer flavoprotein	1	1	C (7.5)	*Bacillus wiedmannii**<br>(99, 100, 1e-179)<br>*Bacillus cytotoxicus*<br>(95, 100, 1e-163)	-	4
15	Isocitrate dehydrogenase	1	1	C (9.9)	Bacillus sp. B25(2016b)*<br>(99, 100, 0.0)<br>Bacillus cytotoxicus<br>(96, 100, 0.0)	C	9
11	Ribosomal protein S9	2	1	C (9.9)	*Streptococcus pneumonia*<br>(99, 98, 1e-85)	I	10
11	PTS system, glucose-specific IIA component	1	2	C (9.9)	*Bacillus bingmayongensis**<br>(94, 100, 2e-105)<br>*Streptococcus pneumonia*<br>(99, 86, 4e-93)	I	4,10
10	Formate acetyl transferase	1	1	C (9.9)	*Bacillus sp. 123MFChir2*<br>(95, 100, 0.0)	I	11
10	DNA-dependent RNA polymerase	1	2	C (9.9)	*Bacillus gaemokensis**<br>(97, 100, 0.0)<br>*Streptococcus pneumonia*<br>(95, 99, 0.0)	-	3,12
9	Major cold shock protein	2	2	C (9.97)	*Staphylococcus carnosus**<br>(96, 100, 8e-39)<br>*Bacillus gaemokensis*<br>(96, 100, 7e-38)	C	4,14
9	NADH dehydrogenase	1	1	CM (9.5)	*Streptococcus pneumonia**<br>(99, 100, 0.0)<br>*Bacillus gaemokensis*<br>(96, 100, 0.0)	SP	1

[a]Maximum number of times any peptide of the protein was observed in the present investigation using crude whole cell lysate of the organism using 1DE- or 2DE- MS/MS approach. ND = not detected in the MS/MS analysis of the present investigation.

[b]Number of times the protein has been described in the organism with an evidence of protein expression in open literature.

[c] **C =** cytoplasmic; **CM** = cytoplasmic membrane; **CW** = cell wall; **EC** = extracellular; **ML** = multiple localization; **U** = unknown.

[d] Asterisk indicates query species recurring after first non-species homolog. The second homolog is the closest after which the query species is not seen.

[e] **I =** immunogenic; **ID =** immunodominant in naturally infected clinical sera; **IP =**immunoprotective; **Vrl =** virulence associated; **S =** surface associated; **OM =** outer membrane; **CE** = cell envelope; **EC =** extracellular; **C =** cytoplasmic; **PP =** Periplasmic

References:

[1] Ariel N, Zvi A, Makarova KS, Chitlaru T, Elhanany E, Velan B, Cohe S, Friedlander AM, Shafferman A. Genome-based bioinformatic selection of chromosomal *Bacillus anthracis* putative vaccine candidates coupled with proteomic identification of surface-associated antigens. Infection and Immunity. 2003. 71 (8): 4563–4579.

[2] Chitlaru T, Gat O, Grosfeld H, Inbar I, Gozlan Y, and Shafferman A. Identification of in vivo-expressed immunogenic proteins by serological proteome analysis of the *Bacillus anthracis* secretome. Infection and Immunity. 2007. 75 (6): 2841–2852.

[3] DelVecchio VG, Connolly JP, Alefantis TG, Walz A, Quan MA, Patra G, Ashton JM, Whittington JT, Chafin RD, Liang X, Grewal P, Khan AS, Mujer CV. Proteomic profiling and identification of immuno-dominant spore antigens of *Bacillus anthracis*, *Bacillus cereus*, and *Bacillus thuringiensis*. Applied and Environmental Microbiology. 2006. 72 (9): 6355–6363.

[4] Francis AW, Ruggierob CE, Koppischa AT, Donga J, Songa J, Brettina T, Iyer S. Proteomic analysis of *Bacillus anthracis* sterne vegetative cells. Biochimica et Biophysica Acta. 2005. 1748 :191–200

[5] Walz A, Mujer CV, Connolly JP, Alefantis T, Chafin R, Dake C, Whittington J, Kumar SP, Khan AS and DelVecchio VG. *Bacillus anthracis* secretome time course under host-simulated conditions and identification of immunogenic proteins. Proteome Science. 2007. 5:11.

[6] Lamonica JM, Wagner MA, Eschenbrenner M, Williams LE, Miller TL, Patra G and DelVecchio VG. Comparative secretome analyses of three *Bacillus anthracis* strains with variant plasmid contents. Infection and Immunity. 2005. 73(6): 3646–3658.

[7] Chitlaru T, Gat O, Gozlan Y, Ariel N, Shafferman A. Differential proteomic analysis of the *Bacillus anthracis* secretome: distinct plasmid and chromosome CO2-dependent cross talk mechanisms modulate extracellular proteolytic activities. Journal of Bacteriology. 2006. 188 (10): 3551–3571.

[8] Kern VJ, Kern JW, Theriot JA, Schneewind O, Missiakasa D. Surface-Layer (S-Layer) Proteins Sap and EA1 Govern the Binding of the S-Layer-Associated Protein BslO at the Cell Septa of *Bacillus anthracis*. Journal of Bacteriology. 2012. 194 (15): 3833–3840.

[9] Gohar M, Gilois N, Graveline R, Garreau C, Sanchis V and Lereclus D. A comparative study of *Bacillus cereus*, *Bacillus thuringiensis* and *Bacillus anthracis* extracellular proteomes. Proteomics. 5: 3696–3711.

[10] Mukhopadhyay S, Akmal A, Stewart AC, Hsia R and Read TH. Identification of *Bacillus anthracis* spore component antigens conserved across diverse *Bacillus cereus* sensu lato strains. Molecular & Cellular Proteomics. 2009. 8 (6): 1174–1191.

[11] Liu X, Wang D, Ren J, Tong C, Feng E, Wang X, Zhu L, Wang H. Identification of the Immunogenic Spore and Vegetative Proteins of *Bacillus anthracis* Vaccine Strain A16R. Plos One. 2013. 8 (3). e57959.

[12] Huang CM, Foster KW, DeSilva TS, Kampen KRV, Elmets CA, Tang DC. Identification of *Bacillus anthracis* proteins associated with germination and early outgrowth by proteomic profiling of anthrax spores. Proteomics. 2004. 4: 2653–2661.

[13] Chenau J, Fenaille F, Caro V, Haustant M, Diancourt L, Klee SR, Junot C, Ezan E, Goossens PL and Becher F. Identification and validation of specific markers of *Bacillus anthracis* spores by proteomics and genomics approaches. Molecular and Cellular Proteomics. 2014. 13 (10): 716 – 732

[14] Lasch P, Beyer W, Nattermann H, Stammler M, Siegbrecht E, Grunow R, Naumann D. Identification of *Bacillus anthracis* by using matrix-assisted laser desorption ionization–time of flight mass spectrometry and artificial neural networks. Applied Environmental Microbiology. 2009. 75 (22): 7229–7242.

[15] Pflughoeft KJ, Swick MC, Engler DA, Yeo HJ, Koehler TM. Modulation of the *Bacillus anthracis* secretome by the immune inhibitor A1 protease. Journal of Bacteriology. 2013. 196 (2): 424–435.

Supplementary Table S2-B: Summary of putative marker proteins for specific detection of *B. melitensis*, *B. abortus*, and *B. suis*.

S. No.	Protein (accession no.)	Observed in crude lysate[a]	No. of times reported[b]	Localization (PSORT)[c]	Nearest homolog (% identity, Coverage, E-value)[d]	Remarks[e]	References
70	31-kDa outer-membrane immunogenic protein	ND	11	OM (10)	*Brucella inopinata** (99, 100, 9e-172) *Mesorhizobium* (55, 100, 8e-69)	OM,IP,CE,I	16,5,2,8,6,18,19,9,12, 13,14
56	DNA starvation/stationary phase protection protein, Dps	4	8	C (9.9)	*Brucella ovis** (99, 100, 5e-117) *Ochrobactrum* (94, 100, 2e-112)	I,ID,C	2,3,5-8,12,15
53	60 kDa Chaperonin, GroEL	8	12	C (9.9)	*Brucella ovis** (99, 100, 0.0) *Ochrobactrum* (97, 100, 0.0)	I,ID,Vrl,C,CE	1,2,4-7,9-13
50	Molecular chaperone DnaK	2	7	C (9.9)	*Brucella ceti** (99, 100, 0.0) *Rhizobiales bacterium 63-22* (95, 100, 0.0)	I,ID,Vrl,OM	1,2,6,10,11,14,16
48	Elongation factor Tu, EF-Tu	13	5	C (10)	*Brucella vulpis** (99, 100, 0.0) *Pseudochrobactrum sp. AO18b* (92, 100, 0.0)	I,ID,Vrl,C	1,2,3,4,5
37	Ribose-binding protein	ND	8	PP (9.7)	*Brucella ovis** (99, 100, 0.0) *Shinella sp. DD12* (88, 100, 0.0)	I	1,7,8,2,5,14,9,11
37	Glyceraldehyde-3-phosphate dehydrogenase	1	6	C (9.9)	*Brucella inopinata* (98, 100, 0.0)	I,ID,Vrl,C	2-4,8,9,13
36	ATP-dependent clp protease	1	5	C (9.9)	*Brucella ovis** (93, 100, 7e-153) *Ochrobactrum rhizosphaerae* (94, 100, 1e-146)	Vrl,C	2,4,5,7,
36	Fe/Mn family superoxide dismutase	1	6	EC (9.6)	*Brucella ceti** (99, 100, 8e-144)	I,ID,Vrl,PP	1,3,10,11,15,17
34	Amino acid ABC transporter substrate-binding protein	ND	7	PP (9.4)	*Brucella ceti** (100, 100, 0.0) *Rhizobiales bacterium* (91, 97, 0.0)	I	1,7,2,5,4,9,14
32	Immunogenic 39-kDa protein	1	2	U (2.5)	*Brucella vulpis** (99, 95, 0.0) *Ochrobactrum anthropic* (93, 95, 0.0)	I,Vrl,PP	5,7

31	Bacterioferritin	7	4	C (9.9)	*Ochrobactrum*<br>(94, 100, 8e-108)	Vrl,C	2,5,7,14
31	Aminotransferase	2	4	C (7.5)	*Brucella inopinata*<br>(99, 100, 0.0)	C	2,9,12,13
31	Ribosomal protein S1	ND	6	C (9.9)	*Brucella vulpis**<br>(99, 98, 0.0)<br>*Bartonella tamiae*<br>(89, 97, 0.0)	C/I	6,7,12,2,4
27	Isovaleryl-CoA dehydrogenase	ND	7	C (9.9)	*Brucella microti**<br>(99, 100, 0.0)<br>*Brucella vulpis*<br>(98, 100, 0.0)	C/I	3,10,2,5
26	Invasion protein B	1	2	U (2.5)	*Brucella ceti**<br>(99, 100, 4e-120)<br>*Ochrobactrum rhizosphaerae*<br>(79, 100, 2e-95)	-	2,15
26	Oxidoreductase	ND	6	U (4.4)	*Brucella ceti**<br>(99, 100, 0.0)<br>*Ochrobactrum intermedium*<br>(94, 100, 0.0)	PP/I/C	20,6,3,4,7,12
26	ATP synthase	ND	9	C (9.9)	*Brucella ovis*<br>(99, 100, 0.0)	C,I	6,12,5,13,2,4,9,14
22	Elongation factor G, EF-G	ND	6	C (9.9)	*Brucella sp. 10RB9215**<br>(99, 100, 0.0)<br>*Ochrobactrum*<br>(98, 100, 0.0)	C/I	5,6,2,4,13,17
21	Aldehyde dehydrogenase	8	3	C (9.6)	*Brucella ovis*<br>(99, 100, 0.0)	I,C	2,13
20	3-hydroxyisobutyrate dehydrogenase	1	3	C (9.2)	*Brucella inopinata**<br>(98, 100, 0.0)<br>*Ochrobactrum anthropic*<br>(90, 99, 0.0)	C,Vrl	2,5,7
17	10 kDa chaperonin, GroES	ND	5	C (9.9)	*Brucella ovis*<br>(98, 100, 1e-59)	C	7,10,12,6,2
17	Alcohol dehydrogenase	1	3	C (9.9)	*Brucella neotomae**<br>(99, 100, 0.0)<br>*Brucella inopinata*<br>(98, 100, 0.0)	C,Vrl	4,5,7,
16	Peptidoglycan-associated lipoprotein	1	1	CM (9.5)	*Brucella ceti**<br>(100, 100, 7e-120)<br>*Ochrobactrum*<br>(99, 99, 1e-118)	Vrl,PP	11
14	L-amino acid-binding periplasmic protein AapJ	1	3	PP (9.7)	*Brucella vulpis**<br>(99, 100, 0.0)	C,Vrl	2,5,7

					Ochrobactrum cytisi<br>(96, 100, 0.0)		
13	Ribosomal protein L9	1	3	C (9.9)	*Brucella ovis**<br>(99, 100, 7e-132)<br>*Ochrobactrum*<br>(96, 100, 2e-128)	I,C	2,9,12,14
12	Ribosome recycling factor	1	3	C (9.9)	*Brucella inopinata**<br>(99, 100, 3e-130)<br>*Brucella vulpis*<br>(98, 100, 7e-129)	I,C	2,4,6,7,
11	Biotin carboxyl carrier protein	1	1	C (9.8)	*Brucella ceti**<br>(97, 81, 3e-102)<br>*Brucella vulpis*<br>(94, 73, 6e-87)	I,ID	3
11	Glucose-6-phosphate 1-dehydrogenase	1	3	CM (8.1)	*Brucella neotomae*<br>(99, 100, 0.0)	I,ID,Vrl	2,4,6
11	Malate dehydrogenase	1	3	U (4.9)	*Brucella ceti**<br>(99, 100, 0.0)<br>*Ochrobactrum anthropic*<br>(99, 100, 0.0)	I,Vrl,CE	2,7,12
11	Universal stress protein	1	1	U (2.0)	*Brucella sp. 10RB9215*<br>(99, 100, 1e-99)	C	2
11	Aminopeptidase P	ND	4	C (9.2)	*Brucella ovis**<br>(99, 100, 0.0)<br>*Brucella inopinata*<br>(99, 100, 0.0)	C/I	2,4,6,12
	Aminopeptidase T	-	-	C (8.9)	*Brucella canis**<br>(99, 100, 0.0)<br>*Brucella inopinata*<br>(99, 100, 0.0)	-	-
	Aminopeptidase N	-	-	C (9.1)	*Brucella sp. CMUL 010**<br>(99, 100, 0.0)<br>*Paramesorhizobium deserti*<br>(72, 100, 0.0)	-	-

[a]Maximum number of times any peptide of the protein was observed in the present investigation using crude whole cell lysate of the organism using 1DE- or 2DE- MS/MS approach. ND = not detected in the MS/MS analysis of the present investigation.

[b]Number of times the protein has been described in the organism with an evidence of protein expression in open literature.

[c] **C =** cytoplasmic; **CM** = cytoplasmic membrane; **CW** = cell wall; **EC** = extracellular; **ML** = multiple localization; **U** = unknown.

[d] Asterisk indicates query species recurring after first non-species homolog. The second homolog is the closest after which the query species is not seen.

[e] **I =** immunogenic; **ID =** immunodominant in naturally infected clinical sera; **IP =**immunoprotective; **Vrl =** virulence associated; **S =** surface associated; **OM =** outer membrane; **CE** = cell envelope; **EC =** extracellular; **C =** cytoplasmic; **PP** = Periplasmic

References:

[1] Wareth G, Melzer F, Weise C, Neubauer H, Roesler U, Murugaiyan J. Proteomics-based identification of immunodominant proteins of *Brucellae* using sera from infected hosts points towards enhanced pathogen survival during the infection. Biochemical and Biophysical Research Communications. 2015. 456: 202–206.

[2] Wagner MA, Eschenbrenn M, Horn TA, Kraycer JA, Mujer CV, Hagius S, Elzer P, DelVecchio VG. Global analysis of the *Brucella melitensis* proteome: Identification of proteins expressed in laboratory-grown culture. 2002. Proteomics. 2: 1047–1060.

[3] Yang Y, Yin J, Guo D, Lang X, Wang X., 2010. Immunization of mice with recombinant S-adenosyl-L-homocysteine hydrolase protein confers protection against *Brucella melitensis* infection. FEMS Immunol Med Microbiol. 2010. 61: 159–167.

[4] Wang Y, Chen Z, Qiao F, Zhong Z, Xu J, Wang Z, Du Z, Qu Q, Yuan J, Jia L, Song H, Sun Y & Huang L. The type IV secretion system affects the expression of Omp25/Omp31 and the outer membrane properties of *Brucella melitensis*. FEMS Microbiol Lett. 2009. 303: 92–100.

[5] Eschenbrenner M, Wagner MA, Horn TA, Kraycer JA, Mujer CV, Hagius S, Elzer P and DelVecchio DG. 2002. Comparative proteome analysis of *Brucella melitensis* vaccine strain rev 1 and a virulent strain, 16M. Journal of Bacteriology. 2002. 184: 4962–4970

[6] Peng ZZ, Fang Y, Hui JW, Yan LD, Xin L, Li X, Qiang DY, Hui YP, Min SX, Zhong L, Liang WX., 2011. Identification of immune-reactive proteins of *Brucella melitensis* by immune-proteomics. Science China Life Sciences. 2011. 54.9: 880–887.

[7] Eschenbrenner M, Horn TA, Wagner MA, Mujer CV, Scandle TLM, DelVecchio VG. Comparative proteome analysis of laboratory grown *Brucella abortus* 2308 and *Brucella melitensis* 16M. Journal of Proteome Research. 2006. 5: 1731-1740

[8] Mujer CV, Wagner MA, Eschenbrenner M, Horn T, Kraycer JA, Redkar R, Hagious S, Elzer P, Vecchio V. Global analysis of *Brucella melitensis* proteomes. Ann. N. Y Acad. Sci. 2002. 969: 97-101

[9] Dahouk SA, Loisel-Meyer S, Schol HC, Tomaso H, Kersten NM, Harder A, Neubauer H, Kohler S and Jubier-Maurin V. Proteomic analysis of *Brucella suis* under oxygen deficiency reveals flexibility in adaptive expression of various pathways. Proteomics. 2009 (9): 3011–3021

[10] Dahouk SA, Nockler K, Scholz HC, Tomaso H, Bogumil R, Neubauer H, 2006. Immunoproteomic characterization of *Brucella abortus* 1119-3 preparations used for the serodiagnosis of Brucella infections. Journal of Immunological Methods. 2006. 309: 34– 47

[11] Lamontagne J, Butler H, Olarte EC, Hunter J, Schirm M, Paquet C, Tian M, Kearney P, Hamaidi L, Chelsky D, Moriyon I, Moreno E, Paramithiotis E. Extensive cell envelope modulation is associated with virulence in *Brucella abortus*. Journal of Proteome Research 2007. 6: 1519 -1529

[12] Connolly JP, Comerci D, Alefantis TG, Walz A, Quan M, Chafin R, Grewal P, Mujer CV, Ugalde RA, DelVecchio VG. Proteomic analysis of *Brucella abortus* cell envelope and identification of immunogenic candidate proteins for vaccine development. Proteomics. 2006. 6: 3767–3780.

[13] Dahouk SA, Maurin VJ, Scholz, Tomaso H, Karges W, Neubauer H, Köhler S. Quantitative analysis of the intra-macrophagic *Brucella suis* proteome reveals metabolic adaptation to late stage of cellular infection. 2008. Proteomics. 8: 3862–3870.

[14] Dahouk SA, Maurin VJ, Neubauer H, Köhler S. Quantitative analysis of the *Brucella suis* proteome reveals metabolic adaptation to long-term nutrient starvation. BMC Microbiology. 2013. 13:199.

[15] Pajuaba ACAM, Silva DAO, Almeida KC, Cunha-Junior JP, Pirovani CP, Camillo LR and Mineo JR. Immunoproteomics of *Brucella abortus* reveals differential antibody profiles between S19-vaccinated and naturally infected cattle. Proteomics 2012. 12: 820–831.

[16] Wang Y, Chen Z, Qiao F, Ying T, Yuan J, Zhong Z, Zhou L, Xinying, Du, Wang Z, Zhao J, Dong S, Jia L, Yuan X, Yang R, Sun Y, Liuyu, Huang. Comparative proteomics analyses reveal the vir B of *B. melitensis* affects expression of intracellular survival related proteins. Plos One. 2009. 4(4): e5368.

[17] Lee JJ, Simborio HL, Reyes AWB, Kim DG, Hop HT, Min W, Her M, Jung SC, Yoo HS, Kim S. Proteomic analyses of the time course responses of mice infected with *Brucella abortus* 544 reveal immunogenic antigens. FEMS Microbiol Lett. 2014. 357: 164–174

[18] Cassataro J, Velikovsky C, Bruno L, Estein S M, Barrera SDL, Bowden R, Fossati CA, Giambartolome GH. Improved immunogenicity of a vaccination regimen combining a DNA vaccine encoding *Brucella melitensis* outer membrane protein 31 (omp31) and recombinant omp31 boosting. Clinical and vaccine immunology. 2007. 14 (7): 869–874

[19] Gupta KV, Radhakrishnan G, Harms J, and Splitter G. Invasive Escherichia coli vaccines expressing *Brucella melitensis* outer membrane proteins 31 or 16 or periplasmic protein bp26 confer protection in mice challenged with *B. melitensis*. Vaccine. 2002. 30 (27): 4017–4022

[20] Yanga Y, Wangd L, Yinc J, Wanga X, Cheng S, Langa X, Wanga X, Qua H, Suna C, Wanga J, Zhang R. Immunoproteomic analysis of *Brucella melitensis* and identification of a new immunogenic candidate protein for the development of Brucellosis subunit vaccine. Molecular Immunology. 2011. 49: 175–184

Supplementary Table S2-C: Summary of putative marker proteins for specific detection of *B. mallei* and *B. pseudomallei*.

Score	Protein (accession no.)	Observed in crude lysate[a]	No. of times reported[b]	Localization (PSORT)[c]	Nearest homolog (% identity, Coverage, E-value)[d]	Remarks[e]	References
65	ABC transporter	2	3	EC (10)	*B. oklahomensis** (82, 100, 0.0) *B. thailandensis* (86, 99, 0.0)	I,IP,Vrl	5,6,7
61	Autotransporter, BpaA, BpaB, BpaC, BpaD, BpaE, BpaF	ND	5	EC (9.6)	*Burkholderia thailandensis** (94, 81, 2e-96) *Burkholderia cepacia* (59, 89, 4e-48)	I,Vrl	12, 13
60	Phasin-like protein,PhaP, PhaZ	ND	3	PP (9.83)	*Burkholderia oklahomensis** (99, 100, 1e-125) *Burkholderia thailandensis* (100, 98, 2e-124)	I	1,3,10
60	Burkholderia intracellular motility A, BimA	ND	4	OM (10)	*Burkholderia ubonensis** (70, 46, 3e-103) *Paraburkholderia caballeronis* (52, 37, 2e-47)	I	12,13,15,16
52	Outer membrane porin	10	3	OM (10)	*Burkholderia thailandensis** (99, 100, 0.0) *Burkholderiacepacia* (78, 100, 0.0)	I,OM	1,2,3
48	60 kDa chaperonin, GroEL	2	6	C (9.9)	*Burkholderia thailandensis** (99, 100, 0.0) *Paraburkholderia sp. SOS3* (97, 100, 0.0)	I,ID,Vrl,C	1,3,7-10
41	Phage major tail sheath protein	1	1	U (2)	*Burkholderia thailandensis** (99, 100, 0.0) *Burkholderia ubonensis* (84, 99 0.0)	C	1
36	Outer membrane protein A, OmpA	4	3	OM (9.3)	*Burkholderia thailandensis** (99, 100, 1e-161) *Burkholderia sp. TSV86* (94, 100, 7e-128)	I,OM	1,2,4
35	TonB-dependent copper receptor	3	1	OM (10)	*Burkholderia thailandensis* (93, 100, 0.0) *Burkholderia* sp. MSMB617WGS (90, 100, 0.0)	I,OM	3
33	Heat shock Hsp20	ND	5	C (8.9)	*Burkholderia oklahomensis* (87, 100, 1e-88)	-	1,3,6,9,10

25	Translocator protein, BipB, BipD	ND	4	EC (9.7)	*Burkholderia ubonensis*<br>(37, 94, 1e-93)	EC/I	9,10,17,20
		-	-	EC (9.45)	*Burkholderia thailandensis**<br>(99, 100, 0.0)<br>*Burkholderia sp. BDU8*<br>(85, 100, 0.0)	-	-
20	Peroxidase/catalase	ND	4	C (9.2)	*Burkholderia oklahomensis*<br>(90, 100, 0.0)	-	8,18,1,6
14	Type III secretion system (TTSS) effector protein, BopE, BsaP, BsaU, BsaE	ND	3	EC (9.4)	*Burkholderia thailandensis*<br>(96, 100, 0.0)	I	10,17, 20
13	Succinyl-CoA:3-ketoacid-coenzyme A transferase, ScoA/B	ND	4	C (9.9)	*Burkholderia oklahomensis*<br>(97, 100, 2e-168)	EC/C	1,9,3,
13	Elongation factor Tu, EF-Tu	1	3	C (9.9)	*Burkholderia thailandensis**<br>(100, 99, 0.0)<br>*Ralstonia solanacearum*<br>(97, 100, 0.0)	I,ID,Vrl,C	3,9,11
12	Peptidyl-prolyl cis-trans isomerase B, PpiB	ND	4	PP (9.7)	*Burkholderia thailandensis**<br>(99, 100, 3e-136)<br>*Burkholderia sp. BDU8*<br>(98, 100, 2e-134)	C/Vrl	9,1,6,21
11	ATP synthase	ND	4	C (9.9)	*Burkholderia sp. RPE64*<br>(97, 100, 0.0)	C/I	1,3,9

[a]Maximum number of times any peptide of the protein was observed in the present investigation using crude whole cell lysate of the organism using 1DE- or 2DE- MS/MS approach. ND = not detected in the MS/MS analysis of the present investigation.

[b]Number of times the protein has been described in the organism with an evidence of protein expression in open literature.

[c] **C =** cytoplasmic; **CM** = cytoplasmic membrane; **CW** = cell wall; **EC** = extracellular; **ML** = multiple localization; **U** = unknown.

[d] Asterisk indicates query species recurring after first non-species homolog. The second homolog is the closest after which the query species is not seen.

[e] **I =** immunogenic; **ID =** immunodominant in naturally infected clinical sera; **IP =**immunoprotective; **Vrl =** virulence associated; **S =** surface associated; **OM =** outer membrane; **CE** = cell envelope; **EC =** extracellular; **C =** cytoplasmic; **PP =** Periplasmic

References:

[1] Wongtrakoongate P, Mongkoldhumrongkul N, Chaijanc S, Kamchonwongpaisanc S, Tungpradabkul S. Comparative proteomic profiles and the potential markers between *Burkholderia pseudomallei* and *Burkholderia thailandensis*. Molecular and Cellular Probe. 2007. 21: 81–91.

[2] Schell MA, Zhao P, Wells L. Outer membrane proteome of *Burkholderia pseudomallei* and *Burkholderia mallei* from diverse growth conditions. Journal of Proteome Research. 2011. 10: 2417–2424.

[3] Harding SV, Tyson MS, Smither SJ, Atkins TP, Oyston PCF, Brown KA, Liu CY, Wait T, Titball RW. The identification of surface proteins of *Burkholderia pseudomallei*. Vaccine. 2007. 25: 2664–2672.

[4] Tiyawisutsri R, Holden MTG, Tumapa S, Rengpipat S, Clarke SR, Foster SJ, Nierman WC, Day NPJ and Peacock S. *Burkholderia* Hep Hag autotransporter (BuHA) proteins elicit a strong antibody response during experimental glanders but not human melioidosis. BMC Microbiology. 2007. 7:19.

[5] Chu KK, Tippayawat P, Walker NJ, Harding SV, Atkins HS, Maillere B, Bancroft GJ, Lertmemongkolchai G, Altmann DM. $CD4^+$ T cell immunity to the *Burkholderia pseudomallei* ABC transporter LolC in melioidosis. Eur J Immunol. 2011. 41(1): 107–115.

[6] Velapatin B, Limmathurotsakul B, Peacock SJ, Speert DP. 2012. Identification of differentially expressed proteins from *Burkholderia pseudomallei* isolated during primary and relapsing melioidosis. Microbes and Infection. 14. 335-340.

[7] Keasey SL, Schmid KE, Lee MS, Meegan J, TomasP, Minto M, Tikhonov AP, Schweitzer B, Ulrich RG. Extensive antibody cross-reactivity among infectious gram-negative bacteria revealed by proteome microarray analysis. Molecular & Cellular Proteomics. 2009. 8:924–935.

[8] Thongboonkerd V, Vanaporn M, Songtawee N, Kanlaya R, Sinchaikul P, Chen ST, Easton A, Chu K, Bancroft GJ, Korbsrisate S. Altered proteome in *Burkholderia pseudomallei* rpoE operon knockout mutant: Insights into mechanisms of rpoE operon in stress tolerance, survival and virulence. Journal of Proteome Research. 2007. 6: 1334-1341.

[9] Al-Maleki AR, Mariappan V, Vellasamy KM, Shankar EM, Tay ST, Vadivelu J. Enhanced intracellular survival and epithelial cell adherence abilities of *Burkholderia pseudomallei* morphotypes are dependent on differential expression of virulence-associated proteins during mid-logarithmic growth phase. Journal of Proteomics. 2014. 106: 205 – 220.

[10] Wongtrakoongate P, Roytrakul S, Yasothornsrikul S, Tungpradabkul S. 2011. A proteome reference map of the causative agent of melioidosis *Burkholderia pseudomallei*. Journal of Biomedicine and Biotechnology. 2011, Volume 2011, Article ID 530926, 5 pages doi:10.1155/2011/530926

[11] Nieves W, Heang J, Asakrah S, Bentrup KHZ, Roy CJ, Morici LA. Immunospecific responses to bacterial elongation factor Tu during Burkholderia infection and immunization. Plos one. 2010. 5(12). e14361.

[12] Adler NRL, Stevens JM, Stevens MP, Galyov EE. 2011. Autotransporters and their role in the virulence of *Burkholderia pseudomallei* and *Burkholderia mallei*. Frontiers in Microbiology. 2011. 2(151): doi: 10.3389/fmicb.2011.00151

[13] Adler NRL, Stevens MP, Dean RE, Saint RJ, Pankhania D, Prior JL, Atkins TP, Kessler B, Nithichanon A, Lertmemongkolchai G, Galyov EE. Systematic mutagenesis of genes encoding predicted autotransported proteins of *Burkholderia pseudomallei* identifies factors mediating virulence in mice, net intracellular replication and a novel protein conferring serum resistance. Plos One. 2015. 10(4): e0121271

[14] Lafontaine ER, Balder R, Michel F, Hogan RJ. Characterization of an autotransporter adhesion protein shared by *Burkholderia mallei* and *Burkholderia pseudomallei*. BMC Microbiology. 2014. 14:92

[15] Stevens JM, Ulrich RL, Taylor LA, Wood MW, DeShazer D, Stevens MP, Galyov EE. Actin-binding proteins from *Burkholderia mallei* and *Burkholderia thailandensis* can functionally compensate for the actin-based motility defect of a *Burkholderia pseudomallei* bimA mutant. Journal of Bacteriology.2005. 22(187): 7857–7862

[16] Kumar S, Malik P, Verma SK, Pal V, Gautam V, Mukhopadhyay C, Rai GP. 2011. Use of a recombinant *Burkholderia* intracellular motility a protein for immuno-diagnosis of glanders. Clinical and vaccine immunology. 2011. 9(18): 1456–1461

[17] Haque A, Chu K, Easton A, Stevens MP, Galyov EE, Atkins T, Titball R, Bancroft GJ. 2006. A live experimental vaccine against *Burkholderia pseudomallei* elicits $cd4^+$ t cell–mediated immunity, priming t cells specific for 2 type III secretion system proteins. The journal of infectious diseases.2006. 194:1241–8.

[18] Donald LJ, Krokhin OV, Duckworth HW, Wiseman B, Deemagarn T, Singh R, Switala J, Carpena X, Fita I, Loewen CP. 2003. Characterization of the catalase-peroxidase katg from *Burkholderia pseudomallei* by mass spectrometry. The Journal of Biological Chemistry. 2003. 37(278): 35687–35692

[19] Chantratitaa N, Tandhavanant S, Wikraiphat C, Trunckc LA, Rholl DA, Thanwisai A, Saiproma N, Limmathurotsakul D, Korbsrisatee S, Day NPJ, Schweizer HP, Peacock SJ. Proteomic analysis of colony morphology variants of *Burkholderia pseudomallei* defines a role for the arginine deiminase system in bacterial survival. Journal of Proteomics. 2011. 75. 1031–1042.

[20] Broek CWV, Chalmers KJ, Stevens MP, Stevens JM. Quantitative proteomic analysis of *Burkholderia pseudomallei* Bsa Type III secretion system effectors using hypersecreting mutants. Molecular & Cellular Proteomics. 2015. 14(4). 905–916

[21] Norville IH, Harmer NJ, Harding SV, Fischer G, Keith KE, Brown KA, Tyson MS, Titbal RW. 2011. A *Burkholderia pseudomallei* macrophage infectivity potentiator-like protein has rapamycin-inhibitable peptidylprolyl isomerase activity and pleiotropic effects on virulence. Infection and Immunity. 2011. 79: 4299–4307.

Supplementary Table S2-D: Summary of putative marker proteins for specific detection of *C. botulinum*.

Score	Protein (accession no.)	Observed in crude lysate[a]	No. of times reported[b]	Localization (PSORT)	Nearest homolog (% identity, Coverage, E-value)	Remarks[c]	References
100	Botulinum neurotoxin type G, BoNT/G	ND	3	EC (9.7)	-	I, Vrl,IP	1,3,4
100	Botulinum neurotoxin type B, BoNT/B	ND	3	EC (9.9)	-	I, Vrl,IP	1,3,4
100	Botulinum neurotoxin type A, BoNT/A	ND	2	EC (9.9)	-	I, Vrl,IP	3,4
100	Botulinum neurotoxin type C, BoNT/C	ND	2	EC (10)	-	I, Vrl,IP	3,4
100	Botulinum neurotoxin type D, BoNT/D	ND	2	EC (9.9)	-	I, Vrl,IP	3,4
100	Botulinum neurotoxin type E, BoNT/E	ND	2	EC (10)	-	I, Vrl,IP	3,4
100	Botulinum neurotoxin type F, BoNT/F	ND	2	EC (9.9)	-	I, Vrl,IP	3,4
85	Haemaglutinin component, HA70	ND	1	U (2.5)	*Clostridium argentinense** (69, 100, 0.0) *Clostridium sp. ND2* (27, 25, 4e-09)	I, Vrl	1
85	Haemaglutinin component, HA17	ND	1	U (2.5)	-	I, Vrl	1
86	Thermolysin metallopeptidase	1	-	EC (9.7)	*Clostridium sporogenes* (78, 100, 0.0) *Bacillus cereus* (34, 97, 9e-96)	-	-
78	Aminopeptidase 1	1	-	U (2.5)	*Clostridium sporogenes** (99, 100, 0.0) *Clostridium tetanomorphum* (47, 95, 3e-129)	-	-
77	E-cinnamoyl-CoA:R-phenyllactate CoA transferase large subunit	1	-	C (9.97)	*Clostridium sporogenes** (99, 99, 0.0) *Clostridioidesmangenotii* (48, 99, 1e-133)	-	-
67	Clostripain	1	-	U (3.3)	*Clostridium sporogenes** (88, 100, 0.0) *Spirochaetes bacterium** (48, 92, 4e-143)	-	-
62	Flagellin (FlaA)	1	-	C (7.5)	*Clostridium tetani** (79, 100, 2e-140) *Anoxybacillus amylolyticus* (68, 100, 1e-109)	-	-
62	Myosin-cross-reactive antigen	1	-	C (7.5)	*Anaerocolum najejuensis** (84, 100, 0.0) *Clostridium uliginosum*	-	-

					(53, 98, 0.0)		
53	Acetyl-CoA acetyltransferase	1	-	C (9.9)	*Clostridium sporogenes** (99, 98, 0.0) *Anaerococcus sp. HMSC065G05* (67, 98, 0.0)	-	-
52	Butyrate kinase	1	-	C (9.9)	*Clostridium sporogenes** (98, 100, 0.0) *Clostridium beijerinckii* (68, 99, 1e-177)	-	-
50	3-hydroxybutyryl-CoA dehydrogenase	1	-	C (7.5)	*Clostridium sporogenes** (99, 100, 0.0) *Clostridium carboxidivorans* (75, 99, 2e-152)	-	-
49	Glycine hydroxymethyltransferase	1	-	C (9.7)	*Clostridium sporogenes** (99, 100, 0.0) *Clostridium sp. K25* (76, 99, 0.0)	-	-
47	Glycosyl hydrolase	1	-	U (3.3)	*Clostridium sporogenes** (99, 100, 0.0) *Clostridium paraputrificum* (77, 98, 0.0)	-	-
44	Chitinase	1	-	EC (0.0)	*Clostridium sporogenes** (97, 100, 0.0) *Bacillus cereus* (71, 70, 0.0)	-	-
44	Serine hydroxymethyltransferase	1	-	C (9.9)	*Clostridium sporogenes** (95, 100, 0.0) *Clostridium pasteurianum* (76, 99, 0.0)	-	-
45	Molecular chaperone DnaK	1	-	C (9.9)	*Clostridium sporogenes** (97, 100, 0.0) *Clostridium cavendishii* (80, 100, 0.0)	-	-
41	C3 exoenzyme	1	1	EC (8.9)	*Brevibacterium ravenspurgense* (79, 59, 5.6)	I, Vrl	2
39	Phosphopyruvate hydratase	1	-	C (9.9)	*Clostridium sporogenes** (99, 100, 0.0) *Clostridium frigidicarnis* (86, 99, 0.0)	-	-
42	Molecular chaperone GroEL	1	-	C (9.97)	*Clostridium sp. L74** (95, 100, 0.0) *Clostridium sp. Ade.TY* (83, 100, 0.0)	-	-

29	Rubrerythrin	1	-	C (7.5)	*Clostridium sporogenes*<br>(96, 100, 3e-137)	-	-
26	Ornithine carbamoyltransferase	1	-	C (9.9)	*Clostridium sporogenes*<br>(99, 93, 0.0)	-	-
37	Thiamine biosynthesis protein ThiC	1	-	C (7.5)	*Clostridium sporogenes**<br>(97, 100, 0.0)<br>*Clostridium paradoxum*<br>(78, 100, 0.0)	-	-
33	Thiolase	1	-	C (9.9)	*Clostridium sporogenes**<br>(99, 100, 0.0)<br>*Clostridium beijerinckii*<br>(77, 99, 0.0)	-	-
29	Chlorohydrolase/aminohydrolase	1	-	C (7.5)	*Clostridium argentinense*<br>(91, 100, 0.0)	-	-
26	Peptidase T	1	-	C (9.9)	*Clostridium sp. L74*<br>(94, 100, 0.0)	-	-
21	Glyceraldehyde-3-phosphate dehydrogenase	1	-	C (9.7)	*Clostridium sporogenes*<br>(99, 100, 0.0)	-	-
16	Glycerol dehydrogenase	1	-	C (7.5)	Clostridium sporogenes*<br>(100, 99, 0.0)<br>Commensalibacter intestine<br>(62, 100, 3e-159)	-	-
16	ATP-dependent Clp protease proteolytic subunit	1	-	C (9.6)	*Clostridium sp. L748**<br>(99, 100, 8e-139)<br>*Clostridium pasteurianum*<br>(86, 100, 2e-120)	-	-
14	Subunit of pyruvate:flavodoxin oxidoreductase	1	-	C (7.5)	*Clostridium sporogenes*<br>(96, 100, 0.0)	-	-
13	Dehydrogenase, FMN-dependent	1	-	C (7.5)	*Clostridium sporogenes*<br>(97, 100, 0.0)	-	-
12	Triosephosphate isomerase	1	-	C (9.9)	*Clostridium perfringens*<br>(73, 100, 3e-132)<br>*Clostridium sporogenes*<br>(98, 100, 9e-176)	-	-

[a]Maximum number of times any peptide of the protein was observed in the present investigation using crude whole cell lysate of the organism using 1DE- or 2DE- MS/MS approach. ND = not detected in the MS/MS analysis of the present investigation.

[b]Number of times the protein has been described in the organism with an evidence of protein expression in open literature.

[c] **C =** cytoplasmic; **CM** = cytoplasmic membrane; **CW** = cell wall; **EC** = extracellular; **ML** = multiple localization; **U** = unknown.

[d] Asterisk indicates query species recurring after first non-species homolog. The second homolog is the closest after which the query species is not seen.

[e] **I =** immunogenic; **ID =** immunodominant in naturally infected clinical sera; **IP =**immunoprotective; **Vrl =** virulence associated; **S =** surface associated; **OM =** outer membrane; **CE** = cell envelope; **EC =** extracellular; **C =** cytoplasmic; **PP =** Periplasmic

References:

[1] Terilli RR, Moura H, Woolfitt AR, Rees J, Schieltz DM and Barr JR. 2011. A historical and proteomic analysis of botulinum neurotoxin type/G. BMC Microbiology. 2011. 11:232.

[2] Muetzelburg MV, Hofmann F, Just I, Pich A., 2009. Identification of biomarkers indicating cellular changes after treatment of neuronal cells with the C3 exoenzyme from *Clostridium botulinum* using the iTRAQ protocol and LC–MS/MS analysis. Journal of Chromatography B. 2009. 877: 1344–1351.

[3] Hines HB, Lebeda F, Hale M, Brueggemann EE. Characterization of botulinum progenitor toxins by mass spectrometry. Appl. Environ. Microbiol. 2005. 71(8): 4478–4486.

[4] Barr JR, Moura H, Boyer AE, Woolfitt AR, Kalb SR, Pavlopoulos A, McWilliams L, Schmidt JG, Martinez RA, Ashley DL. Botulinum neurotoxin detection and differentiation by mass spectrometry. Emerg. Infect. Dis. 2005. 11:1578–1583.

Supplementary Table S2-E: Summary of putative marker proteins for specific detection of *C. perfringens*.

Score	Protein (accession no.)	Observed in crude lysate[a]	No. of times reported[b]	Localization (PSORT)[c]	Homologs in (% identity; E-value)[d]	Remarks[e]	References
100	Electron transfer flavoprotein	3	1	U (2.5)	*Clostridium sp. Ade.TY* (80, 100, 4e-166)	Vrl	2
68	DipeptidasePepV	14	1	C (7.5)	*Clostridium tepidiprofundi* (66, 99, 0.0)	Vrl	2
67	Rubrerythrin	15	1	C (7.5)	*Clostridium baratii* (78, 100, 9e-110)	Vrl	2
65	Fructose-bisphosphate aldolase	12	4	C (7.5)	*Clostridium ventriculi* (85, 100, 1e-180)	EC/I/ Vrl	1,2,3,4
60	Glutaredoxin	5	1	U (2.5)	*Clostridium sp. Marseille* (70, 100, 2e-31)	Vrl	2
57	Hypothetical protein CPE0268	4	1	C (8.7)	*Clostridium ventriculi* (63, 99, 7e-106)	Vrl	2
54	Maltose ABC transporter	2	1	CM (10)	*Clostridium sp. Ade.TY* (76, 98, 4e-176)	Vrl	3
53	Heme oxygenase	2	1	C (7.5)	*Clostridium sp. Ade.TY* (67, 97, 2e-99)	Vrl	2
53	Metallopeptidase, family M24	2	2	C (9.9)	*Clostridium ventriculi* (72, 99, 0.0)	EC/Vrl	2,3
52	DnaK-type molecular chaperone hsp70	17	2	C (7.5)	*Clostridium ventriculi* (88, 100, 0.0)	EC/I/ Vrl	2,3
50	Peptidyl-prolyl cis-trans isomerase	1	2	C (9.9)	*Clostridium fallax* (75, 100, 3e-89)	EC/Vrl	2,3
49	Choloylglycine hydrolase family protein	14	1	U (2.5)	*Paeniclostridium sordellii** (86, 100, 0.0)	Vrl	2
49	Glucose-6-phosphate isomerase	13	1	C (9.9)	*Clostridium cavendishii* (86, 99, 0.0)	Vrl	2
44	Triosephosphate isomerase	9	2	C (9.9)	*Clostridium fallax* (86, 100, 7e-160)	EC/Vrl	2,3
33	Elongation factor Tu	6	1	C (10)	*Clostridium amylolyticum** (92, 100, 0.0)	Vrl	2
36	Hypothetical protein CPE0689	9	1	C (7.5)	*Clostridium sp. ND2* (89, 100, 3e-117)	Vrl	2
39	Ornithine carbamoyl transferase	5	2	C (10)	*Clostridium sp. Ade.TY* (96, 100, 0.0)	EC/I/ Vrl	2,3
34	Clostripain-like protease (Clp)	ND	4	C (9.9)	*Clostridium sp. ND2* (91, 99, 1e-128)	Vrl	2,3,5
49	Thioredoxin	1	1	C (9.6)	*Clostridium sp. ND2* (71, 100, 1e-47)	Vrl	2
45	ABC transporter	3	1	CM (10)	*Clostridium ventriculi*	EC	2

					(85, 100, 1e-159)		
45	Purine nucleoside phosphorylase	1	2	C (7.5)	*Clostridium ventriculi* (80, 100, 3e-164)	EC/Vrl	2,3
43	Deoxyribose-phosphate aldolase	3	1	C (9.9)	*Clostridium sp. Marseille-P2434* (87, 100, 2e-136)	Vrl	2
42	Putative endoribonuclease L-PSP	2	1	C (9.9)	*Clostridium intestinale* (78, 99, 7e-66)	Vrl	2
40	Phosphopyruvate hydratase	2	1	C (9.9)	*Clostridium sp. CL-2* (90, 100, 0.0)	Vrl	2
35	Pyruvate-flavodoxin oxidoreductase	4	1	C (7.5)	*Clostridium cavendishii* (85, 99, 0.0)	Vrl	2
34	Acetaldehyde-CoA/alcohol dehydrogenase	4	1	C (9.9)	*Clostridium sp. Marseille-P2434* (86, 100, 0.0)	Vrl	2
32	Putative PTS system	2	1	C (10)	*Clostridium baratii* (88, 99, 0.0)	Vrl	2
31	Hypothetical protein CPF_1179	1	1	C (7.5)	*Clostridium sp. NCR* (89, 98, 4e-77)	Vrl	2
29	Glyceraldehyde-3-phosphate dehydrogenase (GAPDH)	ND	3	C (9.9)	*Clostridium sp. ND2* (91, 99, 0.0)	Vrl/I/EC	1,2,3
29	Elongation factor G [EF-G]	ND	3	C (9.97)	*Clostridium sp. ND2* (86, 99, 0.0)	Vrl	1,2,3
25	30S ribosomal protein S8	2	1	C (9.6)	*Clostridium sp. ND2* (95, 100, 9e-86)	Vrl	2

[a]Maximum number of times any peptide of the protein was observed in the present investigation using crude whole cell lysate of the organism using 1DE- or 2DE- MS/MS approach. ND = not detected in the MS/MS analysis of the present investigation.

[b]Number of times the protein has been described in the organism with an evidence of protein expression in open literature.

[c] **C =** cytoplasmic; **CM** = cytoplasmic membrane; **CW** = cell wall; **EC** = extracellular; **ML** = multiple localization; **U** = unknown.

[d] Asterisk indicates query species recurring after first non-species homolog. The second homolog is the closest after which the query species is not seen.

[e] **I =** immunogenic; **ID =** immunodominant in naturally infected clinical sera; **IP =**immunoprotective; **Vrl =** virulence associated; **S =** surface associated; **OM =** outer membrane; **CE** = cell envelope; **EC =** extracellular; **C =** cytoplasmic; **PP =** Periplasmic

References:

[1] Kulkarni RR, Parreira VR, Sharif S, and Prescott JF. *Clostridium perfringens* antigens recognized by broiler chicken's immune to necrotic enteritis. Clinical and Vaccine Immunology. 2006. 13 (12): 1358–1362.

[2] Dwivedi P, Alam SI, Kumar O, Kumar RB. Comparative analysis of extractable proteins from *Clostridium perfringens* type A and type C strains showing varying degree of virulence. 2015. Anaerobe. 35:77-91.

[3] Sengupta N, Alam SI, Kumar B, Kumar RB, Gautam V, Kumar S and Singh L. 2010. Comparative proteomic analysis of extracellular proteins of *Clostridium perfringens* Type A and Type C Strains. Infection and Immunity. 2010. 78(9): 3957–3968

[4] Shimizu T, Shima K, Yoshino K, Yonezawa K, Shimizu K and Hayashi H. Proteome and T Transcriptome Analysis of the Virulence Genes Regulated by the VirR/VirS System in *Clostridium perfringens*. Journal of Bacteriology. 2002. 184(10): 2587–2594.

[5] Manabe S, Nariya H, Miyata S, Tanaka H, Minami H, Suzuki M, Taniguchi Y and Okabe A. 2010. Purification and characterization of a clostripain-like protease from a recombinant *Clostridium perfringens* culture. 156, 561–569

Supplementary Table S2-F: Summary of putative marker proteins for specific detection of *C. tetani*.

S. No.	Protein (accession no.)	Observed in crude lysate[a]	No. of times reported[b]	Localization (PSORT)[c]	Nearest homolog (% identity, Coverage, E-value)[d]	Remarks[e]	References
84	Tetanus neurotoxin, TeNT	1	-	EC (9.7)	*Clostridium botulinum* (41, 99, 0.0)	-	-
84	Putative S-layer protein	1	-	CW (10)	*Clostridium novyi* (41, 92, 2e-121)	-	-
40	Electron transfer flavoprotein	1	1	C (7.5)	*Clostridium cochlearium** (95, 100, 0.0)	-	1
39	Acetyl-CoA acetyltransferase	1	-	C (9.9)	*Clostridium novyi* (86, 100,0.0)	-	-
36	Molecular chaperone DnaK	1	1	C (9.9)	*Clostridium cochlearium* (94, 100, 0.0)	-	1
34	60 kDa chaperonin GroEL	1	1	C (9.9)	*Clostridium cochlearium* (96, 99, 0.0)	-	1
34	Butyrate kinase	1	1	C (9.9)	*Clostridium cochlearium* (96, 100, 0.0)	-	1
32	Adenosyltransferase	1	-	C (7.5)	*Clostridium sp. KNHs214* (73, 100, 1e-112)	-	-
30	Methylaspartate ammonia lyase	1	1	C (7.5)	*Clostridium cochlearium* (96, 100, 0.0)	-	1
29	Elongation factor Tu	1	-	C (10)	*Clostridium cochlearium* (96, 100, 0.0)	-	-
29	Methionine gamma lyase	1	-	C (7.5)	*Clostridium sp. HMP27* (76, 98, 0.0)	-	-
28	Pyruvate-flavodoxin oxidoreductase	1	-	U (2.5)	*Clostridium cochlearium* (92, 100. 0.0)	-	-
25	3-hydroxybutyryl-CoA dehydratase	1	-	C (7.5)	*Anaerosalibacter massiliensis* (85, 100, 2e-160)	-	-
19	Rubrerythrin	1	-	C (7.5)	*Clostridium cochlearium* (96, 100, 2e-133)	-	-
19	3-hydroxybutyryl-CoA dehydrogenase	1	-	C (7.5)	*Clostridium cochlearium* (96, 100, 0.0)	-	-
14	Phosphopyruvate hydratase	1	-	C (7.5)	*Clostridium cochlearium* (91, 100, 0.0)	-	-
13	Cobyric acid synthase	1	-	C (7.5)	*Clostridium cochlearium* (92, 99, 0.0)	-	-
12	Aspartate-semialdehyde dehydrogenase	1	-	C (9.6)	*Clostridium cochlearium* (93, 99, 0.0)	-	-
12	Glyceraldehyde 3-phosphate dehydrogenase	1		C (9.9)	*Clostridium cochlearium* (93, 100, 0.0)	-	-

11	Fumarate hydratase	1	-	C (7.5)	*Clostridium cochlearium* (94, 100, 0.0)	-	-
11	Adenylate kinase	1	-	C (9.9)	*Clostridium cochlearium* (94, 100, 3e-147)	-	-
9	Phosphate butyryltransferase	1	-	C (7.5)	*Clostridium cochlearium* (96, 100, 0.0)	-	-
9	Ribose-phosphate pyrophosphokinase	1	-	C (9.9)	*Clostridium cochlearium* (96, 99, 0.0)	-	-
8	Thiolase	1	-	C (7.5)	*Clostridium cochlearium* (97, 100, 0.0)	-	-

[a]Maximum number of times any peptide of the protein was observed in the present investigation using crude whole cell lysate of the organism using 1DE- or 2DE- MS/MS approach. ND = not detected in the MS/MS analysis of the present investigation.

[b]Number of times the protein has been described in the organism with an evidence of protein expression in open literature.

[c] **C =** cytoplasmic; **CM** = cytoplasmic membrane; **CW** = cell wall; **EC** = extracellular; **ML** = multiple localization; **U** = unknown.

[d] Asterisk indicates query species recurring after first non-species homolog. The second homolog is the closest after which the query species is not seen.

[e] **I =** immunogenic; **ID =** immunodominant in naturally infected clinical sera; **IP =**immunoprotective; **Vrl =** virulence associated; **S =** surface associated; **OM =** outer membrane; **CE** = cell envelope; **EC =** extracellular; **C =** cytoplasmic; **PP =** Periplasmic

Reference

1. Alam SI, Bansod S, Singh L. Immunization against *Clostridium perfringens* cells elicits protection against *Clostridium tetani* in mouse model: identification of cross-reactive proteins using proteomic methodologies. BMC Microbiology 2008, 8:194.

Supplementary Table S3: Summary of selected marker proteins for specific detection of target bacterial agents of BW significance.

Supplementary Table S3: Summary of selected marker proteins for specific detection of target bacterial agents of BW significance.

S. No	Protein (accession no.)	Molecular function / biological process	Localization (PSORT)[a]	Remarks[b]
Bacillus anthracis				
1	S-layer protein EA1 (P94217)	Structural molecule activity; External encapsulating structure organization	CW (10)	I,CE,EC
2	Protective antigen, PA (P13423)	Metal ion binding; Toxin activity; Negative regulation of gene expression, MAPK cascade, protein phosphorylation	EC (9.9)	I/EC/Vrl
3	Polysaccharide deacetylase, YxkH, YjeA (P59744)	Hydrolase activity, acting on carbon-nitrogen (but not peptide) bonds, in linear amides; Polysaccharide catabolic process	M (3.3)	C/I/W
4	Lethal factor (P15917)	Metal ion binding; Metallo-peptidase activity; Toxin activity; Negative regulation of gene expression, Pathogenesis	EC (9.9)	EC/Vrl/I
5	ABC transporter, substrate binding protein (Q81J16)	ATPase activity; ATP binding; Transmembrane transport	C (7.5)	I/CM
6	Sulfatase, YvgJ, YflE (Q732W3)	Metal ion; Sulfuric ester hydrolase activity; Metabolic process	CM (9.3)	I
7	ATP-binding subunit of ABC transporter	ATPase activity; ATP binding; Transmembrane transport	CM (9.9)	I/SP/EC
8	60 kDa molecular chaperone GroEL (Q814B0)	ATP binding; Protein folding, unfolding and re-folding; 'de novo' protein folding	C (9.9)	I,Vrl,SP,EC
9	Manganese superoxide dismutase, SodA-2 (Q81JK8)	Metal ion binding; Superoxide dismutase activity; Removal of superoxide radicals	EC (9.7)	I/SP/EC
10	Single-stranded DNA-binding protein (Q814G6)	Single-stranded DNA binding; DNA recombination, repair and replication	C (9.6)	I
11	Universal stress protein (Q817D1)	Response to environmental stress; Cytoplasmic protein	C (7.5)	I
12	Alcohol dehydrogenase (Q818A4)	Acetaldehyde dehydrogenase activity; Alcohol dehydrogenase (NAD) activity; Metal ion binding	C (9.9)	SP
13	Elongation factor Tu, EF-Tu (C3P9Q3)	GTPase activity; Translation elongation factor activity; Protein biosynthesis	C (9.9)	I,SP
14	Histidinol-phosphate aminotransferase (Q81C43)	Pyridoxal phosphate binding; Histidine biosynthetic process	C (7.5)	SP

15	Enolase, Eno (Q815K8)	Magnesium ion binding; Phosphopyruvate hydratase activity; Glycolytic process	C (10)	I/SP/C
16.	Formate acetyltransferase (A0A0F7RMN6)	Activation of pyruvate formate-lyase under anaerobic conditions by generation of an organic free radical	C (9.9)	I/C
17.	Alanine dehydrogenase (A0A2B0VZ84)	Involved in step 1 of the sub-pathway that synthesizes NH(3) and pyruvate from L-alanine.	C (9.9)	I/C
18.	Pyruvate dehydrogenase (NP_846420)	Catalyzes the conversion of lactate to pyruvate	C (7.5)	I/C
19.	Superoxide dismutase (NP_846724)	Destroys superoxide anion radicals produced within the cells and which are toxic to biological systems.	EC (9.7)	I/C
Brucella melitensis, Brucella suis, Brucella abortus				
1	31-kDa outer-membrane immunogenic protein (P0A3U4)	Porin activity; Ion transport	OM (10)	OM,IP,CE,I
2	DNA starvation/stationary phase protection protein, Dps (A0A0H3G988)	Ferric iron binding; Oxidoreductase activity; Cellular iron ion homeostasis	C (9.9)	I,ID,C
3	60 kDa Chaperonin, GroEL (Q8YB53)	ATP binding; Unfolded protein binding; Protein refolding	C (9.9)	I,ID,Vrl,C,CE
4	Molecular chaperone DnaK (Q8YE76)	ATP binding; Unfolded protein binding; Protein folding	C (9.9)	I,ID,Vrl,OM
5	Elongation factor Tu, EF-Tu (P64024)	GTPase activity; Translation elongation factor activity; Protein biosynthesis	C (10)	I,ID,Vrl,C
6	Ribose-binding protein (Q73DH5)	Ribose ABC transporter; High affinity active transport system across the cytoplasmic membrane	PP (9.7)	I
7	Glyceraldehyde-3-phosphate dehydrogenase (Q8YIX9)	NAD and NADP binding; Oxidoreductase activity, acting on the aldehyde or oxo group of donors; Glucose metabolic process	C (9.9)	I,ID,Vrl,C
8	ATP-dependent clp protease (Q8YHC7)	ATP binding; Protein dimerization activity; Unfolded protein binding; Zinc ion binding	C (9.9)	Vrl,C
9	Fe/Mn family superoxide dismutase (Q81LW0)	Metal ion binding; Superoxide dismutase activity; Removal of superoxide radicals	EC (9.6)	I,ID,Vrl,PP
10	Amino acid ABC transporter substrate-binding protein (A0A1Z1ZIT7)	Amino acid transport	PP (9.4)	I
11	Immunogenic 39-kDa protein (O06875)	Carbohydrate transport; Probable sugar binding periplasmic protein	U (2.5)	I,Vrl,PP

12	Bacterioferritin (P49944)	Ferric iron binding and transport; Ferroxidase activity; Cellular iron ion homeostasis	C (9.9)	Vrl,C
13	Aminotransferase (Q8YHS2)	Pyridoxal phosphate binding; Transaminase activity; Biosynthetic process	C (7.5)	C
14	Ribosomal protein S1 (Q8YEG3)	RNA binding; Structural constituent of ribosome; Translation	C (9.9)	C/I
15	Isovaleryl-CoA dehydrogenase (Q8YEF5)	Acyl-CoA dehydrogenase activity; Flavin adenine dinucleotide binding	C (9.9)	C/I
16	Invasion protein B (Q8YFD8)	Major virulence factor; Vector-to-host transmission; Environmental sress associated protein	U	-
17.	Hypothetical protein EIA50_02275 (AZS90677)	Unknown function	U	I/C
18.	Outer membrane protein OprF (A0A0D4D8X8)	Type IV secretion system putative outer membrane lipoprotein	U	I/SP
19.	Outer membrane protein 28 (P0A3U8)	28 KDa periplasmic immunogenic protein	PP (10.0)	I/SP
20.	31 kDa outer membrane protein (P0A3U4)	Major outer membrane protein associated with peptidoglycans. May function as a porin	OM (10.0)	I/SP
Burkholderia mallei / Burkholderia pseudomallei				
1	ABC transporter (Q63XJ5)	ATPase activity; ATP binding; Phosphate transport	EC (10)	I,IP,Vrl
2	Autotransporter, BpaA, BpaB, BpaC, BpaD, BpaE, BpaF (B1N8M4)	Pathogenesis; Pathogen-host adhesion	EC (9.6), OM (10), U (2)	I,Vrl
3	Phasin-like protein,PhaP, PhaZ (Q63SM2)	Surface proteins found covering polyhydroxyalkanoate (PHA) storage granules in bacteria; Involve in stress resistance	-	I
4	Burkholderia intracellular motility A, BimA (A2RXV6)	Pathogenesis; Cell adhesion	OM (10)	I
5	Outer membrane porin (A0A0H2WDH7)	Porin activity**;** Ion transport	OM (10)	I,OM
6	60 kDa chaperonin, GroEL (Q9F712)	ATP binding**;** Protein refolding	C (9.9)	I,ID,Vrl,C
7	Phage major tail sheath protein (Q63YN7)	Subtilisin-like structure, Protein of un-known function	U (2)	C
8	Outer membrane protein A, OmpA (A0A0H2WHA7)	Structural molecule activity; OmpA-like domain forms non-covalent interactions with peptidoglycan	OM (9.3)	I,OM

9	TonB-dependent copper receptor (Q2T7L2)	Receptor activity; Transport	OM (10)	I,OM
10	Heat shock Hsp20 (Q2SUS8)	Molecular chaperon involved in heat induced stress	C (8.9)	
11	Translocator protein, BipB(Q62B07)	Pathogenesis; Type III secretion system translocator	EC (9.7)	EC/I
	BipD (A3MCH1)	Pathogenesis; Type III secretion system translocator	EC (9.45)	-
12	Peroxidase/catalase (Q62H74)	Catalase activity; Heme and metal ion binding; Hydrogen peroxide catabolic process; Response to oxidative stress	C (9.2)	-
13	Type III secretion system (TTSS) effector protein, BopE, BsaP, BsaU, BsaE (A2S1Q9)	Pathogenesis; Binding	EC (9.4)	I
14	Succinyl-CoA:3-ketoacid-coenzyme A transferase, ScoA/B (A0A290Y8N3)	CoA-transferase activity; Metabolic process	C (9.9)	EC/C
15	Elongation factor Tu, EF-Tu (Q62GK3)	GTPase activity; GTP binding; Translation elongation factor activity; Protein biosynthesis	C (9.9)	I,ID,Vrl,C
16	Peptidyl-prolyl cis-trans isomerase B, PpiB (A0A0H2WER4)	Peptidyl-prolyl cis-trans isomerase activity; Protein folding	PP (9.7)	C/Vrl
Clostridium botulinum				
1	Botulinum neurotoxin type G, BoNT/G (Q60393)	Metalloendopeptidase activity; Protein transmembrane transporter activity; Toxin activity; Inhibition of neurotransmitter uptake	EC (9.7)	I, Vrl,IP
2	Botulinum neurotoxin type B, BoNT/B (P10844)	-do-	EC (9.9)	I, Vrl,IP
3	Botulinum neurotoxin type A, BoNT/A (A5HZZ9)	-do-	EC (9.9)	I, Vrl,IP
4	Botulinum neurotoxin type C, BoNT/C (P18640)	-do-	EC (10)	I, Vrl,IP
5	Botulinum neurotoxin type D, BoNT/D (P19321)	-do-	EC (9.9)	I, Vrl,IP
6	Botulinum neurotoxin type E, BoNT/E (Q00496)	-do-	EC (10)	I, Vrl,IP
7	Botulinum neurotoxin type F, BoNT/F (Q00496)	-do-	EC (9.9)	I, Vrl,IP
8	Haemaglutinin component, HA70 (Q9LBR5)	Pathogenesis	U (2.5)	I, Vrl
9	Haemaglutinin component, HA17 (P46083)	Pathogenesis	U (2.5)	I, Vrl
10	Thermolysin metallopeptidase (B1IKQ0)	Metalloendopeptidase activity; Protease inhibitor	EC (9.7)	–

11	Aminopeptidase 1 (A5HYY2)	Metallopeptidase activity; Tripeptide aminopeptidase activity; Zinc ion binding; Peptide metabolic process	U (2.5)	–
12	E-cinnamoyl-CoA:R-phenyllactate CoA transferase (A5I717)	Formyl-CoA transferase activity; Metabolic process	C (9.97)	–
13	Clostripain (A5I344)	Hydrolase activity; Cysteine peptidases	U (3.3)	–
14	Flagellin (FlaA) (C1FU93)	Structural molecule activity; Bacterial-type flagellum-dependent cell motility	C (7.5)	–
15	Myosin-cross-reactive antigen (A7GF03)	FAD binding; Oleate hydratase activity; Fatty acid metabolic process	C (7.5)	–
16	Acetyl-CoA acetyltransferase (A5I6S9)	Acetyl-CoA C-acetyltransferase activity; Aetyl-CoA C-acyltransferase activity; Fatty acid beta-oxidation	C (9.9)	–
17	Butyrate kinase (B2TIN4)	ATP binding; Butyrate kinase activity; Phosphorylation; Metabolic process	C (9.9)	–
18	3-hydroxybutyryl-CoA dehydrogenase (A5I6T0)	3-hydroxyacyl-CoA dehydrogenase activity; Fatty acid metabolic process	C (7.5)	–
19	Glycosyl hydrolase (A7GDF9)	Catalytic activity; Nucleoside metabolic process	U (3.3)	–
20	Chitinase (A5I0Q0)	Carbohydrate binding and metabolism; Chitinase activity	EC (0.0)	–
21	Serine hydroxymethyltransferase (A5I526)	Glycine hydroxymethyltransferase activity; Pyridoxal phosphate binding; Glycine biosynthetic process from serine	C (9.9)	–
22	Molecular chaperone DnaK (A5I640)	ATPase activity; Protein folding; Cellular hyperosmotic salinity, acidic and heat response	C (9.9)	–
23	C3 exoenzyme (U5NYK2)	Pentosyl group ransferase activity, Pathogenesis; Potein ADP-ribosylation	EC (8.9)	I, Vrl
24	Phosphopyruvate hydratase (A7FQP0)	Magnesium ion binding; Phosphopyruvate hydratase activity; Glycolytic process	C (9.9)	–
25	Molecular chaperone GroEL (A5I723)	ATP binding; Protein folding and refolding; ‘de novo’ protein folding	C (9.97)	–
26	Rubrerythrin (A7GD24)	Iron ion binding; Oxidoreductase activity	C (7.5)	–
27	Ornithine carbamoyltransferase (A5I524)	Amino acid binding; Ornithine carbamoyltransferase activity; Arginine catabolic and biosynthetic process; Urea cycle	C (9.9)	–
Clostridium perfringens				

1	Electron transfer flavoprotein (Q8XNM2)	Electron transfer activity; Oxidation of fatty acids	U (2.5)	Vrl
2	DipeptidasePepV (A0A174AVU5)	Dipeptidase activity; Metallopeptidase activity; Zinc ion binding; Hydrolase activity; Metabolic process	C (7.5)	Vrl
3	Rubrerythrin (A0A174HAA1)	Iron ion binding; NADH peroxidase activity	C (7.5)	Vrl
4	Fructose-bisphosphate aldolase (A0A0H2YU97)	Aldehyde-lyase activity; Zinc ion binding; Carbohydrate metabolic process	C (7.5)	EC/I/ Vrl
5	Glutaredoxin (A0A140GTL6)	Electron transfer activity; Protein disulfide oxidoreductase activity; Cell redox homeostasis	U (2.5)	Vrl
6	Hypothetical protein (CPE0268)	Post-translocation chaperonin	C (8.7)	Vrl
7	Maltose ABC transporter (A0A0H2YSK4)	ATPase activit; Transmembrane transporter activity	CM (10)	Vrl
8	Heme oxygenase (B1R8B0)	Heme oxygenase (decyclizing) activity; Metal ion binding	C (7.5)	Vrl
9	Metallopeptidase, family M24 (B1BUS8)	ATPase activity; Metallo-endopeptidase activity; Zinc ion binding; Protein catabolic process	C (9.9)	EC/Vrl
10	DnaK-type molecular chaperone hsp70 (P26823)	ATP binding; Protein folding	C (7.5)	EC/I/ Vrl
11	Peptidyl-prolyl cis-trans isomerase (A0A173Z966)	Peptidyl-prolyl cis-trans isomerase activity; Protein folding	C (9.9)	EC/Vrl
12	Choloylglycine hydrolase family protein (P54965)	Choloylglycine hydrolase activity	U (2.5)	Vrl
13	Glucose-6-phosphate isomerase (Q0TN51)	Glucose-6-phosphate isomerase activity; Gluconeogenesis; Glycolytic process	C (9.9)	Vrl
14	Triosephosphate isomerase (Q0TQY8)	Triose-phosphate isomerase activity; Gluconeogenesis; Glycolytic process	C (9.9)	EC/Vrl
15	Elongation factor Tu (B1BY79)	GTPase activity; Translation elongation factor activity; Protein biosynthesis	C (10)	Vrl
16	Hypothetical protein CPE0689	Iron binding, Protein of unknown function	C (7.5)	Vrl
17	Ornithine carbamoyl transferase (Q0TUR4)	Amino acid binding; Ornithine carbamoyltransferase activity; Arginine catabolic process to ornithine	C (10)	EC/I/ Vrl
18	Clostripain-like protease (Clp) (A0A0H2YP53)	Hydrolase activity**;** Cystein peptidase with hydrolysis activity	C (9.9)	Vrl

19	Thioredoxin (B1BMM8)	Protein disulfide oxidoreductase activity; Cell redox homeostasis; Glycerol ether metabolic process	C (9.6)	Vrl
20	ABC transporter (Q8XNI5)	ATPase activity; DNA binding; Metal ion binding; Transmembrane transport activity	CM (10)	EC
21	Purine nucleoside phosphorylase (Q0TQJ7)	Purine-nucleoside phosphorylase activity; Nucleoside metabolic process	C (7.5)	EC/Vrl
Clostridium tetani				
1	Tetanus neurotoxin, TeNT (P04958)	Metalloendopeptidase activity; Protein transmembrane transporter; Toxin activity; Negative regulation of neurotransmitter secretion	EC (9.7)	–
2	Putative S-layer protein (Q898I0)	Collagen binding; Cell adhesion	CW (10)	–
3	Electron transfer flavoprotein (Q896M7)	Electron transfer activity; Flavin adenine dinucleotide binding; Fatty acid oxidation	C (7.5)	–
4	Acetyl-CoA acetyltransferase (U6EVZ1)	Transferring acyl groups other than amino-acyl groups; Metabolic process	C (9.9)	–
5	Molecular chaperone DnaK (Q892R0)	ATP binding; Unfolded protein binding; Protein folding	C (9.9)	–
6	60 kDa chaperonin GroEL (Q891G4)	ATP binding; Protein refolding	C (9.9)	–
7	Butyrate kinase (Q890U0)	ATP binding; Butyrate kinase activity; Butyrate metabolic process	C (9.9)	–
8	Adenosyltransferase (Q898W7)	ATP binding; Metal ion binding; Methionine adenosyltransferase activity; S-adenosylmethionine biosynthetic process	C (7.5)	–
9	Methylaspartate ammonia lyase (Q890S3)	Metal ion binding; Methylaspartate ammonia-lyase activity; Glutamate fermentation	C (7.5)	–
10	Elongation factor Tu (U6EZ59)	GTPase activity; Translation elongation factor activity; Protein biosynthesis	C (10)	–
11	Methionine gamma lyase (Q890V5)	Methionine gamma-lyase activity; Pyridoxal phosphate binding; Cysteine and methionine metabolism	C (7.5)	–
12	Pyruvate-flavodoxin oxidoreductase (Q893R9)	Iron binding; Oxidoreductase activity, Thiamine pyrophosphate binding; Electron transport chain	U (2.5)	–
13	3-hydroxybutyryl-CoA dehydratase (Q898H3)	3-hydroxybutyryl-CoA dehydratase activity; Metabolic process	C (7.5)	–
14	Rubrerythrin (Q895T5)	Iron ion binding; Oxidoreductase activity	C (7.5)	–

15	3-hydroxybutyryl-CoA dehydrogenase (Q891F6)	3-hydroxyacyl-CoA dehydrogenase activity; NAD^+ binding	C (7.5)	–

[a] **C =** cytoplasmic; **CM** = cytoplasmic membrane; **CW** = cell wall; **EC** = extracellular; **ML** = multiple localization; **U** = unknown.
[b] **I =** immunogenic; **ID =** immunodominant in naturally infected clinical sera; **IP =**immunoprotective; **Vrl =** virulence associated; **S =** surface associated; **OM =** outer membrane; **CE** = cell envelope; **EC =** extracellular; **C =** cytoplasmic; **PP =** Periplasmic

Supplementary Table S4 A to I: Inclusion list of unique and abundant peptides from selected protein markers of selected species.

Supplementary Table S4-A: Inclusion list of unique and abundant peptides from selected protein markers of *Bacillus anthracis*.

S. No.	Protein	Peptide	Strain coverage	Theoretical mass (Da)
1	S-layer protein EA1	SFPDVPAGHWAEGSINYLVDK*	134/139	2302.03
	(>NP_843398.1)	ADLYDTLTTK	133/139	1140.54
		GAITGKPDGTYGPTESIDR	134/139	1934.88
		TLPVTFVTTDQYGDPFGANTAAIK*	137/139	2527.21
		VGQYGQSPDTK*	135/139	1179.53
		DNAQAYVTDVK	139/139	1223.55
		GDGKENFYPEGK*	134/139	1340.56
		ATVEIVQETIAIK**	135/139	1414.76
		FVANNLDGSPANIFEGGEATSTTGK	137/139	2497.12
2	Protective antigen, PA	HPLVAAYPIVHVDMENIILSK[BA]	132/132	2359.28
	(>AJH43206.1)	ENTIINPSENGDTSTNGIK[BA*]	132/132	2003.95
		LDTDQVYGNIATYNFENGR[BA*]	132/132	2190.00
		ILSGYIVEIEDTEGLK[BA**]	132/132	1778.94
		YDMLNISSLQQDGK[BA]	1/132	1611.76
		LPLYISNPNYK[BA**]	132/132	1321.71
3	Lethal factor	EHPTDFSVEFLEQNSNEVQEVFAK[BA*]	116/116	2823.31
	(>AJH43062.1)	NDSEGFIHEFGHAVDDYAGYLLDK[BA*]	116/116	2712.22
		YSISSNYMIVDINERPALDNER[BA*]	116/116	2599.24
		EGYEPVLVIQSSEDYVENTEK [BA*]	116/116	2428.14
		DIQNIDALLHQSIGSTLYNK[BA*]	116/116	2243.16
		YASNIVESAYLILNEWK[BA*]	116/116	2013.03
		DVLQLYAPEAFNYMDK[BA*]	116/116	1916.90
		LQDTGGLIDSPSINLDVR[BA*]	116/116	1912.99
		NASDSDGQDLLFTNQLK[BA*]	116/116	1865.88
		QHYQHWSDSLSEEGR[BA*]	116/116	1858.81
		IQEAQLNINQEWNK[BA*]	116/116	1727.87
		FNEQEINLSLEELK[BA*]	116/116	1705.86
		IQIDSSDFLSTEEK[BA*]	116/116	1611.77
		DALLHEHYVYAK[BA*]	116/116	1458.73
		TNEAEFFAEAFR[BA*]	116/116	1431.65
		DDIIHSLSQEEK[BA*]	114/116	1413.68
		AFAYYIEPQHR[BA*]	116/116	1394.68
		LDIQPYDINQR[BA*]	116/116	1374.70
		IQVDSSNPLSEK[BA*]	116/116	1316.66
		TFQFINDQIK[BA*]	116/116	1253.65
		HISLEALSEDK[BA*]	116/116	1241.63
		EEGSNLTSYGR[BA*]	116/116	1212.54
		VTNYLVDGNGR[BA*]	116/116	1207.60
		LMHSTDHAER[BA*]	116/116	1196.54
		VPSDVLEMYK[BA*]	116/116	1180.59
		ALNVYYEIGK[BA*]	116/116	1169.62
		IYIVDGDITK[BA*]	116/116	1136.61
		LITFNVHNR[BA*]	116/116	1113.61
		NQSDLVTNSK[BA*]	116/116	1105.54
		NNIQSDLIK[BA*]	116/116	1044.56
4	Polysaccharide	YAAIALCTSAILAGCNTSNVSQEPNK	159/161	2639.28
	deacetylase, YxkH, YjeA	ISYNPITHESTNTTIHMTDIK**	156/161	2416.18
	(>NP_842877.1)	EMVDSGIFSMQSHTATHADLPK*	156/161	2402.11
		VPVLMYHAIDDYHGQGIK	159/161	2056.03
		IHHTTTVEQFASSIK	161/161	1698.88
		VNKPIFVTFDDGMK	161/161	1610.82
		DNGYTLLTFER	161/161	1328.64
		NNMNAFHVLQK	156/161	1315.65
		GEFQIEAIGIK	156/161	1204.65

		QAETVQEQGK**	154/161	1117.54
		DTLTEVQYK	161/161	1096.55
		QFSLPFDTK[BA]	132/161	1082.55
5	Sulfatase, YvgJ, YflE	VFLSTTITSLVLIIFMMVNHFK	25/25	2554.41
	(>NP_847224.2)			
6	ABC transporter	LILENMSLQVEEGEFISILGPSGCGK*	126/156	2763.39
	(>NP_842707.1)			
7	GroEL	NVTAGANPMGLR	275/275	1200.61
	(>NP_842820.)			
8	Manganese superoxide	HHATYVNNLNAALENYSELHNK**	154/154	2552.22
	dismutase, SodA-2	NNGGGHYCHSLFWEVMSPR	154/154	2190.95
	(>NP_847842.1)			
9	Single-stranded DNA-	NDDPFSNVGQPIDISDDDLPF	10/10	2320.02
	binding protein	VYVTEVLAESVQFLEPR	10/10	1979.04
	(>NP_847868.2)	AFANQQGER	10/10	1020.48
10	Universal stress protein	MNNTYTNILIAVDGSK	594/595	1753.87
	(>NP_847076.1)	VDLIMCGATGLNAVER	594/595	1661.83
		ANLFAEDLLEDYK	592/595	1540.75
		NNATLTIAHIVDVK	595/595	1508.84
		FLIGSVSEHIIR	595/595	1370.77
		IETVLEFGNPK	595/595	1246.66
		AYSAVEAYSR	590/595	1116.53
11	Alcohol dehydrogenase	VSDIKPGQPIVIYGCGGLGNLAIQYAK**	172/172	2774.49
	(>NP_844655.1)	NAGYSVDGGMAEQCIVTADYAVK	172/172	2362.06
		VPEGLDPAQASSITCAGVTTYK	170/172	2208.08
		EVGADMTINPISQGPADK	170/172	1842.89
12	Histidinol-phosphate	VTEALTSLASQYALYPDGHAFELR**	152/152	2652.33
	aminotransferase	DGIHDLDAMLQQVDDQTK**	149/152	2041.94
	(>NP_843990.1)	NEEGLHQYYAFCK*	152/152	1601.70
		YENLMVLR	152/152	1037.54
13	Formate acetyltransferase	QMAASHGFDISKPATNAQEAFQWLYFAYLAAIK*	172/172	3688.82
	(A0A0F7RMN6)	DLANGTLTEEDVQEIVDHFIMK**	172/172	2517.21
		ADYGDDYGIACCVSAMR	172/172	1809.72
		THNQGVFDAYTPEMR	172/172	1765.79
		MAEQACESYGYEMDK*	172/172	1754.67
		ADLNLTGGVMSEDTMR*	172/172	1709.78
		IVSSITSHEPGYLNK**	172/172	1644.85
		QLWDQVMDLTTK*	172/172	1477.73
		VALYGVDHLIEAK*	172/172	1427.78
14	Alanine dehydrogenase	LVTEEMIQSMEPGSVVVDIAIDQGGIFETTDR	590/595	3479.69
	(A0A2B0VZ84)	VAMTPAGAVHLVQNGHEVFVQK*	590/595	2332.22
		GAGLGSGFTDEEYVQAGAK	590/595	1856.86
		HGVVHYAVANMPGAVPR	590/595	1774.91
		MSAQIGAQFLEK	590/595	1322.67
		ITTHDNPTYEK	590/595	1318.62
15	Pyruvate dehydrogenase	VMDTPLAESGIGGLAVGLALEGFRPVPEIQFFGFVYE VMDSISGQLAR**	172/172	5083.59
	(NP_846420)	VAAADTVFPFSQAESVWLPNHK**	172/172	2414.21
		NDPNVLVFGEDVGVNGGVFR	172/172	2104.04
		EGTDVSVIAYGAMVHAALK	172/172	1931.98
		QAGIAANVVAEINDR	172/172	1540.80
		VVIPSTPYDAK	172/172	1189.64
16	Superoxide dismutase	NNGGGHANHTFFWTILSPNGGGQPVGELATAIEAK**	590/595	3562.74
	(NP_846724)	HELPNLPYAYDALEPHFDK*	590/595	2269.09
		RPDYIGAFWNVVDWNAAEK*	590/595	2251.09
		SVEELVANLNEVPEAIR*	590/595	1881.99

* Unique to the *B. cereus* sensu lato group with one exception.

** Unique to the *B. cereus* sensu lato group with two exceptions.

BA, Unique to *Bacillus anthracis.*

BA* Unique to *Bacillus anthracis* with one exception.

BA** Unique to *Bacillus anthracis* with two exceptions.

Supplementary Table S4-B: Inclusion list of unique and abundant peptides from selected protein markers of *Brucella abortus*.

S. No.	Protein	Peptide	Strain coverage	Theoretical mass (Da)
1	31-kDa outer-membrane	AGWTLGAGAEYAINNNWTLK[MAS]	216/217	2150.06
	immunogenic protein	SAFNLGDDASALHTWSDK[MAS]	216/217	1934.88
	(>CAJ11595.1)	NLVDVDNSFLESK[MAS]	216/217	1479.73
		LMVYGTGGLAYGK[MAS]	216/217	1329.68
2	DNA starvation/stationary phase	AELDDHVDTIAER[AM]	419/419	1483.68
	protection protein, Dps	**ESIKDADDAGDDDTADIFTAASR[M]**	50/419	2399.01
	(>KFJ51506.1)	LKPYPTDIYAVHDHLVALIER[AM*]	419/419	2463.25
		ALWFLEAHVQESN[AM]	419/419	1543.70
3	60 kDa Chaperonin, GroEL	GIDLAVNEVVAELLK[AM]	425/425	1582.90
	(>KFJ50202.1)			
4	Molecular chaperone DnaK	IELSSSQQTEINLPFITADQTGPK[MAS*]	425/425	2617.33
	(>KFJ51541.1)	LGQAMYEAAQAAEGAGAEGGEQASSSK[MAS]	425/425	2569.14
		**VIGIDMGTTNSCVAVMDGK[M]**	3/425	1910.90
		DDVVDADYEEIDDNK[MAS]	425/425	1754.72
		TTPSIIAFTDGDER	425/425	1522.73
		TSLEGEDAEDIK	425/425	1306.60
5	Ribose-binding protein	AANLKPGDFAIAGIDGITDALHAVK[MAS]	206/206	2478.33
	(>KFJ50308.1)	**MIALAAASALIATPSLAQDK[M]**	-	1956.08
		NGEVELTIFDGR[MAS*]	198/206	1349.66
6	Glyceraldehyde-3-phosphate	VIVSAPADGADLTVVYGVNNDK[MAS]	329/329	2217.13
	dehydrogenase	EENVDIALECTGIFTSR	329/329	1896.90
	(>KFJ54059.1)	**EVEVAGDTIDVGYGPIK[AM]**	136/329	1761.89
7	Fe/Mn family superoxide	YLEAFVDSLVNWDYVLEMYEK[MAS]	396/396	2626.24
	dismutase (>KFJ45857.1)	**NQALFNNAGQHYNHILFWK[AM]**	137/396	2315.14
		HHQAYVTNGNK	396/396	1268.61
		ETLEYHHDK	396/396	1171.53
8	Amino acid ABC transporter	FNYEPDYHAASGASDVEAFAEAIEK[MAS]	307/325	2731.21
	substrate- binding protein	VTNVALVYADDSFDVSVADGTR[MAS]	325/325	2314.11
	(>KFJ53116.1)	AEDVITLGASVQLSGPVANTGR[MAS*]	325/325	2155.13
		GPAGQVNPPQYPMPAWNAR[MAS]	325/325	2050.99
		DANYAFGMTAWLPSADLK[MAS]	325/325	1970.93
		QGQINLPQIVVQVQDGK[MAS]	325/325	1864.02
		FATNSTDFTSLISQIK[MAS*]	323/325	1772.90
		IPMVQGGGASDEIYSR[MAS]	325/325	1679.80
		VLACGVAGLSLMTISAK[MAS]	325/325	1633.90
		FDSLYGPIAFDK[MAS]	325/325	1372.67
		QYTQLVTTDK[MAS]	325/325	1196.61
		DAGFTIAADEK[MAS*]	307/325	1137.54
9	Immunogenic 39-kDa protein	EPTNWDELIALLDNFK[MAS*]	338/338	1917.95
	(>KFJ45961.1)	APVNIHSTNWMWINK	338/338	1810.90
		DWAEQGALGNLDTVASK[MAS]	290/338	1774.86
		GTLLGSMAHGYANPAAVK[MAS]	338/338	1757.90
		AFIDLDPEALGSDTMK	338/338	1722.82
10	Bacterioferritin	LLNDWGYTR[MAS]	331/331	1137.56
	(>KFJ46396.1)	IIFLEGFPNLQTVSPLR[MAS]	323/331	1944.06
		**LNDALFLELGAVNQYWLHYR[M]**	8/331	2435.25
		**IIFFEGFPNLQTVSPLR[AM]**	8/331	1978.07
		YGQLNAAPADEAE	331/331	1348.60
		LLNDWGYTR[MAS]	331/331	1137.56
11	Aminotransferase	LLVEQADVAVAPGVGFGEHGDDYVR[MAS]	439/439	2613.29
	(>KFJ52637.1)	SVQAKPDDNFIPTLER[MAS]	439/439	1829.93
		DENLQNVIPLEGHR[MAS]	439/439	1633.82
		DVLVESFGR	439/439	1021.53

12	Ribosomal protein S1	ADFESLLAESFAEHDLAEGYVVK	338/338	2540.21
	(>KFJ51577.1)	**MAIPDAPGVYMSQSNPTR[AM]**	3/338	1934.90
		LVDHDLDSFIR	338/338	1329.67
13	Isovaleryl-CoA dehydrogenase	QFDQPIGEFQLMQCK[MAS]	329/329	1811.84
	(>CAJ12957.1)	**AYVYAMAAACDR[M]**	-	1304.57
14	Invasion protein B	**QEQSSAQAGQR[MA]**	329/329	1189.51
	(>CAJ12957.1)	IDDTAGPNLTFSTCLPQGCLAPVSFDAK[MAS]	329/329	2881.37
		SGTNINVTTTALSPSQPVAFK[MAS*]	329/329	2133.11
		VDGVLLMPFGLDLAK	-	1587.88
		**QEQSSAQAGQR[M]**	-	1189.55
15	Hypothetical protein	KPVPWENPDTGSQGTITTIAESSTNNK[A]	338/338	2872.39
	EIA50_02275 (AZS90677)			
16	Outer membrane protein OprF	QTVNVYFPQDVTVFRPTSAQINQLHTLLWPVPK[MA]	396/396	3837.04
	(A0A0D4D8X8)	TLVMVACAVSLAACSSPPKP PTVSGR[*MA]	396/396	2542.31
		LQVFPQEPTAQATMWPARPPK[*MA]	396/396	2393.24
		ALAIYNWLINQGVPASR[**MA]	396/396	1886.02
		GLTDNNCPPPGDTQVAR[A]	396/396	1754.81
		IPINSPAAQEELR[*MA]	396/396	1437.76
17	Outer membrane protein 28	ASNFLAASFSTIMLVGAFSLPAFAQENQMTTQPAR[*MA]	396/396	3717.84
	(P0A3U8)	ILDESVTLGVNQGGDLNLVNDNPSAVINEAR[MA]	396/396	3236.63
		TMLAAAPDNSVPIAAGENSYNVSVNVVFE[**MA]	396/396	2979.44
		DLQTGGINIQPIYVYPDDK[MAS]	396/396	2149.08
		VVEISELSRPPMPMPIAR[*MAS]	396/396	2022.08
		EPTITGYSVSTSLTVR[*MAS]	396/396	1710.89
18	31 kDa outer membrane protein	EDNEQVSGSLDVTAGGFVGGVQAGYNWQLDNG VVLGAETDFQGSSVTGSISAGASGLEGK[*M]	-	5888.76
	(P0A3U4)	VMVYGTGGLAYGK[M]	-	1315.67

M – present in *B. melitensis* and additional species

A – present in *B. abortus* and additional species

S – present in *B. suis* and additional species

M (bold) – *B. melitensis* specific

A (bold) – *B. abortus* specific

S (bold) – *B. suis* specific

***** Specific to the taxon with one exception

Supplementary Table S4-C: Inclusion list of unique and abundant peptides from selected protein markers of *Brucella melitensis*.

S. No.	Protein	Peptide	Strain coverage	Theoretical mass (Da)
1	31-kDa outer-membrane	AGWTLGAGAEYAINNNWTLK[MAS]	216/217	2150.06
	immunogenic protein	SAFNLGDDASALHTWSDK[MAS]	201/217	1934.88
	(>AAL54086.1)	NLVDVDNSFLESK[MAS]	217/217	1479.73
		LMVYGTGGLAYGK[MAS]	215/217	1329.68
2	DNA starvation/stationary	AELDDHVDTIAER[AM]	215/217	1483.68
	phase protection protein, Dps	**ESIKDADDAGDDDTADIFTAASR[M]**	215/217	2399.01
	(>AAL53161.1)	LKPYPTDIYAVHDHLVALIER[AM*]	215/217	2463.25
		ALWFLEAHVQESN[AM]	215/217	1543.70
3	60 kDa Chaperonin, GroEL	GIDLAVNEVVAELLK[AM]	424/424	1582.90
	(>AAL54290.1)			
4	Molecular chaperone DnaK	IELSSSQQTEINLPFITADQTGPK[MAS*]	428/428	2617.33
	(>AAL53183.1)	LGQAMYEAAQAAEGAGAEGGEQASSSK[MAS]	428/428	2569.14
		**VIGIDMGTTNSCVAVMDGK[M]**	4/428	1910.90
		DDVVDADYEEIDDNK[MAS]	428/428	1754.72
		TTPSIIAFTDGDER	428/428	1522.73
		TSLEGEDAEDIK	428/428	1306.60
5	Ribose-binding protein	AANLKPGDFAIAGIDGITDALHAVK[MAS]	206/206	2478.33
	(>AAL51923.1)	**MIALAAASALIATPSLAQDK[M]**	3/206	1956.08
		NGEVELTIFDGR[MAS*]	198/206	1349.66
6	Glyceraldehyde-3-phosphate	VIVSAPADGADLTVVYGVNNDK[MAS]	438/438	2217.13
	dehydrogenase	EENVDIALECTGIFTSR	438/438	1896.90
	(>AAL51491.1)	**EVEVAGDTIDVGYGPIK[AM]**	329/438	1761.89
7	Fe/Mn family superoxide	YLEAFVDSLVNWDYVLEMYEK[MAS]	3/3	2626.24
	dismutase (>AAL52548.1)	**NQALFNNAGQHYNHILFWK[AM]**	3/3	2315.14
		HHQAYVTNGNK	3/3	1268.61
		ETLEYHHDK	3/3	1171.53
8	Amino acid ABC transporter	FNYEPDYHAASGASDVEAFAEAIEK[MAS]	325/325	2731.21
	substrate-binding protein	VTNVALVYADDSFDVSVADGTR[MAS]	307/325	2314.11
	(>AIJ87683.1)	AEDVITLGASVQLSGPVANTGR[MAS*]	325/325	2155.13
		GPAGQVNPPQYPMPAWNAR[MAS]	325/325	2050.99
		DANYAFGMTAWLPSADLK[MAS]	325/325	1970.93
		QGQINLPQIVVQVQDGK[MAS]	325/325	1864.02
		FATNSTDFTSLISQIK[MAS*]	325/325	1772.90
		IPMVQGGGASDEIYSR[MAS]	323/325	1679.80
		VLACGVAGLSLMTISAK[MAS]	325/325	1633.90
		FDSLYGPIAFDK[MAS]	325/325	1372.67
		QYTQLVTTDK[MAS]	325/325	1196.61
		DAGFTIAADEK[MAS*]	325/325	1137.54
9	Immunogenic 39-kDa protein	EPTNWDELIALLDNFK[MAS*]	339/339	1917.95
	(>AIJ88453.1)	APVNIHSTNWMWINK	339/339	1810.90
		DWAEQGALGNLDTVASK[MAS]	297/339	1774.86
		GTLLGSMAHGYANPAAVK[MAS]	339/339	1757.90
		AFIDLDPEALGSDTMK	339/339	1722.82
10	Bacterioferritin	LLNDWGYTR[MAS]	605/605	1137.56
	(>AIJ88553.1)	IIFLEGFPNLQTVSPLR[MAS]	596/605	1944.06
		**LNDALFLELGAVNQYWLHYR[M]**	9/605	2435.25
		**IIFFEGFPNLQTVSPLR[AM]**	9/605	1978.07
		YGQLNAAPADEAE	605/605	1348.60
		LLNDWGYTR[MAS]	605/605	1137.56
11	Aminotransferase	LLVEQADVAVAPGVGFGEHGDDYVR[MAS]	432/432	2613.29
	(>AIJ90678.1)	SVQAKPDDNFIPTLER[MAS]	432/432	1829.93
		DENLQNVIPLEGHR[MAS]	432/432	1633.82
		DVLVESFGR	432/432	1021.53
12	Ribosomal protein S1	ADFESLLAESFAEHDLAEGYVVK	335/335	2540.21

	(>AAL53096.1)	**MAIPDAPGVYMSQSNPTR**^AM^	4/335	1934.90
		LVDHDLDSFIR	335/335	1329.67
13	Isovaleryl-CoA	QFDQPIGEFQLMQCK^MAS^	293/293	1811.84
	Dehydrogenase(>AAL53104.1)	**AYVYAMAAACDR**^M^	14/293	1304.57
14	Invasion protein B	**QEQSSAQAGQR**^MA^	138/344	1189.51
	(>AIJ89668.1)	IDDTAGPNLTFSTCLPQGCLAPVSFDAK^MAS^	344/344	2881.37
		SGTNINVTTTALSPSQPVAFK^MAS*^	344/344	2133.11
		VDGVLLMPFGLDLAK	344/344	1587.88
		**QEQSSAQAGQR**^M^	138/344	1189.55
15	Hypothetical protein	KPVPWENPDTGSQGTITTIAESSTNNK^A^		2872.39
	EIA50_02275 (AZS90677)			
16	Outer membrane protein OprF	QTVNVYFPQDVTVFRPTSAQINQLHTLLWPVPK^MA^	344/344	3837.04
	(A0A0D4D8X8)	TLVMVACAVSLAACSSPPKP PTVSGR^*MA^	344/344	2542.31
		LQVFPQEPTAQATMWPARPPK^*MA^	344/344	2393.24
		ALAIYNWLINQGVPASR^**MA^	344/344	1886.02
		GLTDNNCPPPGDTQVAR^A^	-	1754.81
		IPINSPAAQEELR^*MA^	344/344	1437.76
17	Outer membrane protein 28	ASNFLAASFSTIMLVGAFSLPAFAQENQMTTQPAR^*MA^	344/344	3717.84
	(P0A3U8)	ILDESVTLGVNQGGDLNLVNDNPSAVINEAR^MA^	344/344	3236.63
		TMLAAAPDNSVPIAAGENSYNVSVNVVFE^**MA^	344/344	2979.44
		DLQTGGINIQPIYVYPDDK^MAS^	344/344	2149.08
		VVEISELSRPPMPMPIAR^*MAS^	344/344	2022.08
		EPTITGYSVSTSLTVR^*MAS^	344/344	1710.89
18	31 kDa outer membrane protein	EDNEQVSGSLDVTAGGFVGGVQAGYNWQLDNG VVLGAETDFQGSSVTGSISAGASGLEGK^*M^	344/344	5888.76
	(P0A3U4)	VMVYGTGGLAYGK^M^	344/344	1315.67

M – present in *B. melitensis* and additional species

A – present in *B. abortus* and additional species

S – present in *B. suis* and additional species

M (bold) – *B. melitensis* specific

A (bold) – *B. abortus* specific

S (bold) – *B. suis* specific

***** Specific to the taxon with one exception

Supplementary Table S4-D: Inclusion list of unique and abundant peptides from selected protein markers of *Brucella suis*.

S. No.	Protein	Peptide	Strain coverage	Theoretical mass (Da)
1	31-kDa outer-membrane	AGWTLGAGAEYAINNNWTLK[MAS]	92/92	2150.06
	immunogenic protein	SAFNLGDDASALHTWSDK[MAS]	92/92	1934.88
	(>AAN30527.1)	NLVDVDNSFLESK[MAS]	92/92	1479.73
		LMVYGTGGLAYGK[MAS]	92/92	1329.68
2	DNA starvation/stationary	AELDDHVDTIAER[AM]	-	1483.68
	phase protection protein, Dps	**ESIKDADDAGDDDTADIFTAASR[M]**	-	2399.01
	(>AAN31039.1)	LKPYPTDIYAVHDHLVALIER[AM*]	-	2463.25
		ALWFLEAHVQESN[AM]	-	1543.70
3	60 kDa Chaperonin, GroEL	GIDLAVNEVVAELLK[AM]	-	1582.90
	(>AAN33401.1)			
4	Molecular chaperone DnaK	IELSSSQQTEINLPFITADQTGPK[MAS*]	426/426	2617.33
	(>AAN31015.1)	LGQAMYEAAQAAEGAGAEGGEQASSSK[MAS]	426/426	2569.14
		**VIGIDMGTTNSCVAVMDGK[M]**	-	1910.90
		DDVVDADYEEIDDNK[MAS]	426/426	1754.72
		TTPSIIAFTDGDER	426/426	1522.73
		TSLEGEDAEDIK	426/426	1306.60
5	Ribose-binding protein	AANLKPGDFAIAGIDGITDALHAVK[MAS]	227/227	2478.33
	(>AAN30155.1)	**MIALAAASALIATPSLAQDK[M]**	-	1956.08
		NGEVELTIFDGR[MAS*]	227/227	1349.66
6	Glyceraldehyde-3-phosphate	VIVSAPADGADLTVVYGVNNDK[MAS]	227/227	2217.13
	dehydrogenase	EENVDIALECTGIFTSR	227/227	1896.90
	(>AAN30627.1)	**EVEVAGDTIDVGYGPIK[AM]**	-	1761.89
7	Fe/Mn family superoxide	YLEAFVDSLVNWDYVLEMYEK[MAS]	259/259	2626.24
	dismutase (>AAN29497.1)	**NQALFNNAGQHYNHILFWK[AM]**	-	2315.14
		HHQAYVTNGNK	259/259	1268.61
		ETLEYHHDK	259/259	1171.53
8	Amino acid ABC transporter	FNYEPDYHAASGASDVEAFAEAIEK[MAS]	157/157	2731.21
	substrate-binding protein	VTNVALVYADDSFDVSVADGTR[MAS]	157/157	2314.11
	(>AAN28972.1)	AEDVITLGASVQLSGPVANTGR[MAS*]	157/157	2155.13
		GPAGQVNPPQYPMPAWNAR[MAS]	157/157	2050.99
		DANYAFGMTAWLPSADLK[MAS]	157/157	1970.93
		QGQINLPQIVVQVQDGK[MAS]	157/157	1864.02
		FATNSTDFTSLISQIK[MAS*]	157/157	1772.90
		IPMVQGGGASDEIYSR[MAS]	157/157	1679.80
		VLACGVAGLSLMTISAK[MAS]	157/157	1633.90
		FDSLYGPIAFDK[MAS]	157/157	1372.67
		QYTQLVTTDK[MAS]	157/157	1196.61
		DAGFTIAADEK[MAS*]	157/157	1137.54
9	Immunogenic 39-kDa protein	EPTNWDELIALLDNFK[MAS*]	298/300	1917.95
	(>AAN33879.1)	APVNIHSTNWMWINK	292/300	1810.90
		DWAEQGALGNLDTVASK[MAS]	248/300	1774.86
		GTLLGSMAHGYANPAAVK[MAS]	300/300	1757.90
		AFIDLDPEALGSDTMK	300/300	1722.82
10	Bacterioferritin	LLNDWGYTR[MAS]	157/157	1137.56
	(>AAN33754.1)	IIFLEGFPNLQTVSPLR[MAS]	149/157	1944.06
		**LNDALFLELGAVNQYWLHYR[M]**	-	2435.25
		**IIFFEGFPNLQTVSPLR[AM]**	-	1978.07
		YGQLNAAPADEAE	157/157	1348.60
		LLNDWGYTR[MAS]	157/157	1137.56
11	Aminotransferase	LLVEQADVAVAPGVGFGEHGDDYVR[MAS]	428/428	2613.29
	(>AAN30193.1)	SVQAKPDDNFIPTLER[MAS]	428/428	1829.93
		DENLQNVIPLEGHR[MAS]	428/428	1633.82
		DVLVESFGR	428/428	1021.53
12	Ribosomal protein S1	ADFESLLAESFAEHDLAEGYVVK	310/310	2540.21

	(>AAN28984.1)	**MAIPDAPGVYMSQSNPTR**$^{\mathbf{AM}}$	-	1934.90
		LVDHDLDSFIR	310/310	1329.67
13	Isovaleryl-CoA dehydrogenase	QFDQPIGEFQLMQCK^{MAS}	394/395	1811.84
	(>AAN28977.1)	**AYVYAMAAACDR**$^{\mathbf{M}}$	-	1304.57
14	Invasion protein B	**QEQSSAQAGQR**$^{\mathbf{MA}}$	-	1189.51
	(>AAN33764.1)	IDDTAGPNLTFSTCLPQGCLAPVSFDAK^{MAS}	-	2881.37
		SGTNINVTTTALSPSQPVAFK^{MAS*}	-	2133.11
		VDGVLLMPFGLDLAK	-	1587.88
		**QEQSSAQAGQR**$^{\mathbf{M}}$	-	1189.55
15	Hypothetical protein	KPVPWENPDTGSQGTITTIAESSTNNK^{A}	-	2872.39
	EIA50_02275 (AZS90677)			
16	Outer membrane protein OprF	QTVNVYFPQDVTVFRPTSAQINQLHTLLWPVPK^{MA}	-	3837.04
	(A0A0D4D8X8)	TLVMVACAVSLAACSSPPKP PTVSGR^{*MA}	-	2542.31
		LQVFPQEPTAQATMWPARPPK^{*MA}	-	2393.24
		ALAIYNWLINQGVPASR^{**MA}	-	1886.02
		GLTDNNCPPPGDTQVAR^{A}	-	1754.81
		IPINSPAAQEELR^{*MA}	-	1437.76
17	Outer membrane protein 28	ASNFLAASFSTIMLVGAFSLPAFAQENQMTTQPAR^{*MA}	-	3717.84
	(P0A3U8)	ILDESVTLGVNQGGDLNLVNDNPSAVINEAR^{MA}	-	3236.63
		TMLAAAPDNSVPIAAGENSYNVSVNVVFE^{**MA}	-	2979.44
		DLQTGGINIQPIYVYPDDK^{MAS}	310/310	2149.08
		VVEISELSRPPMPMPIAR^{*MAS}	310/310	2022.08
		EPTITGYSVSTSLTVR^{*MAS}	310/310	1710.89
18	31 kDa outer membrane protein	EDNEQVSGSLDVTAGGFVGGVQAGYNWQLDNG VVLGAETDFQGSSVTGSISAGASGLEGK^{*M}	-	5888.76
	(P0A3U4)	VMVYGTGGLAYGK^{M}	-	1315.67

M – present in *B. melitensis* and additional species

A – present in *B. abortus* and additional species

S – present in *B. suis* and additional species

M (bold) – *B. melitensis* specific

A (bold) – *B. abortus* specific

S (bold) – *B. suis* specific

***** Specific to the taxon with one exception

Supplementary Table S4-E: Inclusion list of unique and abundant peptides from selected protein markers of *Burkholderia mallei*.

S. No.	Protein	Peptide	Strain coverage	Theoretical mass (Da)
1	ABC transporter	**LATGPAGFGAAAGQRPLAPLNAK**	687/689	2149.18
	(>YP_102545.1)	**NLNFFYNQFHALK****	689/689	1655.83
		**MNMAESHLDPSK***	684/689	1359.60
2	Autotransporter, BpaA[a]	Specific to *B. pseudomallei*	-	-
	(>AAO19442.1)			
	Autotransporter, BpaB[a]	Specific to *B. pseudomallei*	-	-
	(>AAO19443.1)			
	Autotransporter BpaC[a]	Specific to *B. pseudomallei*	-	-
	(>WP_004540242.1)			
3	Phasin-like protein, PhaP,	**MNMSLLTPEQIAAAQK***	22/692	1745.89
	PhaZ	**ANIESLFGLTTK***	692/692	1293.70
	(>YP_103336.1)			
4	Burkholderia intracellular	**STAIGAEANASGQNTVALGAGSIADR**	-	2402.19
	motility A, BimA[a]	DAYSGVAAATALTMIPDVDR*[a]	-	2036.99
	(>WP_080306776.1)	**AGVAMSAGGNAVGIGMSWQW**	-	1949.89
		**IGDLQQSITDTAR****	-	1417.72
		**VSIGVGGAVYK**	-	1049.59
5	Outer membrane porin	**NANASIYNGDLSTPFSTSINQTAATVGLR***	1192/1192	2983.47
	(>YP_105969.1)	**FTSANYAGLQFGGTYSFSNNSQFANNR****	1192/1192	2963.33
		VYGAGASYAYGPLQGGLLWTQSR	1192/1192	2415.20
		YNLTPALGLGVAYTYTYTNAK[b]	53/1192	2294.17
		**ANGESTHWNQVGVQADYALSK***	1192/1192	2275.07
		QAFVGLSSNYGTVTLGR**	1192/1192	1769.91
		AIFTLESGFNIGNGR	1192/1192	1595.81
		AYSAGASYQFQGLK	1192/1192	1490.72
		**LNTNGDVAVNNTVK***	1192/1192	1458.75
		**SLWSVGAGVDQSR***	1192/1192	1361.68
		TDVYAQAVYQR	1192/1192	1313.64
		**LDNLANGAPTIR***	1192/1192	1254.68
		**FNNGGGMFNR****	1192/1192	1113.48
		**ADNYEANVK****	1192/1192	1023.47
6	60 kDa chaperonin,GroEL	**TAIASLTGVNADQNAGIK**	61/61	1743.92
	(>YP_103588.1)			
7	Phage major tail sheath	**QQVANGYLIGGSAWIDPEPNTADILASGK***	-	2985.49
	protein[a]	TAQVAGDSIAEAQMPVVDGPLNPSLAR*[a]	-	2707.37
	(>WP_038737779.1)	ILAAPGLDTQPVAAALAATA QSLR*[a]	-	2319.30
		AYIDYDYTPVPPLENLVLR*[a]	-	2251.16
		DADETTSNVIGTVTPDGK**[a]	-	1819.85
		ALLAAQGALGVKPR*[a]	-	1364.83
		VIEINEGGRPIR	-	1352.76
		DIVESINGWFR*[a]	-	1335.66
		AMAYVSASGCK*[a]	-	1087.49
8	Outer membrane protein A,	**CDGALVAQAPAPAPVAPVAPAITSQK****	663/663	2443.30
	OmpA (>YP_102249.1)	IQGMNVEVVVATGYTDR	663/663	1851.92
9	TonB-dependent copper	**FEGSLAYAWGR**	51/51	1256.61
	receptor	AYAEHLNLAGNAGFGYPANLPVTEPGR[a]	-	2799.38
	(>YP_105214.1)	IDAQVFYNEADHVMDNYTLR[a]	-	2414.10
		DFGPSAGFGVLSLHAQYNVSK	51/51	2194.09
		DLASLPVTWYAGIGHAQR*	51/51	1955.01
		**NDQNVDVTAGTPDFYGR****	51/51	1868.84
		**VSANHAHSQDYEDGNGR****	51/51	1856.79
		**NVQSGAPLPQMPPLEAR****	51/51	1804.93
		**WNADAALGWTPDDNTR****	51/51	1802.80

		LNILANGMPTLGACPGR*	51/51	1697.88
		**GPNGSINAFSAIKPEK**	49/51	1629.85
		**TVQISVGVDNVLDK***	51/51	1486.81
		FPDYWELFSAK*	51/51	1402.66
		MPDPTSSMPMR*	51/51	1249.53
10	Heat shock Hsp20	**LGGFPALNVGTTEDSIEIVVFAPGMR****	687/1316	2690.38
	(>YP_104544.1)	**MSDLYFGADLFSEFDR**	687/1316	1912.84
		**QMANLFGGFPSSIR**	687/1316	1524.76
		VVELPQNADPDK**	687/1316	1324.67
		**YENGCLLISVGK***	687/1316	1295.66
		AADFDVSIDK	687/1316	1080.52
11	Translocator protein, BipB,	**LMQPVMDAILKPLMEMISSLITK**	231/231	2602.40
	(>YP_106121.1)	AELAGAILGAVVTGVALVAAAFVGASAVK	231/231	2596.50
		**ACEQQVDDAVNQATQQYGASASLR**	231/231	2553.16
		AGTVMNVGNQVSQAAGGIVVGVER	231/231	2313.19
		DAASALGTLSPQAYVDVVSAAQR	231/231	2290.16
		VLAQLMQQMSDAGEMQTSTGK	231/231	2254.05
		**MSSGVQGGPAANANAYQTHPLR**	231/231	2227.06
		LTELLGVLMSVISASSLDELK	231/231	2218.22
		**MSQLASEQCDAQPAAHDAR**	231/231	2028.88
		QAVDAFAEHNR	231/231	1257.59
		**DAPPLGASDTGSR**	231/231	1243.59
		LSGAAELTAVLGK	231/231	1229.70
		**AAADAAEQAAAAAK**	231/231	1229.61
		LQELISSGNVK	231/231	1187.66
		**LDAASALATQAR**	231/231	1187.63
		VIDAMAGQLTK	231/231	1146.61
		ALVACGVDQQK	231/231	1131.58
		SDIWNQMSK	231/231	1108.50
		SDEYQAQVK	231/231	1067.50
		ATTGVSFMDK	231/231	1056.50
		AADAAVDAAQK	231/231	1030.51
		EDGMLLANR	231/231	1018.49
		ADALQADATK	231/231	1003.50
12	Translocator protein, BipD[a]	ELGDAVSISDSGVVTINPDK	-	2016.01
	(>ABN85883.1)	ALIQQVIDHLPTMQLPK	-	1945.09
		**SDIDAYATIVEGLTK**	-	1595.81
		YSHQNSNFDNLVK	-	1565.73
		DSLPPDGTVWDTAR	-	1529.72
		DNIQNDVQTLVEK	-	1515.76
		YQAWNTAFSGQK	-	1400.65
		VLSGAISTLTDTAK	-	1376.76
		**LGAAIDEFASLR**	-	1262.67
		**ANLTVFDDAR***	-	1121.55
		**DWPALEALAK***[a]	-	1113.59
		**MNMHVDMGR***[a]	-	1090.45
13	Peroxidase/catalase	**THGAGPASNVGAEPEAAGIEAQGLGWK**	282/282	2575.25
	(>YP_103946.1)	**ADASQEQTDVESMAVLEPVADGFR**	282/282	2565.17
		**DWEANQPEQLAAVLETLEAIR**	282/282	2396.20
		ADTWEPEDVYWGSEK	282/282	1811.77
		**DWWPNQLDLSILHR**	282/282	1792.91
		**CPFHQAAGNGTSNR**	282/282	1459.64
		DFNYAQAFEK	282/282	1232.55
14	Type III secretion system	AGSITAFYTVLLENEEEPDPIGDEIR	-	2878.39
	(TTSS) effector protein,	TSLMNMMIEHAAADVYVVALIGER	-	2634.30
	BopE, BsaP, BsaU, BsaE	RPALDAFLQQAVDEASAFGDTLER	-	2620.30
	(>YP_106114.1)	**IQGPIIEAPLPEVAIGELCAIR**	-	2302.28
		**IDEMQVFLDLGEYR**	-	1727.83
		DLGCDVMLFLDSMTR	-	1715.78

		CNAALVATTIAEYFR	-	1642.82
		DTAILSTLGSTAGLSR	-	1562.83
		GYPASVFEQLPR	-	1363.70
		GENPENDDALDR	-	1344.56
		LFDEIADAPHR	-	1283.63
		**AIDGLLACGVGQR**	-	1272.67
		AIDAAPPDYAAR	-	1230.61
		DLALATGEAPAR	-	1184.62
		GHFPAIDVLR	-	1124.62
		TVVVYATSDR	-	1110.57
		QVALVPTGER	-	1069.60
		FGIFAAAGCGK*	-	1041.51
		AAAGSDTTIGR	-	1019.51
15	ScoA/B	**ITIAEVEEIVENGELDPDAIHTPGIFVQR**	-	3204.64
	(>YP_102101.1)			
16	Peptidyl-prolyl cis-trans	LLLALGSAALLATAPAFAQSAATHPVVQLK	644/644	2943.70
	isomerase B, PpiB	DVPEKPIVIESATIVSK	644/644	1825.03
	(>YP_103284.1)	TNFDEKPTR	644/644	1107.54
		GFMIQGGGYK*	644/644	1057.51
		IEATPTTVR	644/644	987.54

* Unique to the taxon with one exception. Peptides in bold letters are unique to the target species.

[a] Specific to *B. pseudomallei*

[b] Specific to *B. mallei*

Supplementary Table S4-F: Inclusion list of unique and abundant peptides from selected protein markers of *Burkholderia pseudomallei*.

S. No.	Protein	Peptide	Strain coverage	Theoretical mass (Da)
1	ABC transporter	LATGPAGFGAAAGQRPLAPLNAK	621/630	2149.18
	(>ABN83707.1)	NLNFFYNQFHALK**	630/630	1655.83
		MNMAESHLDPSK*	630/630	1359.60
2	Autotransporter, BpaA	STTVSGVGGATDAVLLNAGANTLGTVTVNTAK	13/13	2960.55
	(>AAO19442.1)	APLGYSTTANNQVILGTVSGVPSADAPASR	13/13	2914.49
		NIVDTPEETNYGDIGIGTDTPTSNR*	13/13	2679.23
		ATVPAILASALALNAATGIGATNAPIR	13/13	2518.43
		SGVYLTALDTNASGTGVVIGGGVTSAK*	10/13	2495.29
		SGDQYQNTWWNHLSGDIADMR	13/13	2494.08
		DITVTDAGGINPSAGTAPVSLVANR	13/13	2396.24
		ASTTAGSGSLNVASAMTTANGGYIR*	13/13	2358.13
		LGANQVANQGLNNPVGAATWFK	13/13	2270.16
		GMIDVYAWGNTTDTSNITIR	9/13	2228.06
		FTFGMPVLGPVMSCTGDEDR	13/13	2158.96
		NTGNITSNGAGVAIYAVAGGK	13/13	1934.99
		VSFDTPTNQVQLRPAR*	6/13	1828.96
		AGYDFATGESDLFGELK	6/13	1819.83
		YQQYACIRPLSISAR	13/13	1768.91
		VVGSLPSSINGLVQGNGR	4/13	1753.95
		GSIGSASQVINTAVNR	10/13	1573.82
		AGYNLWNYDTAVK	8/13	1514.72
		GAVFGYGGSQVLTPR	4/13	1508.78
		WSSPYGSSSYIPR	13/13	1486.69
		LTTTSGSVAVAGYR	13/13	1382.72
		FIAPNTASATLNR	5/13	1375.73
		TDNTFSNLDFR	6/13	1329.60
		SQGDIGVGADFTR*	13/13	1322.63
		LTFMDPATLAAR	13/13	1306.68
		GATQPILASSAIR	10/13	1284.72
		QTSGSLTDTITR	13/13	1279.64
		SLTTGTGNIDIR	13/13	1247.65
		YFVGNGLFYK*	13/13	1207.61
		NFNVGSGETVR	5/13	1179.57
		DLDILGAGLSR	10/13	1129.62
	Autotransporter, BpaB	FTFAPGFNSDDSFNAARPGAESDYR	8/10	2739.20
	(>AAO19443.1)	ASTIASEPAAASTEQLSASSMPVLAGAR	5/10	2674.33
		QSLVQPGNISAEPGIPTTDLEGLEAK	10/10	2664.37
		FNGQWANGPIISSEQLQISGAAAVR*	10/10	2614.33
		DTLTFTGNYSSSNAAIEDGTFNVK	7/10	2552.17
		WVDPWLFSLVVLGCGIGAAVPAR	2/10	2426.30
		TSSSQDSQQITQALEQLR	3/10	2019.99
		ELMEHTLQYTMPTSYR	10/10	1999.92
		VYGSWDNTGTSLTGLNR	10/10	1840.88
		GPSSLPSYVVHGSVVIAY	10/10	1831.95
		LRPFIGQPLDSSLIQK	7/10	1812.03
		TLTDDLTFLNTSPWR	10/10	1779.89
		TGVNWGDAFGIVGSR	10/10	1535.76
		EAAQEQCGGIGIPSR	7/10	1515.72
		DITRPQPSSDEAAK	10/10	1514.74
		MLPSDLAVSPPSQR	5/10	1497.77
		YFNLPAGFVLHGR	10/10	1490.79
		LPALSSQAVADSYR	7/10	1477.76
		LAAAADELMATFSR	2/10	1466.73

		**LNDANVLQTSIDR**	3/10	1458.75
		**TVAALQQQLR**	3/10	1127.65
		**LRPGDNVDLK***	10/10	1126.62
		**GDPQMNVSLK**	9/10	1088.54
		**FTLASVGVGAR**	10/10	1077.60
		**DQLSAVPTAR**	2/10	1057.56
		**EIGSITLASR***	10/10	1046.58
	Autotransporter, BpaC	**ETAVVPLSAGEMVAAAWHYPLVFVR**	7/10	2713.41
	(>WP_004540242.1)	**TSALCAALVDQDILEPLTATITQPGK**	8/10	2669.40
		**DGNAMNEWTQIEAFLK**	8/10	1866.86
		**VTVNGEADPNLLVCVEK**	10/10	1799.92
		**SSWNVTGFLGVNEGK***	8/10	1594.78
		**DLPDETVIEWHR**	8/10	1509.73
		**DYVEEAADEGCR**	4/10	1356.53
		**YSSPQSLPPFVK**	4/10	1349.70
		**IGLHLFSLQR**	10/10	1183.69
3	Phasin-like protein, PhaP,	**MNMSLLTPEQIAAAQK***	23/695	1745.89
	PhaZ	**ANIESLFGLTTK***	695/695	1293.70
	(>ABN83492.1)			
4	Burkholderia intracellular	**STAIGAEANASGQNTVALGAGSIADR**	-	2402.19
	motility A, BimA	DAYSGVAAATALTMIPDVDR*[a]	290/290	2036.99
	(>WP_080306776.1)	**AGVAMSAGGNAVGIGMSWQW**	281/290	1949.89
		**IGDLQQSITDTAR****	290/290	1417.72
		**VSIGVGGAVYK**	265/290	1049.59
5	Outer membrane porin	**NANASIYNGDLSTPFSTSINQTAATVGLR***	662/663	2983.47
	(>ABN87764.1)	**FTSANYAGLQFGGTYSFSNNSQFANNR****	663/663	2963.33
		VYGAGASYAYGPLQGGLLWTQSR	660/663	2415.20
		YNLTPALGLGVAYTYTYTNAK[b]	-	2294.17
		**ANGESTHWNQVGVQADYALSK***	660/663	2275.07
		QAFVGLSSNYGTVTLGR**	663/663	1769.91
		AIFTLESGFNIGNGR	663/663	1595.81
		AYSAGASYQFQGLK	663/663	1490.72
		**LNTNGDVAVNNTVK***	663/663	1458.75
		**SLWSVGAGVDQSR***	662/663	1361.68
		TDVYAQAVYQR	663/663	1313.64
		**LDNLANGAPTIR***	504/663	1254.68
		**FNNGGGMFNR****	663/663	1113.48
		**ADNYEANVK****	663/663	1023.47
6	60 kDa chaperonin,GroEL	**TAIASLTGVNADQNAGIK**	316/654	1743.92
	(>ABN82526.1)			
7	Phage major tail sheath	**QQVANGYLIGGSAWIDPEPNTADILASGK***	345/367	2985.49
	protein	TAQVAGDSIAEAQMPVVDGPLNPSLAR*[a]	93/367	2707.37
	(>WP_038737779.1)	ILAAPGLDTQPVAAALAATA QSLR*[a]	305/367	2319.30
		AYIDYDYTPVPPLENLVLR*[a]	361/367	2251.16
		DADETTSNVIGTVTPDGK**[a]	328/367	1819.85
		ALLAAQGALGVKPR*[a]	356/367	1364.83
		VIEINEGGRPIR	356/367	1352.76
		DIVESINGWFR*[a]	350/367	1335.66
		AMAYVSASGCK*[a]	354/367	1087.49
8	Outer membrane protein A,	**CDGALVAQAPAPAPVAPVAPAITSQK****	678/681	2443.30
	OmpA (>ABN85030.1)	IQGMNVEVVVATGYTDR	681/681	1851.92
9	TonB-dependent copper	**FEGSLAYAWGR**	724/724	1256.61
	receptor	AYAEHLNLAGNAGFGYPANLPVTEPGR[a]	641/724	2799.38
	(>WP_004546785.1)	IDAQVFYNEADHVMDNYTLR[a]	25/724	2414.10
		DFGPSAGFGVLSLHAQYNVSK	722/724	2194.09
		DLASLPVTWYAGIGHAQR*	724/724	1955.01
		**NDQNVDVTAGTPDFYGR****	724/724	1868.84
		**VSANHAHSQDYEDGNGR****	713/724	1856.79
		**NVQSGAPLPQMPPLEAR****	718/724	1804.93

		**WNADAALGWTPDDNTR****	724/724	1802.80
		LNILANGMPTLGACPGR*	724/724	1697.88
		**GPNGSINAFSAIKPEK**	708/724	1629.85
		**TVQISVGVDNVLDK***	713/724	1486.81
		FPDYWELFSAK*	724/724	1402.66
		MPDPTSSMPMR*	722/724	1249.53
10	Heat shock Hsp20	**LGGFPALNVGTTEDSIEIVVFAPGMR****	697/704	2690.38
	(>ABN84084.1)	**MSDLYFGADLFSEFDR**	704/704	1912.84
		**QMANLFGGFPSSIR**	704/704	1524.76
		VVELPQNADPDK**	704/704	1324.67
		**YENGCLLISVGK***	704/704	1295.66
		AADFDVSIDK	704/704	1080.52
11	Translocator protein, BipB	**LMQPVMDAILKPLMEMISSLITK**	753/1418	2602.49
	(>ABO26346.1)	AELAGAILGAVVTGVALVAAAFVGASAVK	753/1418	2596.50
		**ACEQQVDDAVNQATQQYGASASLR**	753/1418	2553.16
		AGTVMNVGNQVSQAAGGIVVGVER	746/1418	2313.19
		DAASALGTLSPQAYVDVVSAAQR	746/1418	2290.16
		VLAQLMQQMSDAGEMQTSTGK	753/1418	2254.05
		**MSSGVQGGPAANANAYQTHPLR**	334/1418	2227.06
		LTELLGVLMSVISASSLDELK	753/1418	2218.22
		**MSQLASEQCDAQPAAHDAR**	753/1418	2028.88
		QAVDAFAEHNR	753/1418	1257.59
		**DAPPLGASDTGSR**	753/1418	1243.59
		LSGAAELTAVLGK	753/1418	1229.70
		**AAADAAEQAAAAAK**	747/1418	1229.61
		LQELISSGNVK	753/1418	1187.66
		**LDAASALATQAR**	753/1418	1187.63
		VIDAMAGQLTK	753/1418	1146.61
		ALVACGVDQQK	753/1418	1131.58
		SDIWNQMSK	753/1418	1108.50
		SDEYQAQVK	751/1418	1067.50
		ATTGVSFMDK	753/1418	1056.50
		AADAAVDAAQK	753/1418	1030.51
		EDGMLLANR	753/1418	1018.49
		ADALQADATK	745/1418	1003.50
12	Translocator protein, BipD	ELGDAVSISDSGVVTINPDK	665/1418	2016.01
	(>ABN85883.1)	ALIQQVIDHLPTMQLPK	665/1418	1945.09
		**SDIDAYATIVEGLTK**	665/1418	1595.81
		YSHQNSNFDNLVK	665/1418	1565.73
		DSLPPDGTVWDTAR	665/1418	1529.72
		DNIQNDVQTLVEK	664/1418	1515.76
		YQAWNTAFSGQK	664/1418	1400.65
		VLSGAISTLTDTAK	664/1418	1376.76
		**LGAAIDEFASLR**	665/1418	1262.67
		**ANLTVFDDAR***	665/1418	1121.55
		**DWPALEALAK***[a]	665/1418	1113.59
		**MNMHVDMGR***[a]	665/1418	1090.45
13	Peroxidase / Catalase	**THGAGPASNVGAEPEAAGIEAQGLGWK**	743/745	2575.25
	(>ABN83678.1)	**ADASQEQTDVESMAVLEPVADGFR**	745/745	2565.17
		**DWEANQPEQLAAVLETLEAIR**	743/745	2396.20
		ADTWEPEDVYWGSEK	745/745	1811.77
		**DWWPNQLDLSILHR**	745/745	1792.91
		**CPFHQAAGNGTSNR**	745/745	1459.64
		DFNYAQAFEK	732/745	1232.55
14	Type III secretion system	AGSITAFYTVLLENEEEPDPIGDEIR	23/25	2878.39
	(TTSS) effector protein,	TSLMNMMIEHAAADVYVVALIGER	21/25	2634.30
	BopE, BsaP, BsaU, BsaE	RPALDAFLQQAVDEASAFGDTLER	22/25	2620.30
	(>OMS79528.1)	**IQGPIIEAPLPEVAIGELCAIR**	22/25	2302.28
		**IDEMQVFLDLGEYR**	25/25	1727.83

		DLGCDVMLFLDSMTR	23/25	1715.78
		CNAALVATTIAEYFR	25/25	1642.82
		DTAILSTLGSTAGLSR	25/25	1562.83
		GYPASVFEQLPR	25/25	1363.70
		GENPENDDALDR	25/25	1344.56
		LFDEIADAPHR	25/25	1283.63
		**AIDGLLACGVGQR**	23/25	1272.67
		AIDAAPPDYAAR	25/25	1230.61
		DLALATGEAPAR	25/25	1184.62
		GHFPAIDVLR	23/25	1124.62
		TVVVYATSDR	25/25	1110.57
		QVALVPTGER	25/25	1069.60
		FGIFAAAGCGK*	25/25	1041.51
		AAAGSDTTIGR	25/25	1019.51
15	ScoA/B	**ITIAEVEEIVENGELDPDAIHTPGIFVQR**	696/703	3204.64
	(>YP_108553.1)			
16	Peptidyl-prolyl cis-trans	LLLALGSAALLATAPAFAQSAATHPVVQLK	675/709	2943.70
	isomerase B, PpiB	DVPEKPIVIESATIVSK	709/709	1825.03
	(>YP_108840.1)	TNFDEKPTR	706/709	1107.54
		GFMIQGGGYK*	709/709	1057.51
		IEATPTTVR	709/709	987.54

* Unique to the taxon with one exception. Peptides in bold letters are unique to the target species.

[a] Specific to *B. pseudomallei*

[b] Specific to *B. mallei*

Supplementary Table S4-G: Inclusion list of unique and abundant peptides from selected protein markers of *Clostridium botulinum*.

S. No.	Protein	Peptide	Strain coverage	Theoretical mass (Da)
1	Botulinum neurotoxin type G,	FTYGFQPDQFNASTGVFSK**	3/3	2140.0
	BoNT/G	DVYEYYDPTYLK**	3/3	1567.7
	(>CAA52275.1)	FAANVANVSINK**	3/3	1246.7
		FCPSCLNVFNNVQENK**	3/3	1968.9
		ALQNFQDIANR**	3/3	1288.7
		LNIVSSAQGSGIDISLYK**	3/3	1864.0
		ALMFGFTETNLAGEYGIK**	3/3	1961.0
		YSYFSEYLPPIK**	3/3	1505.7
		LLDNTIYTQNEGFNIASK**	3/3	2040.0
		AVNKEAYEEISLEHLVIYR**	3/3	2275.2
		GVIDDFTSESTWK**	0/3	1425.7
		IIWTLIDVNAK**	3/3	284.7
		FVWIKDFNIFGR**	3/3	1540.8
		TNFNNAAINYQNLYLGLR**	3/3	2098.1
2	Botulinum neurotoxin type B,	EGFGGIMQMK**	109/109	1097.5
	(>AIJ01283.1)	LGCNWQFIPK*	104/109	1262.6
		YFSIFNTELSQSNIEER	14/109	2077.0
		LISNPGEVER*	81/109	1112.6
		VDDLPIVPNEK	92/109	1237.7
		YSIDVESFDK*	83/109	1201.6
		ASYFSDSLPPVK**	109/109	1309.7
		NLLDNEIYTIEEGFNISDK**	108/109	2226.1
		IFTDENTIFQYLYSQTFPLDIR*	75/109	2723.4
		QIVNDFVIEANK	14/109	1388.7
		TIDNALTK	94/109	874.5
		ALNYQAQALEEIIK**	109/109	1603.8
		SNINIDFNDINSK	63/109	1492.7
		LLDFDNTLK	91/109	1077.6
		NLLNYIDENK**	109/109	1234.6
		LYLIGSAEYEK*	78/109	1284.7
		YNSEILNNIILNLR	94/109	1687.9
		DNNLIDLSGYGAK	46/109	1379.7
		VEVYDGVELNDK	54/109	1378.7
		EDISEYINR	54/109	1137.5
		DSPVGEILTR	79/109	1085.6
		SNSQSINDDIVR*	105/109	1346.6
		KEDYIYLDFFNLNQEWR	39/109	2292.1
		LFLAPISDSDEFYNTIQIK	33/109	2213.1
		KDEESTDEIGLIGIHR*	83/109	1810.9
3	Botulinum neurotoxin type A,	SFGHEVLNLTR	44/49	1272.6
	BoNT/A	GANLSTNFNGQNTEINSR	-	1936.9
	(>WP_012720356.1)	YTMFHYLR*	49/49	1130.6
		YVDVNNVGIR	39/49	1148.6
		LVASNWYNR**	49/49	1122.5
		IPNAGQMQPVK*	35/49	1181.6
		IWVIPER*	45/49	911.6
		GIPFWGGSTIDTELK**	45/49	1619.8
		SFGHEVLNLTR	44/49	1271.7
		FATDPAVTLAHELIHAGHR	44/49	2055.1
		LYGIAINPNR**	45/49	1129.6
		FIDSLQENEFR*	45/49	1396.7
		VNYTIYDGFNLR	44/49	1473.8
		NFTGLFEFYK**	49/49	1264.6

		VNTQIDLIR**	45/49	1070.6
		EALENQAEATK	43/49	1202.6
		NNINFNIDDLSSK*	49/49	1492.7
		RLEDFDASLK	45/49	1192.6
		GTLIGQVDR	44/49	957.5
		VNNTLSTDIPFQLSK	44/49	1675.9
		VNFDPIDK	44/49	946.5
		DLYDNQSNSGILK	48/49	1465.7
		YVDVNNVGIR	39/49	1147.6
4	Botulinum neurotoxin type C,	PITINNFNYSDPVDNK**	8/8	1849.9
	BoNT/C	NILYLDTHLNTLANEPEK**	8/8	2097.0
	(>BAA08418)	ITGNIWVIPDR	-	12872.7
		NSNPNLNKPPR	-	1249.7
		SGYYDPNYLSTDSDKDTFLK	-	2328.0
		EIGEELIYR**	8/8	1120.6
		TGSINPSVIITGPR**	8/8	1414.8
		ENIIDPETSTFK**	8/8	1395.7
		LNSITTANPSSFNK**	8/8	1492.7
		FVVESSGEVTVNR**	-	1421.6
		NKFVELYNELTQIFTEFNYAK	-	2610.3
		SNLNVLFMGQNLSR**	8/8	1591.8
		NTDLPFIGDISDVK	-	1532.8
		KDINEETEVIYYPDNVSVDQVILSK	-	2909.5
		DINEETEVIYYPDNVSVDQVILSK	-	2781.3
		NTSEHGQLDLLYPSIDSESEILPGENQVFYDNR	-	3778.8
		TQNVDYLNSYYYLESQK**	6/8	2127.0
		LSDNVEDFTFTR	-	1442.7
		SIEEALDNSAK	-	1175.6
		VYTYFPTLANK	-	1315.7
		VNAGVQGGLFLMWANDVVEDFTTNILR	-	2978.5
		IITQFNNISYQMYDSLNYQAGAIK	-	2794.3
		YSGSDKENIK**	8/8	1139.6
		ISEAMNNINK**	8/8	1132.6
		VIDELNEFDR	-	1248.6
		LINLIDSHNIILVGEVDK	-	2004.1
		VNNSFQNTIPFNIFSYTNNSLLK	-	2674.3
		DIINEYFNNINDSK	-	1697.8
5	Botulinum neurotoxin type D,	DFNYSDPVNDNDILYLR**	5/5	2072.1
	BoNT/D	AFMITQNIWVIPER**	5/5	1717.1
	(>WP_039241004)	FNSLYSDLTNVMSEVVYSSQYNVK**	5/5	2786.6
		LSSESVVDLFTK**	5/5	1323.8
		IYTFLPSLAEK**	5/5	1280.8
		GVQAGLFLNWANEVVEDFTTNIMK**	5/5	2695.6
		TIENCLEQR**	5/5	1161.6
		VAPEFLLTFSDVTSNQSSAVLGK**	5/5	2409.4
		TKTELINLIDSHNIILVGEVDR**	2/5	2491.6
6	Botulinum neurotoxin type E,	INSFNYNDPVNDR*	195/257	1566.7
	BoNT/E	NIWIIPER*	257/257	1039.6
	(>WP_012451426)	NVIGTTPQDFHPPTSLK*	193/257	1850.9
		INNNLSGGILLEELSK*	193/257	1712.9
		ANPYLGNDNTPDNQFHIGDASAVEIK*	198/257	2799.3
		NNYMPSNHGFGSIAIVTFSPEYSFR*	253/257	2834.3
		DASGIYSVNINK*	195/257	1279.6
		LYSFTEFDLATK*	255/257	1433.7
		LSNLLNDSIYNISEGYNINNLK*	192/257	2510.3
		GQNANLNPR*	192/257	982.5
		EIDDTVTSNNNYENDLDQVILNFNSESAPGLSDEK**	257/257	3898.8
		LNLTIQNDAYIPK**	241/257	1501.8
		VPEGENNVNLTSSIDTALLEQPK*	239/257	2467.2

		IADISIVVPYIGLALNIGNEAQK**	257/257	2410.1
		DALELLGAGILLEFEPELLIPTILVFTIK**	241/257	3179.8
		EVYSFIVSNWMTK**	254/257	1602.7
		EQMYQALQNQVNAIK	208/257	1776.9
		YNSYTLEEK**	257/257	1145.5
		QIENELNQK**	243/257	114.6
		VSIAMNNIDR*	190/257	1131.6
		FLTESSISYLMK**	257/257	1417.7
		LSSYTDDK**	256/257	927.4
		IVNVNNEYTIINCMR**	257/257	1851.9
		VSLNHNEIIWTLQDNAGINQK	249/257	2406.2
		WIFVTITNDR**	257/257	1263.7
		LYINGNLIDQK	194/257	1289.6
		SILNLGNIHVSDNILFK**	257/257	1896.0
		YFNIFDKELDETEIQTLYSNEPNTNILK	190/257	3390.7
		DFWGNYLLYDK**	257/257	1432.7
		DSTLSINNIR**	199/257	1131.6
		KNDQVYINFVASK*	209/257	1524.8
		THLFPLYADTATTNK	189/257	1691.8
7	Haemaglutinin component,	**NNIQTVFTNFTEANQIPIGFEFSK**	28/80	2759.36
	HA70	**AEYYLPSLGYCEVTNAPSPESEVVK**	80/80	2745.29
	(>YP_001253337.1)	**NLMNSSVNIIDNLNSTGAHY YTR**	59/80	2597.24
		**QSPDVHDYISYEFTIPGNFNNK**	36/80	2585.19
		**VNDNAIPYYYPTPSFNEEYIK**	80/80	2537.18
		**QNQILGGSVISNGSTGIVGDLR**	79/80	2185.15
		**DLIGTLLIEAGSSGSIIQPR**	80/80	2040.13
		**VINYSDTIDLADGNYVVR**	28/80	2027.00
		**DAFNVQLFNTSTSLFK**	80/80	1831.92
		**VPQTSSNIENQIQFK**	42/80	1732.88
		**YELIDYQNGSIVNK**	78/80	1655.82
		NLYMYLQYTYIR*	80/80	1640.81
		**LYTSYNQGIGTLFR**	66/80	1632.83
		**NTTRPLFTTSNDTK**	31/80	1595.80
		**AINYITGFDSPNAK**	45/80	1510.75
		**VLPYSNGLYVINK**	80/80	1479.82
		**NFQSGNLCDDDIK**	58/80	1468.63
		**VEVTELNNYNIR**	75/80	1463.74
		AVLYVPSLGYVK*	71/80	1308.75
		CILNEQFLYK*	78/80	1270.65
		**NIPTNNIFNSK**	80/80	1261.65
		**INAQNNLPSLK**	52/80	1211.67
		**FSQQYTEER***	80/80	1187.53
		**DFYFLTNDK**	80/80	1162.54
		**VLQHEIIER***	80/80	1136.64
		**LLNGAIYILK**	80/80	1117.69
		**IYEAIGSGNR**	69/80	1079.54
		**SYLVVLLNK**	80/80	1048.64
		**IYNDIQEK***	72/80	1022.51
		**FVEEAPSDK**	31/80	1021.48
		**SIEFNPGEK**	80/80	1020.49
8	Haemaglutinin component,	**YLSYDNFGFISLDSLSNR**	72/91	2111.00
	HA17	**SLTFSNESSANNQK**	70/91	1526.70
	(>YP_001253338.1)	**SIFSGSLYLNPVSK**	28/91	1511.81
		**IAVNTYIMLSLNK**	75/91	1479.82
		**WNVEYMAENR**	74/91	1311.57
		**TFLPNGNYNIK**	75/91	1280.66
9	Thermolysin metallopeptidase	**LLATVLSAVITFSTVSAVYAAPVGK***	57/85	2478.42
	(>YP_001253958.1)	**YENESGALNESFSDIMGVAVEGK**	85/85	2446.10

		**NQVNEYDLYNYLSRPNYK**	23/85	2293.08
		**TNYHMIYEVEGIPVYYGR***	82/85	2204.04
		**TSLDGLVNIDVTYGNGK**	41/85	1765.89
		**NFVLGEDCWVAGGVMR**	82/85	1752.81
		SLDVVGHELSHGVTNK*	85/85	1691.87
		**ASNANNVIDVQGQSVK**	48/85	1643.83
		IDTVFENGNWK*	70/85	1322.63
		ELYGENSNYVK	63/85	1315.61
		GMNINGFVHVGR	70/85	1300.65
		**FNNTPTLIDTK**	85/85	1263.65
		**IFYTANCYK**	85/85	1122.52
		WDETTNFAK	72/85	1111.50
		FNNSEEITK	83/85	1081.51
		DSSMDSINGR	81/85	1081.45
10	Aminopeptidase 1	**SNNLLPFEENYFHSFGCFYPSK***	59/63	2640.18
	(>CAL81728.1)	NSTLLNFDMIGGSESVPLCIMGSDK	62/63	2628.23
		LFYITIILTLLCFSIQTTLFIK	63/63	2604.51
		NINFNYMFENASDHYFFR	63/63	2329.01
		SFNINSVLNNINLISSNEFK	59/63	2267.16
		DIIFVSFNAEEFGCLGSK	63/63	1975.94
		IINSAFSGNLFYIYYK	63/63	1912.98
		EILIISFILLFVFIK	32/63	1808.13
		GINAITFCDNDTSK	63/63	1498.68
		EAPYNMYIVTNK	12/63	1442.69
		ETELNNVVGYIK	63/63	1378.72
		IICFIPYSIK	46/63	1196.67
		VIDSQGNLVK	55/63	1072.59
		ELSVLCSFK	60/63	1025.53
11	E-cinnamoyl-CoA:R-	GTVPPNVVPGLGDHQAGMFLAAGMAGALYK	71/71	2939.49
	phenyllactate CoA transferase	**YTAPSEGRPLSQEENTTYDLENANK***	71/71	2827.30
	(>YP_001255776.1)	**DDYFVQVCMPPYDVFYDR***	71/71	2271.97
		**EMGYTEEDIQELEK***	64/71	1713.75
		**QSPQIAENTAEVLK***	66/71	1527.80
		**VIELANFIAAPAAGR***	71/71	1512.85
		**NETPNPFIVSYK***	71/71	1408.71
		**MENNANMFSGVK***	39/71	1341.59
		**LVFAQITGYGEK***	71/71	1325.70
		**ETDILLTNWR**	16/71	1260.65
		FFADGGAEVIK	71/71	1153.58
		**IIEEQMVTK**	71/71	1090.58
		**QGLDYETLK***	71/71	1066.54
		GGVSGTLYEK	71/71	1010.51
12	Clostripain	VTIMYYCDADNNLESSLLSDIEEMK	93/96	2896.29
	(>YP_001254422.1)	EFPEITLNSNYEANMGDADTLK	84/96	2472.12
		**NFDPATITNEQLGALFVEEQR***	84/96	2392.17
		GYVNNPNLNLVTLIDR	96/96	1814.97
		VGNWFELLDSWFDK	96/96	1755.83
		GSYDQHLSFYDATK	38/96	1631.73
		SIDNLAINLSNENK	84/96	1544.79
		IDEMVVYSFGGPSK*	85/96	1528.73
		SGGGSSNEDDLTLGGK	92/96	1493.67
		TNGADGGVNHYQW*	87/96	1418.60
		INEGNNFSNETK*	12/96	1366.62
		YVLIMANHGGGAK	96/96	1330.69
		LTWCQDGQDPR*	94/96	1318.58
		TVFGEDFEDAR*	91/96	1285.56
		NGLSIFLPDGDK	93/96	1275.65
13	Flagellin (FlaA)	**ELTVQAANDTNVTVDR***	60/61	1745.86
	(>YP_001255230.1)	**AMTFQIGANSGQTIK***	60/61	1566.79

		**QIATIDNAINSVSK**	44/61	1473.79
		**EVAELQSEINR***	60/61	1287.65
		**SLINGAVSEDAK***	35/61	1203.62
		**ISSQTQFNTK***	44/61	1153.58
		**QMAINTGNNGK**	61/61	1147.55
		**LDDTAANVTK**	43/61	1047.53
14	Myosin-cross-reactive antigen	**IEELAESCSAVPVMMPFITSQFMPR**	30/96	2813.33
	(>CAL83585.1)	SIEDVLSEELLNTDFWTYWR	69/96	2516.19
		FEYGVTVNNVEFSISDDK*	80/96	2062.96
		ITFLEQLDIPGGSLDGEVR*	79/96	2059.07
		**ELAAFVNVTDEELEDK**	64/96	1821.87
		YLLNAGVCLLDGEKPK*	96/96	1732.93
		NIAAQSDEFGKPDK*	75/96	1519.73
15	Acetyl-CoA acetyltransferase	ANVKPEDVDEVIMGNVLQAGLGQNSTR	4/11	2854.43
	(>YP_001255689.1)	DGTVTAGNASGINDGAAALVIMSEEK*	4/11	2491.19
		LGDDDIVVAGGTENMSAAPYLLEK	4/11	2478.20
		IADMDLIEANEAFAAQSLAVAK	4/11	2291.15
		HGLATLCIGGGMGTAIIVER	4/11	1969.03
		QSAVAAGIPVEVPSFTINK	4/11	1928.04
		SVSAIELGSIVIK	4/11	1315.78
		IMGYGPFYATK	11/11	1247.61
		**IVAYGTSGVDPK***	11/11	1206.63
		ANELGIKPLAK*	11/11	1153.69
		EEQDEFSAK*	4/11	1082.46
		AVSLATQLIK	4/11	1043.64
16	Butyrate kinase	YEELNLIVTHMGGGASVGTHEK	88/88	2342.14
	(>YP_001255907.1)	IGVQGQHASNLGGIIANEIGK	79/88	2076.11
		NLNIPAFIVDPVVVDEMK	88/88	2013.07
		IGVYEDENQILEETLR	88/88	1920.95
		FPGEDELLALAQGGLR	75/88	1685.88
		NFDVNELDAIVGR	54/88	1461.73
		GGVVAYLNTNDFR	88/88	1425.71
		VDAILLTGGIAYSK	88/88	1420.80
		**GMVSFIAPVVR**	61/88	1175.66
		AGGLPVGDLVK	71/88	1025.59
17	3-hydroxybutyryl-CoA	ICKPETIFATNTSSLSITTIASSTNRPDK*	70/92	3096.58
	dehydrogenase	MLIPMINEAIGIYAEGIATVEDIDLAMK	89/92	3034.55
	(>YP_001255690.1)	VFILGGGTMGAGIVEVFAK	78/92	1866.01
		EPVEVEEAPGFVVNR	92/92	1670.83
		ITVTTDMDLAK	87/92	1207.62
		YRPHPLLIK	92/92	1136.69
		DADLVVEASK	80/92	1046.53
18	Glycosyl hydrolase	**IYENGVLISEADLTANTPQAQTHSVK**	34/34	2799.41
	(>YP_001255332.1)	IALFDEWAATGIDFGDGWDSPYK*	32/34	2574.18
		EGDTVDNPNDQGTPLADESEK	30/34	2230.95
		**NNDNYGNYDINISIPTDSR***	22/34	2184.97
		IVAYFTEWSVYGGHNNYK*	26/34	2148.01
		**AYVGCILSLFMLFSIIPLK***	26/34	2128.19
		DNNYGGIIFWELAGDAPLK*	34/34	2093.03
		**LYENGVEILTENIDGVNGK**	28/34	2077.04
		ELPGLFATATGGANGTWDGGR*	28/34	2047.98
		EGSYTYTAELVNEYGSSK*	34/34	1997.89
		VLISIGGWSQSAGFHNVAK	32/34	1971.04
		WDLDGADIDWEYPTFK*	34/34	1970.88
		**LYENDQVILEEDISK**	28/34	1807.89
		GEMYTYEDETSLGEK*	34/34	1751.73
		**YTYYAELINGAGITR**	28/34	1704.85
		ISMEVLNDNNGNELK*	34/34	1689.81
		**SDDLIVNVTESNN**	24/34	1419.65

		VTHINYAFATIK*	32/34	1377.75
		NPYDNHEDIVK*	26/34	1343.62
		GFFGDGGIPSDDK*	30/34	1311.58
		**VFADSVVEFIR***	34/34	1281.68
		YYELTAAVGCGK	28/34	1274.60
		AAGCNPYHYIK	32/34	1236.58
		NYYNVDTAMK*	26/34	1218.54
		LVVGSPYYSR*	34/34	1140.60
		VPYLYSESK*	34/34	1085.55
		GSSLTDVIYK*	34/34	1082.57
		**LFETYGIAK***	26/34	1041.56
19	Chitinase	VTDLQWDSLTHIQYSFAMVDQATNK*	100/102	2911.39
	(>YP_001253269.1)	SGNWQNINAGATVNLQGMIGLCFSDVR*	88/102	2865.37
		GFYTMLDTDEGINTFADSCVDFIK*	43/102	2702.20
		LIGYFPEWAYNSEAQGYFK*	97/102	2283.07
		LEPAGANPLWHVLNLMEK*	100/102	2032.06
		ETASQIMPTLCMDWAYR*	100/102	2015.90
		DYILSAAVTASPWVLGGVK*	96/102	1947.05
		QYPDVNLLISVGGWAGSR*	102/102	1931.99
		LVAGIMAATFSLSLLTTK	33/102	1837.05
		GWENVQGGQNGLHGSSR*	102/102	1782.82
		YNIWGDDLDNDGK*	102/102	1524.66
		VPYVWQNQER*	100/102	1318.65
		LSNGLTNMGACSK*	87/102	1295.60
		**FNDMKPVGDQK***	37/102	1278.61
		GGWEVSFDLPK	69/102	1234.61
		ILMGIPYYTR*	102/102	1226.66
		DNGDFTTVTIK*	102/102	1210.59
		GHFNLLQTMK*	102/102	1188.61
		VELDPNLPYK*	96/102	1187.63
		VFLSFENER*	100/102	1140.56
		SSWGGNYTLK*	102/102	1112.53
		HAALEEEFK*	102/102	1073.52
		VYWDDVEK*	100/102	1053.48
		YTFGDTLTK	74/102	1045.52
20	Serine	NTIPFETLSPFITSGIR*	79/101	1893.01
	hydroxymethyltransferase	IAYFMNYSIEHR	101/101	1543.73
	(>YP_001255091.1)	**NTDPELLDMIK***	73/101	1288.64
		MIVSGASAYPR	93/101	1151.58
		EYMQQVVK	73/101	1024.51
21	Molecular chaperone DnaK	DVLLLDVTPLTLGIETFGGVSTTLIEK	95/167	2844.58
	(>YP_001255452.1)	IELSSATQTNINLPFITADATGPK	105/167	2502.30
		SQVFSTAADGQTSVEIHVVQGER	105/167	2445.20
		GVNPDEVVAMGAAIQAGVLTGEVK	105/167	2325.21
		**VNIDSTEYTPQQISAMVLQK**	87/167	2265.14
		EANITITASTNLTDDEIEK	95/167	2078.01
		ATEDLTQTFYGISSK	103/167	1660.80
		SLEGSGYAMSDIDK	95/167	1472.65
		NNADQIVYQTEK*	88/167	1422.68
		FNELTHDLVQR*	91/167	1371.70
		LIDYIAETFK*	105/167	1212.65
22	Phosphopyruvate hydratase	HADNNVDLQEFMIMPAGAPSFSEALR	23/38	2860.33
	(>YP_001252774.1)	**AVDNVNTIIADELVGMNVLDQVAIDK***	14/38	2769.43
		**AGYTPGEDIFIALDPASSEIFEDGK**	7/38	2642.25
		LNQIGTLTETLNAIEMAER*	24/38	2117.09
		VLTPEEMANYYVELAEK	24/38	1998.97
		AAANSLGISLYQYIGGVNGK	2/38	1996.04
		AQGYDTGVGDEGGFAPNLK	15/38	1895.87
		SNEEAIVVIIEAIK	24/38	1527.86

		IEEELNDMGEYR	24/38	1497.65
		LGANAMLGVSLACAK	23/38	1418.74
		MCSEVYHALK*	25/38	1180.54
23	Molecular chaperone GroEL	SALQNAASIASTFLTTEAAVADIPE	92/119	2619.35
	(>YP_001255782.1)	EMLQDIAILTGGEVISEELGR	92/119	2273.16
		LLIISEDIEGEALSTLVLNK	80/119	2170.22
		AAVEEGIVPGGGTAYIDIIPK	92/118	2070.11
		SMGTDLEVVEGMQFDR	79/118	1813.80
		MEAVLDDVYILITDK	91/118	1737.89
		ENTPPMAPGMGMDGMY	91/118	1698.66
		QIANNAGAEGSVIIEK	80/118	1613.84
		DVTIDMLGTADSVK	79/118	1464.72
		**GYVSAYMVTDTEK**	79/118	1463.67
		ATEAGVGYDALNDK	56/118	1423.66
		IADLTSDIIDVK	79/118	1302.71
		NVTAGANPIQIR*	91/118	1253.69
		VAAISAASEEVGK	91/118	1231.65
24	Rubrerythrin	EDLQGDAIEITASYPIALYDNTADNLK	55/83	2953.43
	(>YP_001253845.1)	EGFIQISNIFIETAEQER	78/83	2124.06
		**AAASGENEEWTELYPSFAK***	63/83	2099.95
		EEGFPEVAVAYDMISK	40/83	1784.84
		CSNCGFIYEGTAAPEK	68/83	1689.72
		SYFEIACENY	83/83	1238.50
		LLENVENDK	83/83	1073.54
25	Ornithine carbamoyltransferase	GCDVLYTDVWVSMGEPDSVWESK	94/94	2602.14
	(>YP_001255089.1)	FMHCLPAFHDEETAVGK	94/94	1931.87
		YGLSEMEVSHELFESK	94/94	1884.86
		**DQGAHVTYLGPTGSHIGK***	94/94	1837.91
		EHFNKPLNEIK	94/94	1368.72
		NFLTLMDFTPK	94/94	1326.67
		**VTITDNIEEGVK***	91/94	1317.68
		EINYFLDLAR	94/94	1253.65
		EIPTDAALVAK	71/94	1127.63
		AVMVATLGDQ	91/94	1004.50

* Unique to the species with one exception. Peptides in bold letters are unique to the target species.

Supplementary Table S4-H: Inclusion list of unique and abundant peptides from selected protein markers of *Clostridium perfringens*.

S. No.	Protein	Peptide	Strain coverage	Theoretical mass (Da)
1	Electron transfer flavoprotein	**NIFETSDDEILVWSADDIDVDK**	28/28	2539.17
	(>ABG84135.1)	**DNNGAEVTVLSMGPPQAK**	28/28	1827.89
		LEYDIIFAGR	28/28	1196.63
		**VEAPVLLTAIK**	14/28	1153.71
		VEEDGLLVNR	28/28	1143.60
2	Dipeptidase PepV	GINAIMQTIK	28/35	1088.57
	(>ABG82974.1)	FGILLQDEASGK	33/35	1277.63
		VFGVSAHGSTPEK	35/35	1315.62
		DIEYYLEHEK	35/35	1338.58
		LSFNVGVIDLNDK	33/35	1433.71
		TLDDMMTPFNER	35/35	1469.59
		FEVSHKNDEIELK	33/35	1587.75
		FLNDNIGEDVYGEK	35/35	1612.69
		LSFNVGVIDLNDKVGR	33/35	1745.89
		GTTDDKGPIMASLYGLK	35/35	1766.83
		APVAGFTPDAEFPIINGEK	26/35	1973.92
		IDEMKDDLIESVQNIIR	31/35	2030.96
		GTGIEIENFEHQKPLYFSPDHPLIK	31/35	2909.35
3	Rubrerythrin	AETAHYNR	33/33	961.42
	(>ABG84689.1)	YTYFSSTAR	33/33	1095.47
		CGNCGFIWEGAEAPLK	33/33	1808.74
		CPACLHPQAYFEVFK	29/33	1866.85
		CPACLHPQAYFEVFKETY	29/33	2259.99
		FLKDDLQGEAVEINAAYPVELPTDTLTNLK	32/33	3317.63
		FAADGEHDELSNLYPSFADVADEEGFPEVAAAFR	27/33	3686.55
		FIEQGEDLAFADEHR	0/33	1776.77
		QADREGFPEVAEAYQR	0/33	1865.86
4	Fructose-bisphosphate aldolase	YPANWAGLNFEALANIK	-	1891.91
	(>ABG82556.1)	AATGDMPLVLHGGTGIPSDMIAEAISLGVSK	27/27	3008.43
		**AATGDMPLVLHGGTGIPSDM IAEAISLGVSK**	27/27	3008.54
		**NVSVEAEVGSIGGEEDGVVG AGEIADPAECK**	27/27	2987.37
		QIAELGVTMLAAGIGNIHGK	27/27	1993.08
		**YPANWAGLNFEALANIK**	27/27	1891.97
5	Glutaredoxin	SVPVAVIGEEVIVGFDK	27/27	1757.93
	(>ABG83947.1)	**LGVQYETITVADGK**	27/27	1493.78
		**NEVLEVSGQR**	27/27	1130.58
6	Hypothetical protein CPE0268	YPAQQQAMLASDEGKR	8/9	1792.82
	(>BAB79974.1)	AFEDVKEQVVNMLIQER	8/9	2047.99
		VLATVGNTEITSDYIDEIIAR	9/9	2293.14
7	Maltose ABC transporter	GYQFVQDLVQK	-	1324.62
	(>ABG84625.1)	KSGFYISGPWDVSAFK	-	1788.77
		**FEIASAISIIIFLILGTISYYQMK**	35/35	2734.51
		MATQFAGYTDILASVNYK	29/35	1992.97
		FVGFANFIEVLTGPFK	32/35	1785.95
		LSGQFEEVE	-	1037.47
8	Hemeoxygenase	FINEVANSYVYNIAISNELDFIR	30/31	2704.36
	(>ABG84805.1)	INEIGETAPELLVAHAYTR	31/31	2097.09
		FLLGENLNTMKPLASTR	26/31	1905.02
		LNNIELNEEMK	31/31	1346.66
		NNSNDLHAVAEK	31/31	1311.62
		YETLSDGPEMK	27/31	1269.56
		IDEEGLNYYK	31/31	1243.58
		DFVLPEIYR	31/31	1151.60

		MNSFMMDIK	24/31	1116.48
		GFVMNYHNK	28/31	1109.51
9	Metallopeptidase, family M24	AHFLIQLLDR	-	1225.65
	(>ABG83666.1)	IVAYHEVGHALVAALLNNTD PVHK	40/40	2581.38
		VPFFSMSGSDFVEMFVGMGAAR	40/40	2369.07
		YIVIYSIIAFGILLTFNMVK	40/40	2318.32
		FVIQEDLDEAVEVIIAGQEK	40/40	2245.15
		**SVNFSGNQIEITPSDSSNLK**	40/40	2137.04
		**AAEEVVFNSITTGASNDIER**	40/40	2123.02
		**ILYTTNPAVAGITQPELIK**	25/40	2042.15
		**NCSETTAAIADEEVLQVIK**	40/40	2034.00
		STPGAVGADLANIVNEAALR	40/40	1939.02
		ETIMGDEFMEIVYGK	34/40	1761.80
		**ESLVEIVDFLHDTR**	38/40	1672.85
		**EEMIDQISVMLGGR**	40/40	1577.76
		VDYSTFMQMLDK	40/40	1477.67
		ANNEANNDALDSSK	28/40	1462.64
		ELLDEITGVLLDK	40/40	1457.80
		FDMMALEAMSNR	40/40	1415.61
		LYAENETGITFK	40/40	1385.69
		**LSDDVSLEEIAK**	40/40	1318.67
		**NMITIYGMSER**	40/40	1314.61
		**AMIEAQEEAAK**	38/40	1190.57
		**SNVNDGATEEK**	32/40	1163.51
		DGAIQGNDER	40/40	1074.48
10	DnaK-type molecular chaperone hsp70	AMEDLTQAFYK	34/34	1316.56
	(>ABG85010.1)	SGNVSLNDIDKVILVGGSTR	34/34	2044.00
		IIGIDLGTTNSCVAVMEGGE PVVITNSEGAR	34/34	3102.54
		**IELSSSTQTLINLPFITADATGPK**	34/34	2517.34
		GVNPDECVAMGAAIQAGVLTGDVK	34/34	2315.13
		**EANITITASTNLSDAEIDK**	34/34	2005.99
		**GIPQIEVAFDIDANGIVK**	32/34	1899.02
		NNAEQTVYQTEK	28/34	1424.66
		**AMEDLTQAFYK**	34/34	1316.61
		IIDYIAEDFK	34/34	1226.63
		SGNVSLNDIDK	34/34	1161.57
11	Peptidyl-prolyl cis-trans isomerase	IKIELYPEIAPNTVR	32/32	1755.97
	(>ABG82378.1)	**IIPGFMIQGGCPEGTGMGNPGYR**	32/32	2352.09
		**TDYNDMPDEPQVMAK**	32/32	1753.74
		VTVENAEGIEEPEIL	30/32	1641.82
		MNPIVTIEMATGEK	30/32	1533.76
		IELYPEIAPNTVR	32/32	1514.82
12	Choloylglycine hydrolase family	GLAGGLLFLK	37/39	988.59
	protein	**QGLIAINSGEIVTWILSNFESVSDIK**	35/39	2833.49
	(>ABG84479.1)	**TVITPISNGTGLLGLPGDYTSPSR**	13/39	2416.27
		**YAFLGINLCGSTLFFDGVNEK**	33/39	2308.13
		**MCTHIHISSVNNNLYWGR**	15/39	2145.01
		ESDFSSYMCAYDQSLGK	13/39	1930.78
		**MGESVVLEPTNHGHFR**	33/39	1809.87
		**VVVTSDDIPSLGELGK***	33/39	1628.87
		**TLDTSFNPFEVGSK**	17/39	1541.74
		**NLIGDITDEEAPAR**	33/39	1513.74
		**NFTLETQSKPWK**	33/39	1478.76
		**ENELVTYSI***	15/39	1067.52
13	Glucose-6-phosphate isomerase	**AIEILVNYEPSVHYFNEWWK**	33/33	2537.24
	(>ABG82971.1)	**LHNGTGAGNDFLGWVDLPVNYDK**	33/33	2502.20
		**ACGISGYVLGINPFDQPGVEAYK**	33/33	2398.17
		**EEIVIEANDENIDGLNFLAGK**	33/33	2303.14

		**TPAIFYAGNNISSSYMADLLK**	33/33	2276.12
		**SHEVAYMQETINQAHNK**	33/33	1999.92
		**AAIEMLTNNFYNSMSK**	25/33	1833.85
		**EEYANPSLADNECYK**	33/33	1745.73
		**DIFETVINVGSPR**	26/33	1446.75
		**AIDGLDVSLNVISK**	33/33	1443.80
14	Triosephosphate isomerase	AAFAHNLTPILCCGETLEQR	30/33	2187.06
	(>ABG82634.1)	**MLEAMNIDYVIIGHSER***	33/33	1990.97
		**IQYGGSVKPNTIAEQMAK**	33/33	1935.00
		**CEVVVCPTFVCLDAVK**	33/33	1724.84
		TATSDQANETIAAIR	33/33	1561.78
		**AMVAEMFGQEVADK**	33/33	1525.70
		**AQITADLEGLTK***	33/33	1259.68
		**MHYTIDEAVK**	33/33	1206.58
		**ENGTTNDVIK**	33/33	1090.53
15	Elongation factor Tu	**TTLTAAITTVLAQAGGAEAFK**	35/35	2035.10
	(>ABG83459.1)	**GSALVALENPTDEAATACIR***	35/35	2001.99
		**GVLHVGDEVEVIGLTEER***	35/35	1951.01
		**GQVLAQVGTINPHK***	35/35	1461.81
		TVGSGVVTSIIE	35/35	1161.63
16	Hypothetical protein CPE0689	IIEGLQANFTGECTEVGMYLAMSR*	9/34	2633.24
	(>BAB80395.1)	FVCTICGYVYEGEK*	9/34	1610.72
17	Ornithine carbamoyl transferase	GADVIYTDIWVSMGEDESLYPER	28/31	2645.20
	(>ABG84847.1)	CAFECGAAEEGAHVTFLTNSQMGK	31/31	2501.08
		NTLFMHCLPSFHDEDTEVCK	31/31	2366.02
		**MGMHFVALGPDSLKPDEDILK**	31/31	2313.16
		**ETGATIEFSSNVDEAVK**	28/31	1796.85
		QSTVEELAK	31/31	1004.52
18	Clostripain-like protease (Clp)	FALPNAEIMIHQPLGGFQGQATDIDHAK	-	3132.59
	(Q0TQK21)	**DYFMEASEAVEYGLIDK**	80/80	1979.89
		**LNQILSENTNQPLEK**	80/80	1740.91
		**MSNLVPMVVEQTSK**	80/80	1562.79
19	Thioredoxin	**MIAPVIEELANEMENVK**	52/52	1929.96
	(A0A0H2YNW2)	VVDTLVGFRPK	52/52	1230.72
20	ABC transporter	**AIAMNPEIILMDEPTSALDPISTLK**	74/74	2683.39
	(Q0TTG6)	**TAFFLNGELVEFSDTNTIFTNPR**	74/74	2633.28
		**DLDLFYASNHALK**	74/74	1506.75
		**IEGEIQVDGK**	74/74	1087.56
21	Purine nucleoside phosphorylase	**DYTPGDLMIISDHLNLSGSNPLIGK**	49/49	2670.34
	(A0A0H2YR30)	**ILGADAVGMSTVPEVIIANHSGMK**	49/49	2410.24
		**FHYYEGYSMQEVTCPVR**	49/49	2108.92
		**EGVYAMFSGPTYETPAEVR**	49/49	2103.96
		**LLGVETLVVTNAAGAVNK**	49/49	1769.01
		**TFIELMTNIIK**	49/49	1322.73

* Unique to the taxon with one exception. Peptides in bold letters are unique to the target species.

Supplementary Table S4-I: Inclusion list of unique and abundant peptides from selected protein markers of *Clostridium tetani*.

S. No.	Protein	Peptide	Strain coverage	Theoretical mass (Da)
1	Tetanus neurotoxin,	**SNAASTIEIHNIDDNTIYQYLYAQK**	21/21	2885.39
	TeNT	**LSSANLYINGVLMGSAEITGLGAIR***	17/21	2520.34
	(>AAO37454)	**AMDIEYNDMFNNFTVSFWLR**	21/21	2513.12
		**FDTNSNSVSFNLLEQDPSGATTK***	14/21	2472.15
		**IPNLLDDTIYNDTEGFNIESK***	21/21	2411.16
		VSASHLEQYGTNEYSIISSMK	20/21	2344.11
		**FQILYNSIMYGFTEIELGK***	21/21	2266.14
		**YSDPVNNDTIIMMEPPYCK**	20/21	2229.98
		**NSFSEEPFQDEIVSYNTK**	15/21	2133.96
		**ILGCDWYFVPTDEGWTND***	16/21	2130.91
		**NITDYMYLTNAPSYTNGK**	21/21	2065.95
		**VNQGAQGILFLQWVR**	21/21	2034.01
		**NASLGLVGTHNGQIGNDPNR***	16/21	2032.95
		**NLDCWVDNEEDIDVILK***	21/21	2011.92
		**LSQVTSCNDPNIDIDSYK***	21/21	1999.14
		**ISDVSTIVPYIGPALNIVK**	17/21	1909.94
		**LYVSYNNNEHIVGYPK***	21/21	1894.03
		**IINAIPYLGNSYSLLDK***	21/21	1885.06
		**SAMLTNLIIFGPGPVLNK***	20/21	1788.96
		**AIHLVNNESSEVIVHK***	21/21	1735.84
		**YDTEYYLIPVASSSK**	20/21	1733.90
		**DILIASNWYFNHLK***	21/21	1728.95
		**ITMTNSVDDALINSTK**	17/21	1722.85
		**DIIDDFTNESSQK**	21/21	1511.68
		**LYTSYLSITFLR**	21/21	1476.80
		**HSLSIGSGWSVSLK**	21/21	1457.77
		**NKPLNFNYSLDK**	21/21	1452.74
		**LSYFSMNHDPVK***	18/21	1437.68
		**YTPNNEIDSFVK***	18/21	1426.68
		**TASLTDLGGELCIK**	20/21	1420.73
		**CNNNNQYVSIDK**	21/21	1411.62
		**SFLVNQMINEAK**	18/21	1393.71
		**DSNGQYIVNEDK**	21/21	1381.62
		**VFSTPIPFSYSK**	21/21	1372.71
		**WLGTVNTQFQK**	21/21	1321.68
		**IYSYFPSVISK**	21/21	1303.69
		**VGYNAPGIPLYK**	21/21	1291.70
		**EQIADEINNLK**	21/21	1286.65
		**WVFITITNDR**	21/21	1264.66
		**AMININIFMR**	19/21	1222.64
		**QLLEFDTQSK**	21/21	1208.61
		**IIVDYNLQSK**	12/21	1192.65
		**NEDLTFIAEK**	20/21	1179.58
		**SLEYQVDAIK**	21/21	1165.60
		**NNVAGEALLDK**	21/21	1143.60
		**DGNAFNNLDR**	21/21	1135.51
		**MPITINNFR**	20/21	1105.58
		**GNNLIWTLK**	21/21	1058.59
		**GIPYAPEYK**	15/21	1037.53
		**NILMQYIK**	21/21	1022.57
		DFWGNPLR	21/21	1004.49
2	Putative S-layer protein	VVVYVGNVK	-	976.56

	(>AAO35101)	GVPVPTFEYVK	-	1235.62
		DSLLVVFSEPVSR	-	1447.72
		SVNDYVYILYSR	-	1491.68
		GGYAFTALIELPGYSK	-	1686.80
		FEAINDGVDTTVETLR	-	1779.80
		YNVFYGENGNKDQWK	-	1862.77
		QVTQDADITAEDVVLYR	-	1935.89
		NILIDNQYNYYHVLAPK	-	2078.02
		NVYIVGGEGVVKPSIESTLK	-	2089.06
3	Electron transfer	NVMDIGEAEFLVAGGR	-	1693.77
	flavoprotein	MLEELAELLGGNVAGSR	-	1758.85
	(>AAO35314)	VIYAQNPLLSHYTTDGYTK	-	2184.02
		VTVITMGPQQAESTLR	14/14	1730.84
		TWNADFLEVDK	14/14	1337.59
		VTVITMGPQQAESTLR	14/ 14	1730.84
		AFAGADTLATATALSAAIK	14/14	1763.88
4	Acetyl-CoA	AGDADVVIAGGMENMSAAPYVLPNAR	13/13	2589.13
	acetyltransferase	**AGIKPEIIDEVIMGNVIQAGLGQSPGR***	13/13	2762.48
	(>AAO34954)	**AGDADVVIAGGMENMSAAPYVLPNAR**	13/13	2589.24
		DGLWESFNDYHMGMTAENIAEK	13/13	2558.09
		DGTVTAGNASGINDGAAALVIMSAEK	13/13	2433.19
		VEDLDLIEANEAFASQSLAVAK	13/13	2333.18
		GLDPAYMGYGPVGATK	13/13	1596.77
		EMQDEFACASQNK	10/13	1500.60
		AGIPVEVPAFTLNK	13/13	1455.82
		DVPAVELGATVIK	13/13	1311.75
		FGATVESLAK	10/13	1022.55
5	Molecular chaperone	ADAEAYLGGTVTQAVITVPAYFNDSQR	15/15	2857.40
	DnaK	IELSSSTQTNINLPFITADATGPK	15/15	2518.30
	(>AAO36534)	SQVFSTAADGQTSVEIHVVQGER	15/15	2445.20
		DVNPDECVAMGAAVQAGVLTGDVK	15/15	2359.12
		EANITITASTNLSDDEVEK	15/15	2049.98
		**FNELTQDLVEGTLTPMK**	12/15	1935.97
		ATEDLTQAFYQISSK	15/15	1701.83
		TTPSVVSFQDNGER	15/15	1536.72
		**ALQDAEMSIGEIDK**	15/15	1519.73
		NNAEQIFYQTEK	15/15	1484.70
		IIDHIAETFK	15/15	1186.64
6	60 kDa chaperonin	SALQNAASVASTFLTTEAAIADIPEK	12/12	2619.35
	GroEL	**EMLEDIATLTGGQVISEEIGR**	12/12	2261.13
	(>AAO36881)	**HSEAGIGYDALNNEYVNMIK**	12/12	2238.04
		ITNIQEILPVLEQIVQQGK (Filifactor alocis)	12/12	2163.23
		AAVEEGIIPGGGTAYAMVIK	9/12	1947.02
		MEASLDDAYILITDK	12/12	1697.83
		**NDTPMPGAPGMMDGMY**	12/12	1684.64
		**QIACNAGVEGSIVIEK**	12/12	1630.84
		NVTGGANPMLVR	12/12	1228.64
		GIQMAVEEAVK	12/12	1174.61
		**LNSETHDIK**	9/13	1056.53
		SIMFGEDAR	13/13	1025.47
7	Butyrate kinase	**FIAPVVLYPGEDELLALTQG GLR**	14/14	2471.35
	(>AAO37005)	**SLNIPAFIVDPVVVDELQDIAR**	11/14	2423.31
		**SYEDVNIVVVHMGGGSSVGAHK**	14/14	2242.09
		**CSVVLEGNVDAIVLTGGIAYSK**	14/14	2208.15
		**GGLLKPVEGGTYEVNDAMLK***	11/14	2091.07
		**IGVFEDENLIFEETLR**	11/14	1923.96
		GEHASNLGGIIGNEIAK	14/14	1679.87
		YSNVYEQFPFR	14/14	1449.67
		LILDAFIYQISK	14/14	1423.81

		GFDINTLDAIVGR	11/14	1390.73
		GGAVAYLGTNDFR	11/14	1340.65
		SGGVPIGDLVR	14/14	1069.60
8	Adenosyl transferase	-		
	(>AAO34962)			
9	Methyl aspartate	GAGHDGFTYTGEPVTEGFTQIR	14/14	2339.99
	ammonia lyase	**ENYTPIFHIDVYGTIGAAFDCDIK**	9/14	2702.28
	(>AAO37022)	DEYNPGGEIAPVPVFTQSGDDR	12/14	2363.07
		**GAGHDGFTYTGEPVTEGFTQIR**	14/14	2340.08
		IVDVLCTPGFTGFYFDDQR	14/14	2193.03
		TPDLGGVNNAAEAIMYCK	14/14	1866.87
		VELVADEWCNTVEDVK	14/14	1848.86
		EADVLPHALINNVEEK	14/14	1790.92
		ANGMGAYCGGTCNETNR	14/14	1718.66
		DPLFLASDFIPVIEK	14/14	1703.92
		AMADYLQTLEK	14/14	1282.63
10	Elongation factor Tu	ADQVDDAELIELVEMEVR	7/12	2089.94
	(>AAO37057)	ELMNEYGFPGDDAPVVVGSALK	12/12	2308.11
		TTDVTGSIALPEGVEMVMPGDHIDMK	12/12	2743.29
		ELMNEYGFPGDDAPVVVGSALK	12/12	2308.11
		ATDKPFLMPVEDIFTITGR	12/12	2151.11
		**ADQVDDAELIELVEMEVR**	7/12	2074.00
		**VGDEIEIVGLSDESK***	7/12	1589.79
		TTLTAAITTILGHK	7/12	1440.84
		**GQVLAATGSVKPHK**	7/12	1392.79
		**TVGSGVVSEITE**	12/12	1177.59
		SFTGQVYVLK	7/12	1141.62
11	Methionine gamma lyase	**LANLAVSLGDAETLIQHPASMTHSPYTK**	15/15	2965.50
	(>AAO36990)	**DTQYGALSTPIYQTSTFIFDSAEQGGK**	15/15	2925.37
		**MAILEGGEAVIATASGMGAISSALWTALK**	9/15	2819.46
		**AGDHVVAGETLYGCTFALLCHGLPR**	12/15	2600.27
		**FGVEVTFVDTTNLEEVEK**	12/15	2056.01
		**VYYPGLNDSEYYDIAK**	12/15	1909.88
		**DMTGSVLSPFDAFLVLR**	15/15	1867.96
		**MPGAMISFEVNGGLEEGK**	15/15	1865.87
		LSVGLENVEDIIEDLR	15/15	1813.95
		**VAEFLQGHEAVSK**	15/15	1414.73
		**FAGEESGHIYTR**	15/15	1366.63
		**LGNPTTGQLEDK**	15/15	1272.64
		**EAAGISDNLVR**	15/15	1144.59
		**QEFINNVR***	15/15	1019.52
12	Pyruvate-flavodoxin	**GQGPAWGNSLFEDNAEYGFGMYLGDK**	16/16	2823.23
	oxidoreductase	**MMIANATGCSSIWGGSAPSIPYTTNNK**	12/16	2772.27
	(>AAO35733)	**DDVTNTSLSPEEFVDTTPEGTISCK**	12/16	2685.20
		**HIIVAMGSICDTVEETIDYLMSK**	16/16	2568.23
		**EAEQYNGPSLIIAYSPCISHGIK**	16/16	2490.23
		**IQVSPLDCTGCGNCADVCPALGK**	8/16	2264.01
		**YIAENNINFYTIDAIDIAR***	12/16	2229.11
		**FYDEVPDTVENYMNEINK**	12/16	2219.98
		**RPLLEFHGACPGCGETAYVK**	16/16	2148.03
		**IAGLMAELIQEDIPQNMK**	12/16	2014.03
		**SPYLVYNADYIACHNR***	16/16	1898.88
		**VLQILNNHGYENNPK**	16/16	1752.90
		IVQMNYTAVDEGVQK	16/16	1694.84
		**QYHPFDYYGAPDAK**	16/16	1671.74
		**EPGSLSEPLYLDISK**	16/16	1647.84
		**MEDGTFPVGTTAYEK**	12/16	1645.74
		**NGGNFVLNCPWNER**	12/16	1619.73
		YTSLANVFPEAAEK	16/16	1539.76

		**GIAVMVPQWQIEK**	16/16	1498.80
		GTAENPDIYFQGR	16/16	1467.68
		**QEGDDLPVSSFLK**	14/16	1434.71
		**FWGLGSDGTVGANK***	16/16	1408.68
		**AVLSGYWHLYR**	14/16	1364.71
		**DTTPSDIIAIFR**	14/16	1348.71
		**IETIDYEDIAK**	14/16	1309.65
		**IANVIPIEDAIK**	16/16	1295.75
		**VHLYRPFSEK***	16/16	1275.68
		**SLNPERPTLR**	16/16	1182.65
		NELTVPDFVK	16/16	1161.61
		**TTIMDPHTVK**	14/16	1142.58
		SGMGSSVAEEK	16/16	1081.48
		**QNKPVIVGGR**	16/16	1067.63
13	3-hydroxybutyryl-CoA	ELIYTADIIGAEEALR	2/5	1776.89
	dehydratase	IAVLTINRPDALNALNSELLK	2/5	2278.22
	(>AAO36889)	ELIFTTDMIDANEAYR	1/5	1901.89
		**SLNALNSETLNELGAAIK**	1/5	1857.99
		YEADIFGLCFATEDQK	3/5	1849.83
		**AFVAGADITEMQNLNAK**	1/5	1792.88
		**AAINDGMNMDTESAYK**	3/5	1730.73
		**ENGIVEITINRPK**	1/5	1482.82
		**VYEADELIDK**	1/5	1194.58
14	Rubrerythrin	VADEEGFPTIATVFR	-	1651.79
	(>AAO35755)	YAADGENEEWSELYPEFAR	-	2275.90
		**FYICPVCGFTIEVESLNEVPDK**	9/13	2502.19
		EQVGDFTVPAGAVFGK	13/13	1621.82
		SAYGGESMAHMR	13/13	1296.54
		VHASNHFTVLK	13/13	1252.67
		SFHYAVEAEK	13/13	1180.56
		LFEAVAYAER	13/13	1168.59
		YLVWGESAK	13/13	1052.54
15	3-hydroxybutyryl-CoA	MAQEDMDSILGR	12/12	1365.56
	dehydrogenase	**ICKPETILSSNTSSLSITEIATATNRPDK**	12/12	3090.59
	(>AAO36889)	**ILIPMINEAIGIYAEGIATAEDIDK**	12/12	2673.40
		AADCDLVVEAAIENMEIK	12/12	1933.92
		ICVLGAGTMGAGIAQAFAAK	12/12	1849.96
		DPVEVAEAPGFVVNR	12/12	1598.81
		GMATSQETFDAVK	12/12	1384.64
		**MAQEDMDSILGR**	12/12	1365.61

* Unique to the species with one exception. Peptides in bold letters are unique to the target species.

Supplementary Table S5: Results from proof-of-concept studies.

Supplementary Table S5: Verification exercise using blind samples; number coded soil or sand samples were spiked with one or two bacterial select agents and subjected to directed and shotgun analysis using tandem mass spectrometry after extraction of proteins. Abundant proteins from Coomassie-stained SDS-PAGE were excised and subjected to in gel digestion and identification by tandem mass spectrometry as described in the text.

Protein identified	MS* (MP)	Mascot score	Peptide (MS/MS)	m/z	Ion score	RMS error	Agent identified	Agent spiked
**SHOT-GUN ANALYSIS**								
**A (Sand)**								
**SAP, S-layer protein**	12	248	APVLDQYGK	990.45	39	72	*Bacillus anthracis*	*C. perfringens*
			GMFEPGKELTR	1264.54	44		*Bacillus cereus*	*B. anthracis*
			VSAEGAAVASISNWTVAEQNK	2131.91	61		*Bacillus thuringiensis*	
			TFPDVPADHWGIDSINYLVEK	2415.99	95			
**Formate acetyltransferase**	20	927	QMQFFGAR	984.41	43	58	*Bacillus anthracis*	
			SLQPYGGIR	990.47	39		*Bacillus thuringiensis*	
			NAWENFKGEK	1222.51	57		*Bacillus cereus*	
			SGVITGLPDAYGR	1305.60	49			
			TSTFLDIYIER	1357.61	74			
			IEMALHDTNVLR	1411.65	71			
			KSGVITGLPDAYGR	1433.69	30			
			EGHHLNINVFNR	1449.65	82			
			TGEPFAPGANPMHGR	1538.62	64			
			TSAIQYENDDIMR	1555.61	48			
			THNQGVFDAYTPEMR	1765.68	92			
			YGNNDDRVDEIAVNLVK	1933.83	53			
			VKPIRDENGIAVDFEIEGDFPK	2488.09	61			
**Chaperonin GroEL**	14	738	IEDALNSTR	1018.45	33	74	*Bacillus anthracis*	
			QIAINAGLEGSVVVER	1654.79	91		*Bacillus cereus*	
			AQLEETTSEFDREK	1682.66	45		*Bacillus thuringiensis*	
			GFTTELDVVEGMQFDR	1843.71	93			
			VASIVAEGDEATGINIVLR	1926.90	74			
			TNDVAGDGTTTATVLAQAMIR	2105.88	99			
			AMLEDIAILTGGEVITEELGR	2229.99	103			
			SSIAQVAAISAADEEVGQLIAEAMER	2659.08	175			
**1-pyrroline-5- carboxylate**		244	VNPGQIWLKR	1210.62	41	71	*Bacillus anthracis*	
**dehydrogenase**			AMQVADETFQTWR	1582.61	65		*Bacillus cereus*	
			AVIHEDVYDHVLNR	1679.74	72		*Bacillus thuringiensis*	
			ILAGGEGDDSKGWFIQPTIVADVAEDAR	2930.20	69			

**Alanine dehydrogenase**	8	416	SLPLLAPMSEVAGR	1440.68	69	77	*Bacillus anthracis*	
			IGIPTEIKNNENR	1497.68	68		*Bacillus thuringiensis*	
			VKEPVASEYGYFR	1544.65	57		*Bacillus cereus*	
			VVSIAYETVQLDNR	1606.71	86			
			HGVVHYAVANMPGAVPR	1774.77	58			
**Alcohol dehydrogenase**	13	272	LVLDGIEVVGSLVGTR	1626.84	59	60	*Bacillus anthracis*	
			LVLDGIEVVGSLVGTRK	1754.93	48		*Bacillus thuringiensis*	
			VVAVGLPVETMDLNIPR	1822.90	81		*Bacillus cereus*	
			VVPVVQTCSLDKVQNVFEEMEQGR	2791.19	85			
**50S ribosomal protein L3**	7	366	RPGSMGPVAPNR	1238.58	61	78	*Bacillus cereus*	
			LTNKPEQGHTAK	1323.63	41		*Bacillus anthracis*	
			TTETDGYNAIQLGFEDKR	2057.87	93		*Bacillus weihenstephanensis*	
			MGGDQVTIQNLEIVQVDTER	2245.00	172			
**Superoxide dismutase [Mn]**	10	324	FGSFDAFKEEFAK	1522.63	80	59	*Bacillus cereus*	
			SVEELVANLNEVPEAIR	1881.87	116		*Bacillus anthracis*	
			RPDYIGAFWNVVDWNAAEK	2250.95	40		*Bacillus thuringiensis*	
			TPVIGLDVWEHAYYLNYQNR	2451.04	88			
**Alkyl Hydroperoxide reductase**	3	209	TITTNFNVLMEEEGLAAR	2008.91	120	46	*Bacillus cereus*	
			GTFIIDPDGVIQSMEINADGIGR	2418.07	89		*Bacillus anthracis*	
							Bacillus thuringiensis	
**B (Soil)**								
**Formate acetyltransferase**	17	123	-	-		-	*Bacillus thuringiensis*	*B. anthracis*
**S-layer protein EA1**	13	313	AEAAQFIALTDKK	1405.71	62	43	*Bacillus cereus*	
			ATVEIVQETIAIK	1414.76	58		*Bacillus anthracis*	
			SVNFKPVQTENFVEK	1765.79	46		*Bacillus thuringiensis*	
			LSGKDFALNSQNLVVGEK	1918.95	57			
			NVVFALDADNDGVVNYGSK	1996.89	94			
**Formate acetyltransferase**	15	125	TGEPFAPGANPMHGR	1538.65	53	42	*Bacillus cereus*	
							Bacillus anthracis	
							Bacillus thuringiensis	

**Chaperonin GroEL**	12	521	ALEEPVR	813.42	36	13	*Bacillus anthracis*	
			QIAINAGLEGSVVVER	1654.88	54		*Bacillus cereus*	
			AQLEETTSEFDREK	1682.75	65		*Bacillus thuringiensis*	
			VASIVAEGDEATGINIVLR	1927.02	71			
**Phosphopyruvate hydratase**	-	210	STIIDVYAR	1037.54	36	10	*Bacillus anthracis*	
			AIVPSGASTGEHEAVELR	1822.91	40		*Bacillus cereus*	
			GNPTVEVEVYTESGAFGR	1911.89	84		*Bacillus thuringiensis*	
			GLNTAVGDEGGFAPNLGSNR	1945.92	50			
**Dihydro-lipoamide dehydrogenase**	11	223	-	-		-	-	
**Alanine dehydrogenase**	9	539	MSAQIGAQFLEK	1322.57	65	83	*Bacillus anthracis*	
			SLPLLAPMSEVAGR	1440.66	77			
			IGIPTEIKNNENR	1497.68	57			
			VVSIAYETVQLDNR	1606.70	62			
			HGVVHYAVANMPGAVPR	1774.76	73			
			IAVGLGADVTIIDLSAER	1812.84	96			
			GAGLGSGFTDEEYVQAGAK	1856.70	88			
**TPP-dependent acetoin**	-	214	LIVIDEANPR	1139.63	58	6	*Bacillus anthracis*	
**dehydrogenase E1 beta-**			CSIATDIAAIVADR	1475.74	42		*Bacillus cereus*	
**subunit**			ITAPHTPVPFSPPLEK	1730.93	72		*Bacillus thuringiensis*	
			LVLDGIEVVGSLVGTR	1626.92	58			
			VVAVGLPVETMDLNIPR	1822.99	86			
**Alcohol dehydrogenase**	9	-	-	-		-	*Bacillus cereus*	
**Pyruvate dehydrogenase complex E1**	11	417	ATEGLQAEFGEDR	1422.55	39	72	*Bacillus anthracis*	
**Component subunit beta**			QAGIAANVVAEINDR	1540.70	59		*Bacillus cereus*	
			EGTDVSVIAYGAMVHAALK	1931.82	42			
			SFRQDVPEGEYTIDLGK	1953.81	42			
			NDPNVLVFGEDVGVNGGVFR	2103.87	111			
**Superoxide dismutase, Mn**	6	309	FGSFDAFKEEFAK	1522.60	82	80	*Bacillus anthracis*	
			SVEELVANLNEVPEAIR	1881.84	103		*Bacillus cereus*	
			RPDYIGAFWNVVDWNAAEKR	2406.98	49		*Bacillus thuringiensis*	
			TPVIGLDVWEHAYYLNYQNR	2451.00	75			
**Alkyl hydroperoxide reductase**	6	402	TITTNFNVLMEEEGLAAR	2008.83	120	78	*Bacillus cereus*	
			ANAYHNGEFIQVTDESLK	2035.82	75		*Bacillus anthracis*	

			GTFIIDPDGVIQSMEINADGIGR	2417.99	122		*Bacillus thuringiensis*	
			AWHDSSETIGKIEYIMIGDPTR	2519.01	66			
**C (Soil)**								
**No protein identified**	-	-	-	-		-	-	Blank
**D (Soil)**								
**S-layer protein**	-	142	NGDAVFNAGDVK	1206.49	61	72	*Bacillus anthracis*	*C. perfringens*
**EA1**			SVNFKPVQTENFVEK	1765.77	41		*Bacillus thuringiensis*	*B. anthracis*
			GAITGKPDGTYGPTESIDR	1934.80	41			
**Bifunctional acetaldehyde-**	15	210	-	-		-	*Clostridium perfringens*	
**CoA/alcohol dehydrogenase**								
**Formate acetyltransferase**	9	94	QMQFFGAR	984.41	37	61	*Bacillus cereus*	
			TGEPFAPGANPMHG	1538.62	49		*Bacillus anthracis*	
							Bacillus thuringiensis	
**Dihydrolipoyl dehydrogenase**	8	152	GIIEIDEQCR	1232.53	39	58	*Bacillus cereus*	
			GEIQTVEADYVLVTVGR	1848.85	50		*Bacillus anthracis*	
			VVGDFPIELDTVVVGAGPGGYVAAIR	2571.23	42		*Bacillus thuringiensis*	
**Alanine dehydrogenase**	10	389	MSAQIGAQFLEK	1322.62	44	39	*Bacillus anthracis*	
			SLPLLAPMSEVAGR	1440.72	68			
			IGIPTEIKNNENR	1497.74	71			
			VKEPVASEYGYFR	1544.72	24			
			VVSIAYETVQLDNR	1606.78	47			
			HGVVHYAVANMPGAVPR	1774.84	42			
			GAGLGSGFTDEEYVQAGAK	1856.80	65			
**Acetoin dehydrogenase**	8	111	LIVIDEANPR	1139.57	52	58	*Bacillus cereus*	
**E1 component beta-subunit**			ITAPHTPVPFSPPLEK	1730.84	59		*Bacillus anthracis*	
							Bacillus thuringiensis	
**NADH dehydrogenase subunit 2**	6	60	-	7639		-	*Bacillus cereus*	
**Superoxide dismutase [Mn]**	4	190	FGSFDAFKEEFAK	1522.65	63	49	*Bacillus cereus*	
			SVEELVANLNEVPEAIR	1881.89	77		*Bacillus anthracis*	
			TPVIGLDVWEHAYYLNYQNR	2451.07	49		*Bacillus thuringiensis*	
**E (Sand)**								

**No protein identified**	-	-	-	-		-	-	Blank
**F (Soil)**								
**Bifunctional acetaldehyde-**	12	172	-	94580		-	*Clostridium perfringens*	*C. perfringens*
**CoA/alcohol dehydrogenase**								
**Glyceraldehyde-3-phosphate**	4	96	-	35340		-	*Clostridium perfringens*	
**dehydrogenase, type I**								
**Hypothetical protein CPE1233**	4	85	VALDADDQIIGYR	1448.64	57	75	*Clostridium perfringens*	
**G (Sand)**								
**Alanine dehydrogenase**	10	498	MSAQIGAQFLEK	1322.59	77	67	*Bacillus anthracis*	*B. anthracis*
			SLPLLAPMSEVAGR	1440.69	81		*Bacillus pseudomycoides*	
			IGIPTEIKNNENR	1497.70	53		*Bacillus mycoides*	
			HGVVHYAVANMPGAVPR	1774.80	51		*Bacillus cereus*	
			GAGLGSGFTDEEYVQAGAK	1856.73	102			
			ALIDNKVVSIAYETVQLDNR	2261.04	99			
**TPP-dependent acetoin**	9	227	LIVIDEANPR	1139.57	45	59	*Bacillus anthracis*	
**dehydrogenase E1**			GFDLLDAPIKR	1244.62	30		*Bacillus cereus*	
			ITAPHTPVPFSPPLEK	1730.83	78		*Bacillus thuringiensis*	
			SLSPLDEDTILSSVEKTNR	2103.96	34			
**30S ribosomal protein S4**	12	402	VDIPSYR	849.39	31	75	*Bacillus anthracis*	
			HGENFMILLEAR	1429.62	51		*Bacillus cereus*	
			RPYAPGPHGPNQR	1446.63	59		*Bacillus thuringiensis*	
			QLVNHGHIMVDGAR	1546.67	37			
			AELPAEINEALIVEFYSR	2063.90	120			
			HAERAELPAEINEALIVEFYSR	2557.08	39			
			EAIEVNNFVPEYLTFDADKLEATYTR	3048.22	68			
**50S ribosomal protein L6**	6	116	-	19504		-	*Bacillus cereus*	
**Ribosomal protein S7p/S5e**	8	64	-	8821		74	*Bacillus anthracis*	
**H (Sand)**								
**Pyruvate- flavodoxin**	14	103	IPFVNFFDGFR	1358.63	38	45	*Clostridium perfringens*	*C. perfringens*
**oxidoreductase**			GTAQNPDIYFQER	1538.65	44			
**Bifunctional acetaldehyde**	17	389	YGALGVALR	919.47	36	65	*Clostridium perfringens*	
**CoA/alcohol dehydrogenase**			FNAVDNPR	932.39	43			

			YPLISEIK	962.49	58			
			NAVFFSPHPR	1171.52	38			
			FSQEQVDEIFR	1397.58	83			
			VANFEEALVKAER	1475.68	34			
			VLIGEVESVELEEPFSHEK	2169.95	119			
**70kDa heat shock protein**	9	153	AKFNELTHDLVER	1571.69	65	70	*Clostridium perfringens*	
**Elongation factor Tu**	7	151	LLDEAQAGDNIGALLR	1668.76	56	72	*Clostridium perfringens*	
			GVLHVGDEVEVIGLTEER	1950.87	48			
**Elongation factor Tu**	14	633	TVVTGIEMFR	1152.53	37	68	*Clostridium perfringens*	
			LLDEAQAGDNIGALLR	1668.77	133			
			ELMDAVDSYIPTPER	1735.70	84			
			GITINTAHVEYETANR	1788.76	67			
			GVLHVGDEVEVIGLTEER	1950.87	50			
			HTPFFDGYRPQFYFR	1977.80	30			
			GSALVALENPTDEAATACIR	2058.87	72			
			GVLHVGDEVEVIGLTEERR	2106.96	46			
			ATDKPFLMPVEDVFTITGR	2136.94	81			
			ATDKPFLMPVEDVFTITGR	2152.94	46			
**TARGETED SEARCH**								
**A (Sand)**								
**Formate acetyltransferase**	16	147	-	-		-	*Bacillus anthracis*	*C. perfringens*
							Bacillus cereus E33L	*B. anthracis*
**S-layer protein EA1**	27	433	ADLYDTLTTK	1140.51	40	53	*Bacillus anthracis*	
			GAITGKPDGTYGPTESIDR	1934.84	57		*Bacillus thuringiensis*	
			SFPDVPAGHWAEGSINYLVDK	2302.00	125			
			TLPVTFVTTDQYGDPFGANTAAIK	2527.13	124			
**Formate acetyltransferase**	32	288	ADLNLTGGVMSEDTMR	1709.69	40	51	*Bacillus anthracis*	
			THNQGVFDAYTPEMR	1765.70	37		*Bacillus thuringiensis*	
			ADYGDDYGIACCVSAMR	1923.66	32			
			ETLMDAMEHPEKYPQLTIR	2301.99	48			
**Chaperonin GroEL**	19	123	-	-		-	*Bacillus anthracis*	
							Bacillus thuringiensis	
**Elongation factor Tu**	6	179	GYDQIDAAPEER	1363.53	40	58	*Bacillus cereus*	
			HYAHVDCPGHADYVK	1768.68	67		*Bacillus pseudomycoides*	

			IIELMAEVDAYIPTPER	1959.89	57		*Bacillus anthracis*	
**Alanine dehydrogenase**	16	154	IGIPTEIKNNENR	1497.70	47		*Bacillus mycoides*	
			VKEPVASEYGYFR	1544.68	33		*Bacillus cereus*	
			GAGIGSGFTDEEYVQAGAK	1856.76	51		*Bacillus anthracis*	
							Bacillus thuringiensis	
**Acetoin:2,6- dichlorophenolind**	9	173	LIVIDEANPR	1139.59	45	46	*Bacillus cereus*	
**ophenol oxidoreductase subunit beta**			GEVPEGYYTIPLGK	1522.69	43		*Bacillus anthracis*	
			ITAPHTPVPFSPPLEK	1730.86	63		*Bacillus thuringiensis*	
**50S ribosomal protein L3**	12	79	-	-		-	*Bacillus thuringiensis*	
**Superoxide dismutase [Mn]**	3	119	FGSFDAFKEEFAK	1522.64	86	52	*Bacillus cereus*	
			RPDYIGAFWNVVDWNAAEK	2250.96	33		*Bacillus anthracis*	
**B (Soil)**								
**S-layer protein EA1**	16	245	YTDKGTLVFK	1171.61	39	12	*Bacillus anthracis*	*B. anthracis*
			ATVEIVQETIAIK	1414.81	47		*Bacillus thuringiensis*	
			SFPDVPAGHWAEGSINYLVDK	2302.13	57			
			FVANNLDGSPANIFEGGEATSTTGK	2497.23	33			
			TLPVTFVTTDQYGDPFGANTAAIK	2527.29	42			
**Formate acetyltransferase**	21	163	-	-			*Bacillus anthracis*	
							Bacillus thuringiensis	
							Bacillus cereus	
**Dihydrolipoyllysine-residue**	12	68	-	-		-	*Bacillus cereus*	
**acetyltransferase component of**								
**pyruvate dehydrogenase complex**								
**Alanine dehydrogenase**	7	295	ITTHDNPTYEK	1318.53	32		*Bacillus anthracis*	
			MSAQIGAQFLEK	1322.59	40		*Bacillus thuringiensis*	
			IGIPTEIKNNENR	1497.70	64		*Bacillus cereus*	
			HGVVHYAVANMPGAVPR	1774.80	58			
			GAGLGSGFTDEEYVQAGAK	1856.76	63			
			ALIDNKVVSIAYETVQLDNR	2261.06	40			
**Acetoin dehydrogenase E1**	5	169	LIVIDEANPR	1139.57	57	63	*Bacillus cereus*	

			ITAPHTPVPFSPPLEK	1730.83	112		*Bacillus anthracis*	
							Bacillus thuringiensis	
**Pyruvate dehydrogenase E1**	13	234	VVIPSTPYDAK	1189.57	34	60	*Bacillus cereus*	
			QAGIAANVVAEINDR	1540.71	44		*Bacillus anthracis*	
			EGTDVSVIAYGAMVHAALK	1931.84	34		*Bacillus weihenstephanensis*	
			AAEELEKEGISLEVVDLR	1999.92	34			
			NDPNVLVFGEDVGVNGGVFR	2103.91	35			
			VAAADTVFPFSQAESVWLPNHK	2414.07	30			
**C (Soil)**								
**No protein identified**	-	-	-	-		-	-	Blank
**D (Soil)**								
**S-layer protein EA1**	8	153	ADLYDTLTTK	1140.52	39	56	*Bacillus anthracis*	*C. perfringens*
			DNAQAYVTDVK	1223.52	31		*Bacillus thuringiensis*	*B. anthracis*
**Alanine dehydrogenase**	14	141	MSAQIGAQFLEK	1322.61	40	43	*Bacillus anthracis*	
			IGIPTEIKNNENR	1497.73	37		*Bacillus thuringiensis*	
			HGVVHYAVANMPGAVPR	1774.84	36		*Bacillus mycoides*	
			GAGLGSGFTDEEYVQAGAK	1856.78	48			
**Acetoin:2,6-Dichlorophenolindo**	8	70	ITAPHTPVPFSPPLEK	1730.87	47	35	*Bacillus cereus*	
**phenol oxidoreductase**							*Bacillus thuringiensis*	
							Bacillus anthracis	
**Superoxide dismutase**	6	64	FGSFDAFKEEFAK	1521.66	64	29	*Bacillus cereus*	
							Bacillus anthracis	
							Bacillus thuringiensis	
**E (Sand)**								
**No protein identified**	-	-	-	-		-	-	Blank
**F (Soil)**								
**Hypothetical protein**	6	69	-	-		-	*Clostridium sp.*	*C. perfringens*
**HMPREF1020_023 52**								
**G (Sand)**								

**Alanine dehydrogenase**	7	151	MSAQIGAQFLEK	1322.62	53	34	*Bacillus cereus*	*B. anthracis*
			HGVVHYAVANMPGAVPR	1774.85	31		*Bacillus anthracis*	
			GAGLGSGFTDEEYVQAGAK	1856.80	67		*Bacillus thuringiensis*	
**Acetoin dehydrogenase E1**	11	98	ITAPHTPVPFSPPLEK	1730.91	51	22	*Bacillus cereus*	
							Bacillus anthracis	
							Bacillus thuringiensis	
**Superoxide dismutase [Mn]**	2	80	FGSFDAFKEEFAK	1522.64	80	49	*Bacillus cereus*	
							Bacillus anthracis	
							Bacillus thuringiensis	
**50S ribosomal protein L6**	6	72	-	-		-	*Bacillus cereus*	
**H (Sand)**								
**Putative replication protein**	5	59	-	-		-	*Clostridium tetani*	*C. perfringens*
**Bifunctional acetaldehyde-**	16	121	QMYINAFEGKK	1329.60	33	44	*Clostridium perfringens*	
**CoA/alcohol dehydrogenase**			FSQEQVDEIFR	1397.60	69			
**Elongation factor Tu**	4	72	-	-		-	*Clostridium perfringens*	
**Elongation factor Tu**	12	175	FVGQVYVLK	1052.55	36	58	*Clostridium perfringens*	
			GQVLAQVGTINPHK	1461.73	36		*Clostridium botulinum*	
			GQVLAQVGTINPHKK	1589.82	37		*Clostridium sporogenes*	
			GITINTAHVEYETANR	1788.78	36			
			ATDKPFLMPVEDVFTITGR	2136.96	38			

* MP = Matched peptides, these are the number of peptides matched to the identified protein.

Supplementary material S6: Methods for *in silico* analysis

METHODS FOR IN SILICO ANALYSIS

Selection of species specific putative marker proteins

In order to select putative marker proteins, the following criteria were envisaged: 1) the protein should have evidence of abundant expression by the microorganism; 2) it should not be closely related to its homologs in other bacterial species; and 3) should preferably be identifiable in wet lab experiments using whole cell lysate of pure culture.

The evidence for expression was sought by extensive literature mining for each pathogen (*Brucella suis*, *Brucella abortus*, *Brucella melitensis*, *Burkholderia pseudomallei*, *Burkholderia mallei*, *Clostridium perfringens*, *Clostridium botulinum*, *Clostridium tetani*, and *Bacillus anthracis*) using appropriate key words (e.g. expression, proteome, proteomics, virulence determinant, immuno protection etc.) employing PUBMED search engine at NCBI (www.ncbi.nlm.nih.gov). During the literature mining, pathogenicity related attributes (for example, immunogenicity, immuno-dominance in naturally infected clinical sera, immuno-protective nature, role in virulence, and surface localization) of each of the proteins were also noted. Predicted molecular function and cellular processes for each of the selected proteins were also listed using the ExPASy Proteomics tools (http://www.expasy.ch). Localisation of protein in the cell was also predicted using the online PSORT tool at ExPASy (http://www.expasy.ch). All these putative protein markers, previously shown to be expressed in abundance in the respective pathogenic bacterial species (9) were initially ranked in the decreasing order of the number of reports for a given protein with a cut-off of at least three reports for each. Several independent reports showing experimental evidence of expression increased the likelihood of identifying the protein using MS, from diverse matrices where the given species is present.

FASTA sequences for each of these putative marker proteins, short-listed on the basis of three or independent reports of expression, were retrieved from the reference strain of the selected bacterial species and subjected to global protein BLAST against the non-redundant protein database at NCBI and percent identity of the query sequence with its nearest homolog in another species was noted. A low percent identity of the protein with its closest homolog in any other bacterial species was given maximum weightage for an increased likely hood of getting species specific unique peptides (table 2).

Using MALDI-TOF-TOF, abundant proteins were identified in the laboratory from the cell lysates of selected bacterial species after minimal fractionation on SDS-PAGE. The peptides with significant MS/MS ion score were listed (Supplementary Table 1 A - F). The identified protein and their peptides with significant MS/MS data, indicated abundance and amenability of the peptides for the tandem MS analysis.

Prioritisation of the species specific putative marker proteins

Putative marker proteins for each of the selected species were prioritized according to an arbitrarily designed scoring scheme that was aimed at selecting proteins / peptides unique to the species and expressed by the microorganism in abundance, in turn increasing the probability of getting better signal on MS analysis. The putative marker proteins were judiciously selected to impart selectivity by employing a ranking scheme to prioritize candidate protein markers for each of the selected species by taking into consideration the following three parameters:

1. Extensive literature mining provided strong evidence of expression indicating abundance and role in virulence at the experimental level as indicated by the number of scientific reports (supplementary table 2 A-F). The number of reports for the expression of a

given protein was assigned a maximum of 25 points. Number of independent reports for the expression of a given protein, was assigned a maximum of 25 points with 5 points for 3 reports and 5 additional points for every additional report up to 7 (≥7 reports for the given protein assigned complete 25 points).

2. MS/MS based experimental identification of a given protein in our laboratory for a particular species was assigned a maximum of 25 points; significant MS/MS ion score for 1 peptide given 5 points and 5 additional points for every additional peptide up to 5 (≥5 peptides given complete 25 points). This parameter further augments the chances of scoring the protein / peptides during the envisaged verification of biological agents.
3. Percent identity with the nearest homolog was assigned a maximum of 50 points. Low percent identity with the nearest homolog in any other species was given maximum weightage for a better probability of getting unique marker peptides for directed MS based assay development. For example, 100% sequence identity fetched 0 point (100 – 100), while for a protein showing ≤50% sequence identity with its nearest homolog in any other species was given the complete 50 points on this identity parameter. Between these two extremes, a protein showing X% identity with its nearest homolog was assigned 100 - X points. This way, 15 – 27 proteins were short-listed as putative marker proteins for each of the selected species.

After adding all the points for each protein in a given species, 15 – 27 proteins were short-listed as marker proteins for each of the selected species (Table 1).

In silico digestion of species specific marker proteins and unique peptide selection

The FASTA sequences of the selected putative marker proteins were subjected to *in silico* tryptic digestion using the Peptide Mass algorithm at ExPASy Proteomics tools (http://www.expasy.org). after in silico digestion, peptides were selected in the mass range of 1000 – 3000 Da from each selected protein from the selected bacterial species. These peptides were subjected to global protein BLAST against the GeneBank non-redundant protein database (http://wwww.ncbi.nlm.nih.gov) with search parameters adjusted for short input sequence. Peptides showing less than 100% sequence identity with any other bacterial species (except the query species) were selected for the inclusion list of unique peptides. This selection was based on the assumption that a difference of even one single amino acid is most likely to ensure that the selected mass will not come from any other species except the target taxon. Peptide BLAST was given a tolerance for very closely species within the *Bacillus cereus* sensu lato group, including six other species (*B. cereus*, *B. mycoides*, *B. pseudomycoides*, *B. thuringiensis*, *B. weihenstephanensis*, and *B. cytotoxicus*). The tolerance within the *B. cereus* sensu lato group meant that the peptide queried did not show 100% sequence identity with any other species but a complete match with one or more of the six species within the group was allowed. Similarly, the closely related species of *Burkholderia* (*B. mallei* and *B. pseudomallei*) and *Brucella* (*B. suis*, *B. abortus*, and *B. melitensis*) were considered together for the peptide blast search and scoring unique peptides. In order to discriminate between these closely related pathogens, the list of putative markers was further appended with peptides unique to these related species using reported literature.

The unique peptides in the inclusion list were further screened for strain coverage using all the available sequences retrieved by species specific BLAST search and counting for the presence of a given peptide from the alignment data of these sequences from

diverse strains in the genome data base. The peptides were further curated to reduce background signal by deducting isobaric peptides observed in the MS analysis of tryptic digest from environmental bacterial consortium from garden soil.

This way, an inclusion list of masses, corresponding to the peptides selected as described above, were generated for each of the nine bacterial species and a consolidated list of nine was used for the validation experiment for the targeted analysis.

Supplementary Figure S1: MS and MS/MS spectra of tryptic peptides obtained from three representative marker proteins.

MS and MS/MS spectra of tryptic peptides obtained from DnaK from *C. perfringens* 13124

DnaK: MS SPECTRUM

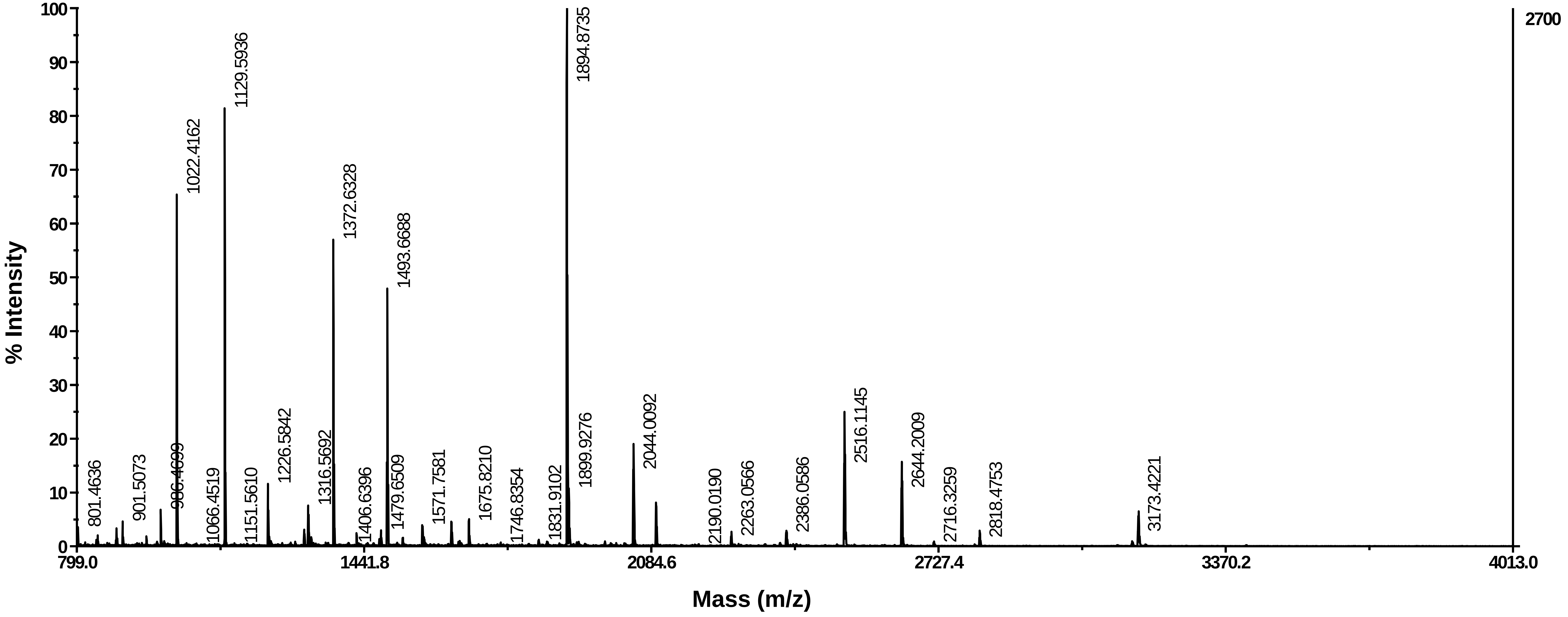

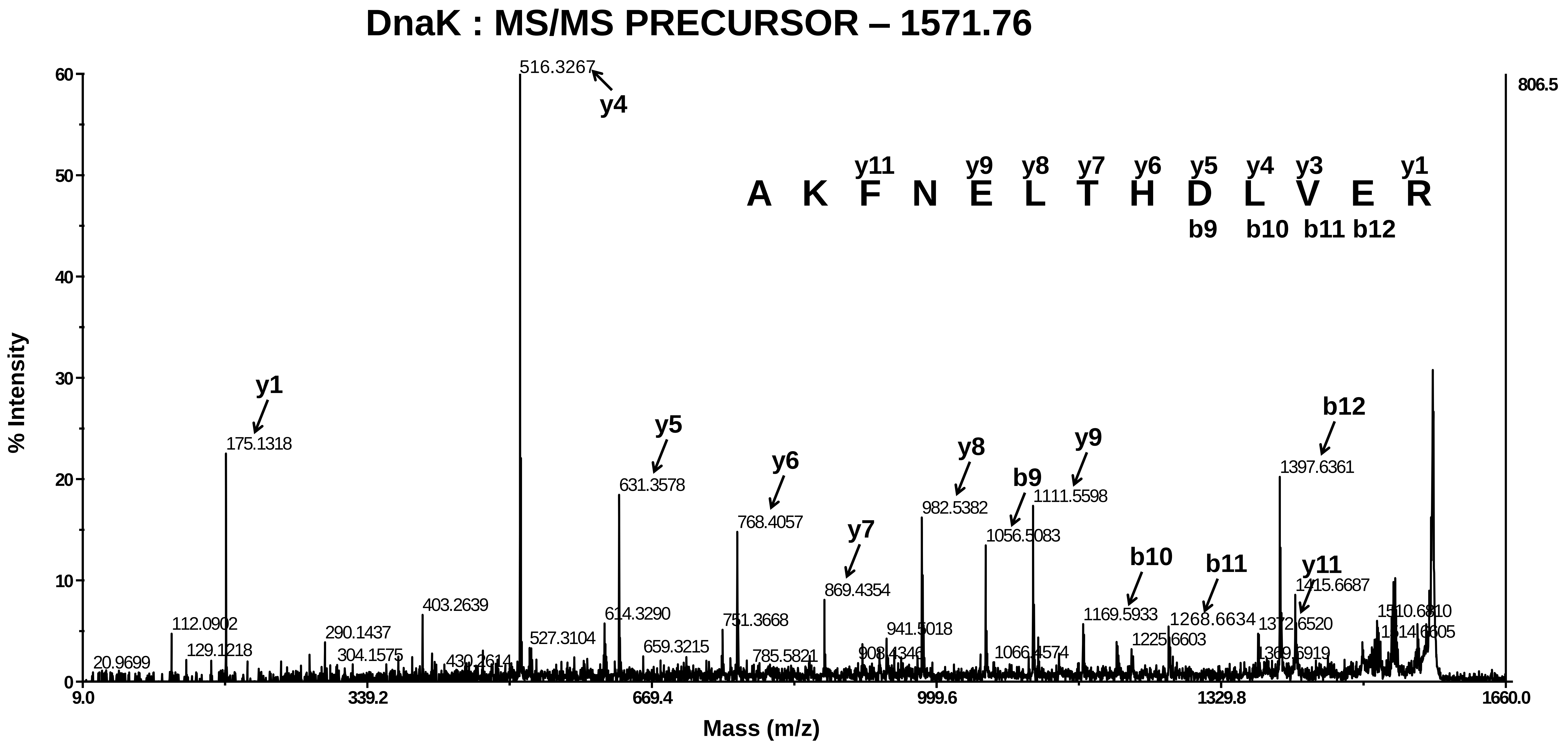
DnaK : MS/MS PRECURSOR – 1571.76
% Intensity
Mass (m/z)
806.5
A K F N E L T H D L V E R
y11 y9 y8 y7 y6 y5 y4 y3 y1
b9 b10 b11 b12
516.3267
y4
y1
175.1318
y5
631.3578
y6
768.4057
y7
869.4354
y8
982.5382
b9
1056.5083
y9
1111.5598
b10
1169.5933
b11
1268.6634
b12
1397.6361
y11
1415.6687
112.0902
129.1218
20.9699
290.1437
304.1575
403.2639
430.2614
527.3104
614.3290
659.3215
751.3668
785.5821
908.4346
941.5018
1066.4574
1225.6603
1372.6520
1369.6919
1510.6810
1514.6605
9.0
339.2
669.4
999.6
1329.8
1660.0

DnaK : MS/MS PRECURSOR – 986.47

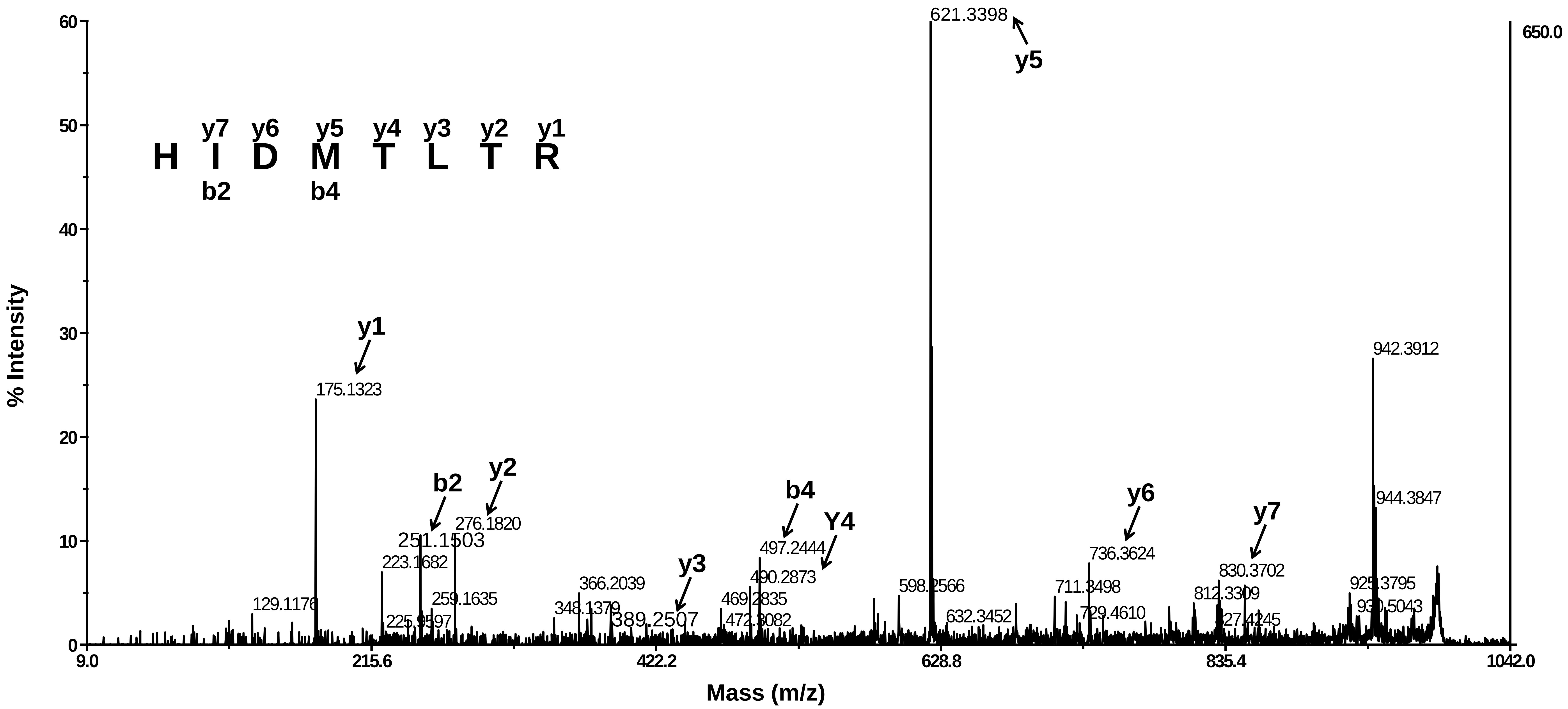

DnaK : MS/MS PRECURSOR – 2818.48

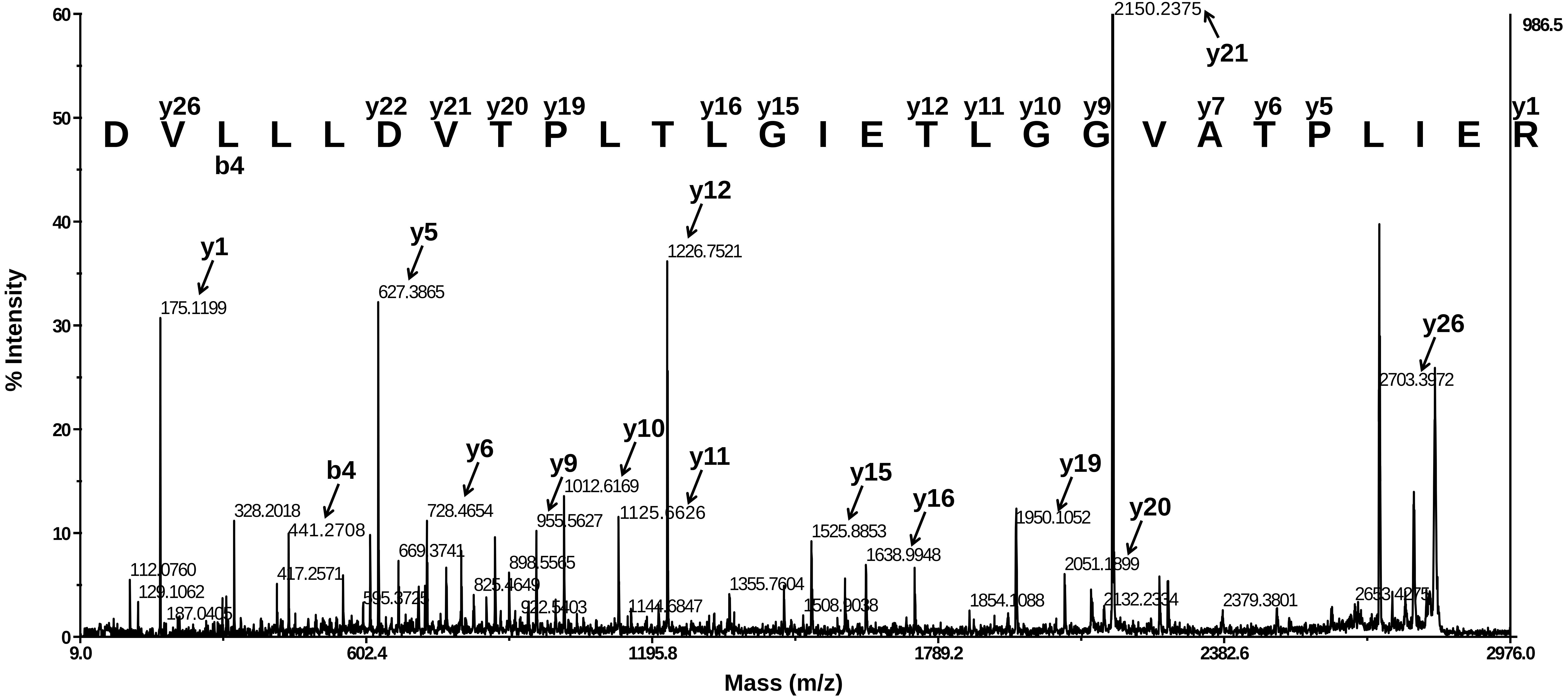

DnaK : MS/MS PRECURSOR – 1675.82

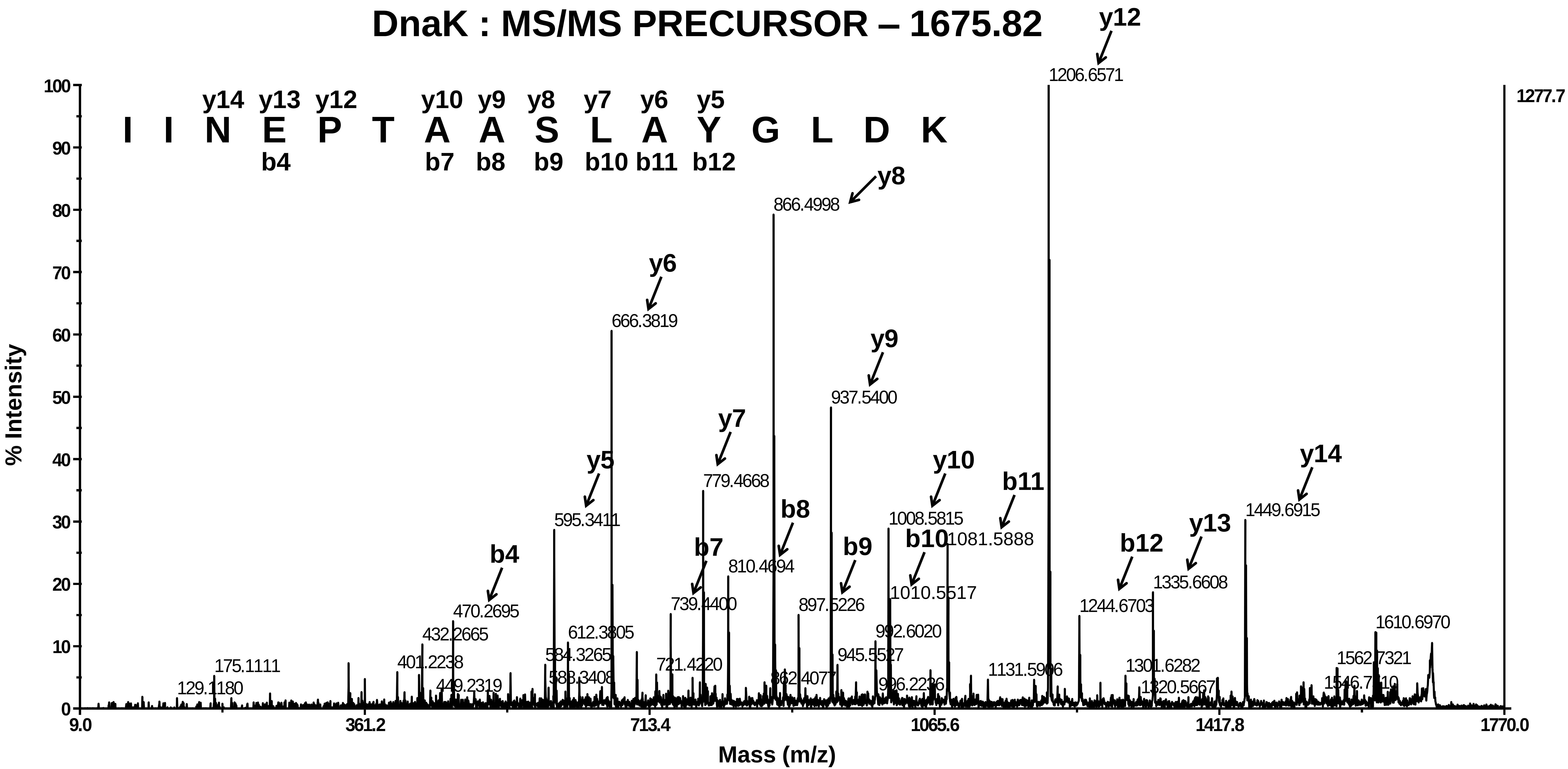

DnaK : MS/MS PRECURSOR – 1636.76

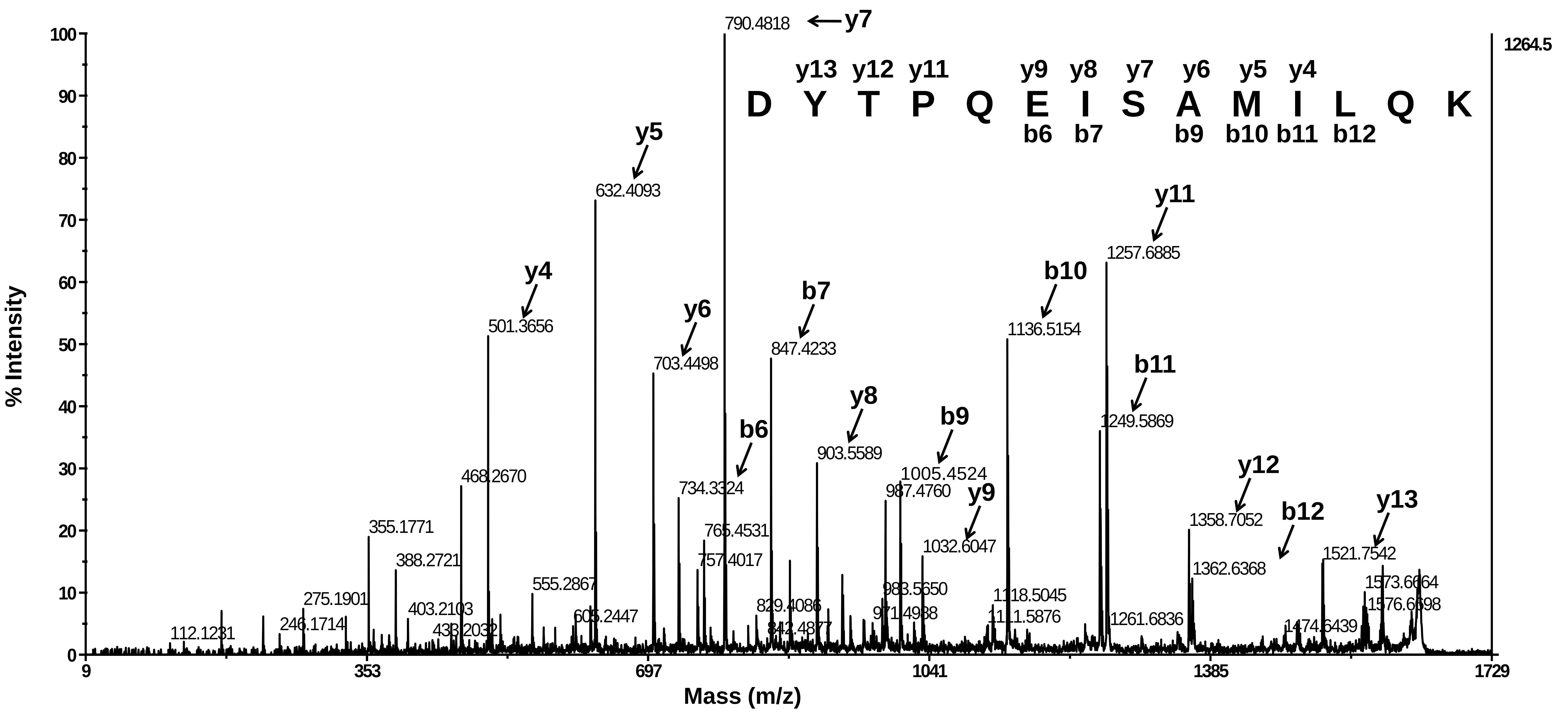

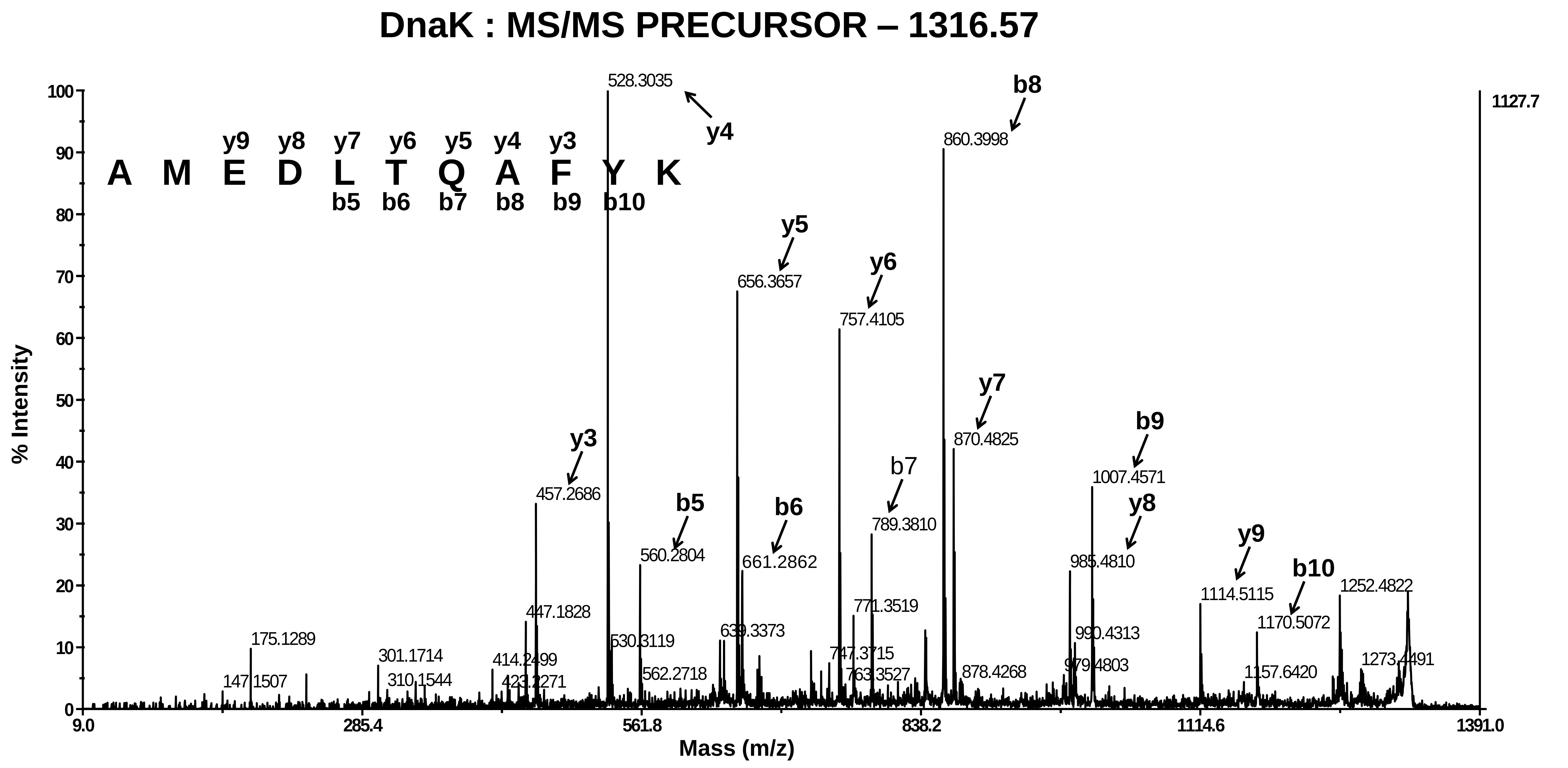

DnaK : MS/MS PRECURSOR – 1316.57
A M E D L T Q A F Y K
y9 y8 y7 y6 y5 y4 y3
b5 b6 b7 b8 b9 b10
528.3035
y4
860.3998
b8
1127.7
y5
656.3657
y6
757.4105
y7
870.4825
b9
1007.4571
y8
985.4810
b7
789.3810
y3
457.2686
b5
560.2804
b6
661.2862
y9
1114.5115
b10
1170.5072
1252.4822
771.3519
639.3373
447.1828
175.1289
530.3119
990.4313
301.1714
414.2499
747.3715
979.4803
1273.4491
147.1507
310.1544
423.2271
562.2718
763.3527
878.4268
1157.6420
% Intensity
Mass (m/z)
0
10
20
30
40
50
60
70
80
90
100
9.0
285.4
561.8
838.2
1114.6
1391.0

DnaK : MS/MS PRECURSOR – 2094.98

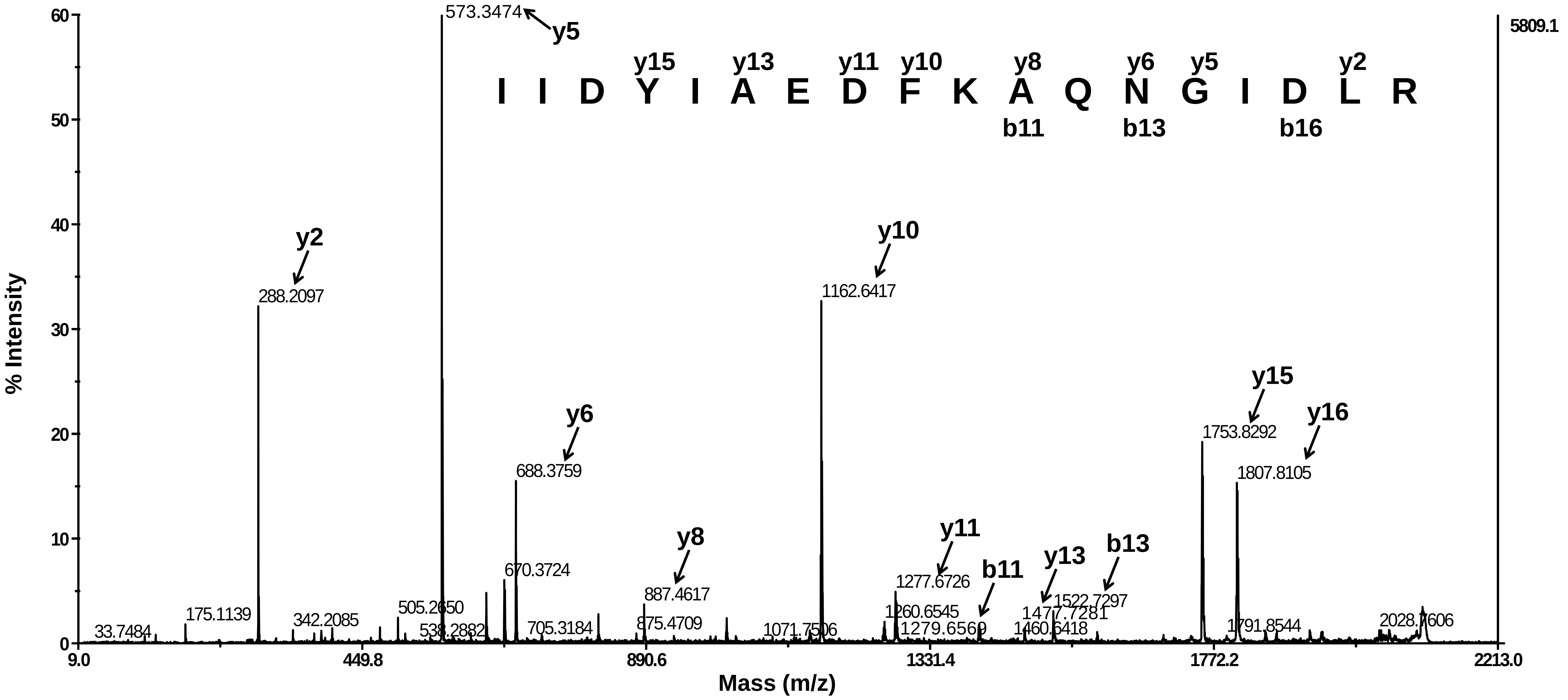

DnaK : MS/MS PRECURSOR – 1226.58

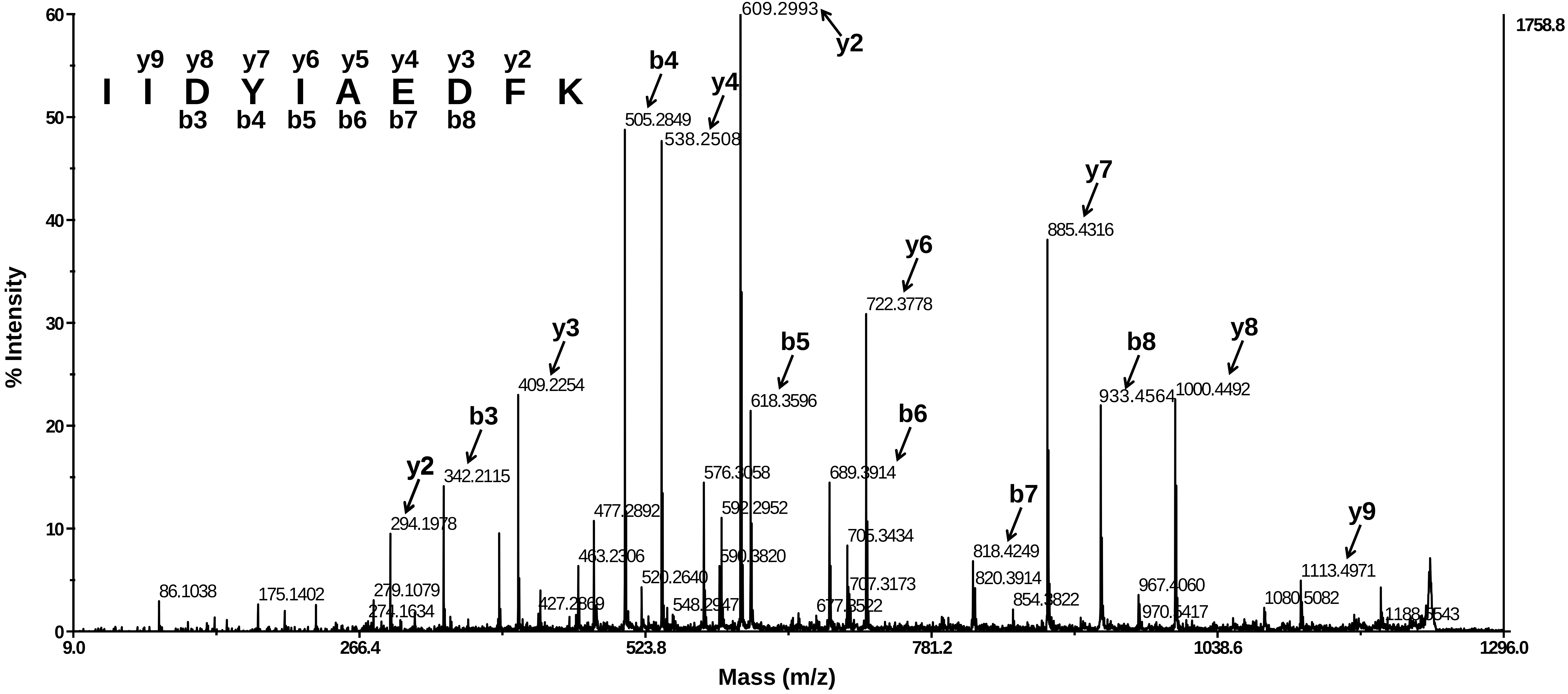

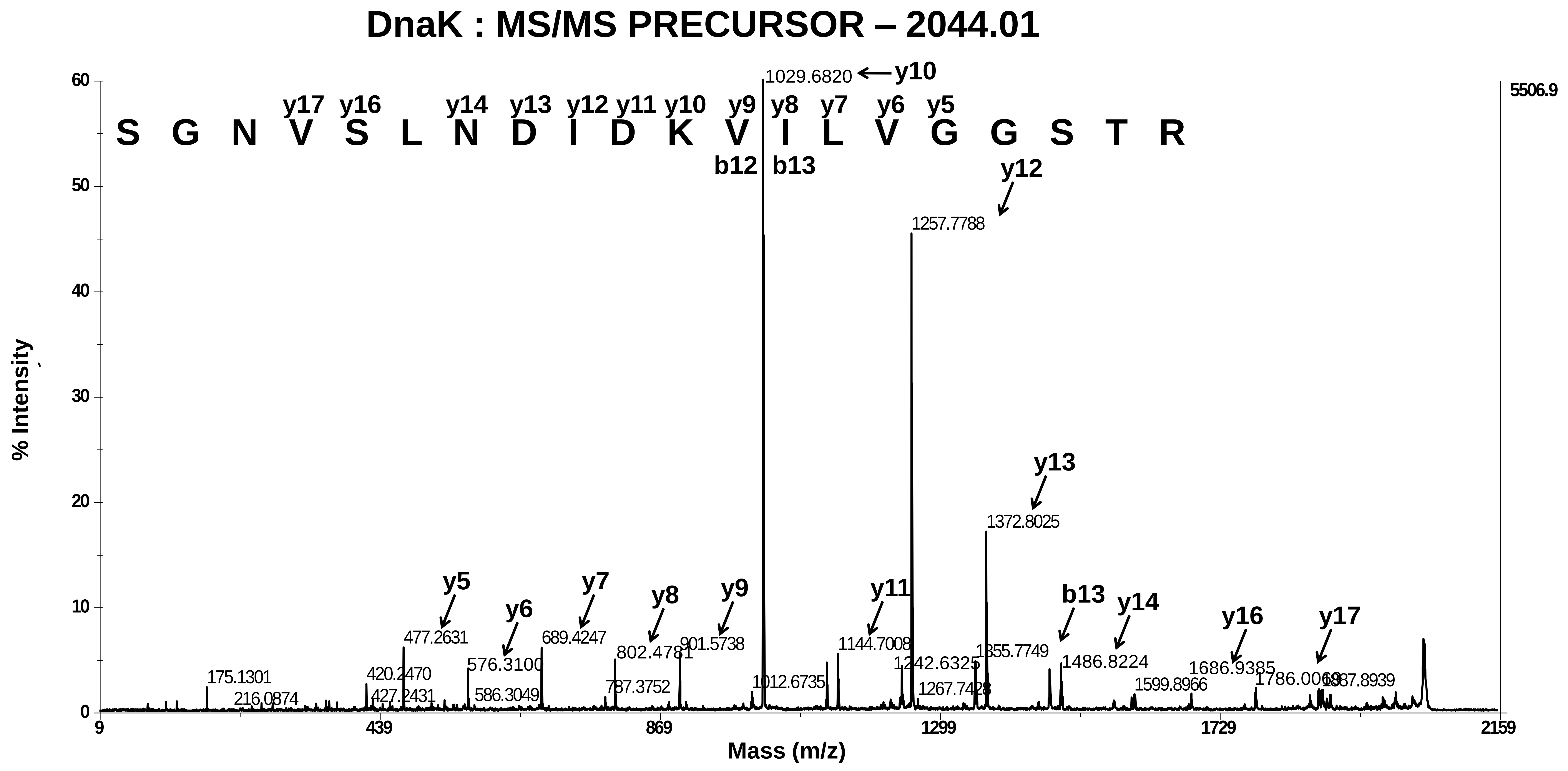

DnaK : MS/MS PRECURSOR – 2044.01
S G N V S L N D I D K V I L V G G S T R
y17 y16 y14 y13 y12 y11 y10 y9 y8 y7 y6 y5
b12 b13
1029.6820 ← y10
y12
1257.7788
y13
1372.8025
y5
477.2631
y6
576.3100
y7
689.4247
y8
802.4781
y9
901.5738
y11
1144.7008
b13
y14
y16
y17
175.1301
216.0874
420.2470
427.2431
586.3049
787.3752
1012.6735
1242.6325
1267.7428
1355.7749
1486.8224
1599.8966
1686.9385
1786.0069
1887.8939
5506.9
% Intensity
Mass (m/z)
0
10
20
30
40
50
60
9
439
869
1299
1729
2159

DnaK : MS/MS PRECURSOR – 2644.2

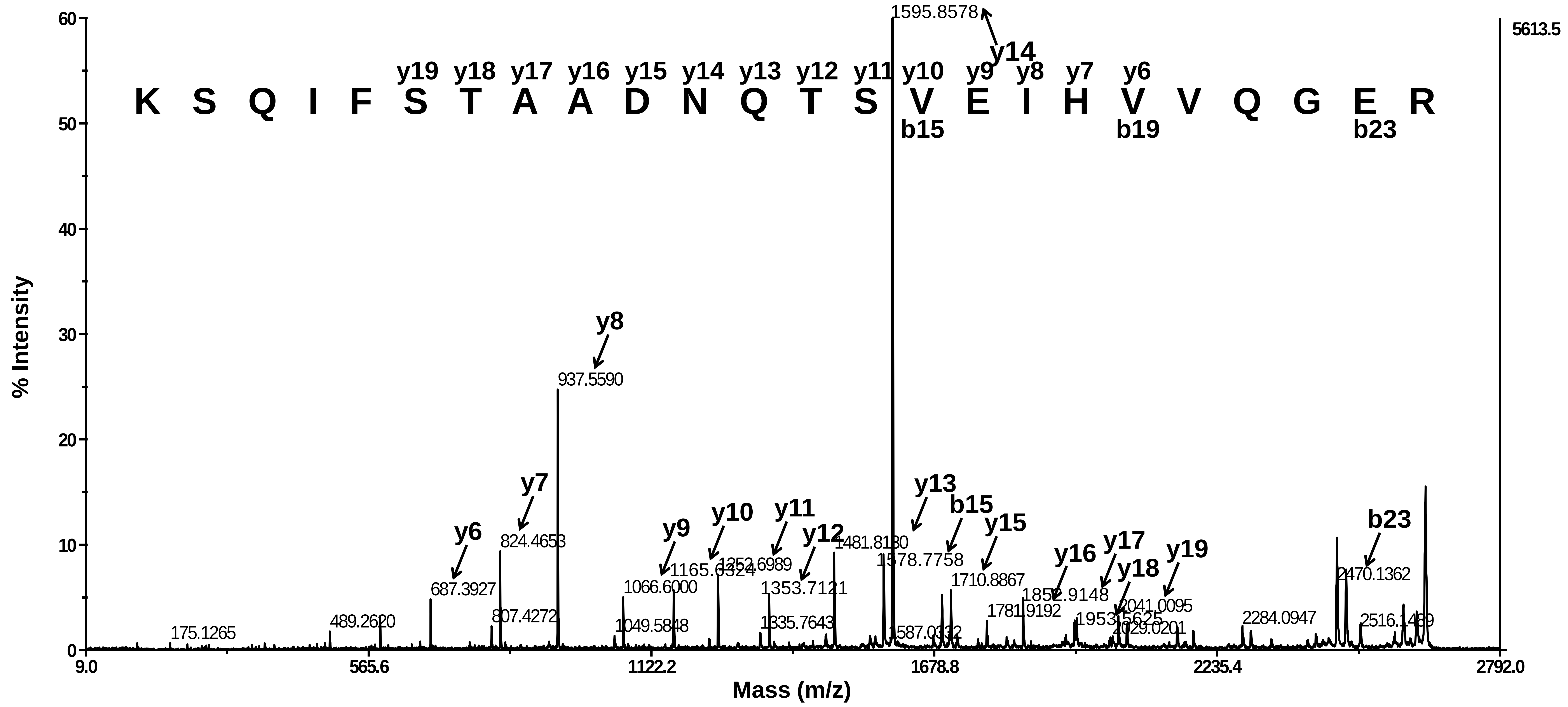

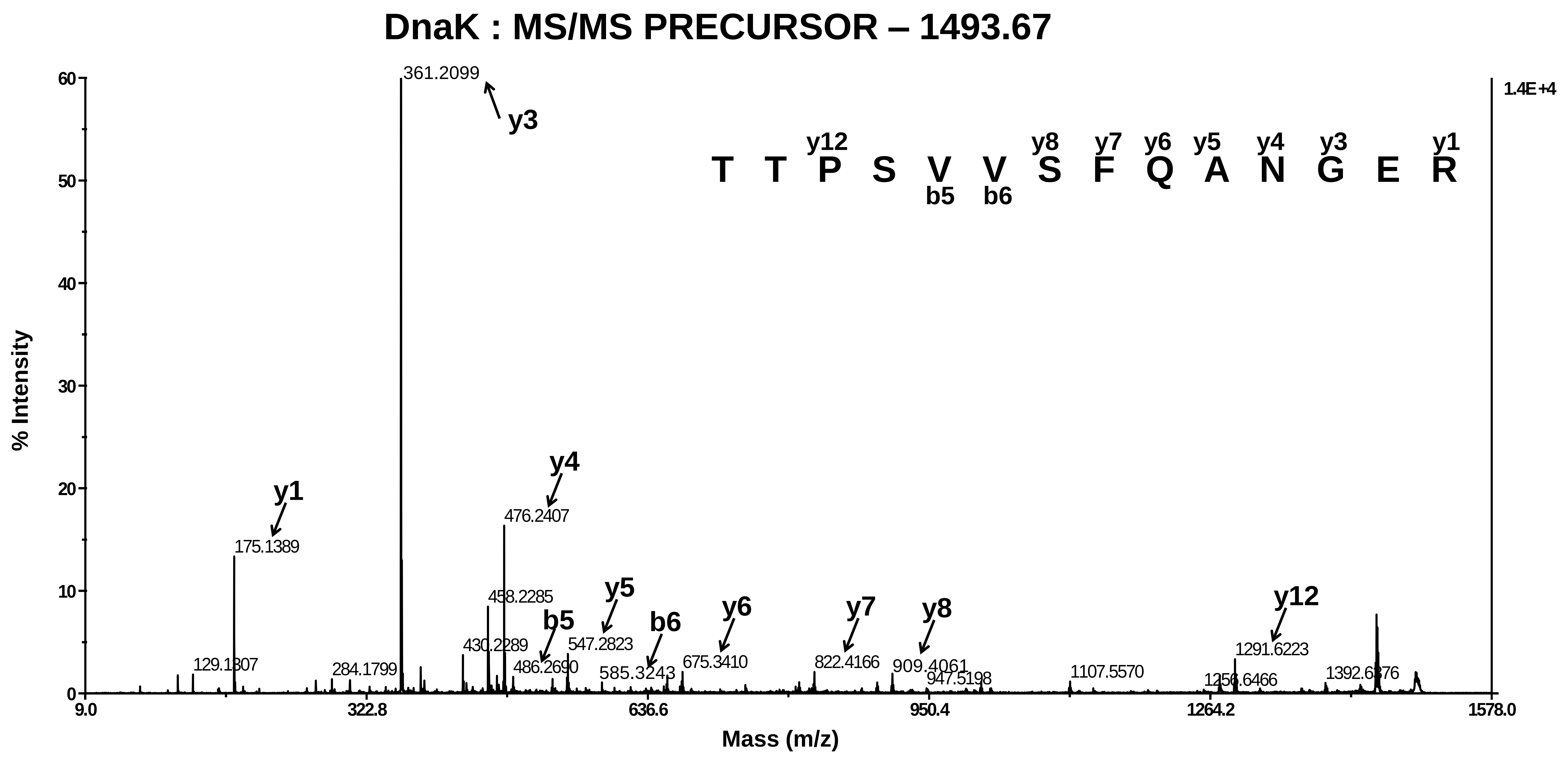

DnaK : MS/MS PRECURSOR – 1493.67
% Intensity
Mass (m/z)
1.4E+4
T T P S V V S F Q A N G E R
y12 y8 y7 y6 y5 y4 y3 y1
b5 b6
361.2099
y3
y1
175.1389
y4
476.2407
458.2285
430.2289
b5
486.2690
y5
547.2823
b6
585.3243
y6
675.3410
y7
822.4166
y8
909.4061
947.5198
1107.5570
y12
1291.6223
1256.6466
1392.6376
129.1307
284.1799

DnaK : MS/MS PRECURSOR – 2516.11

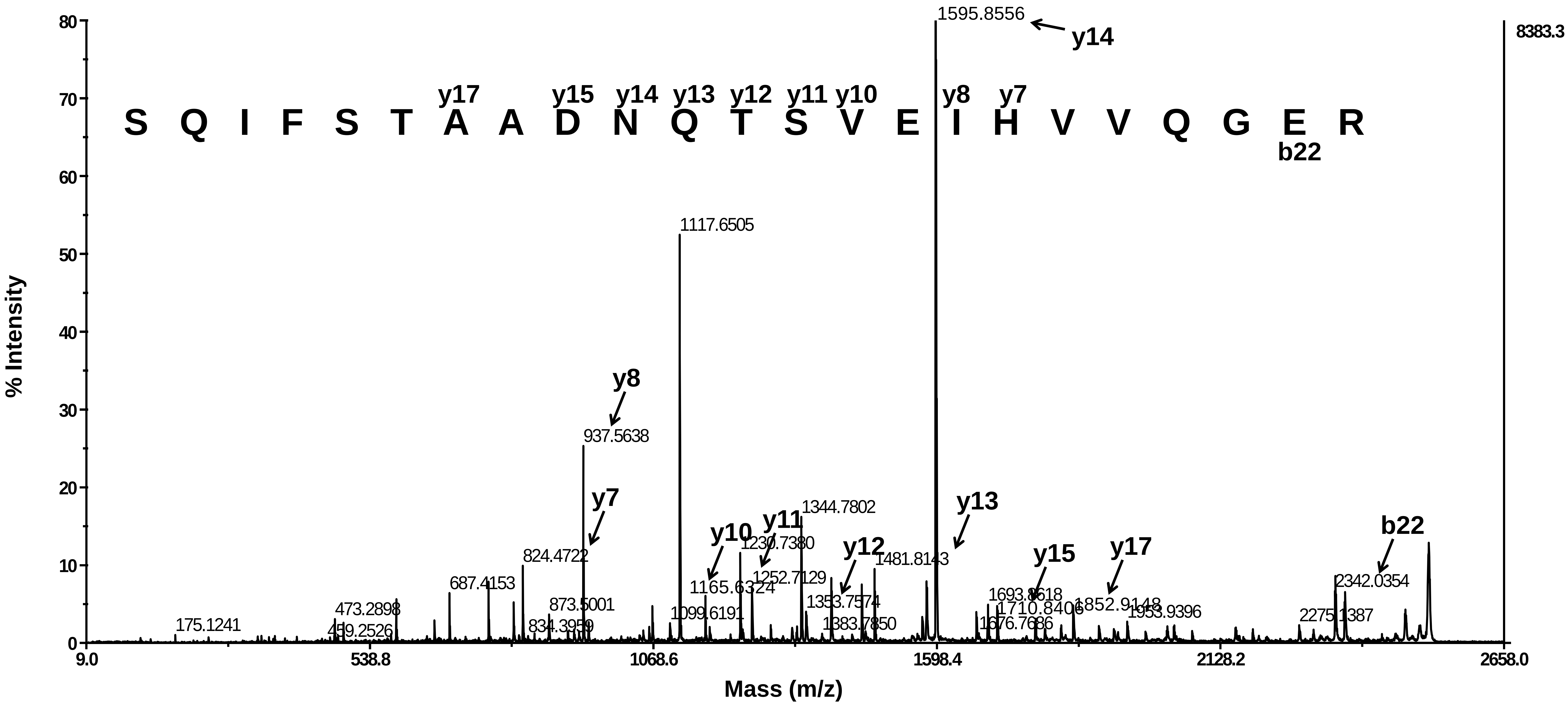

DnaK : MS/MS PRECURSOR – 1022.41

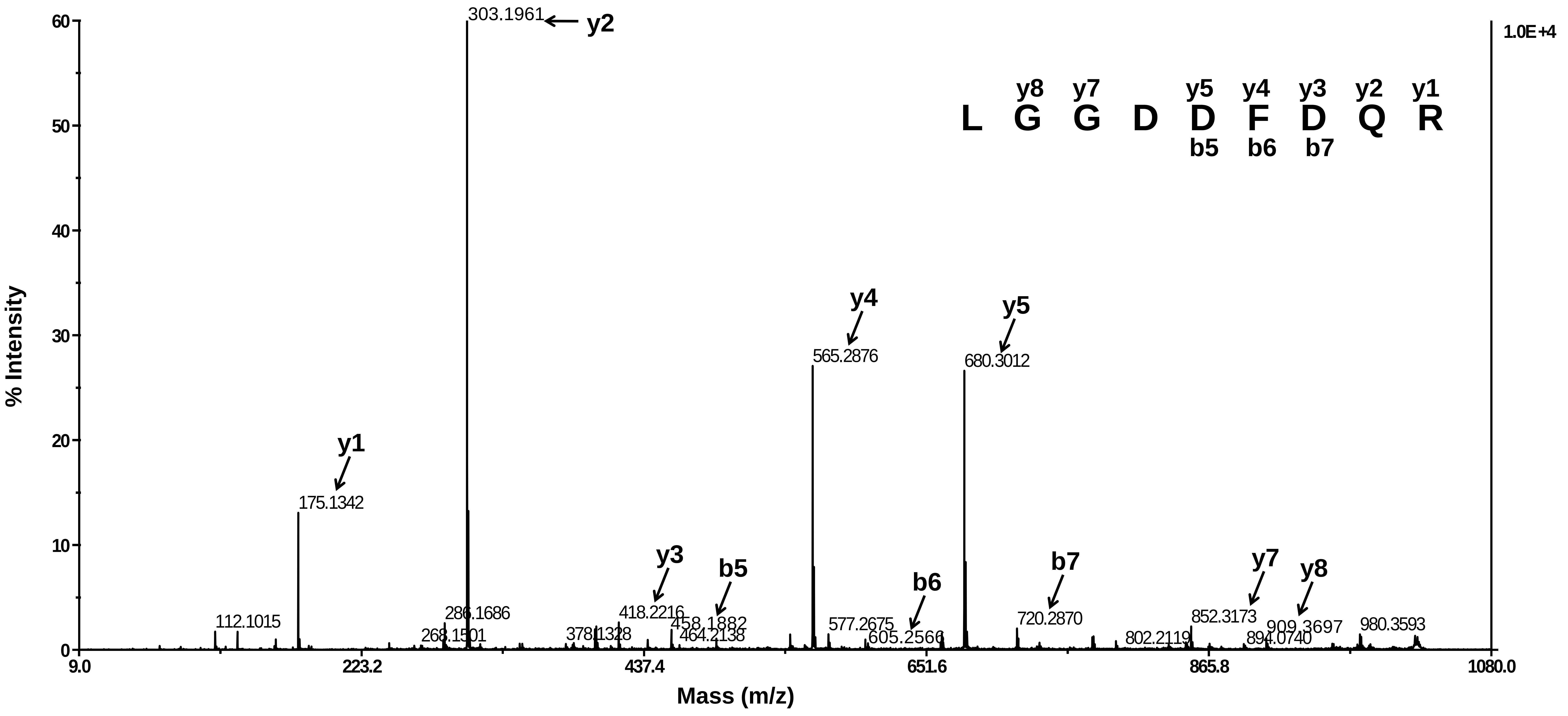

DnaK : MS/MS PRECURSOR – 1372.63

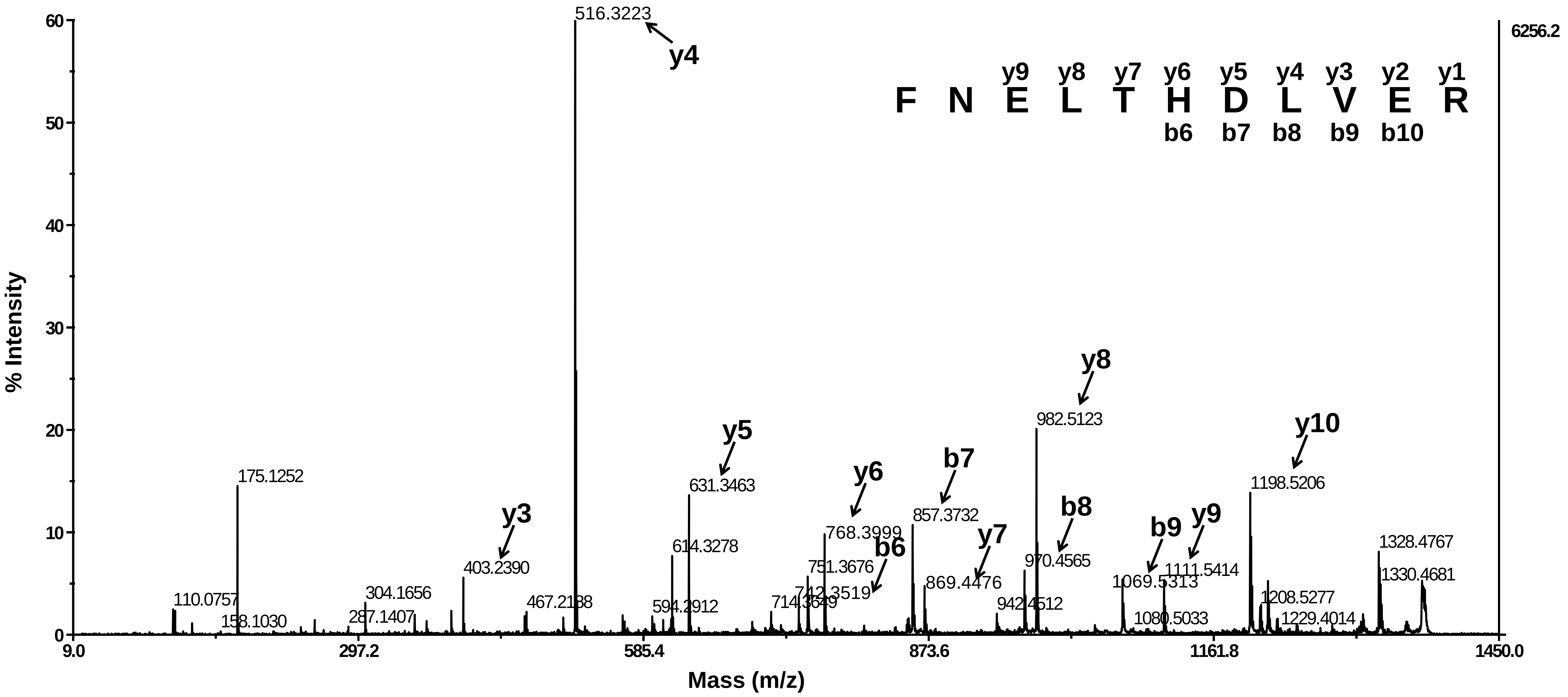

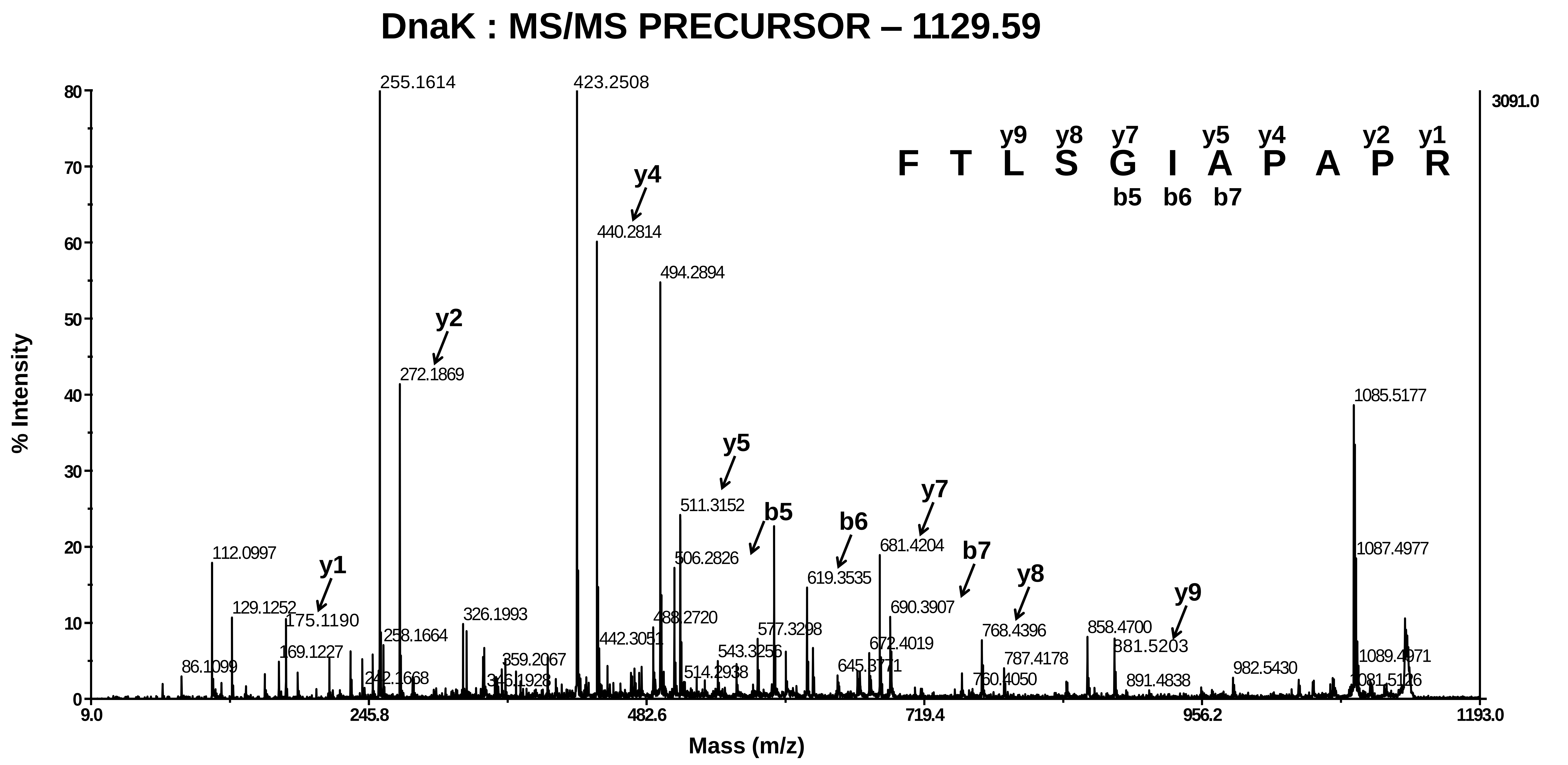
DnaK : MS/MS PRECURSOR – 1129.59
% Intensity
Mass (m/z)
3091.0
F T L S G I A P A P R
y9 y8 y7 y5 y4 y2 y1
b5 b6 b7
255.1614
423.2508
440.2814
494.2894
272.1869
1085.5177
1087.4977
511.3152
506.2826
681.4204
619.3535
690.3907
112.0997
129.1252
175.1190
326.1993
258.1664
488.2720
442.3051
577.3298
543.3256
672.4019
768.4396
858.4700
881.5203
169.1227
86.1099
359.2067
242.1668
346.1928
514.2938
645.3771
787.4178
760.4050
891.4838
982.5430
1089.4971
1081.5126
y1
y2
y4
y5
b5
b6
y7
b7
y8
y9

DnaK : MS/MS PRECURSOR – 1894.87

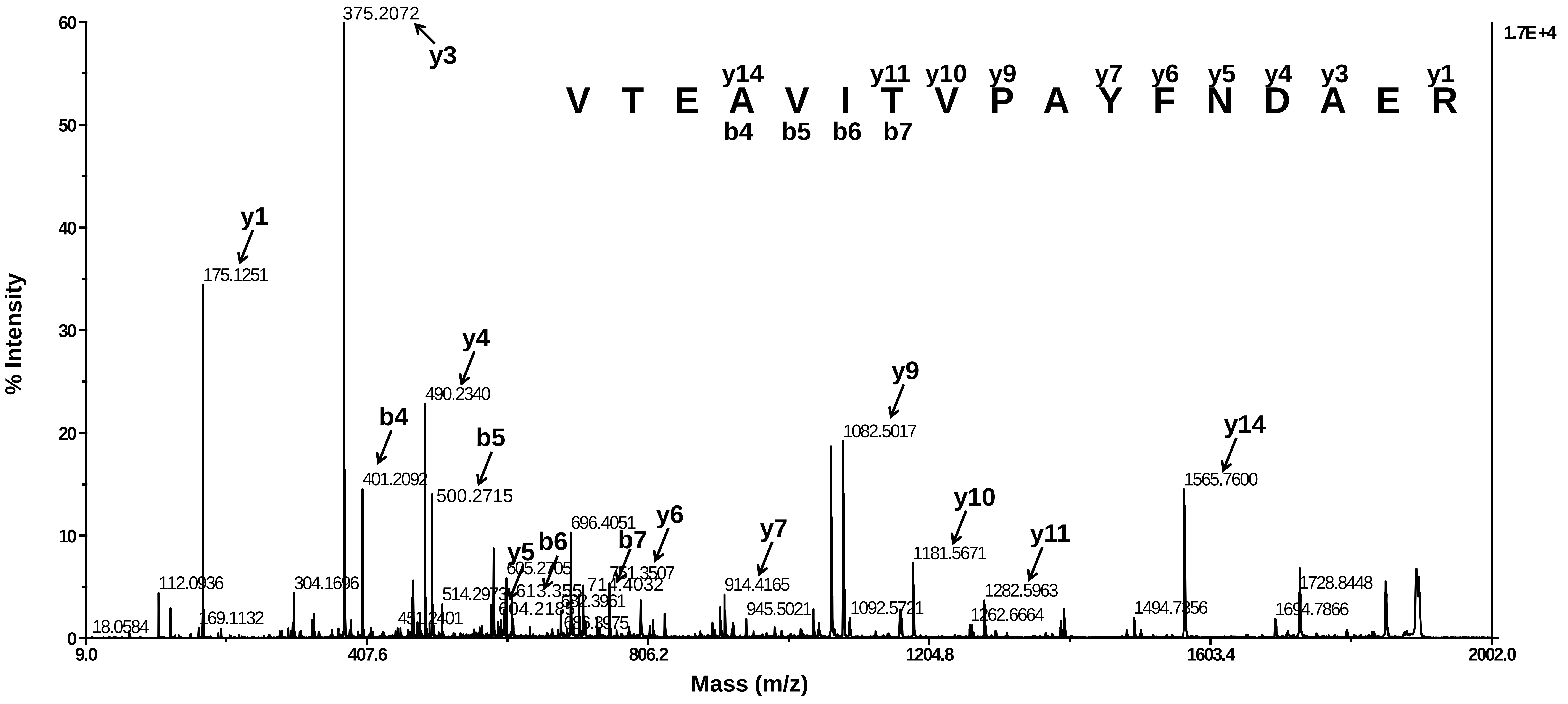

MS and MS/MS spectra of tryptic peptides obtained from EF-Tu from *C. tetani*

EF-Tu: MS SPECTRUM

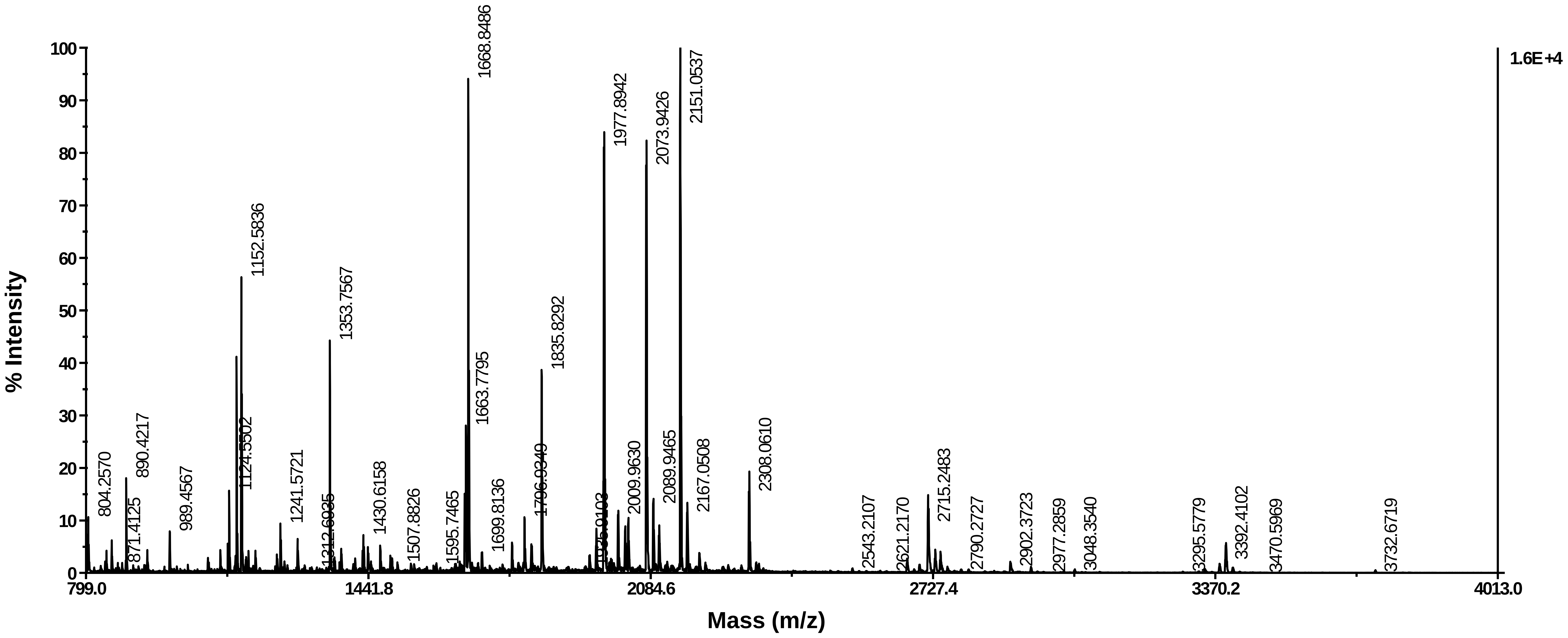

EF-Tu : MS/MS PRECURSOR – 1796.94

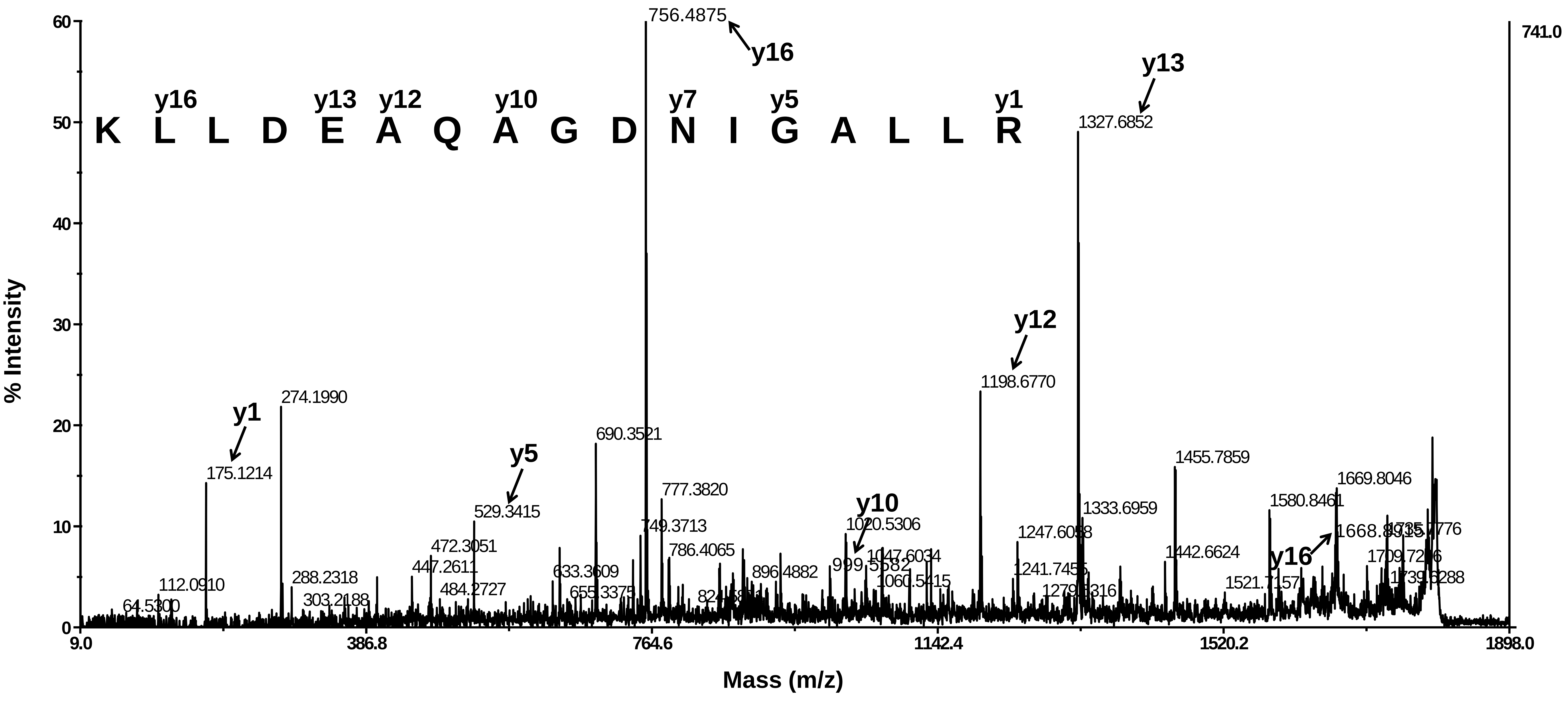

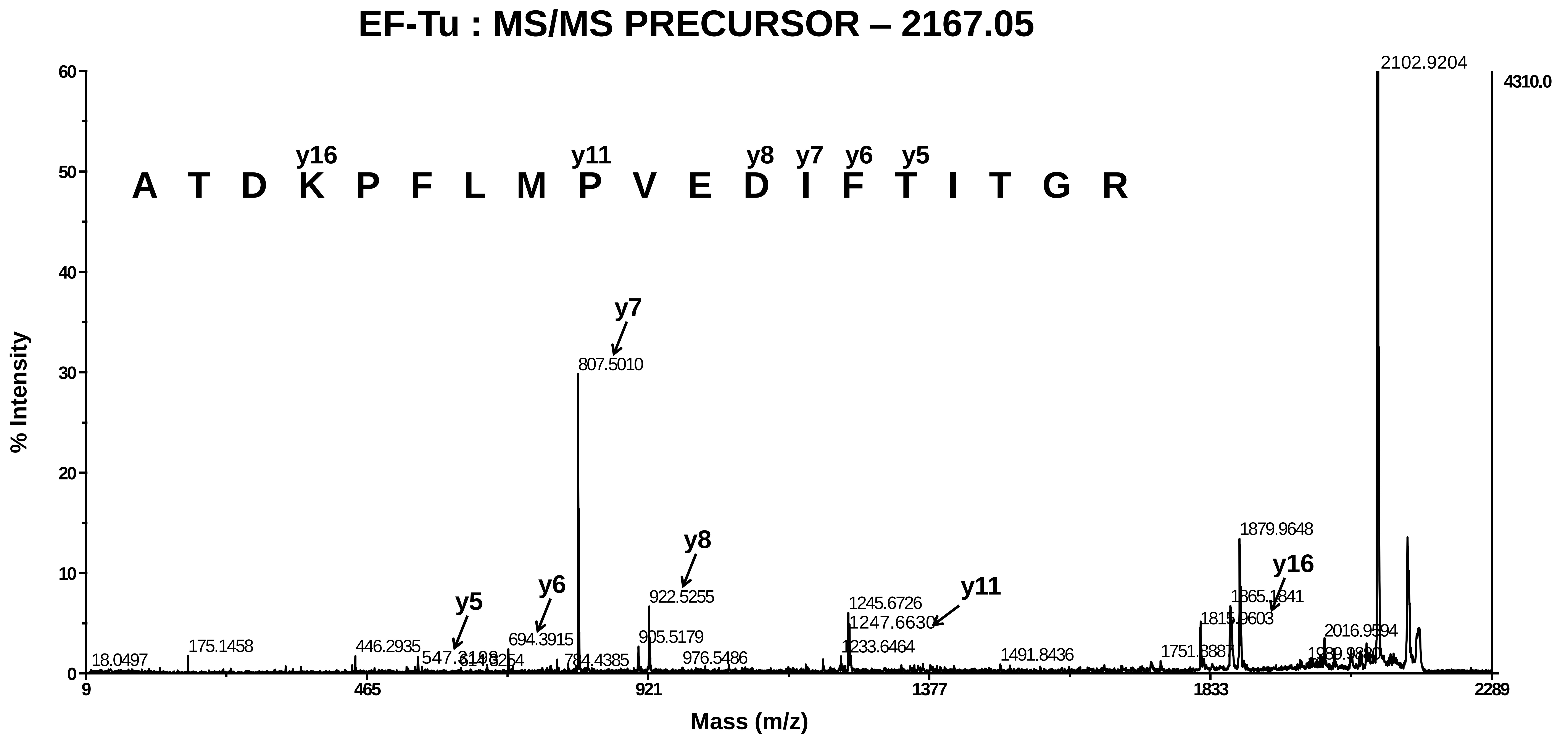
EF-Tu : MS/MS PRECURSOR – 2167.05
A T D K P F L M P V E D I F T I T G R
y16 y11 y8 y7 y6 y5
% Intensity
Mass (m/z)
0
10
20
30
40
50
60
9
465
921
1377
1833
2289
4310.0
2102.9204
807.5010
922.5255
905.5179
976.5486
1245.6726
1247.6630
1233.6464
1491.8436
1751.8887
1815.9603
1865.1841
1879.9648
2016.9594
175.1458
446.2935
694.3915
784.4385
18.0497
y5
y6
y7
y8
y11
y16

EF-Tu : MS/MS PRECURSOR – 2089.95

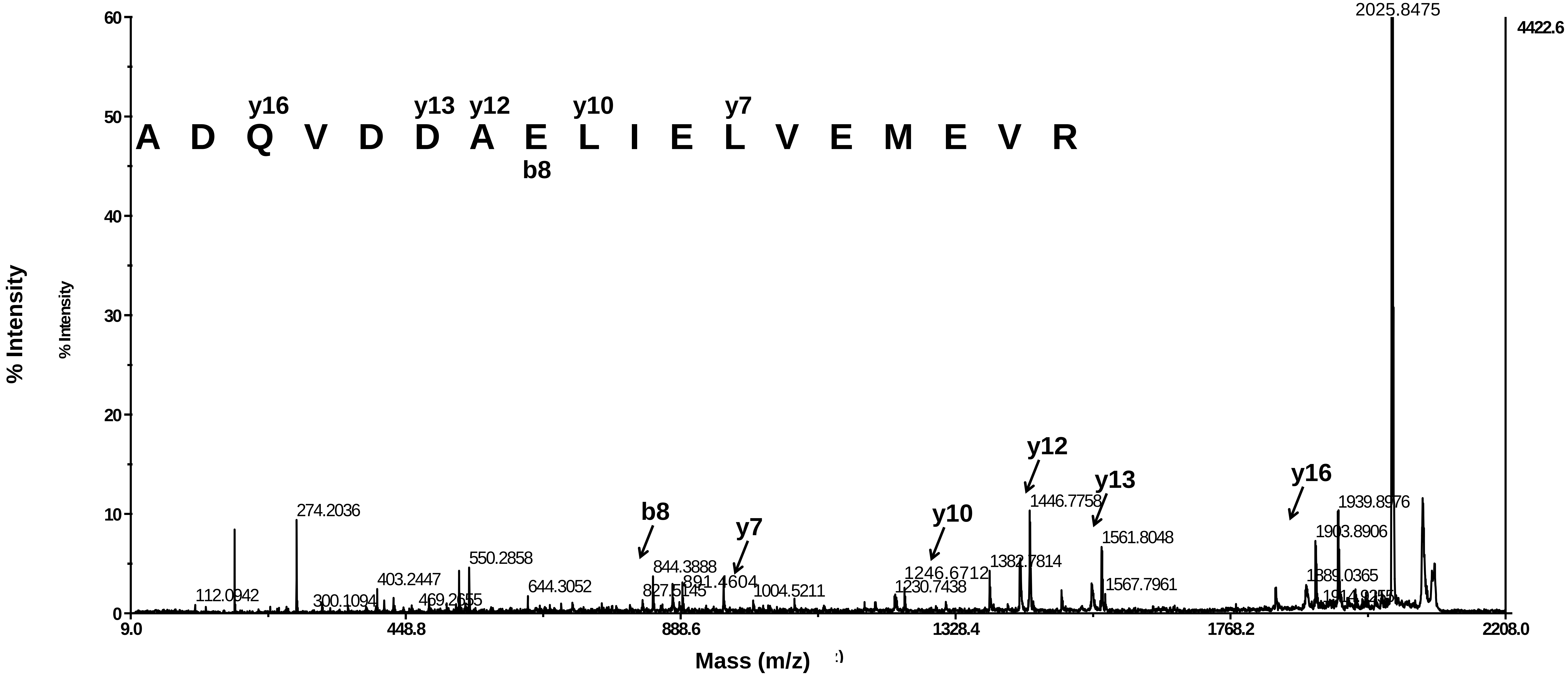

EF-Tu : MS/MS PRECURSOR – 1141.6

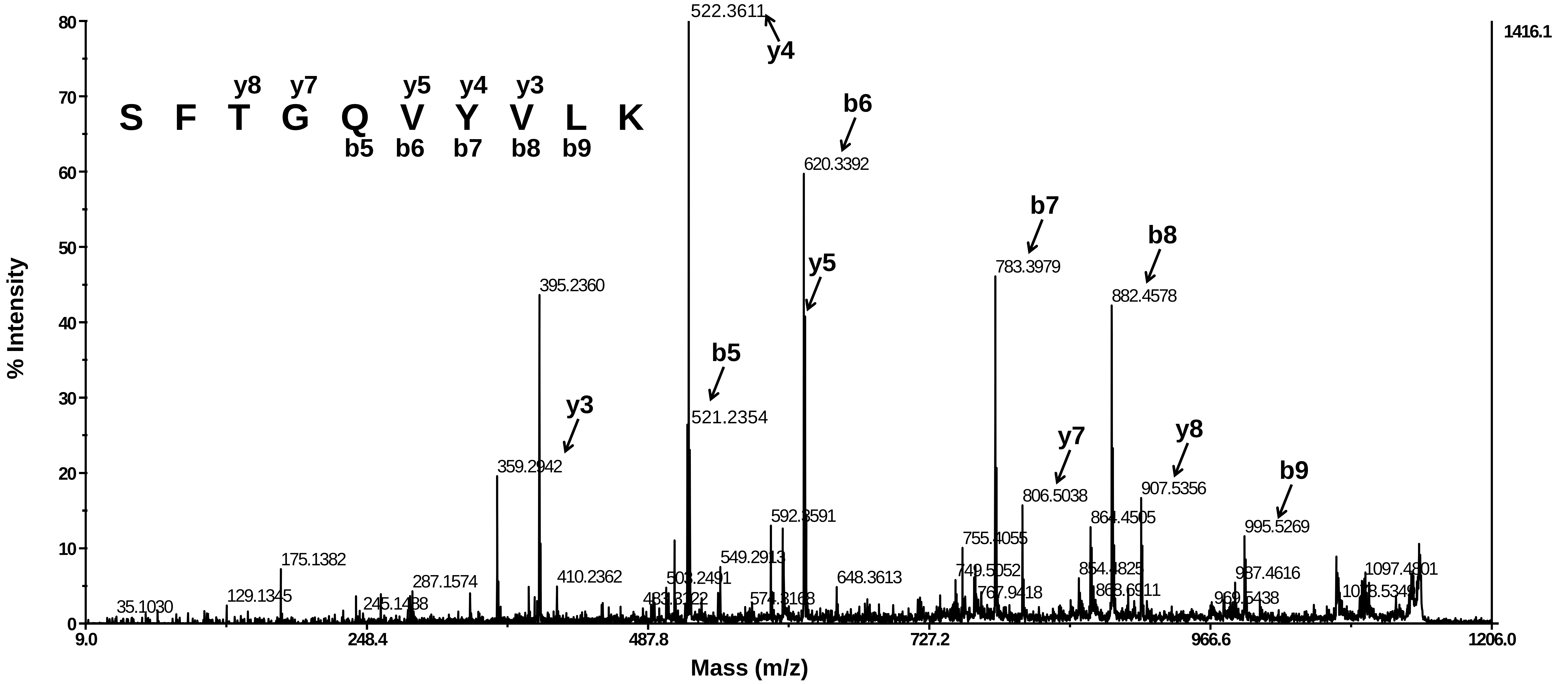

EF-Tu : MS/MS PRECURSOR – 2308.06

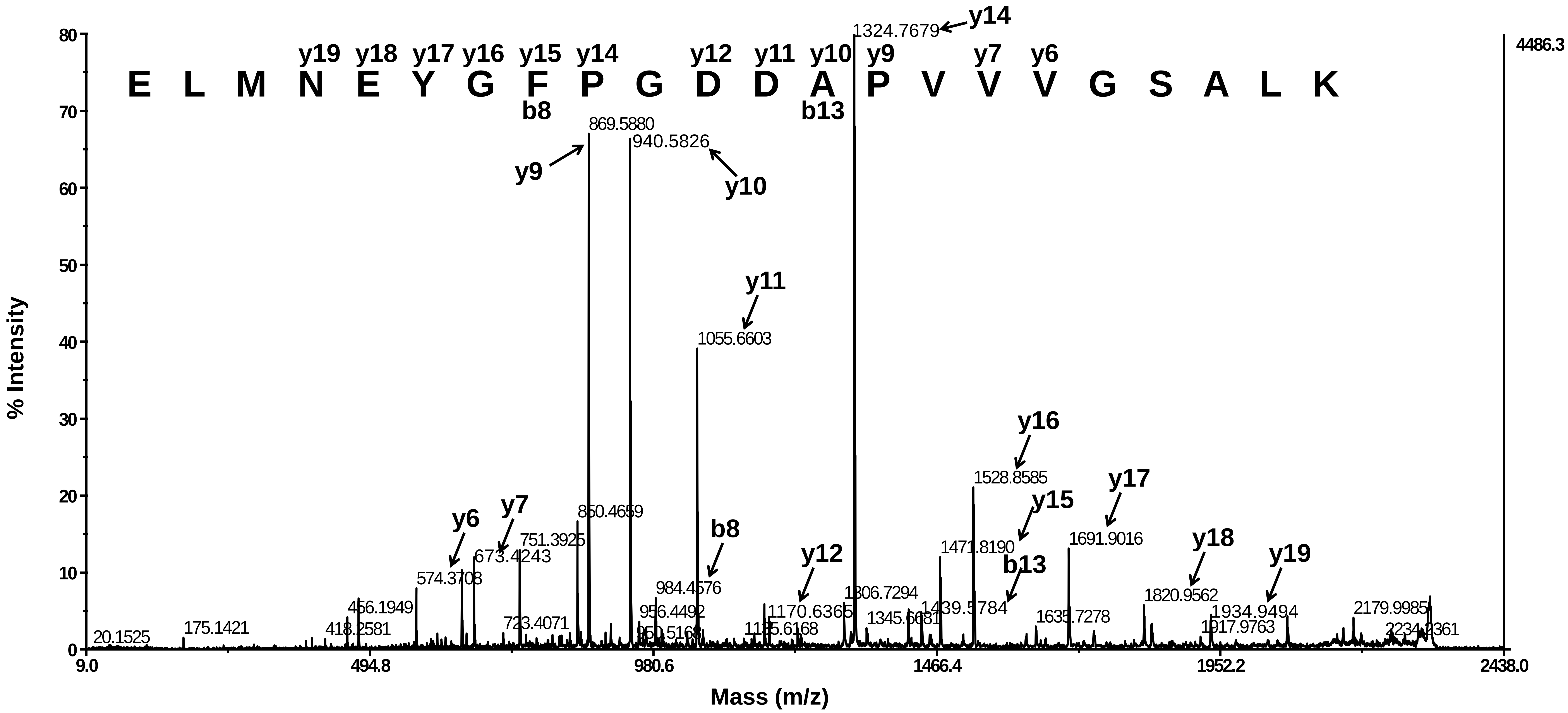

EF-Tu : MS/MS PRECURSOR – 1353.76

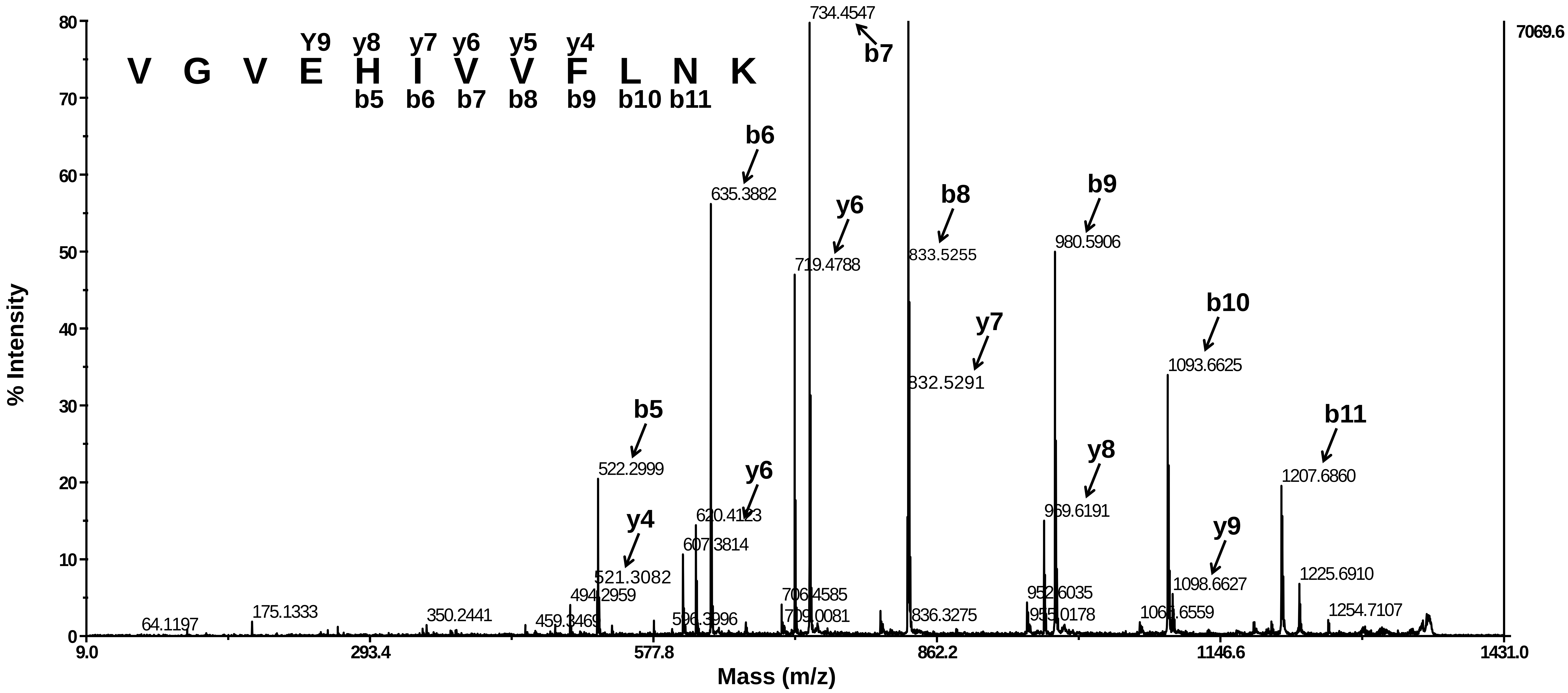

EF-Tu : MS/MS PRECURSOR – 2715.25

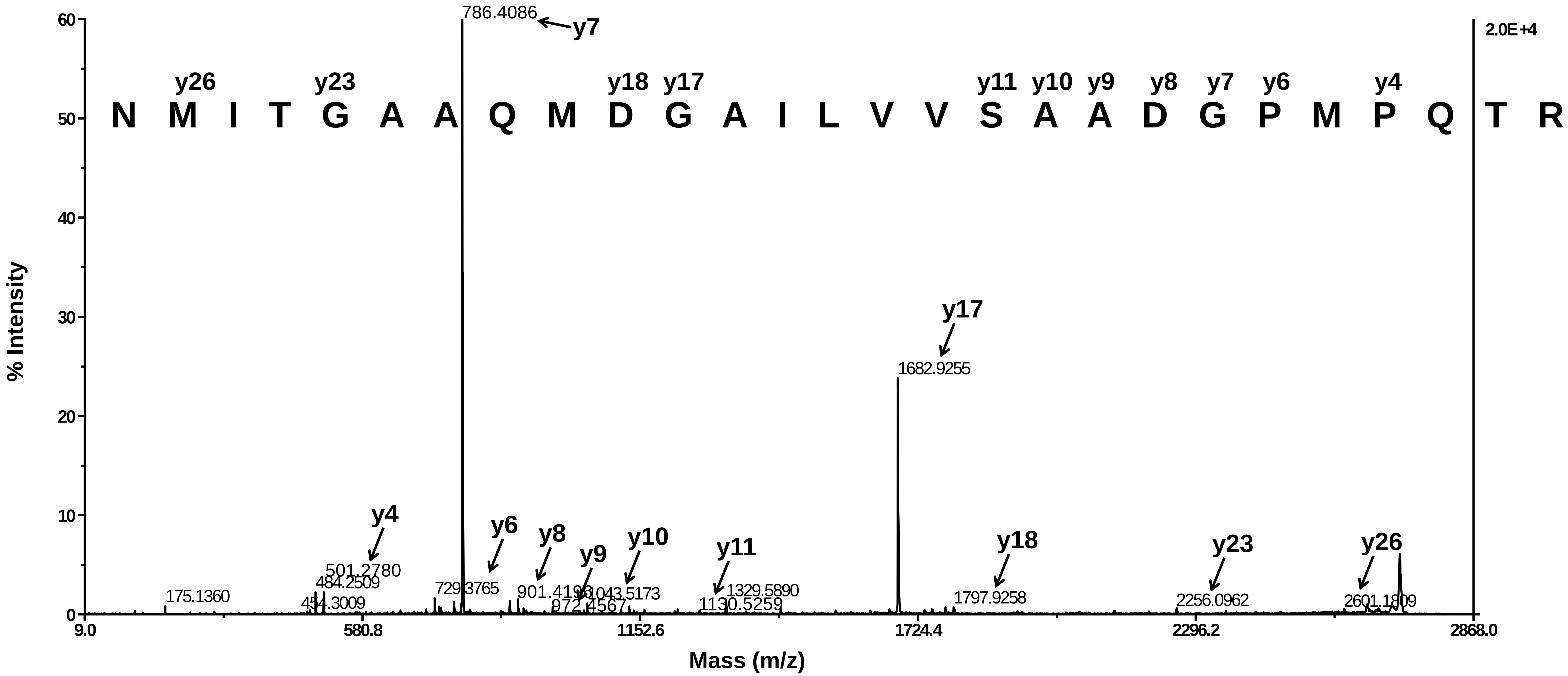

EF-Tu : MS/MS PRECURSOR – 1152.58

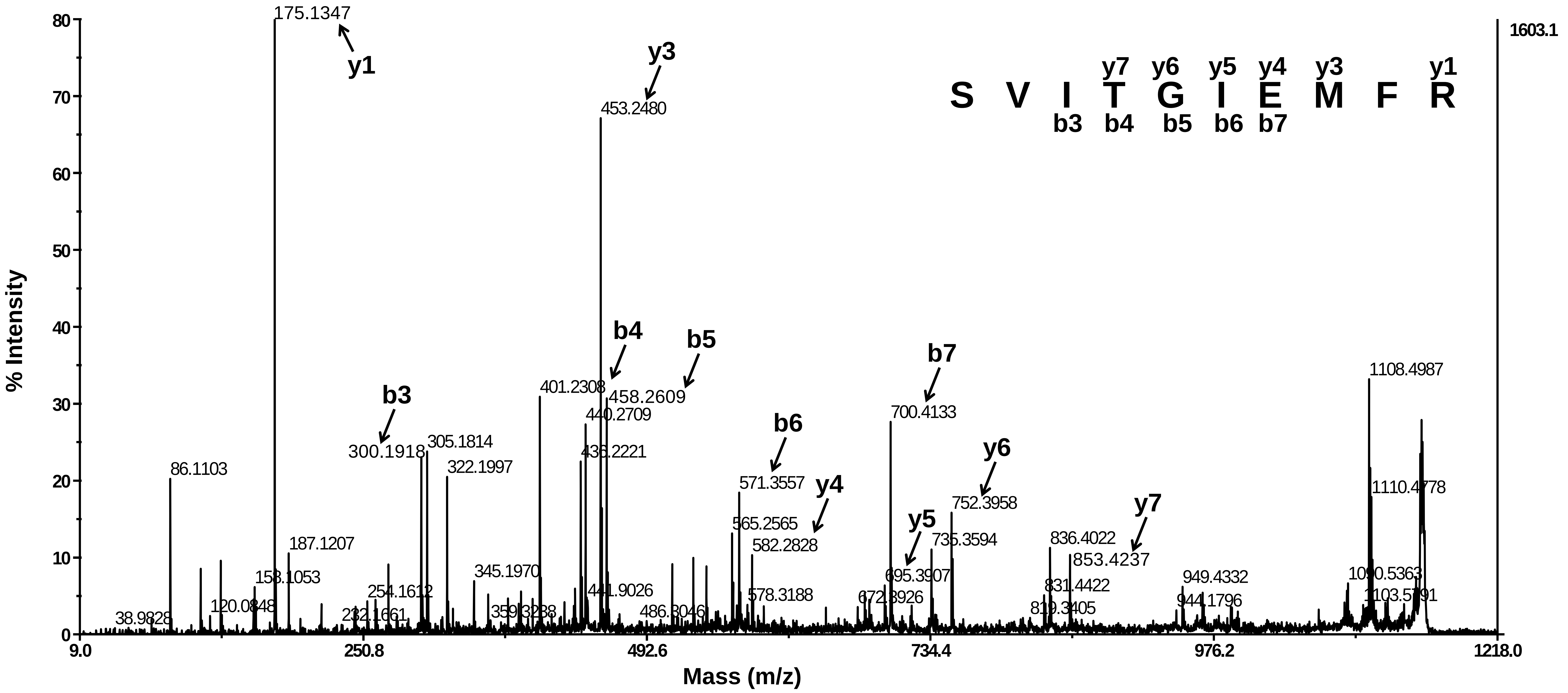

EF-Tu : MS/MS PRECURSOR – 1835.83

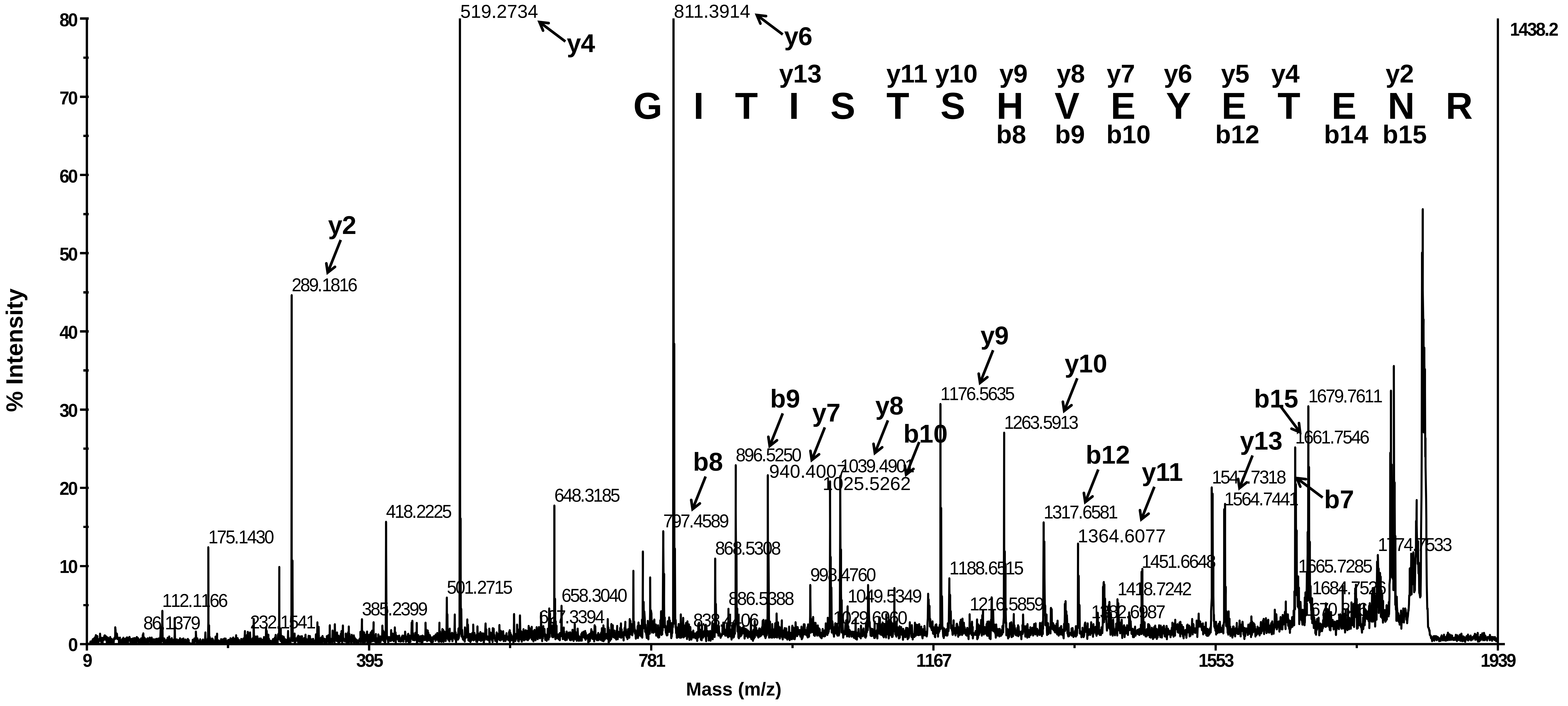

EF-Tu : MS/MS PRECURSOR – 1668.85

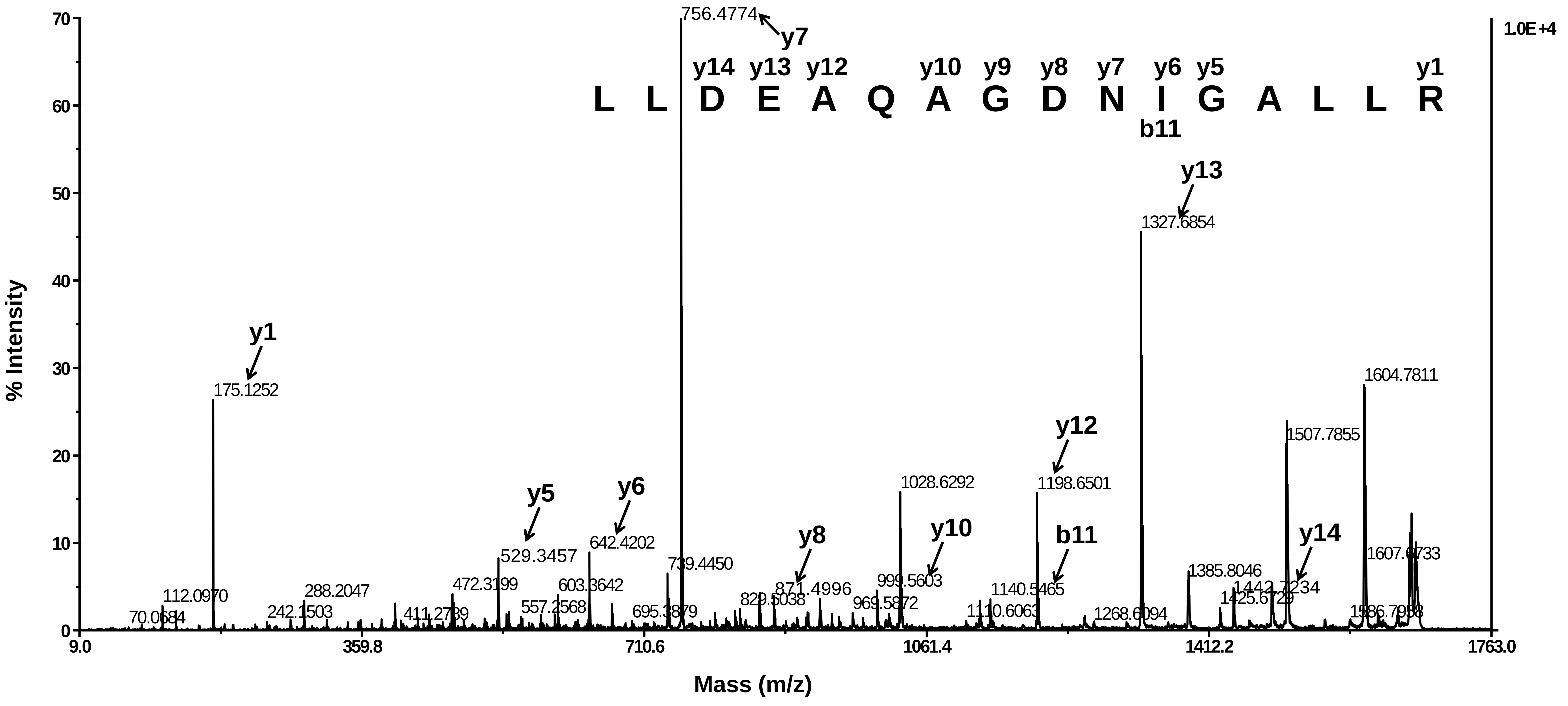

EF-Tu : MS/MS PRECURSOR – 1977.89

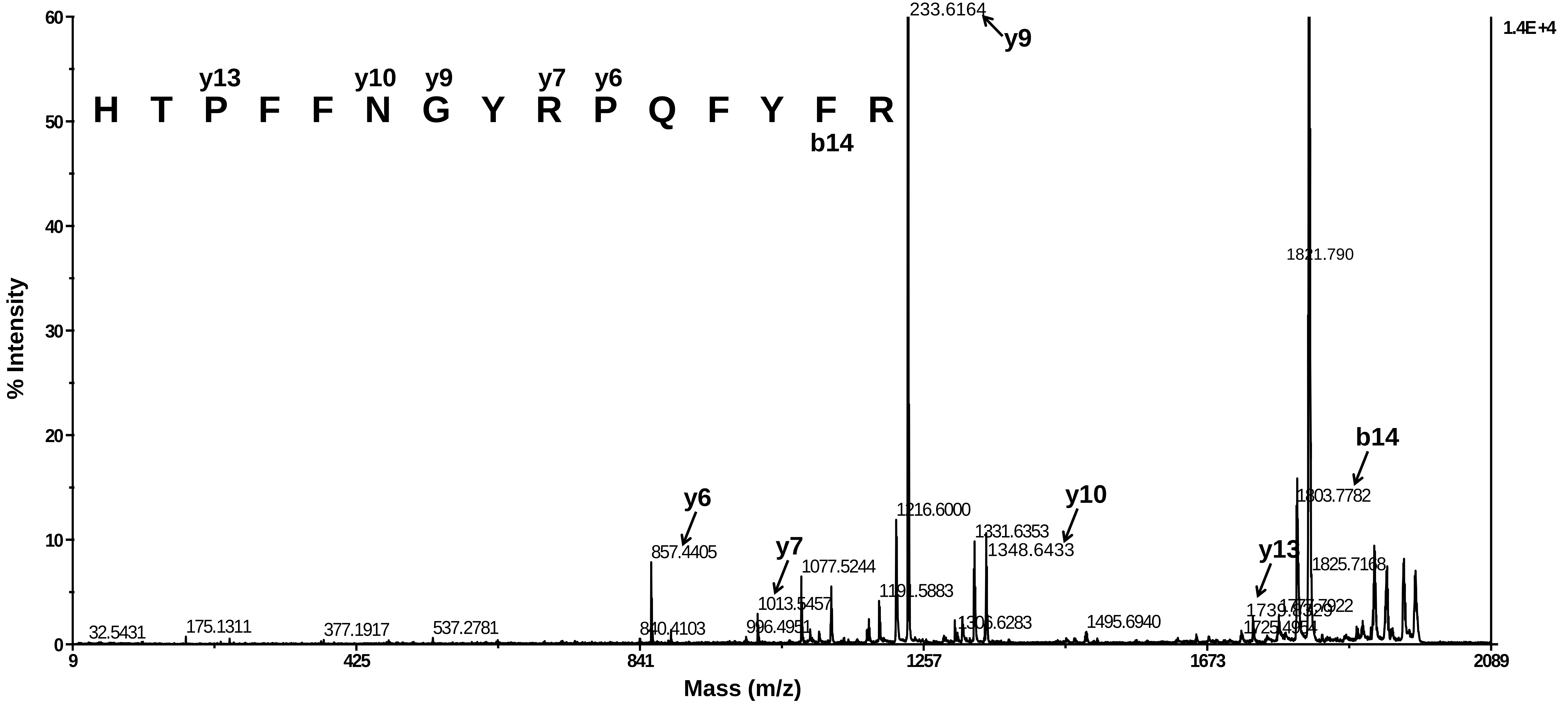

EF-Tu : MS/MS PRECURSOR – 2073.94

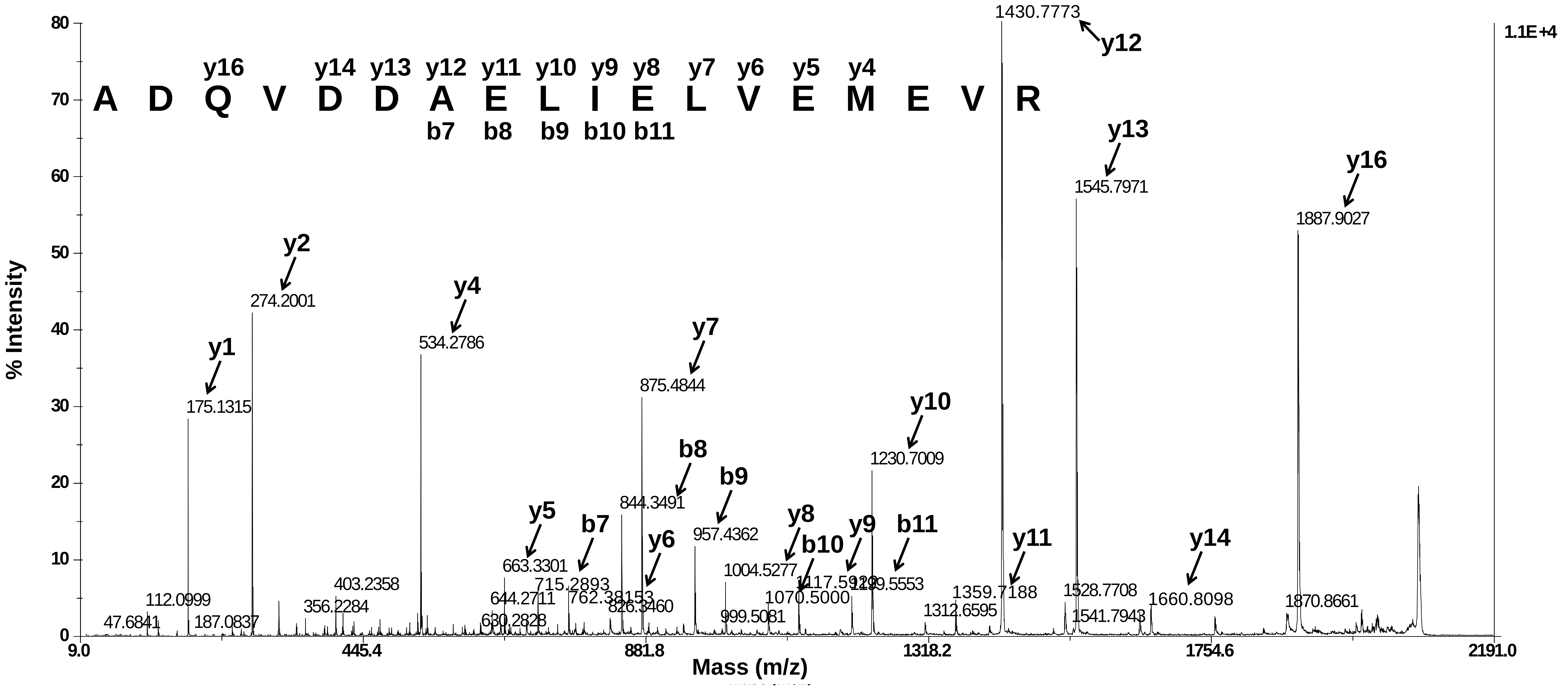

EF-Tu : MS/MS PRECURSOR – 2151.05

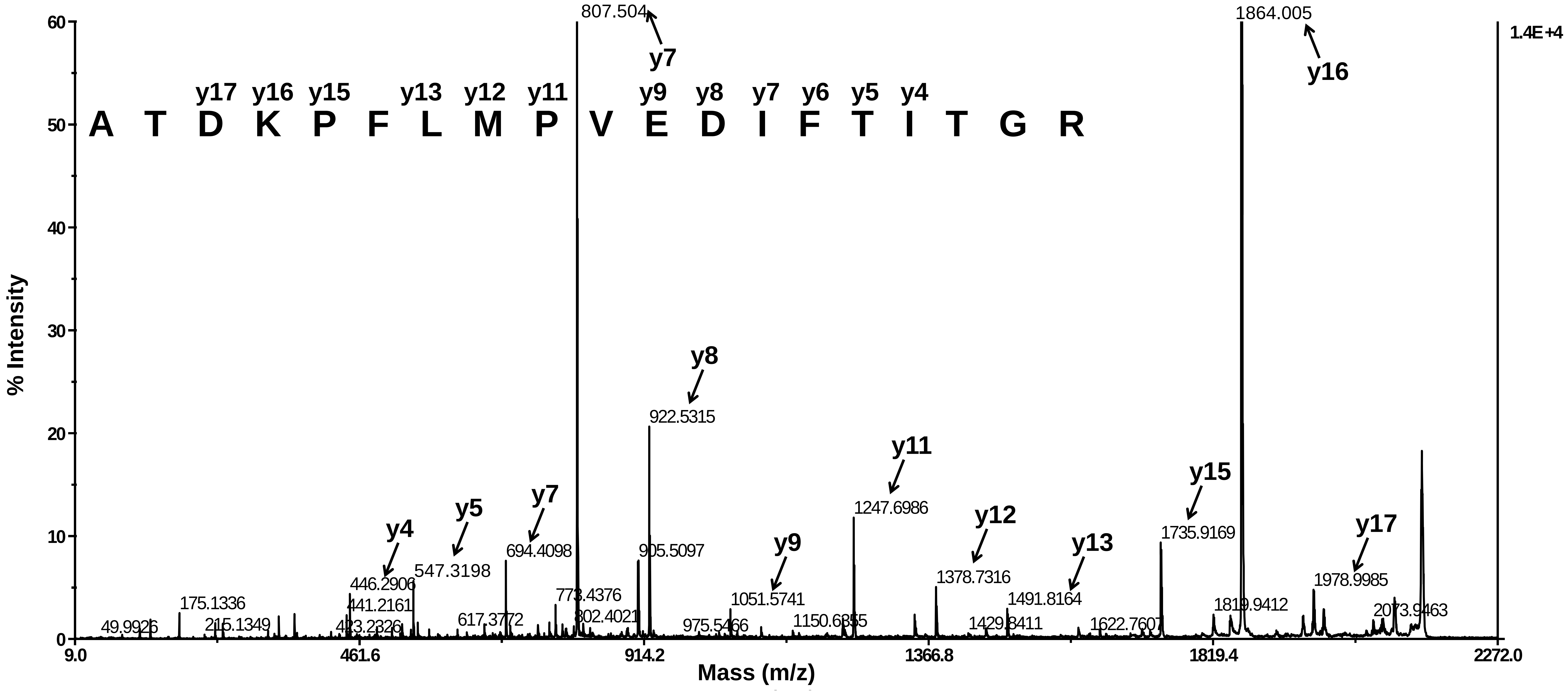

MS and MS/MS spectra of tryptic peptides obtained from GroEL from *B. anthracis*

GroEL: MS SPECTRUM

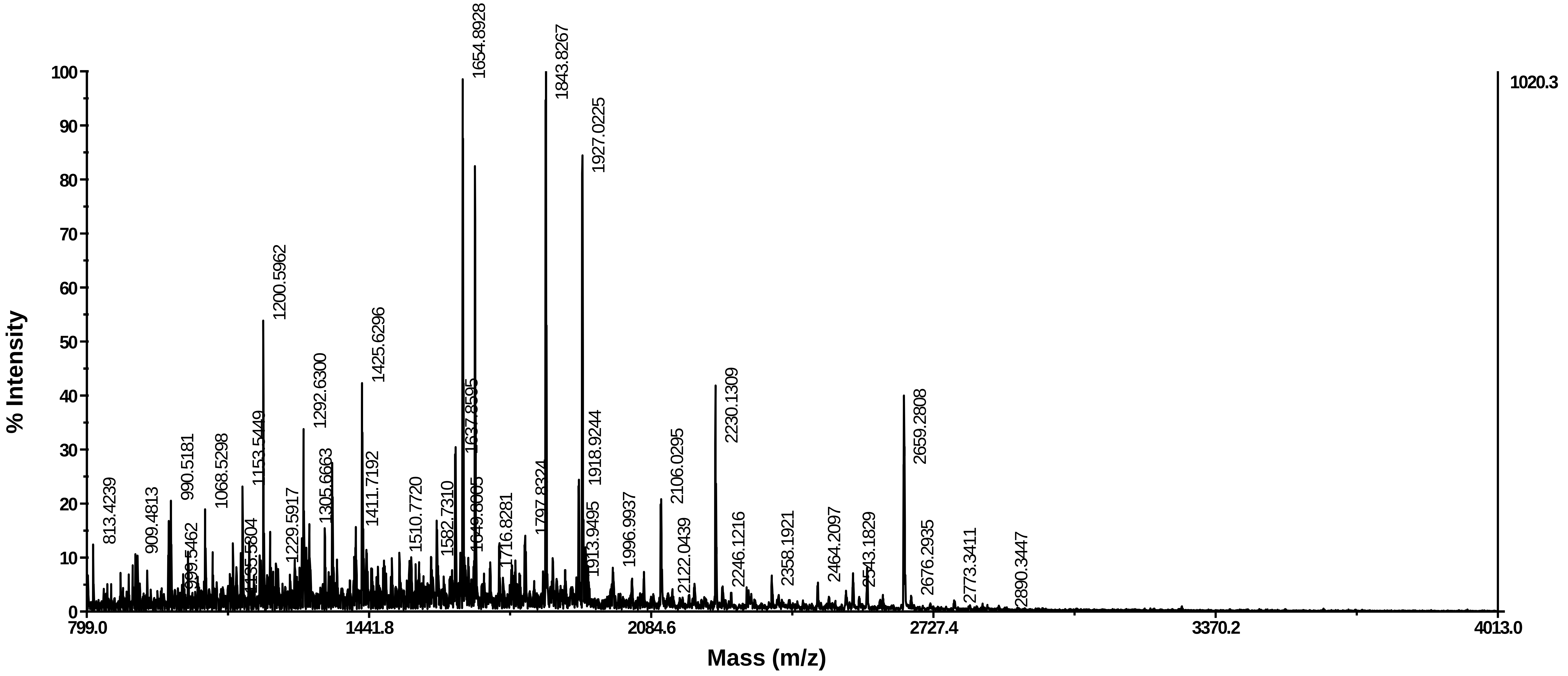

GroEL: MS/MS PRECURSOR – 1595.72

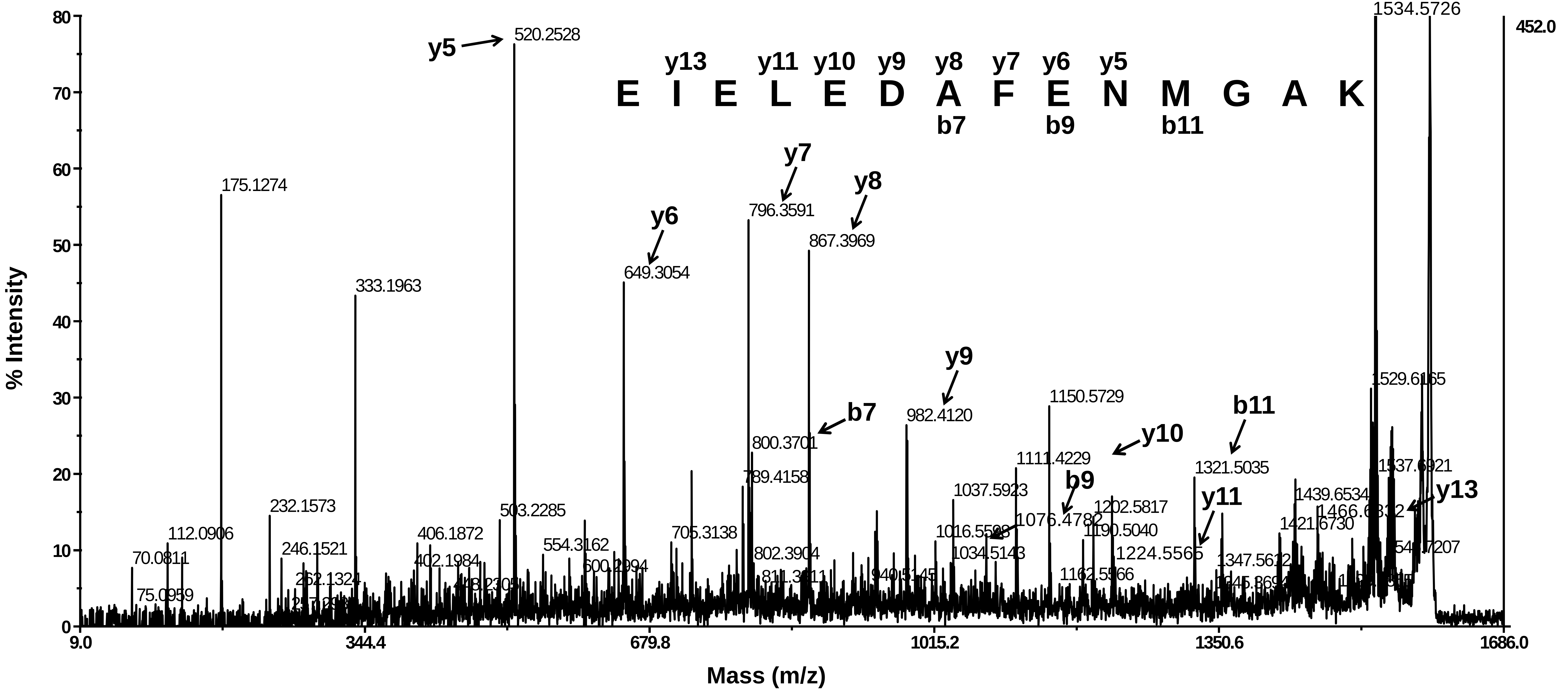

GroEL: MS/MS PRECURSOR – 2106.03

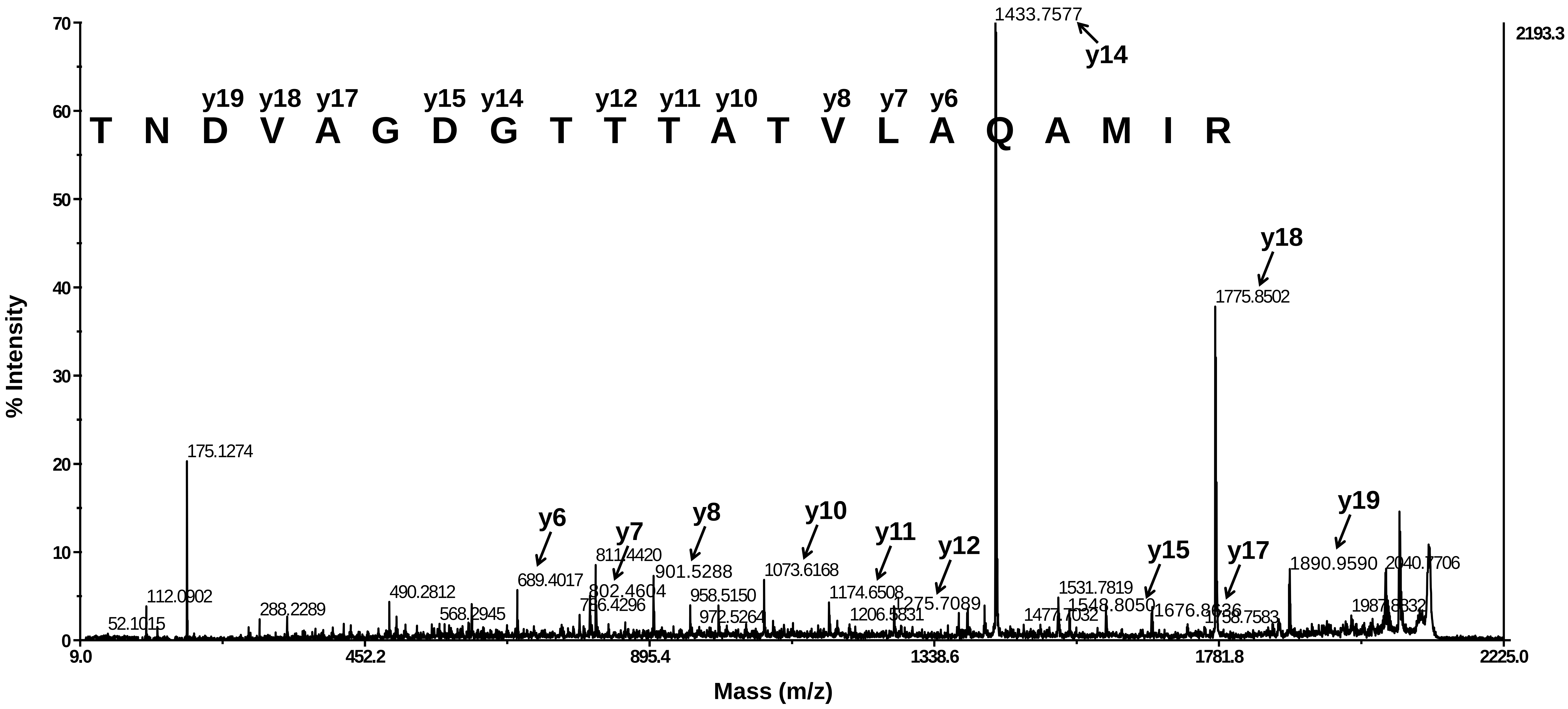

GroEL: MS/MS PRECURSOR – 1425.63

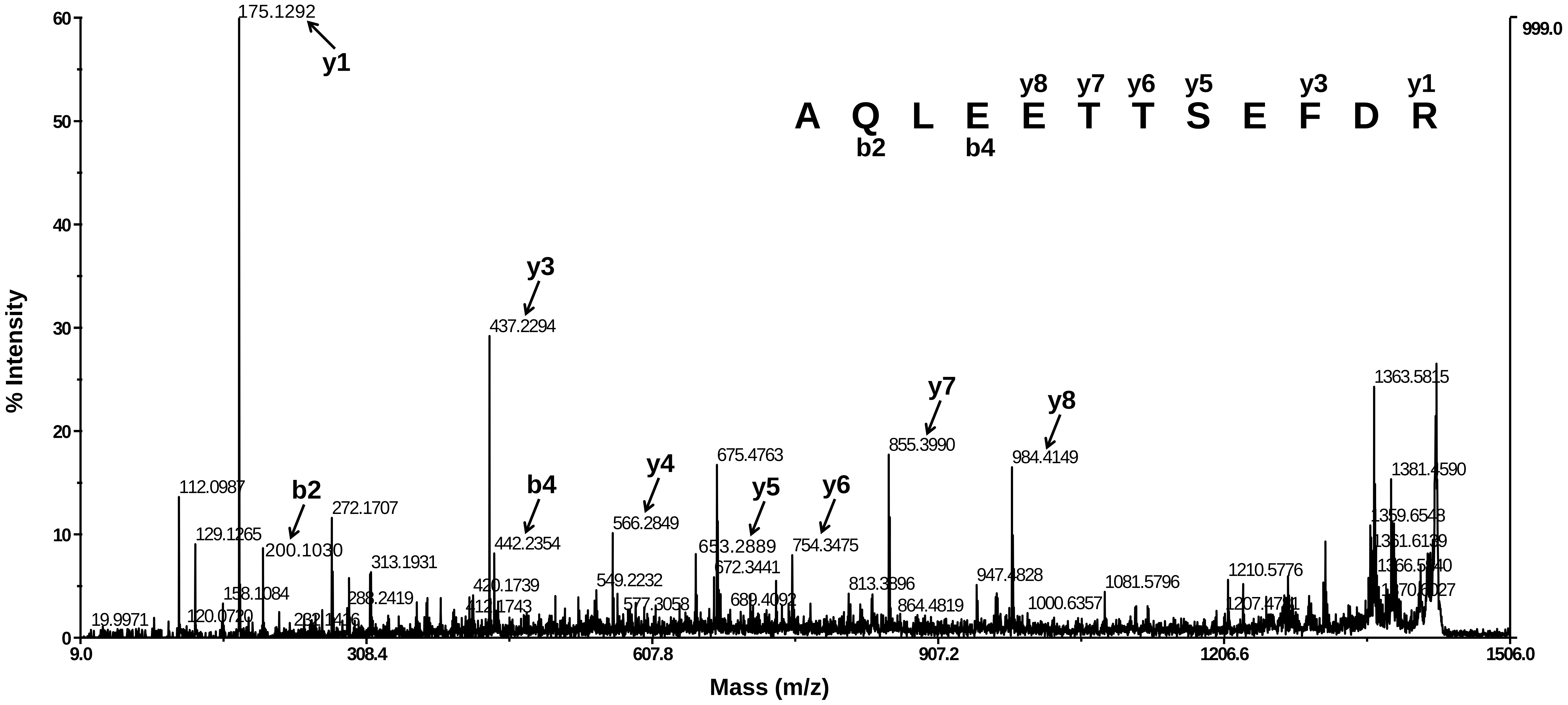

GroEL: MS/MS PRECURSOR – 1200.6

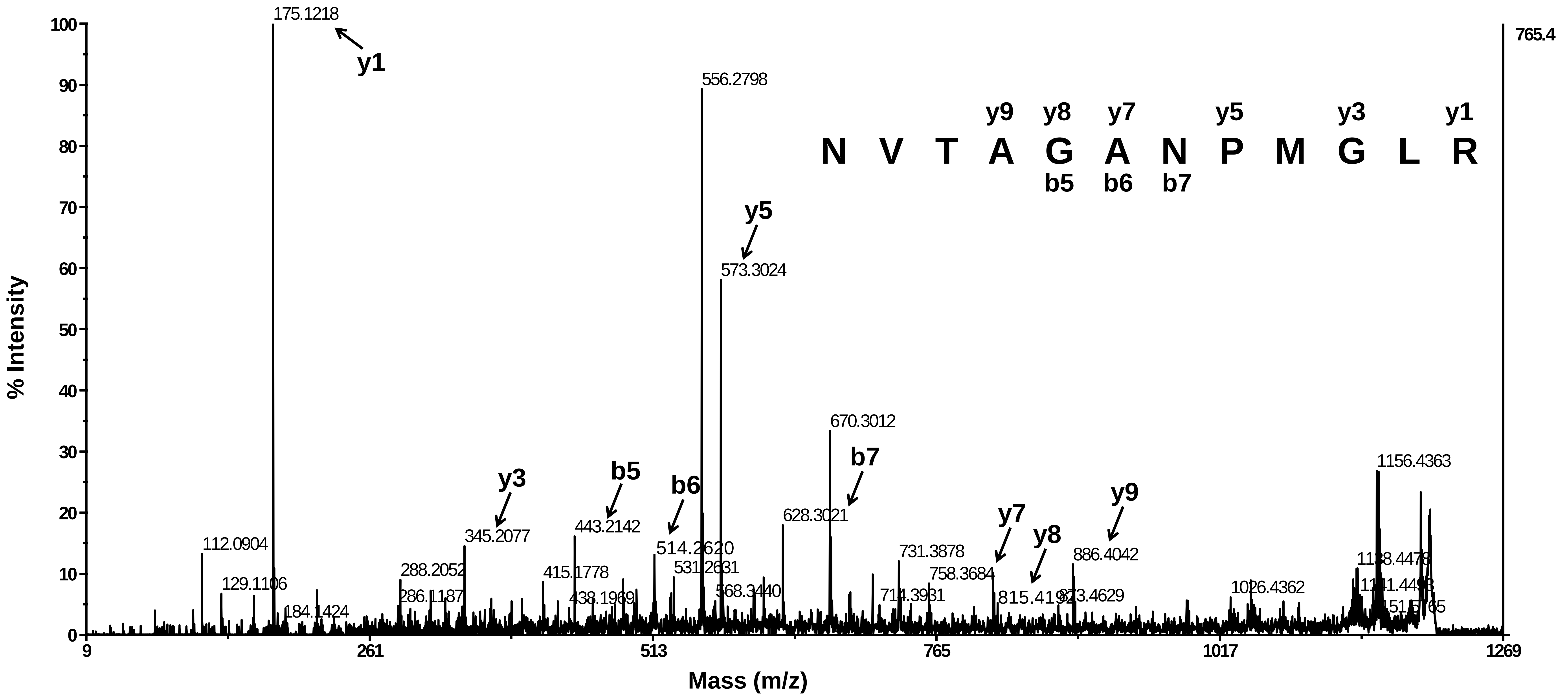

GroEL: MS/MS PRECURSOR – 2230.13

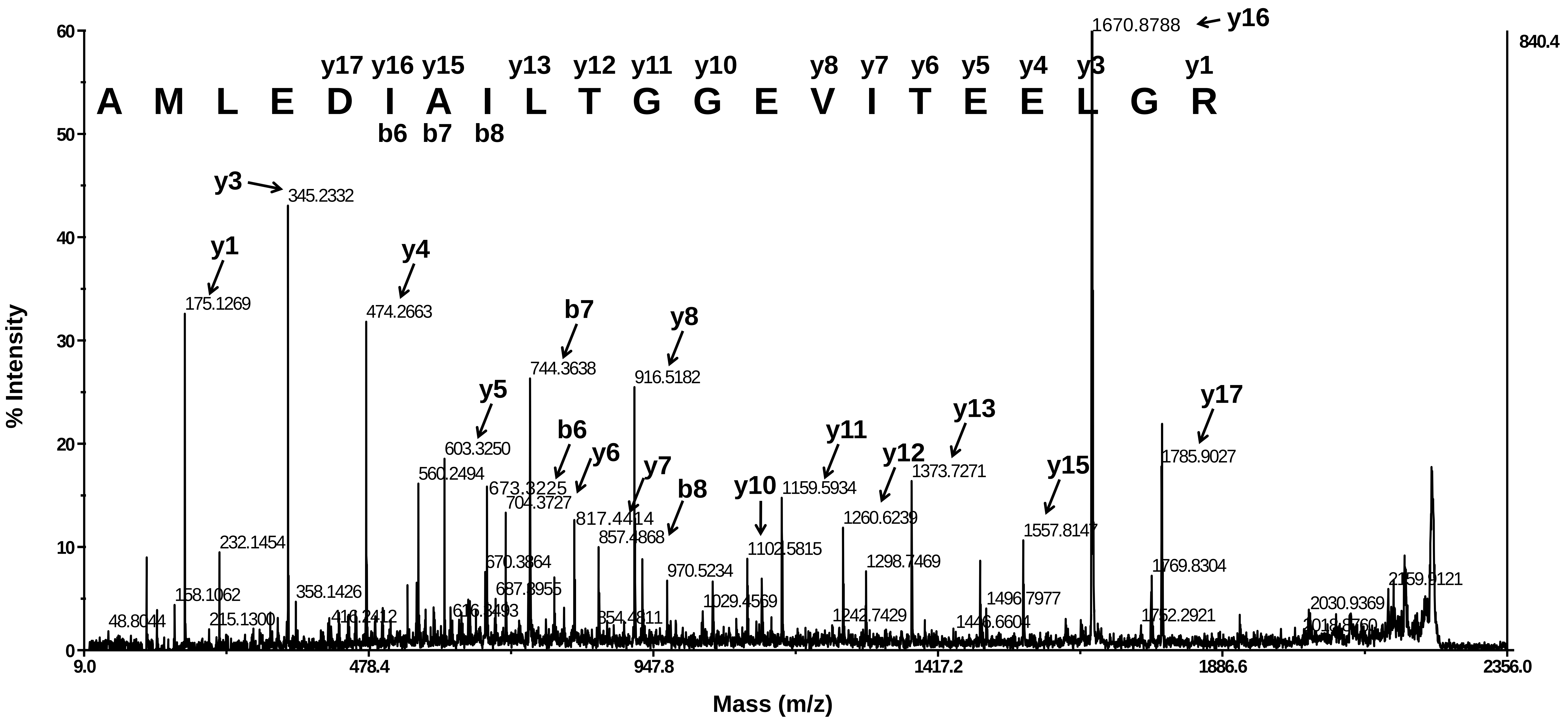

GroEL: MS/MS PRECURSOR – 2659.28

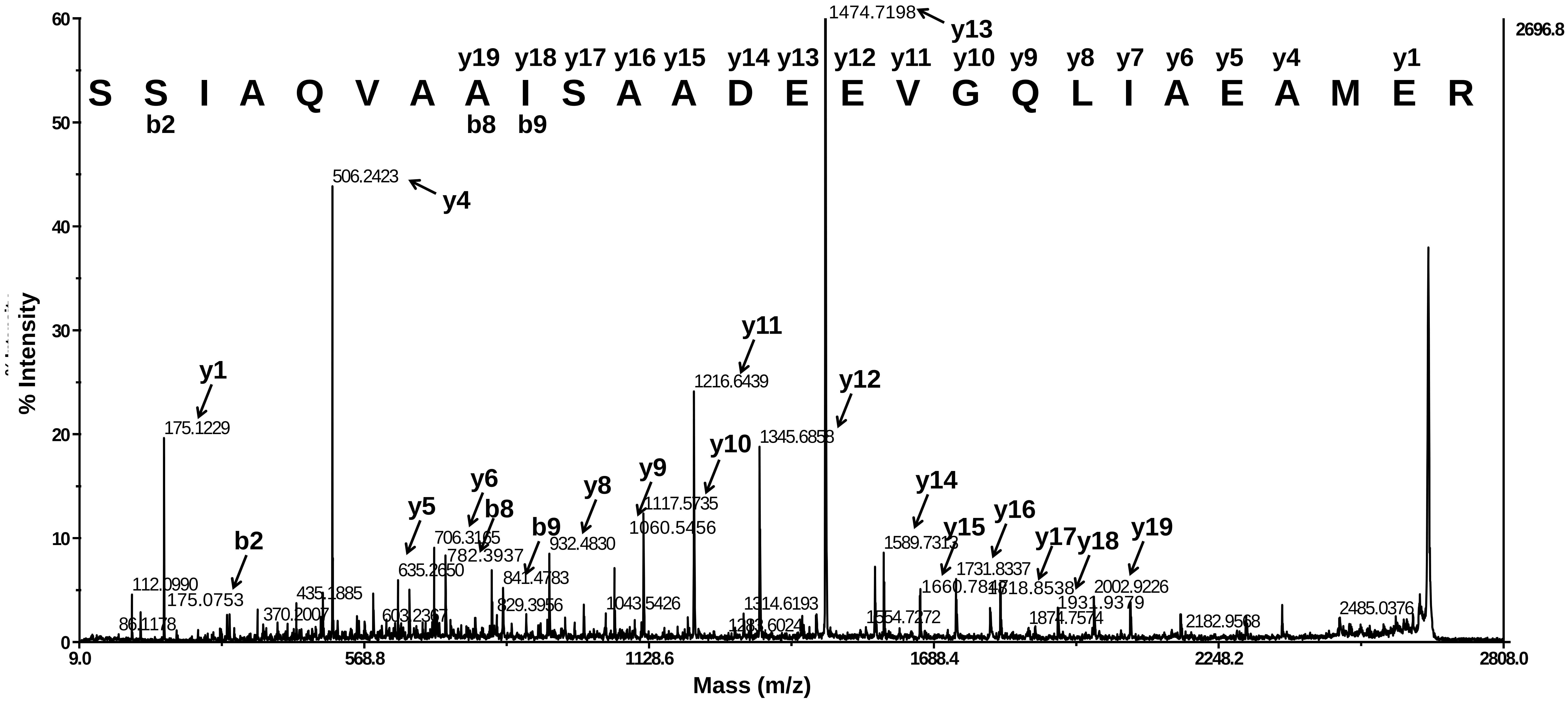

GroEL: MS/MS PRECURSOR – 1682.77

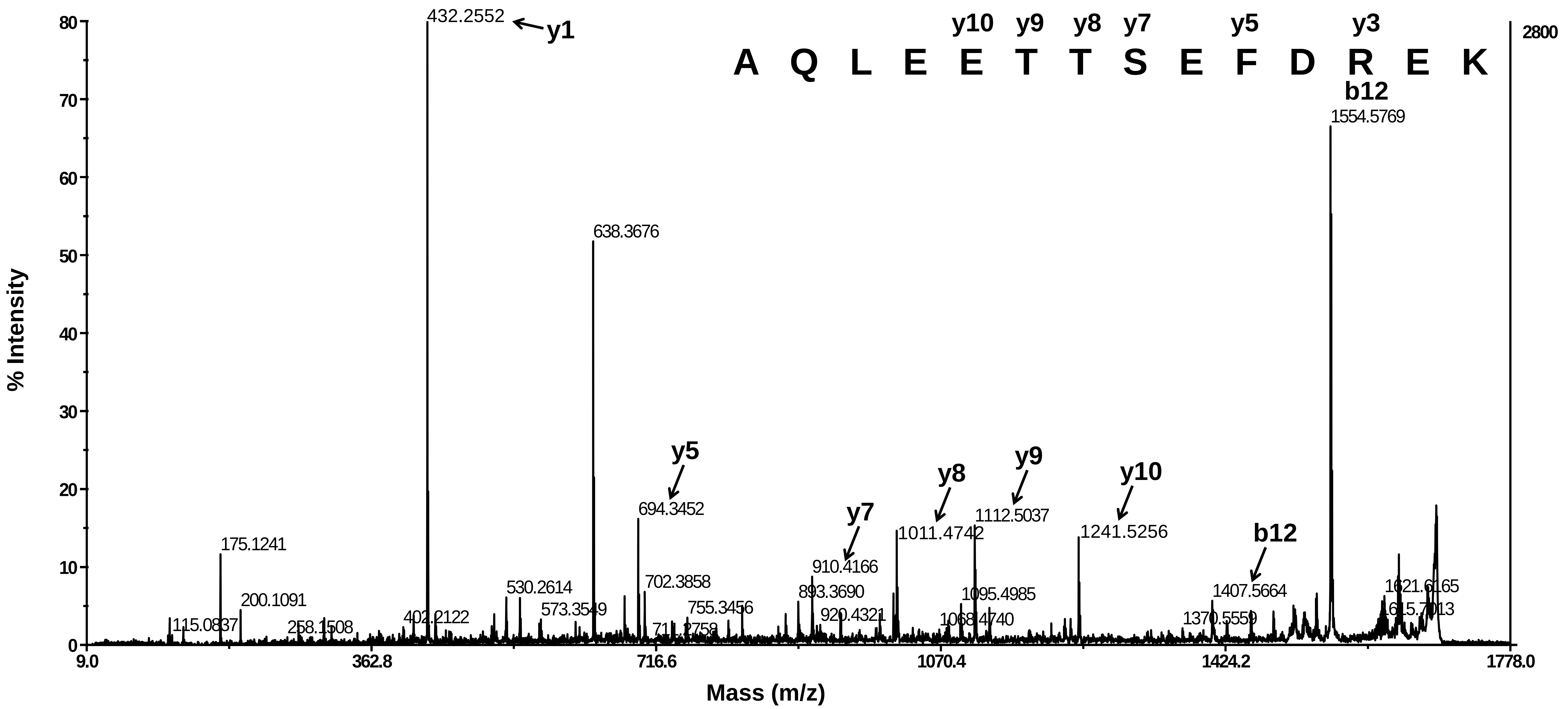

GroEL: MS/MS PRECURSOR – 1654.89

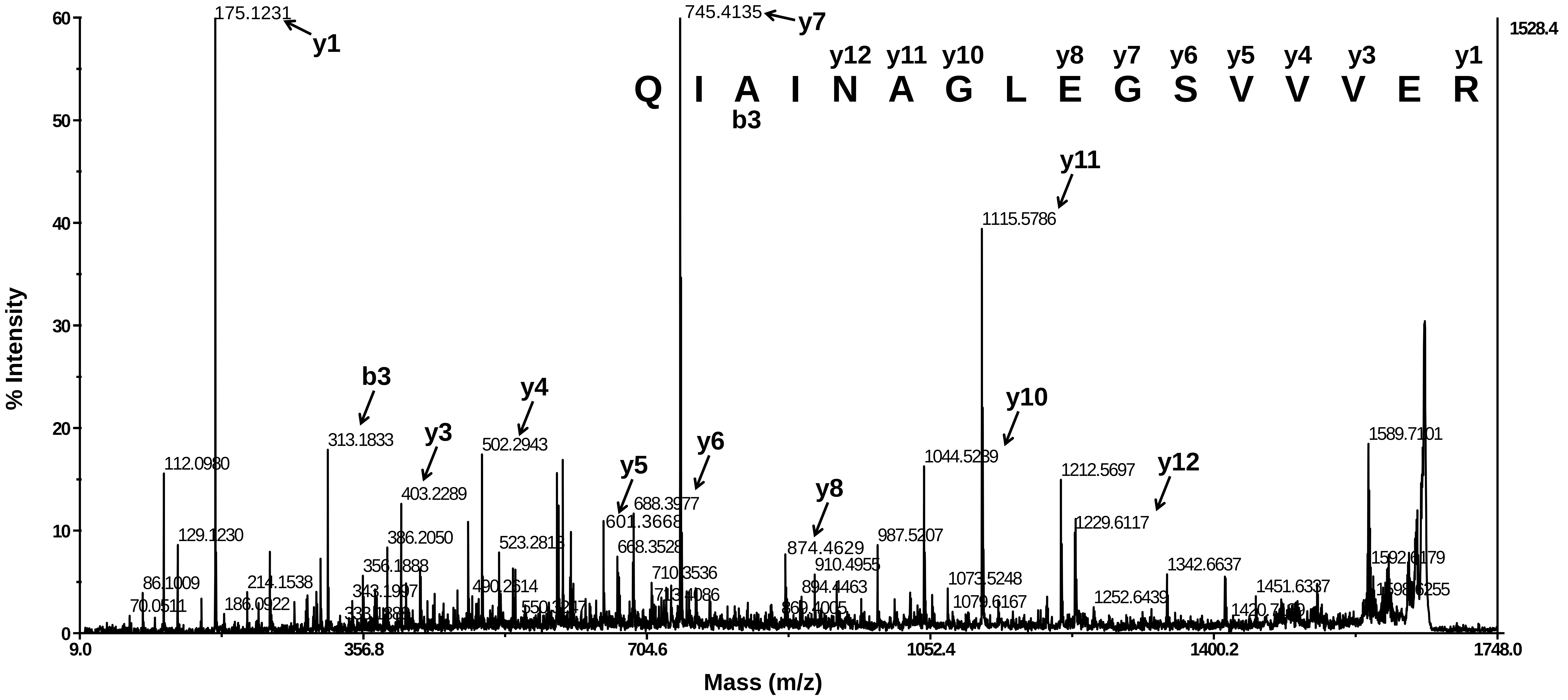

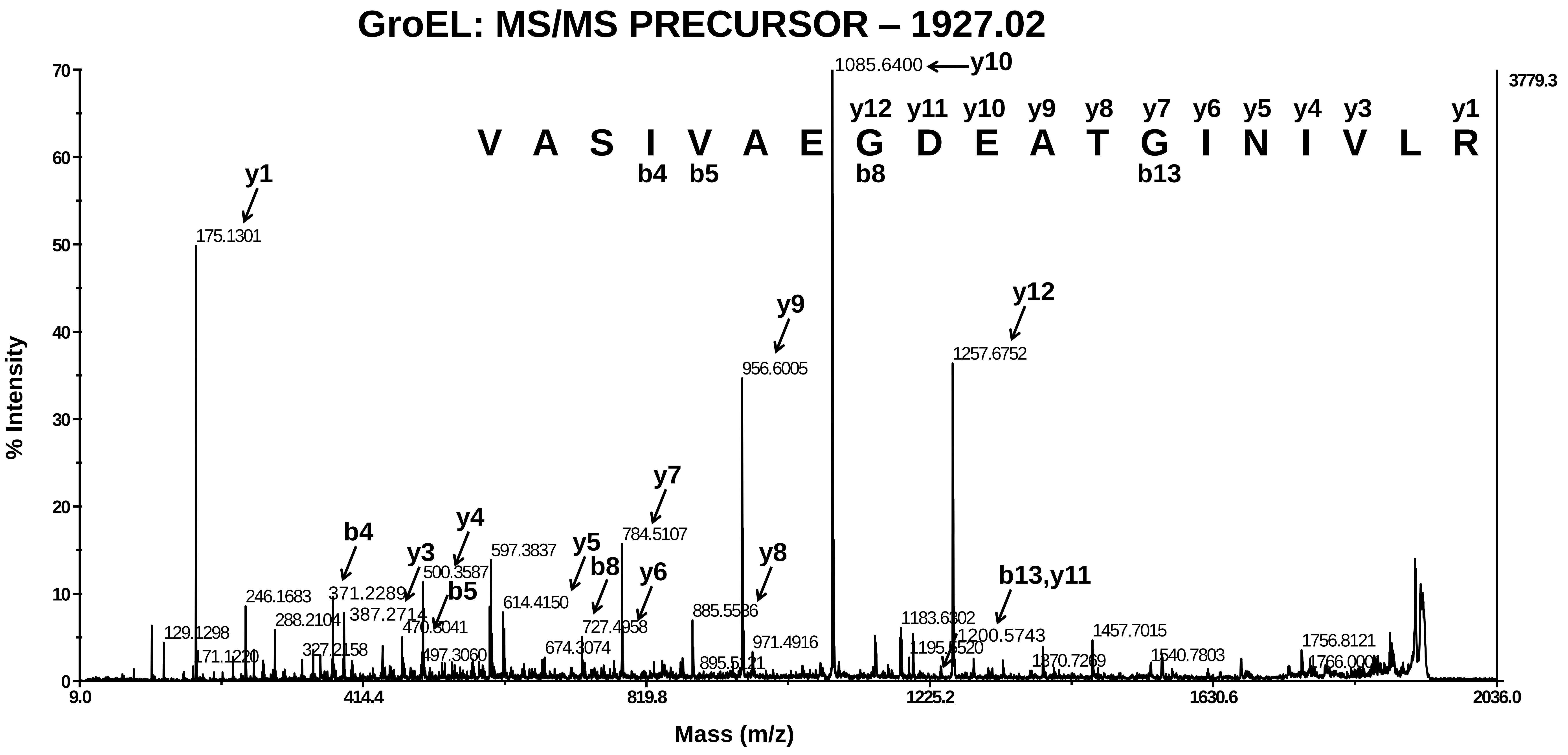

GroEL: MS/MS PRECURSOR – 1927.02
% Intensity
Mass (m/z)
3779.3
V A S I V A E G D E A T G I N I V L R
y12 y11 y10 y9 y8 y7 y6 y5 y4 y3 y1
b4 b5 b8 b13
1085.6400 y10
y1 175.1301
y9 956.6005
y12 1257.6752
y7 784.5107
y4 500.3587
597.3837
b4 371.2289
y3 387.2714
b5 470.3041
y5
b8 727.4958
y6
y8 885.5536
b13,y11 1200.5743
614.4150
246.1683
288.2104
129.1298
171.1220
327.2158
497.3060
674.3074
895.5121
971.4916
1183.6302
1195.5520
1457.7015
1370.7269
1540.7803
1756.8121
1766.000

GroEL: MS/MS PRECURSOR – 1843.83

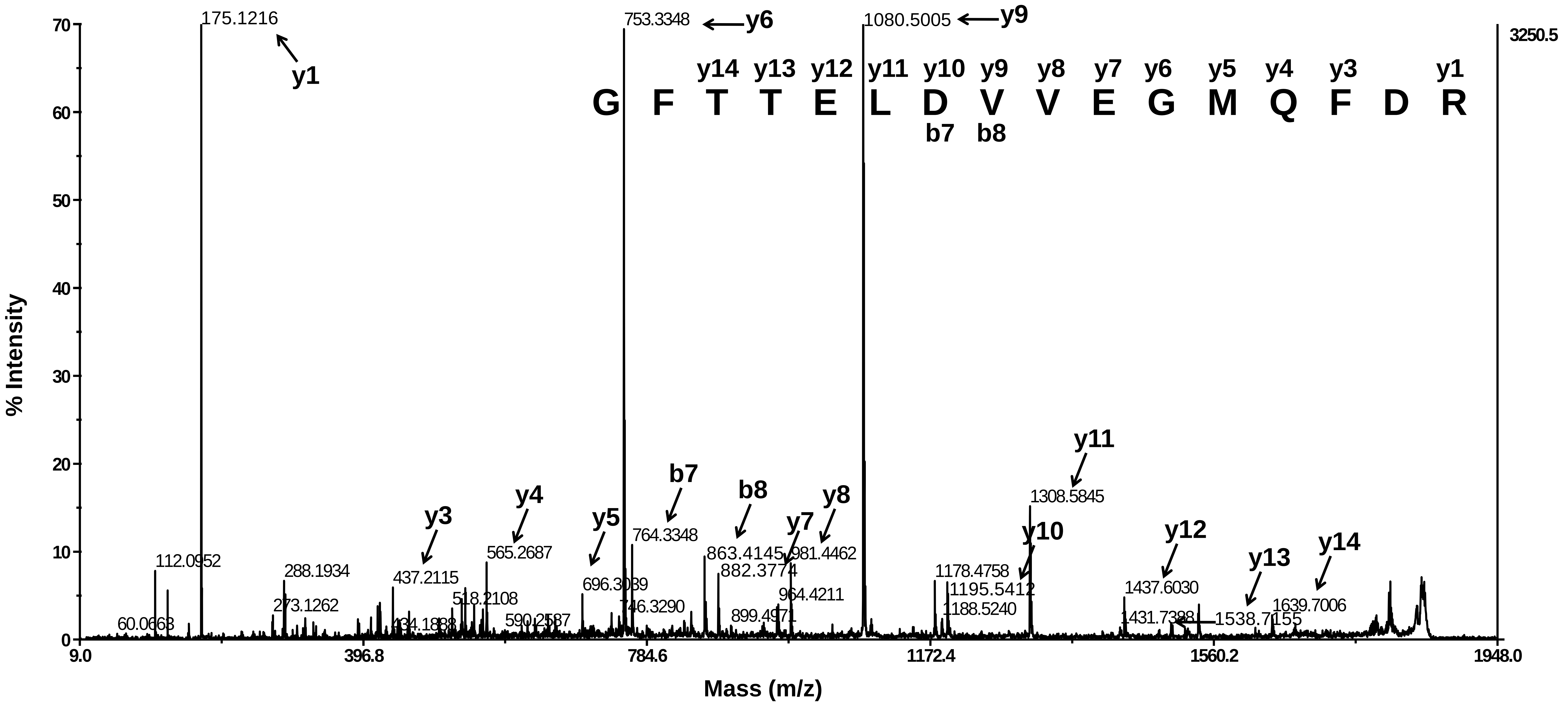

Supplementary Figure S2: Gel image for the proof-of-concept study using blind samples

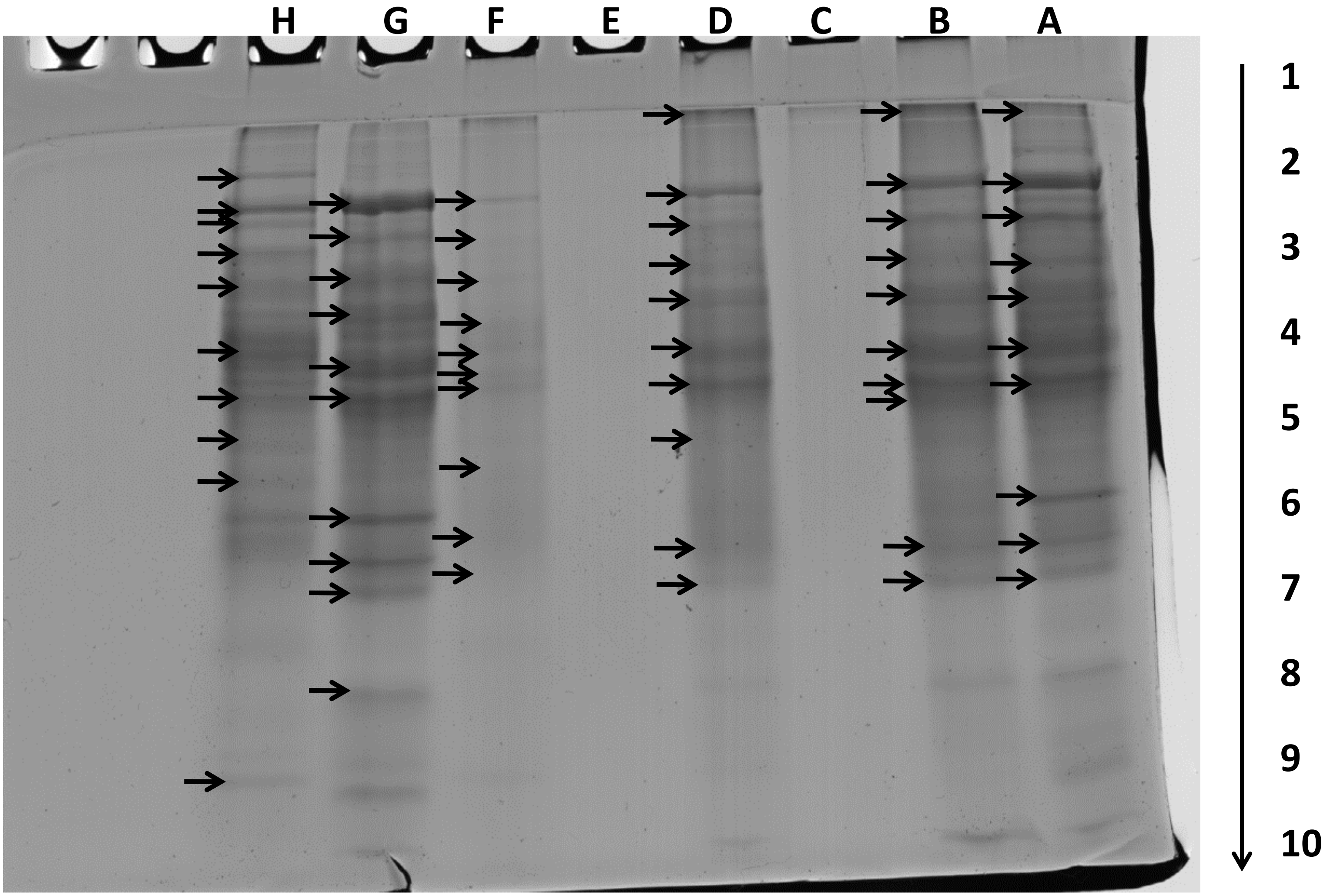

Supplementary figure S2: Proof-of-concept study using blind samples; number coded soil or sand samples spiked with one or two bacterial select agents were subjected to targeted and shotgun analysis using tandem mass spectrometry after extraction of peptides. Ten most abundant proteins (indicated by arrows) from Coomassie-stained SDS-PAGE were excised and subjected to in gel digestion and identification by tandem mass spectrometry as described in the text.

Supplementary Figure S3: MS and MS/MS spectra of tryptic peptides obtained from representative proteins identified in proof-of-concept study.

MS and MS/MS spectra of formate acetyl transferase: MASCOT Score 927

Corresponding to results shown in supplementary table S5

Formate acetyl transferase: MS Spectrum

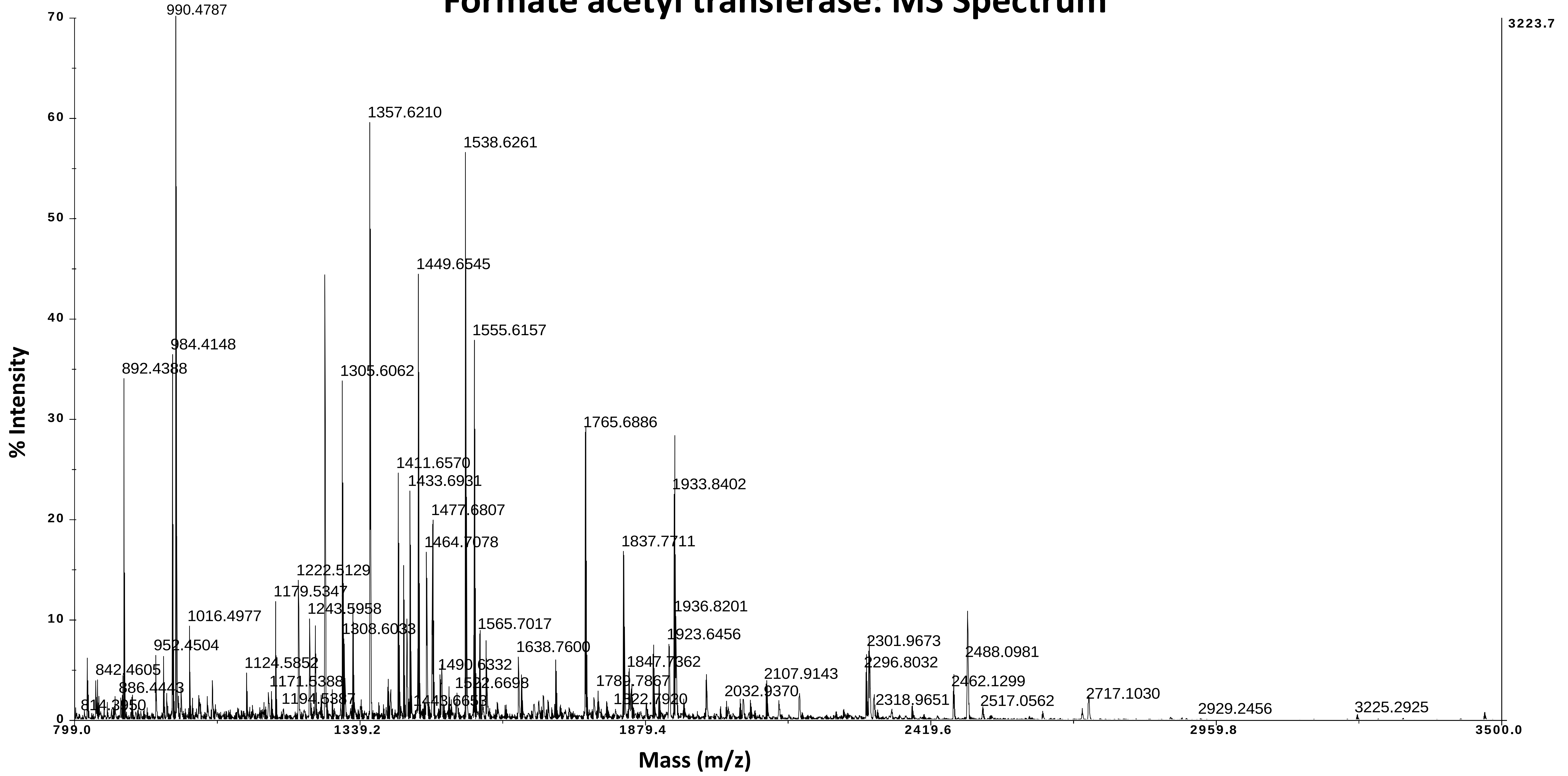

Formate acetyl transferase: MS/MS Precursor – 1222.512

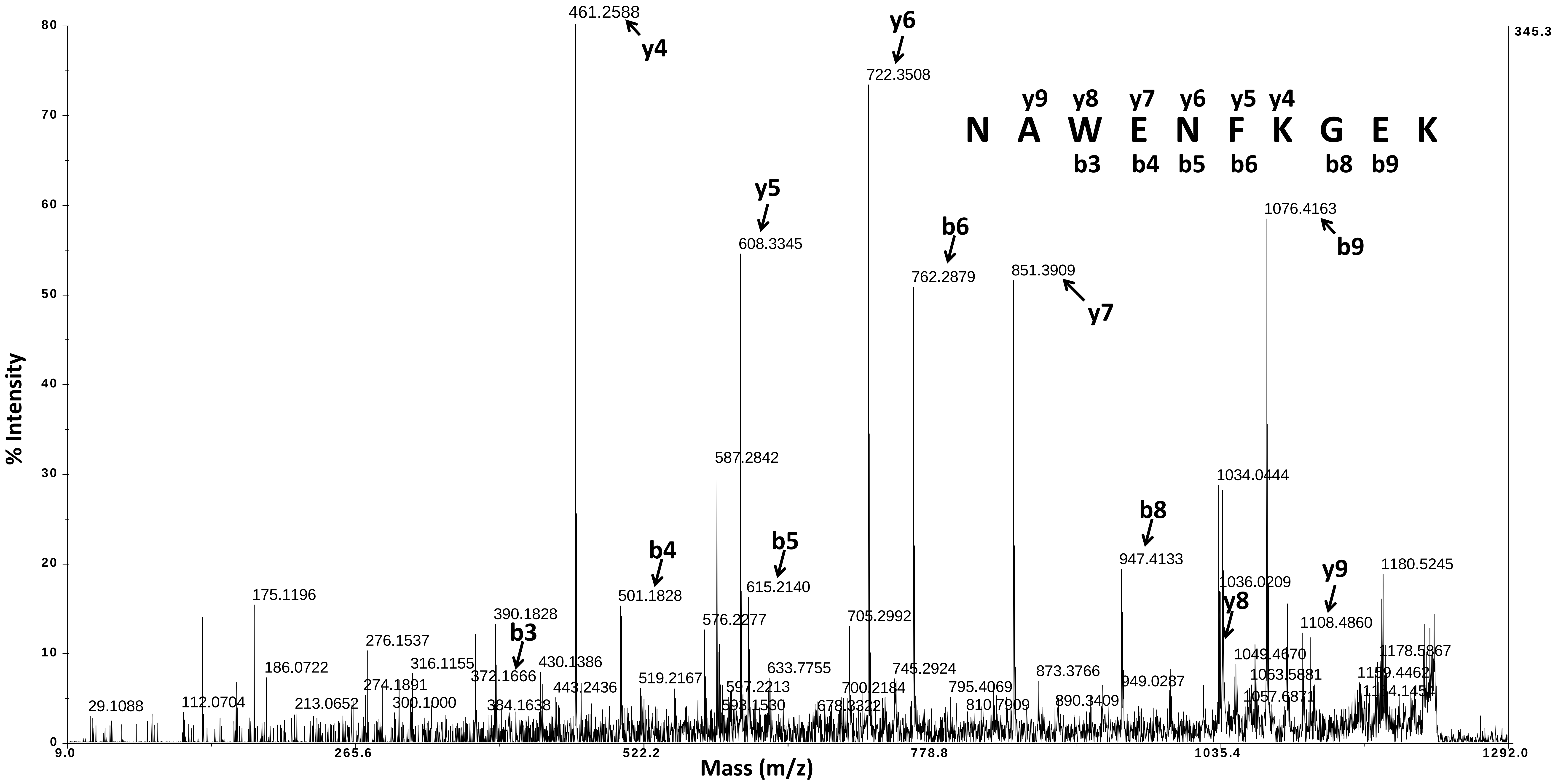

Formate acetyl transferase: MS/MS Precursor – 1433.69

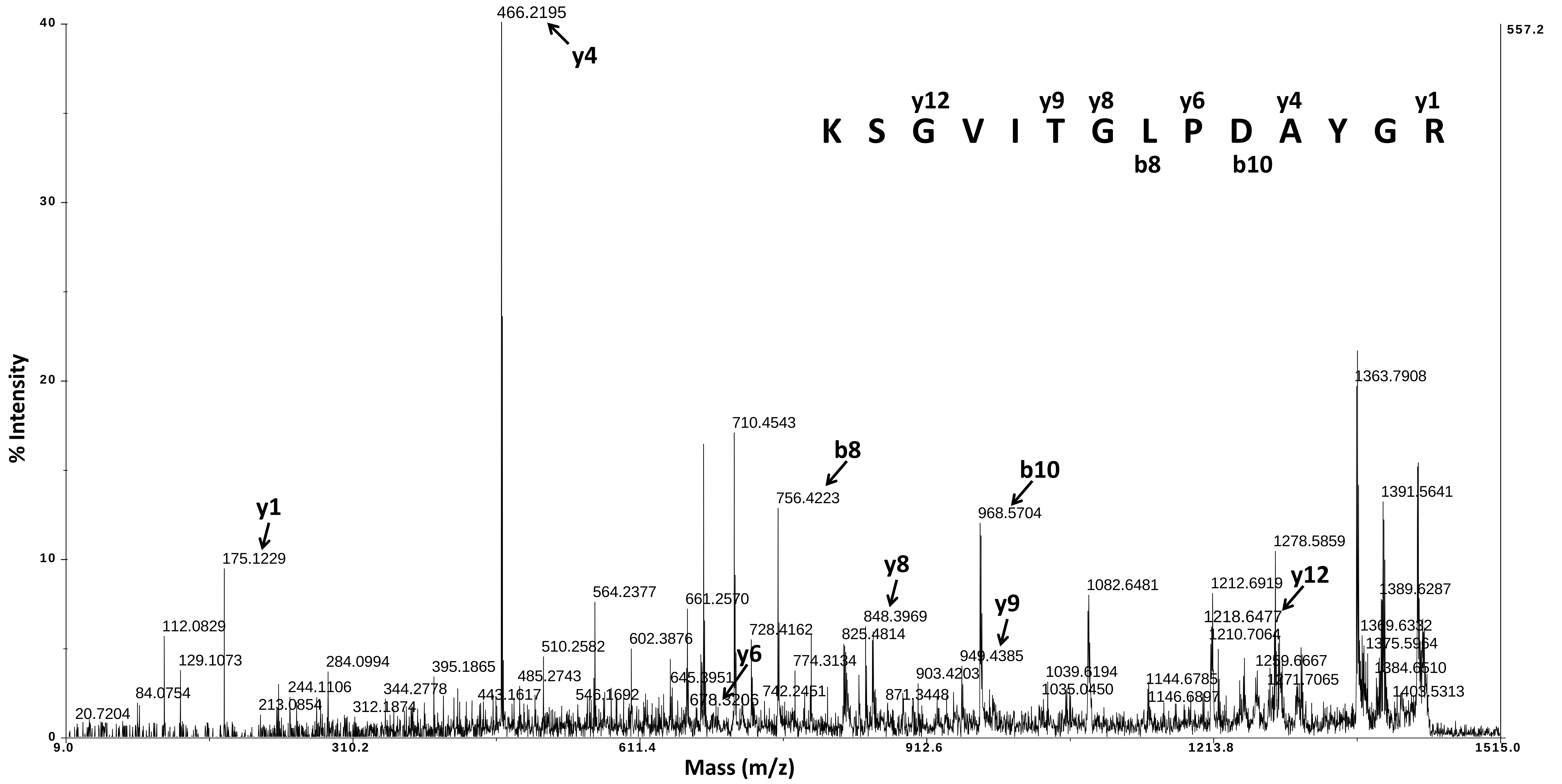

Formate acetyl transferase: MS/MS Precursor – 1411.65

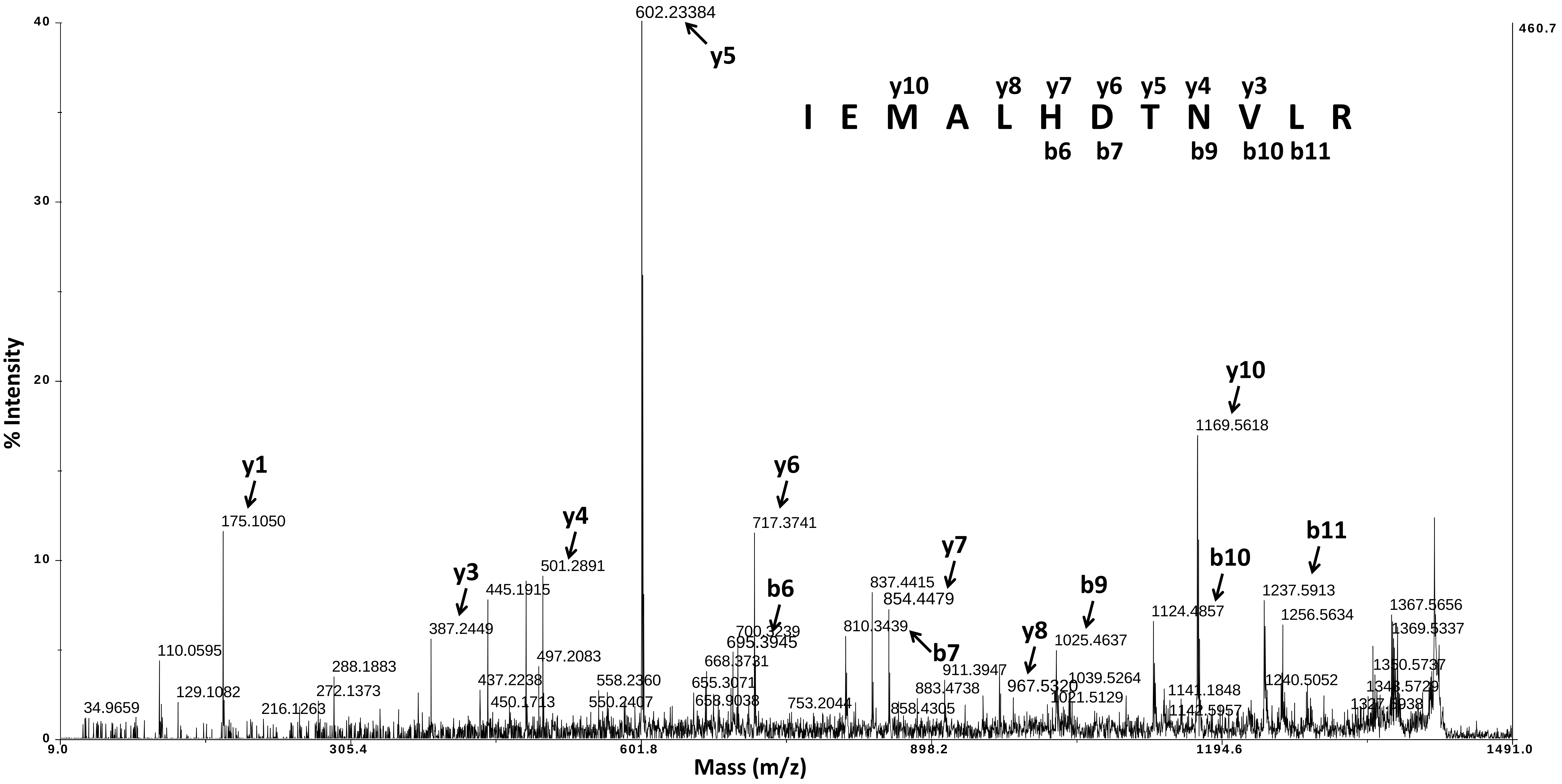

Formate acetyl transferase: MS/MS Precursor – 984.415

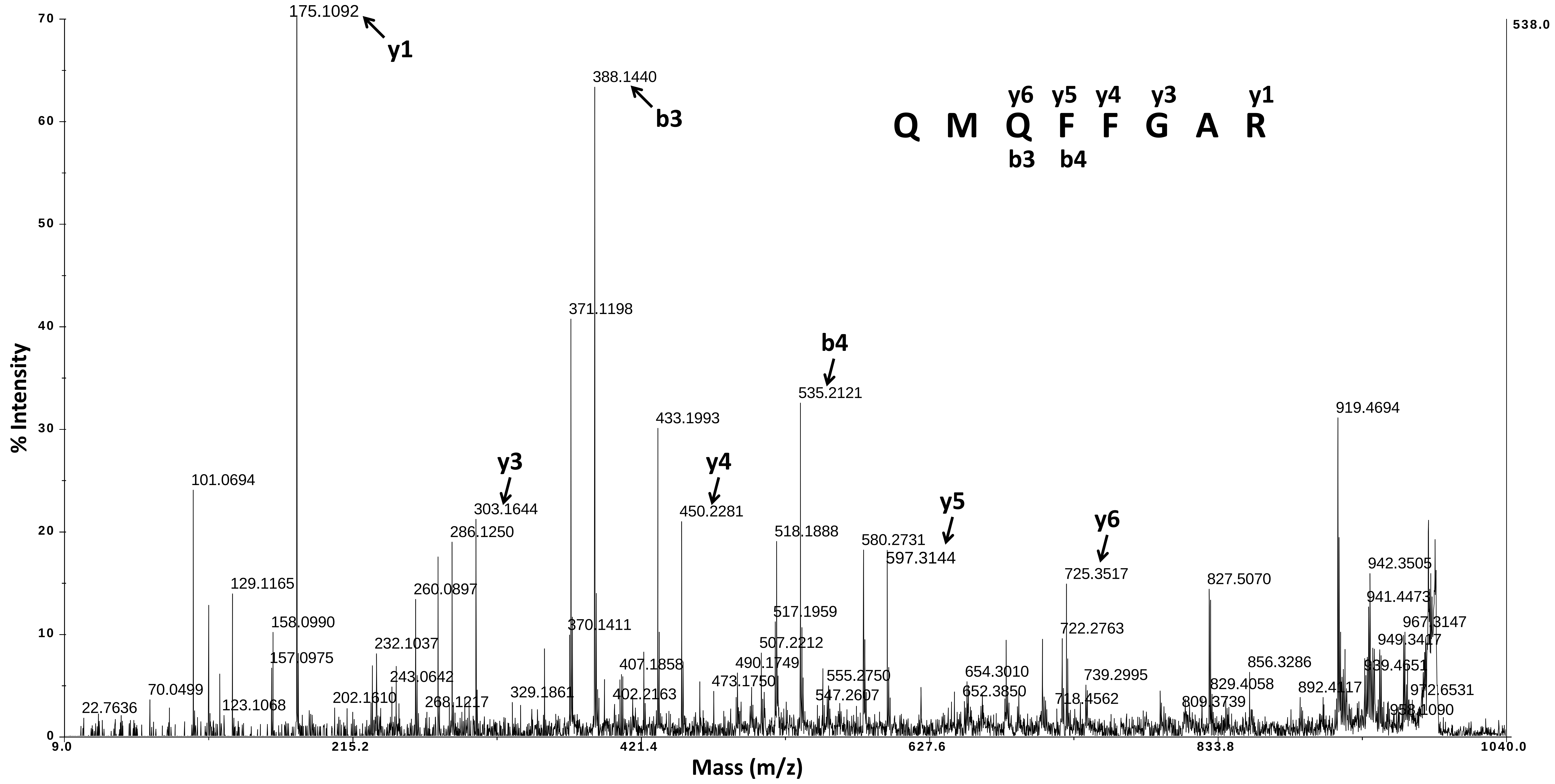

Formate acetyl transferase: MS/MS Precursor – 1305.61

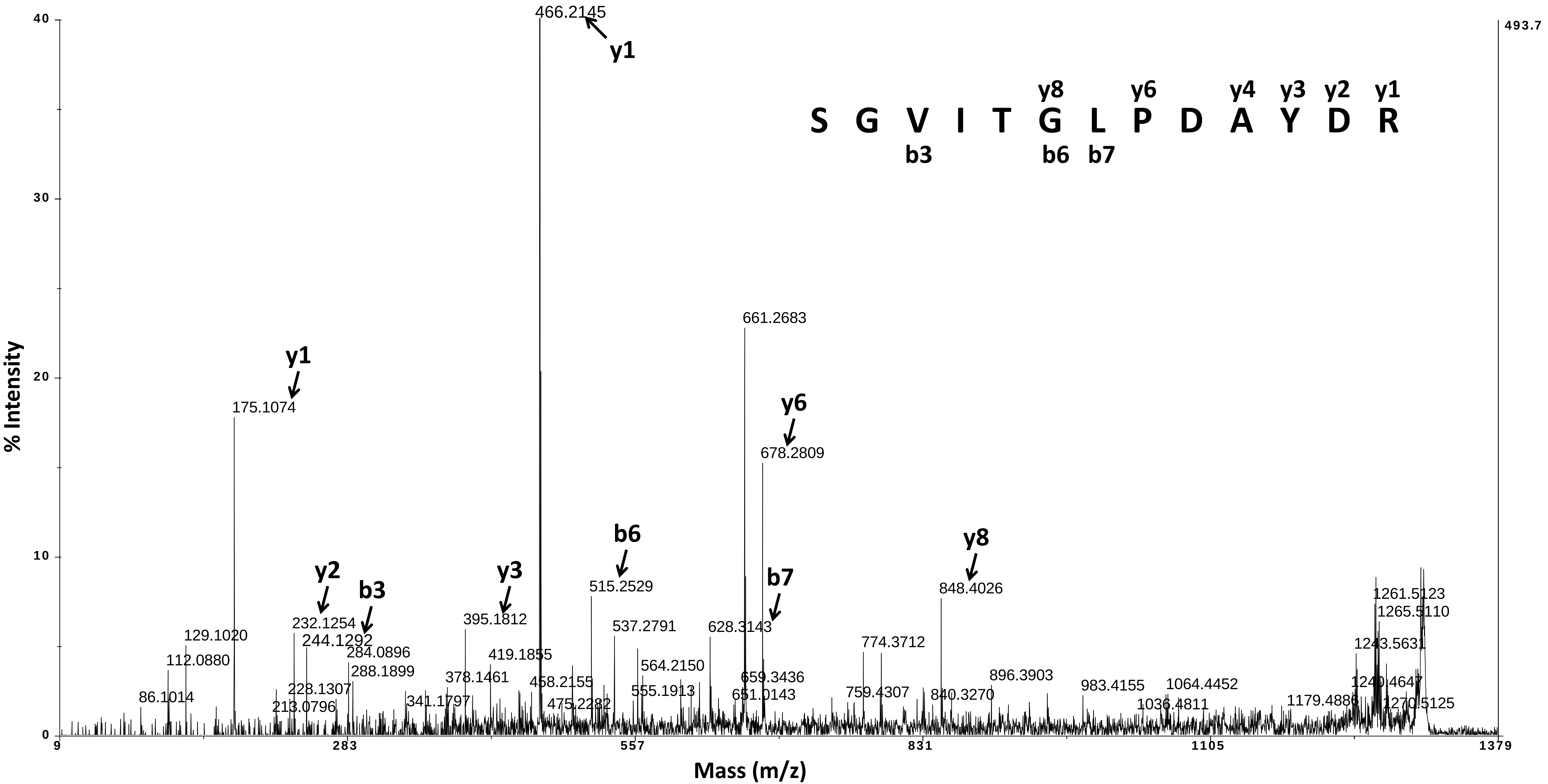

Formate acetyl transferase: MS/MS Precursor – 1555.61

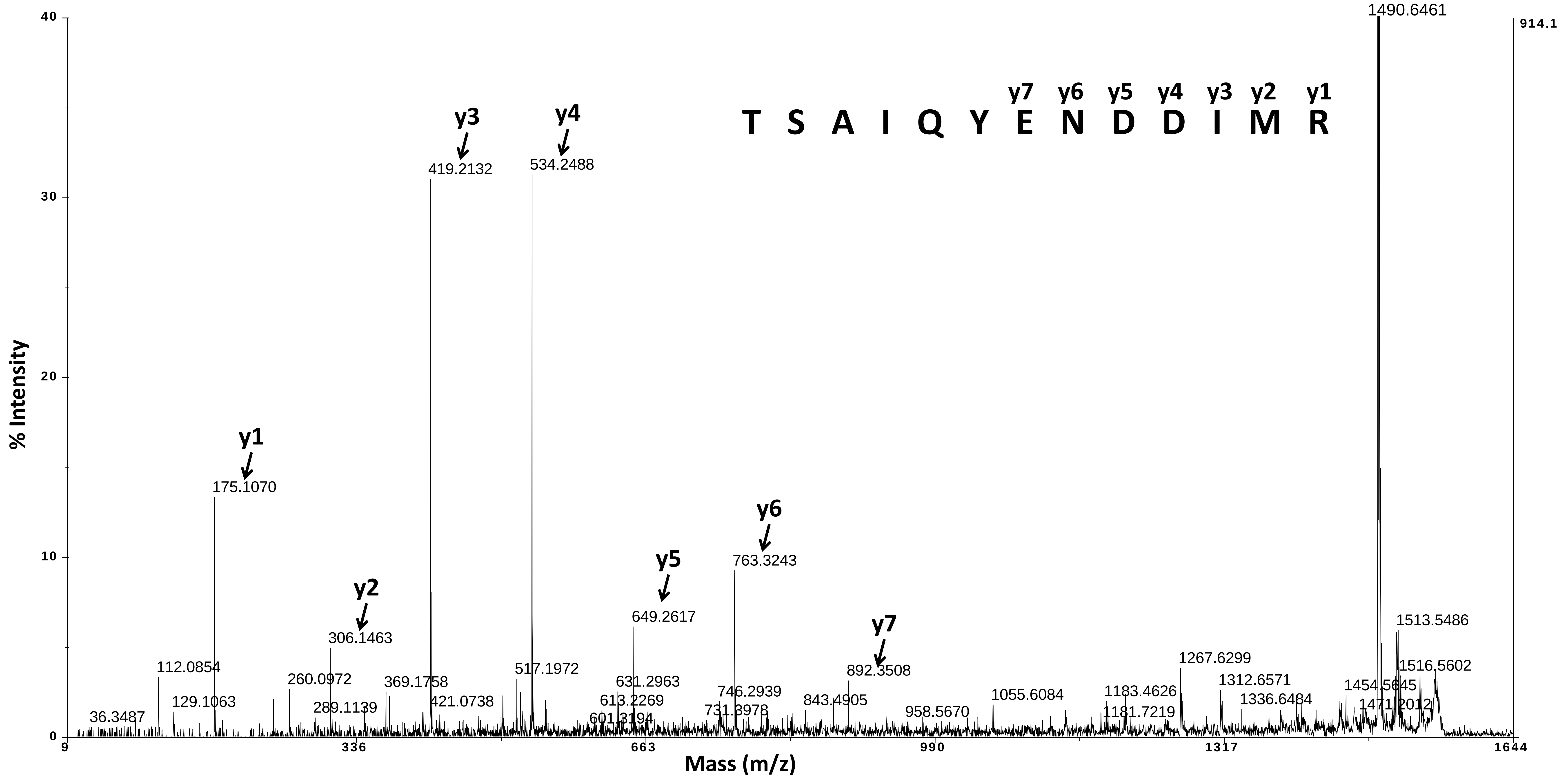

Formate acetyl transferase: MS/MS Precursor – 1449.65

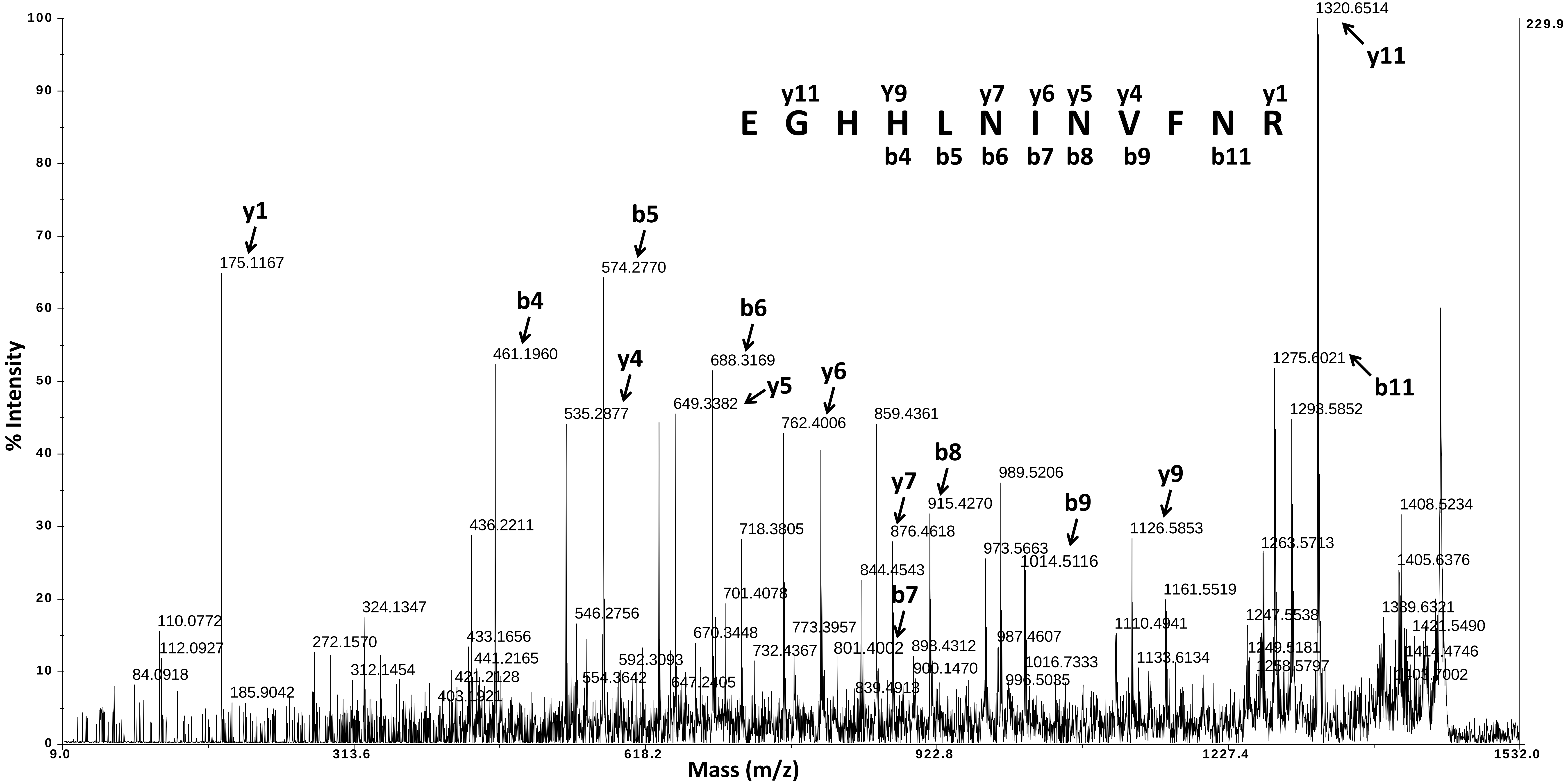

Formate acetyl transferase: MS/MS Precursor – 1765.69

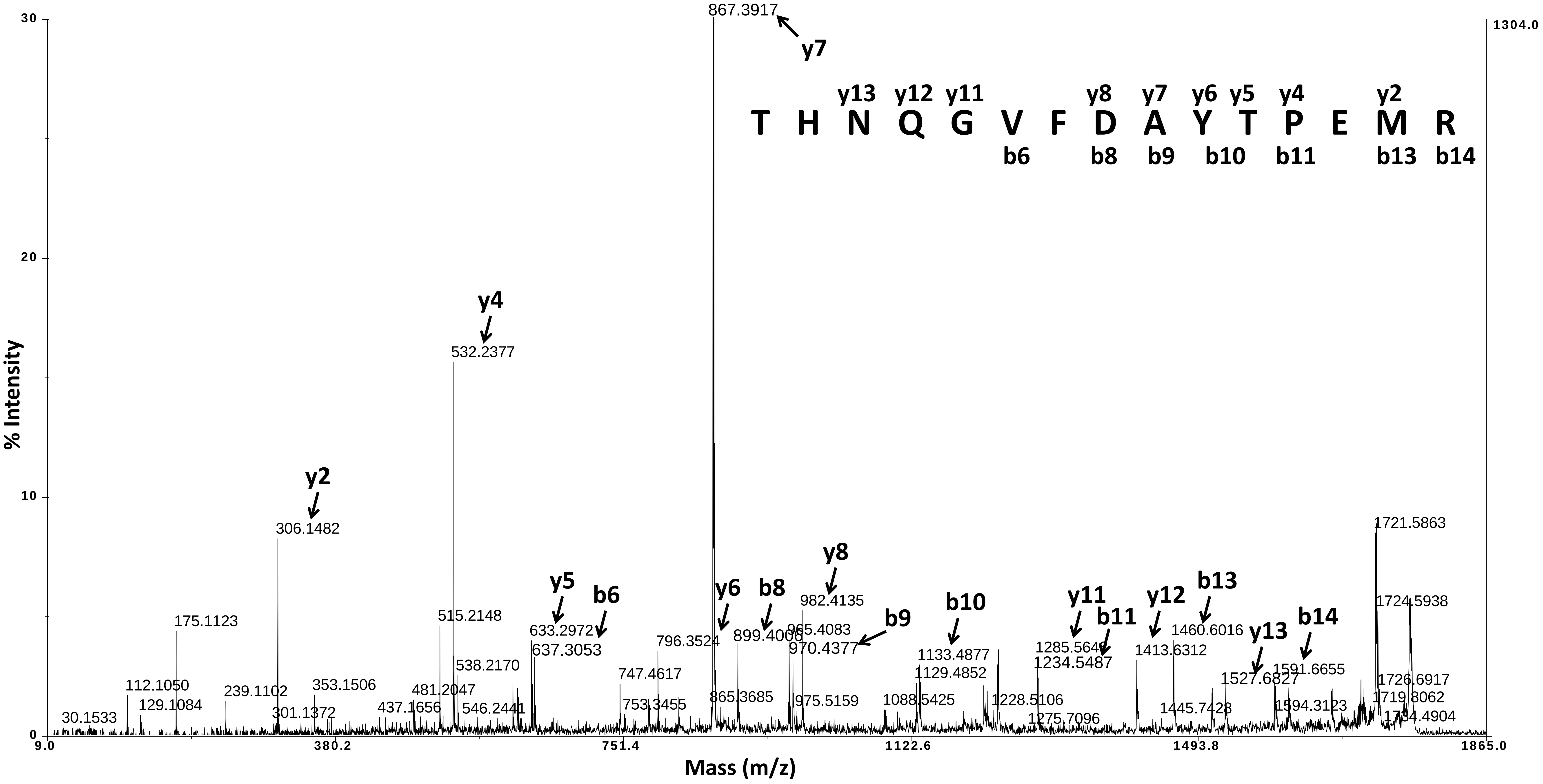

Formate acetyl transferase: MS/MS Precursor – 1933.84

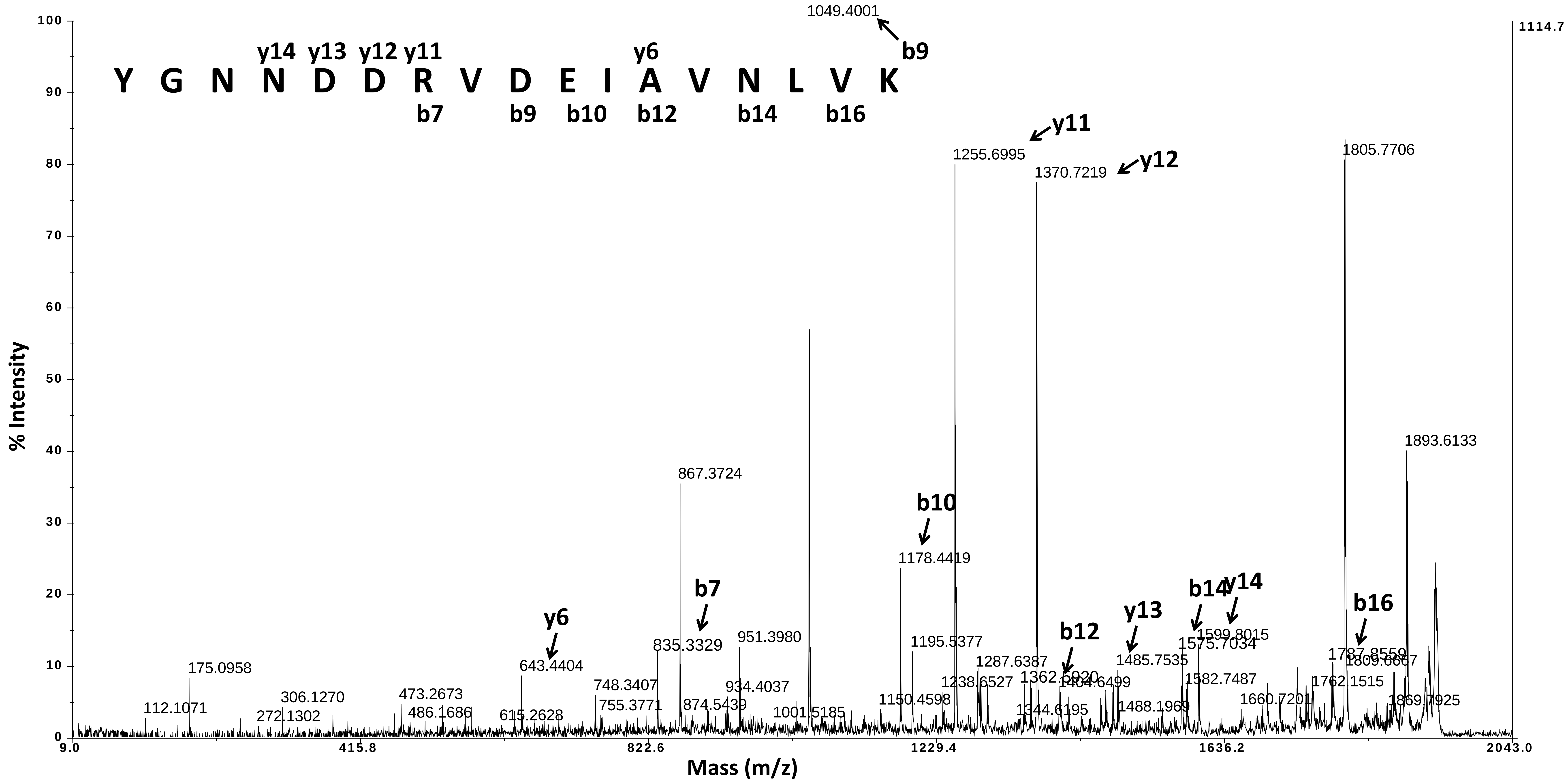

Formate acetyl transferase: MS/MS Precursor – 1357.62

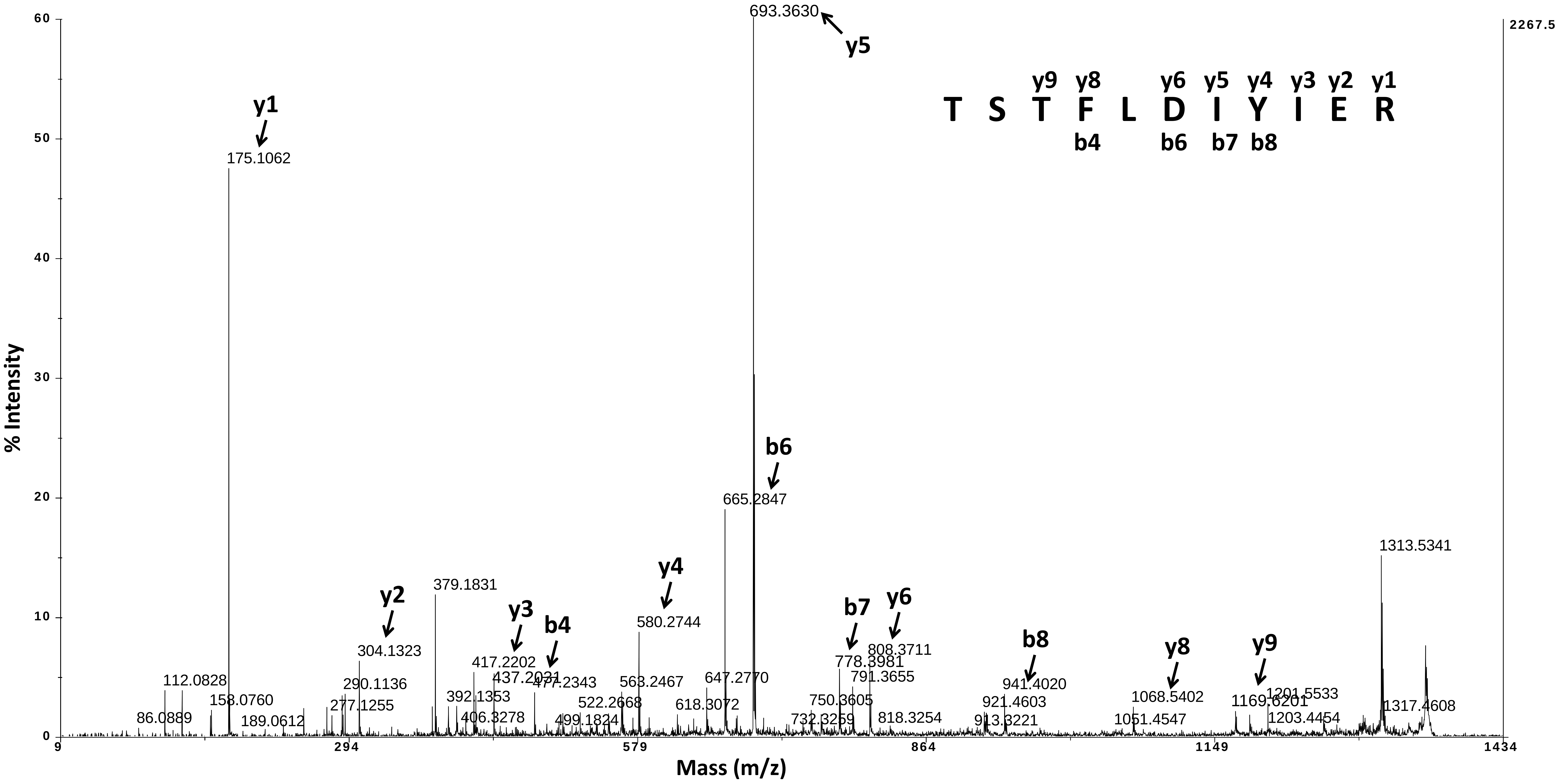

Formate acetyl transferase: MS/MS Precursor – 1538.63

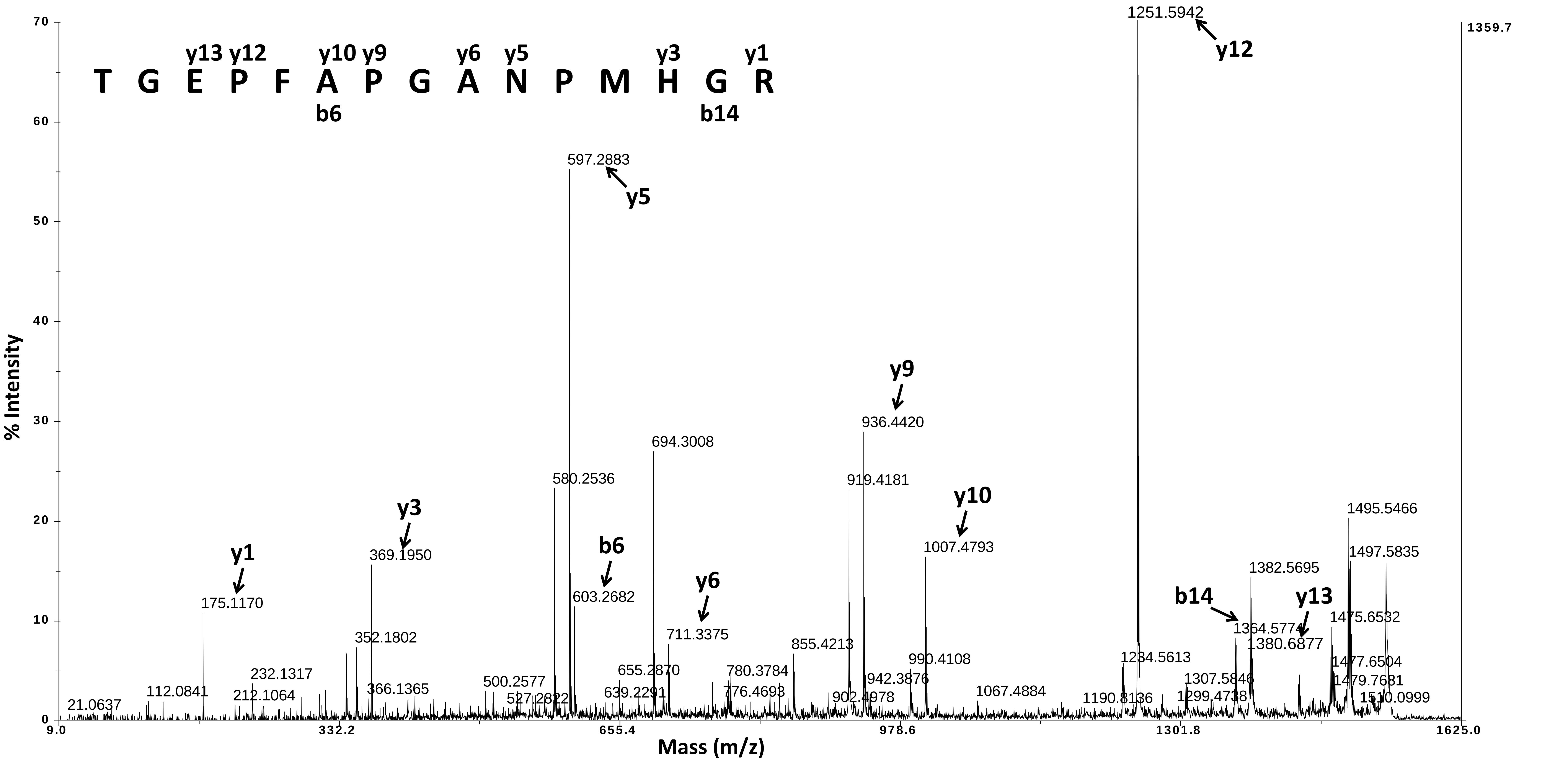

Formate acetyl transferase: MS/MS Precursor – 990.478

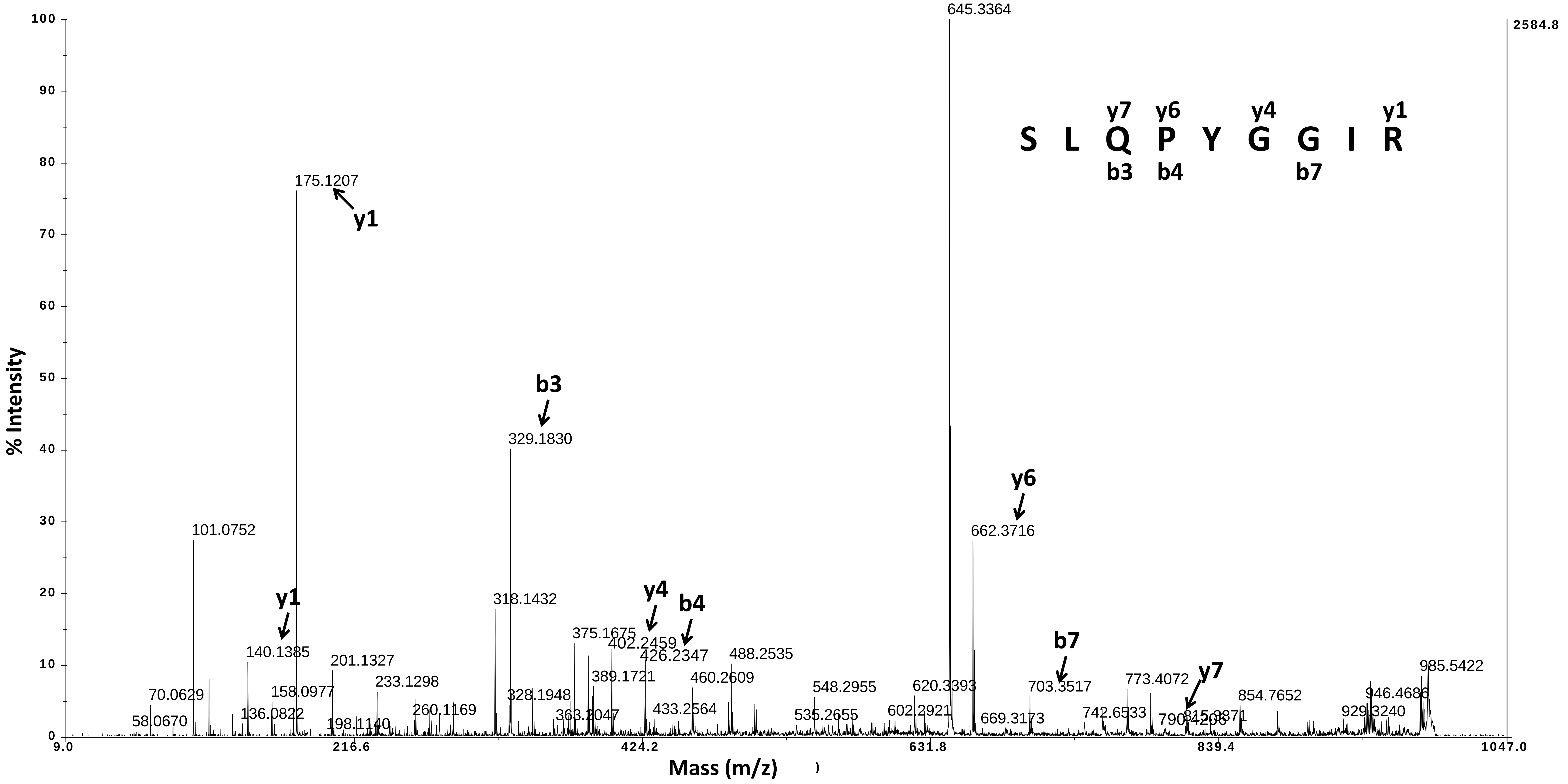

Formate acetyl transferase: MS/MS Precursor – 2488.1

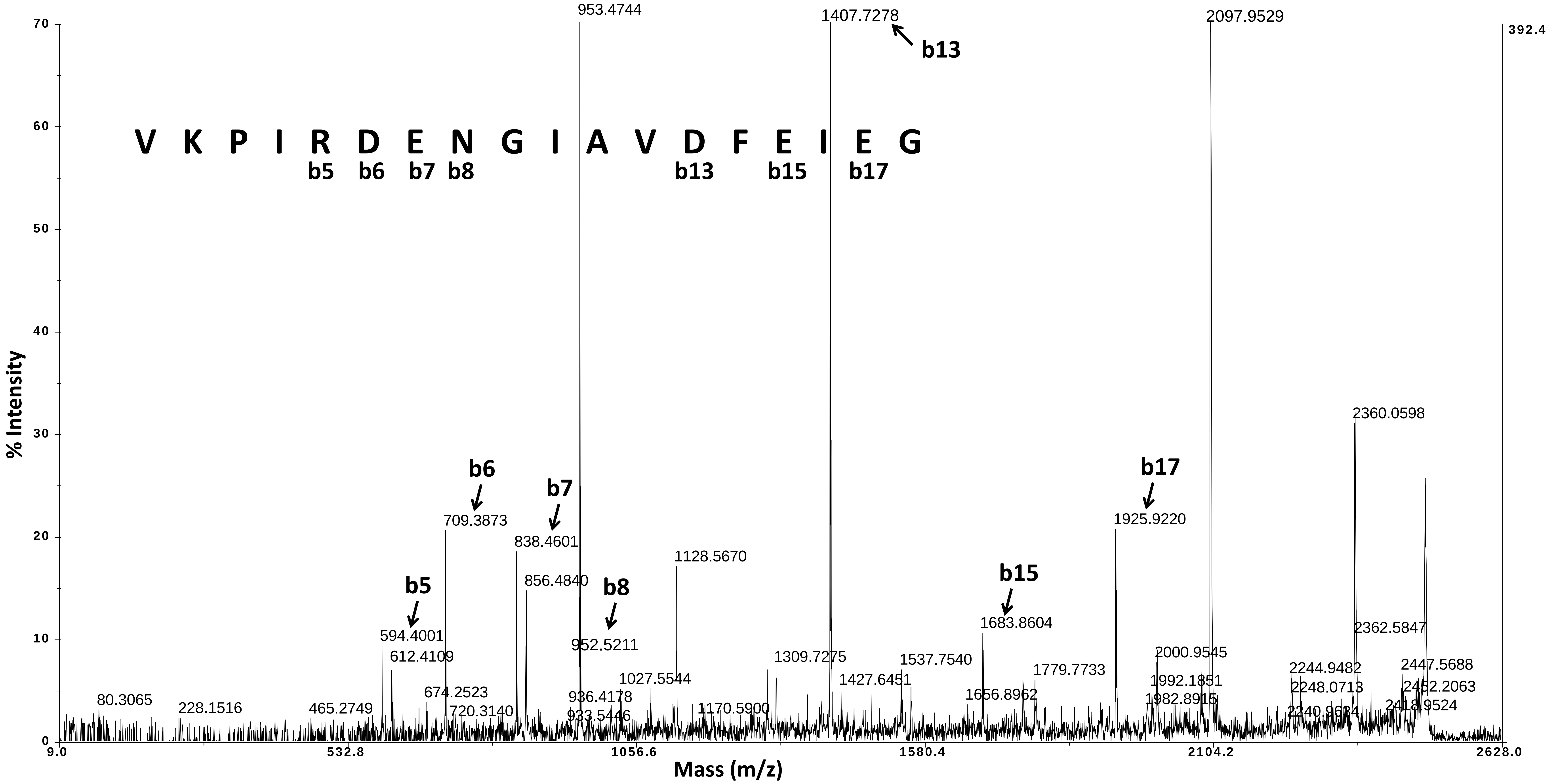

MS and MS/MS spectra of Chaperonin GroEL: MASCOT Score 521

Corresponding to verification results shown in supplementary table S4

Chaperonin GroEL: MS Spectrum

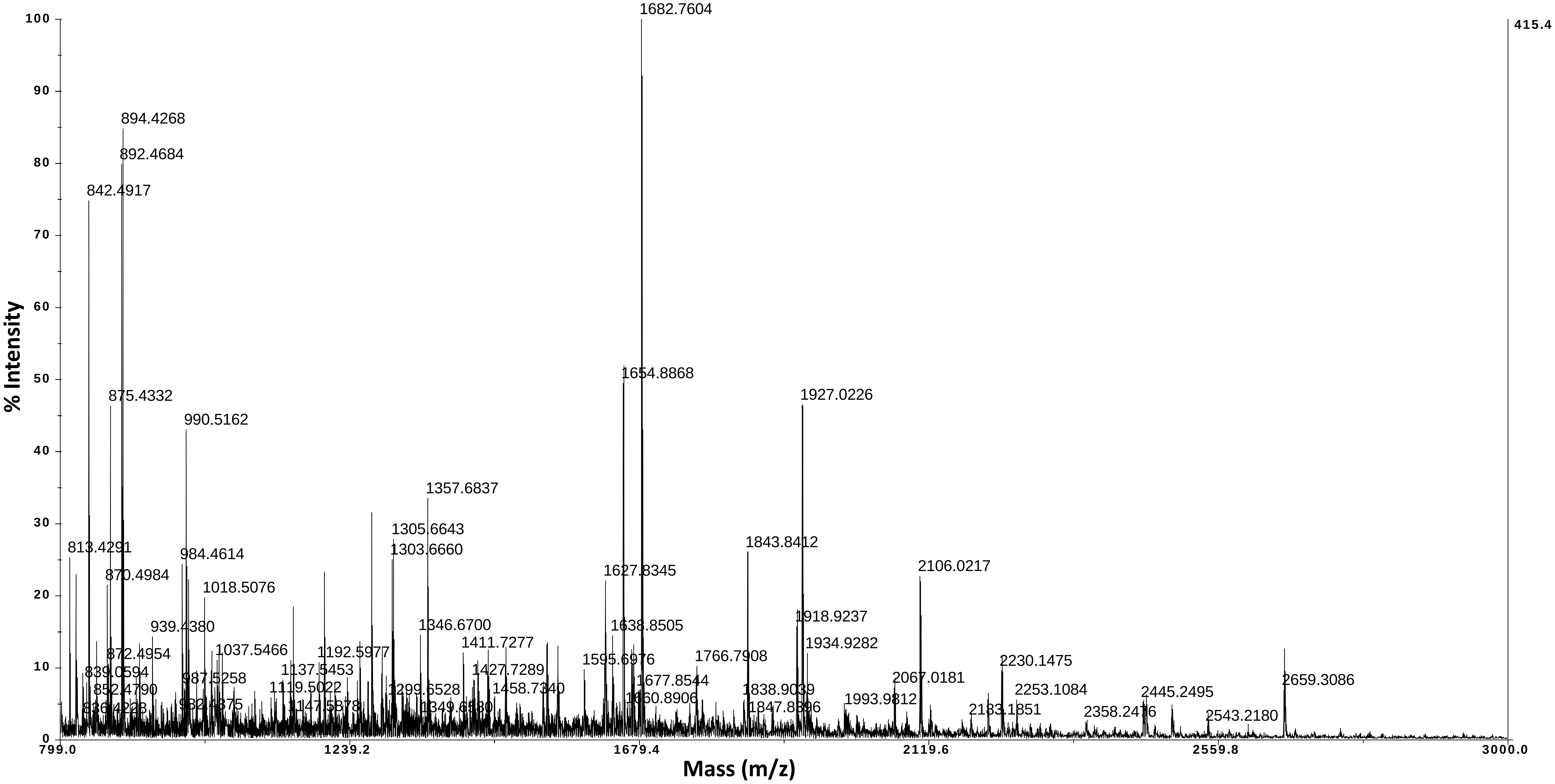

Chaperonin GroEL: MS/MS Precursor – 813.429

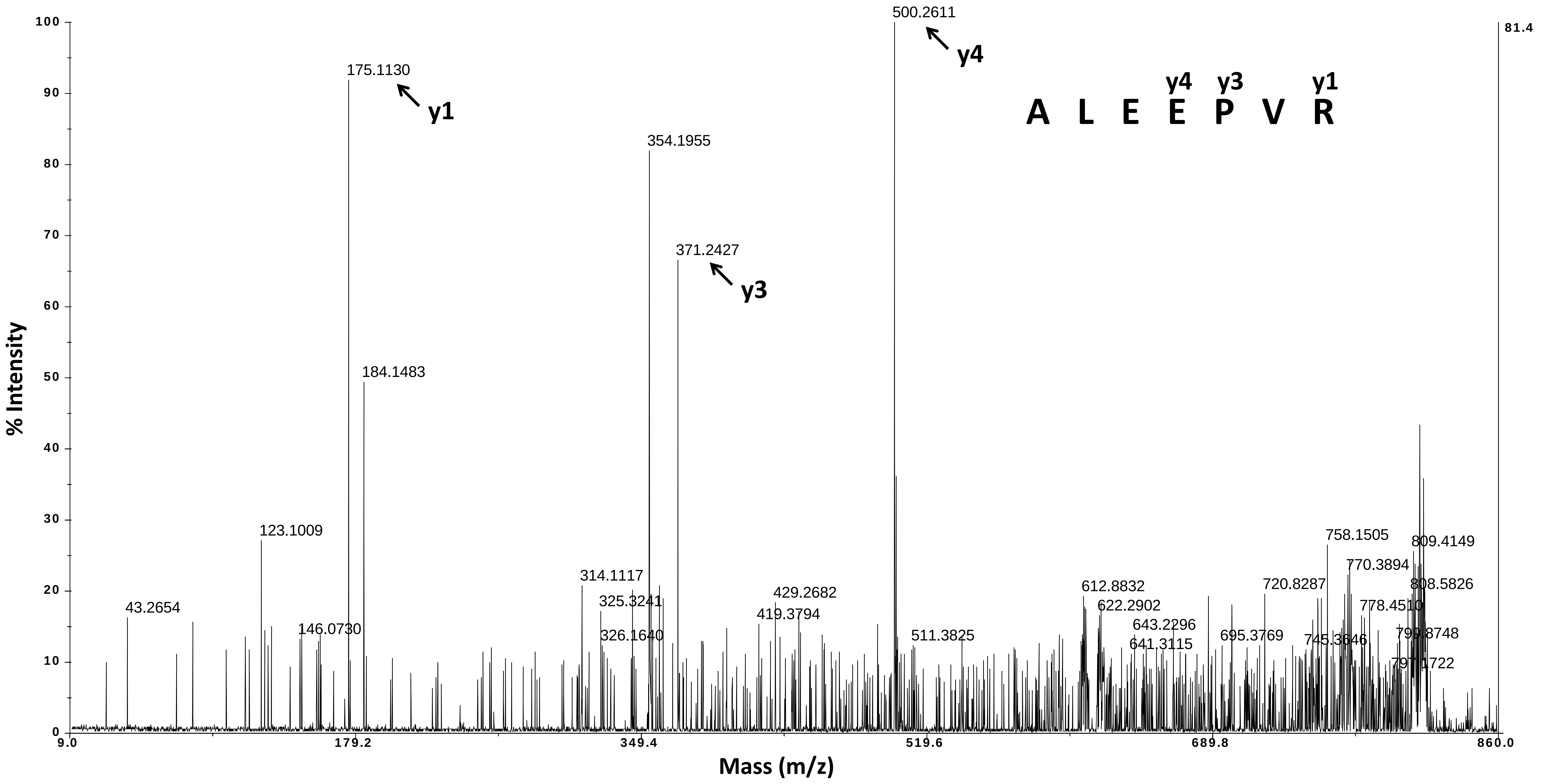

Chaperonin GroEL: MS/MS Precursor – 1654.89

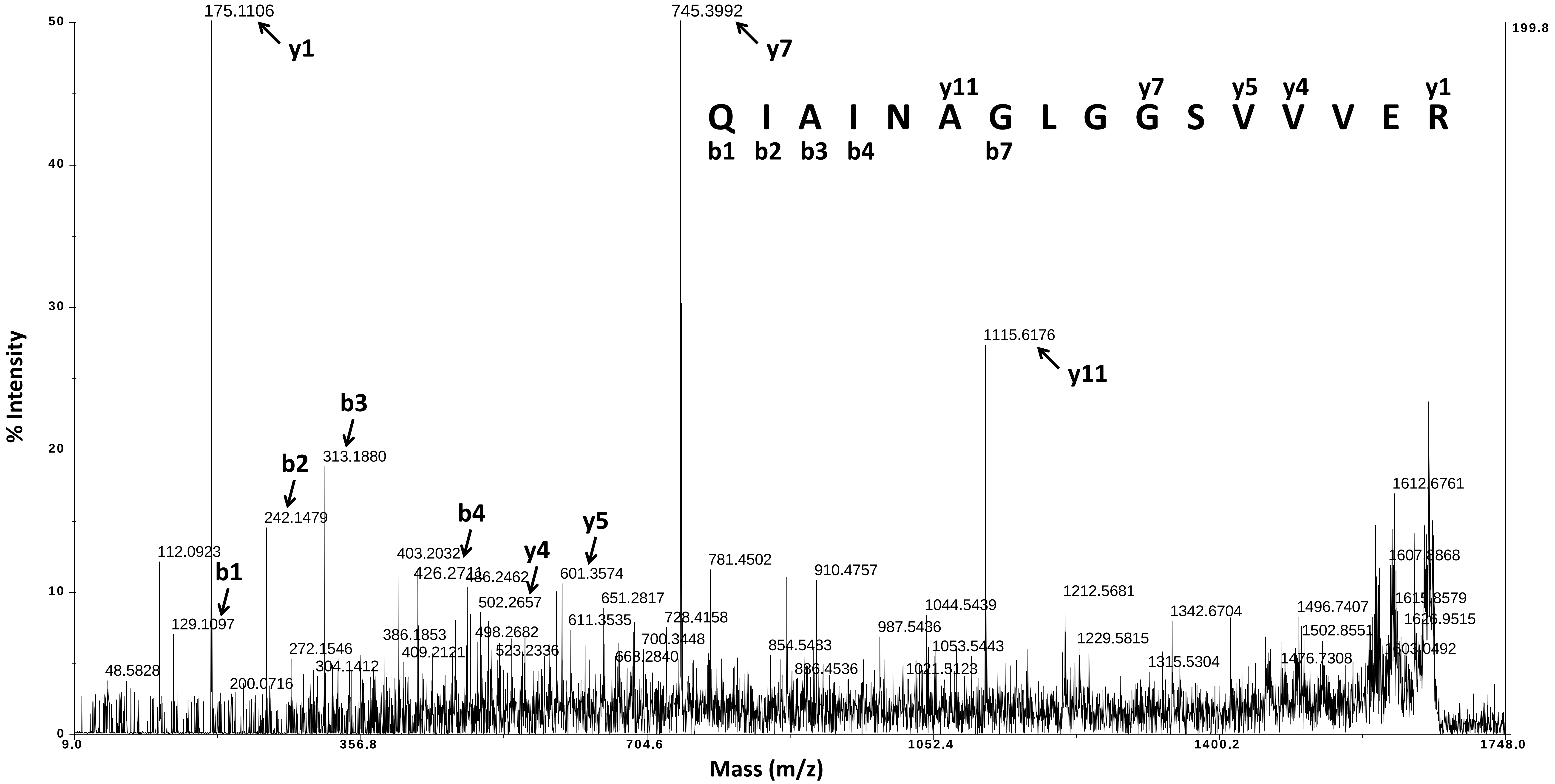

Chaperonin GroEL: MS/MS Precursor – 1927.02

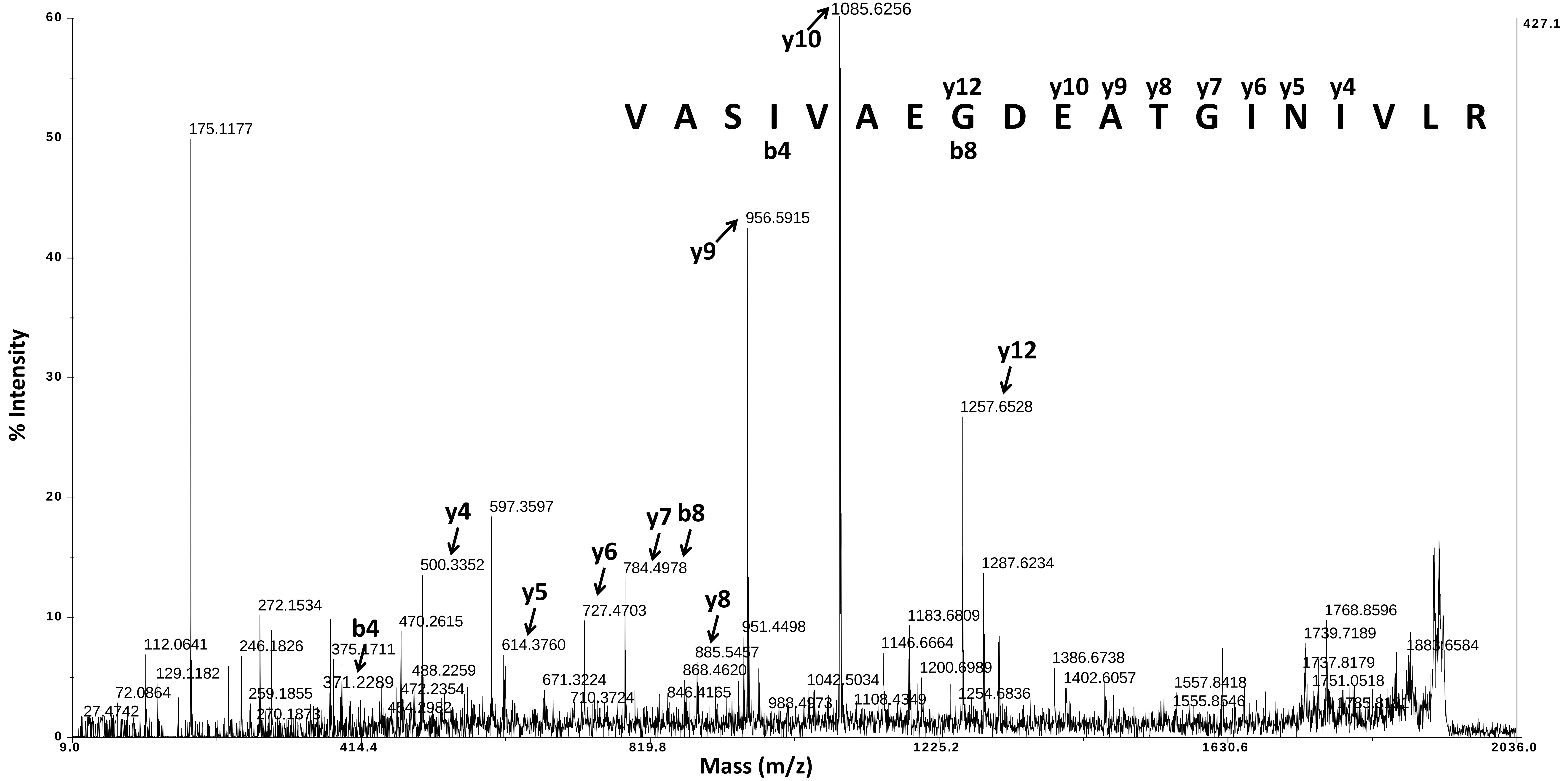

Chaperonin GroEL: MS/MS Precursor – 1682.76

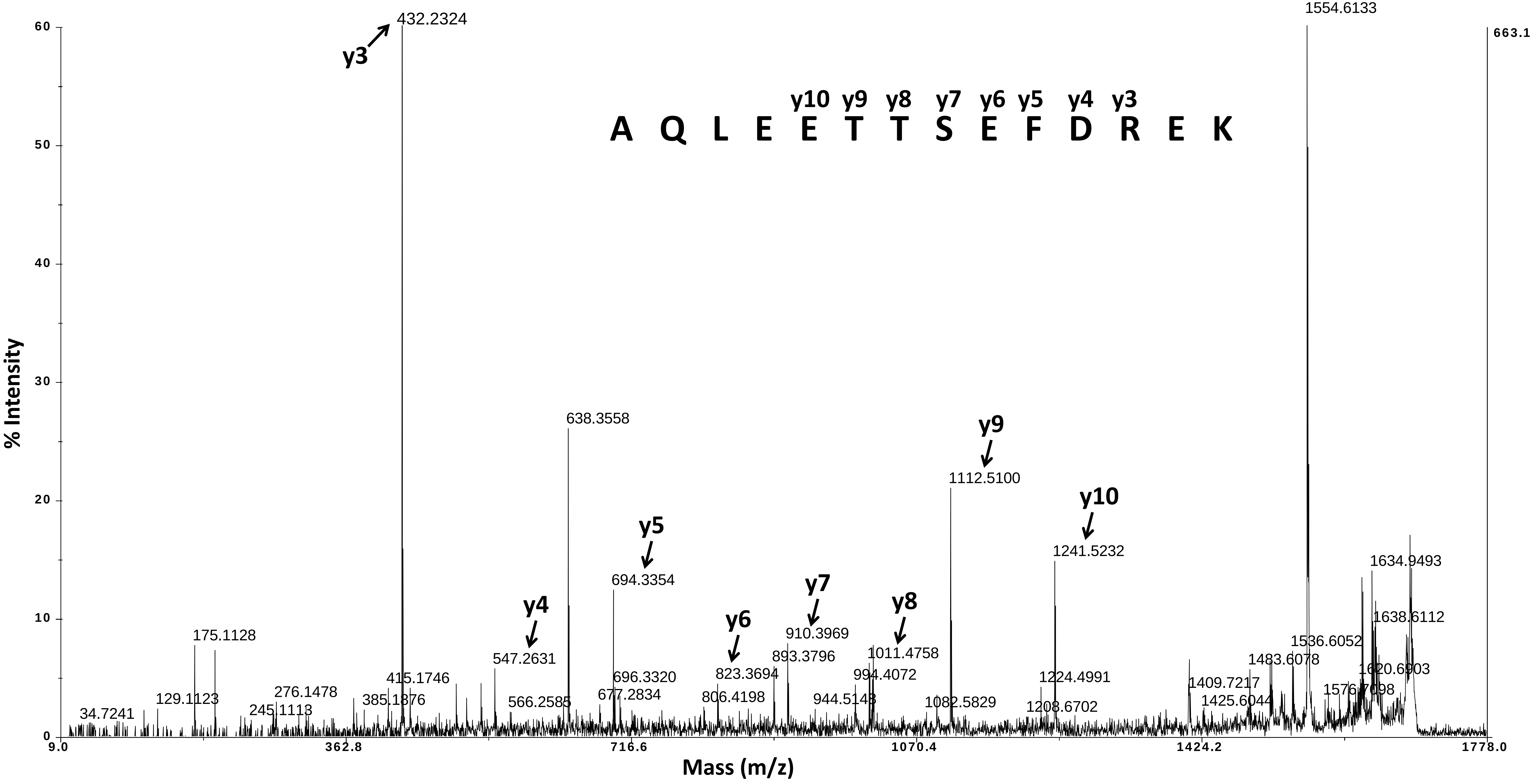

MS and MS/MS spectra of S-layer protein: MASCOT Score 433

Corresponding to verification results shown in supplementary table S4

S – Layer Protein EA1: MS Spectrum

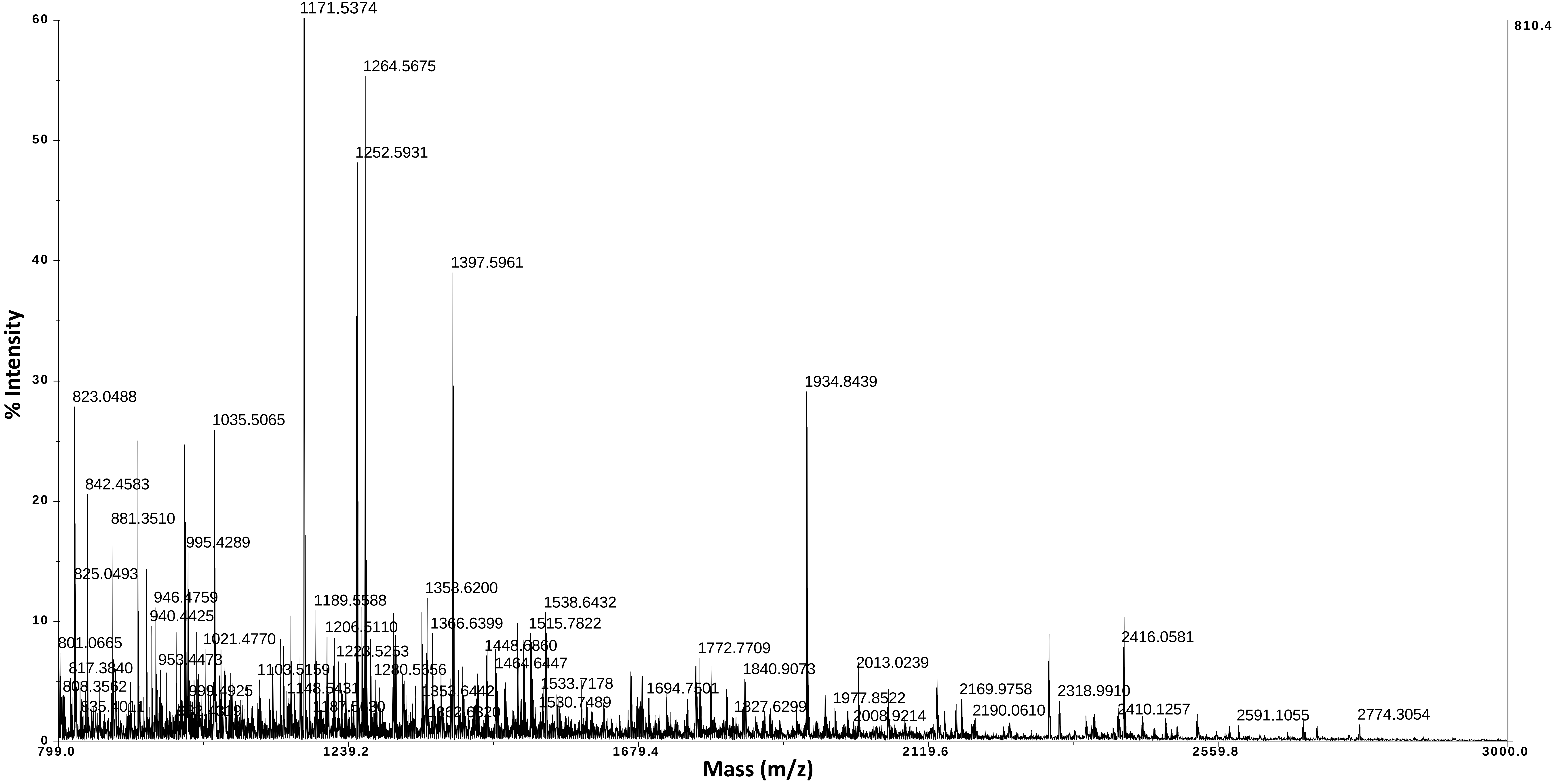

S – Layer Protein EA1: MS/MS Precursor – 2527.13

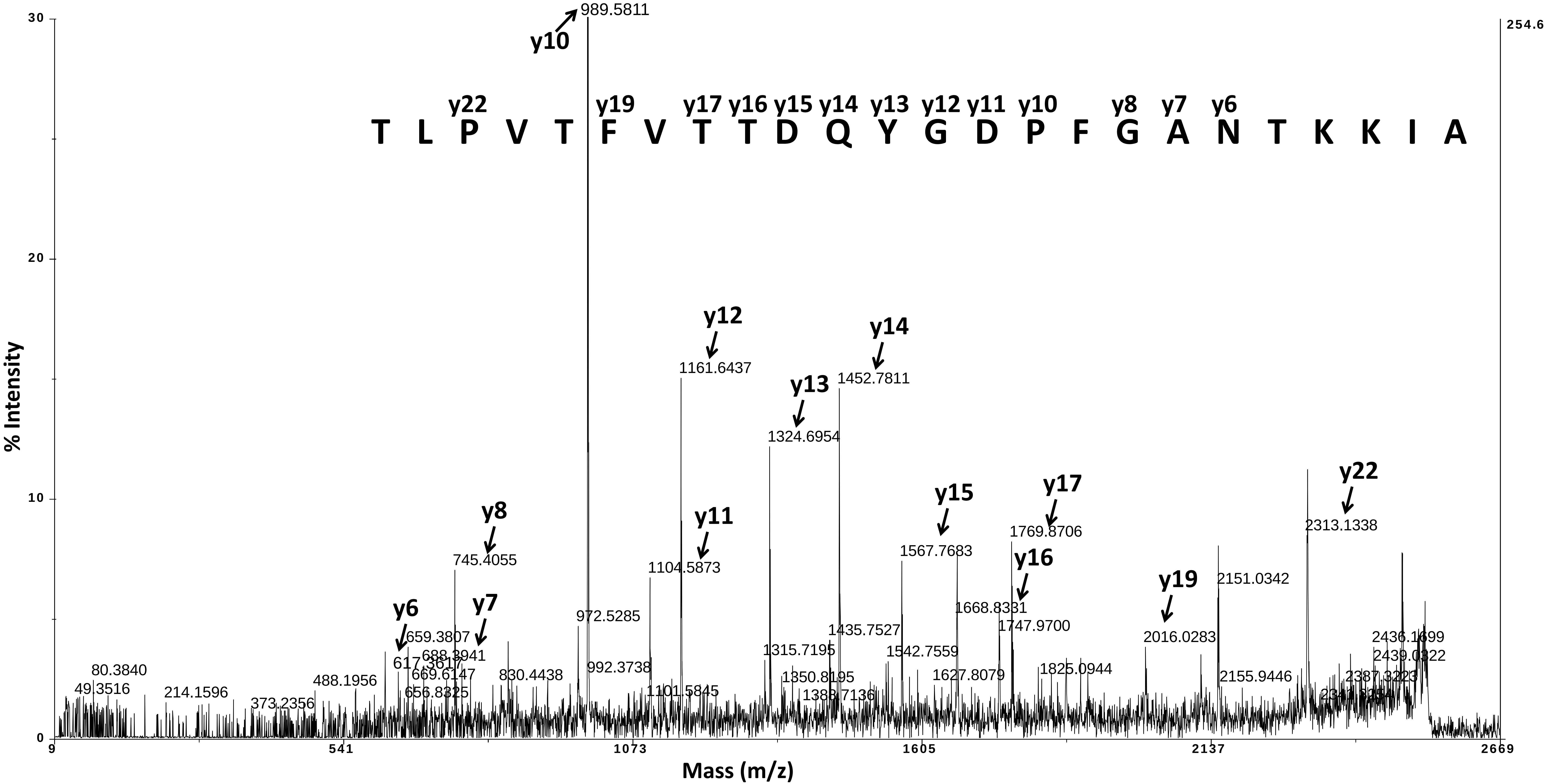

S – Layer Protein EA1: MS/MS Precursor – 1934.84

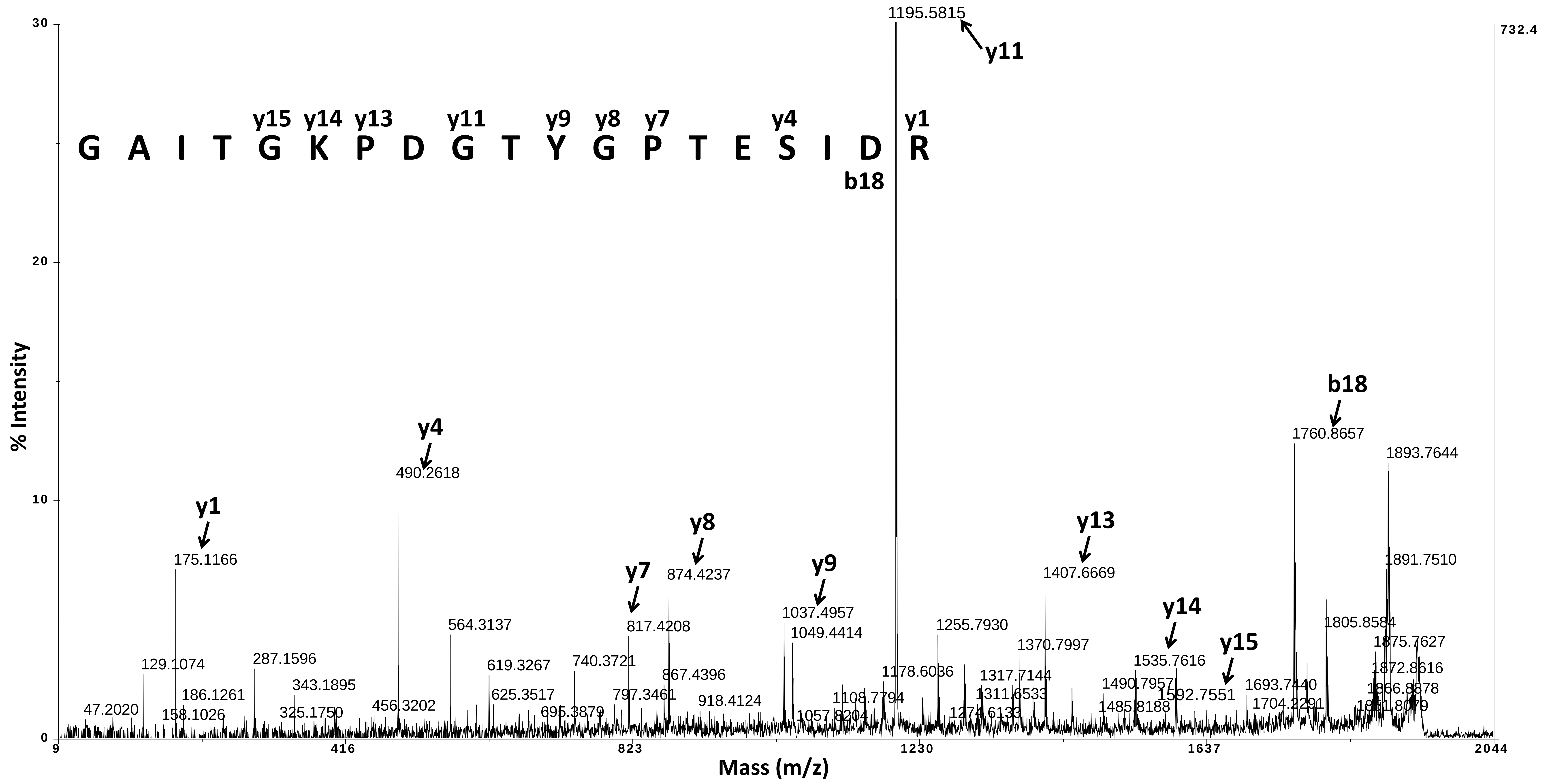

S – Layer Protein EA1: MS/MS Precursor – 1140.52

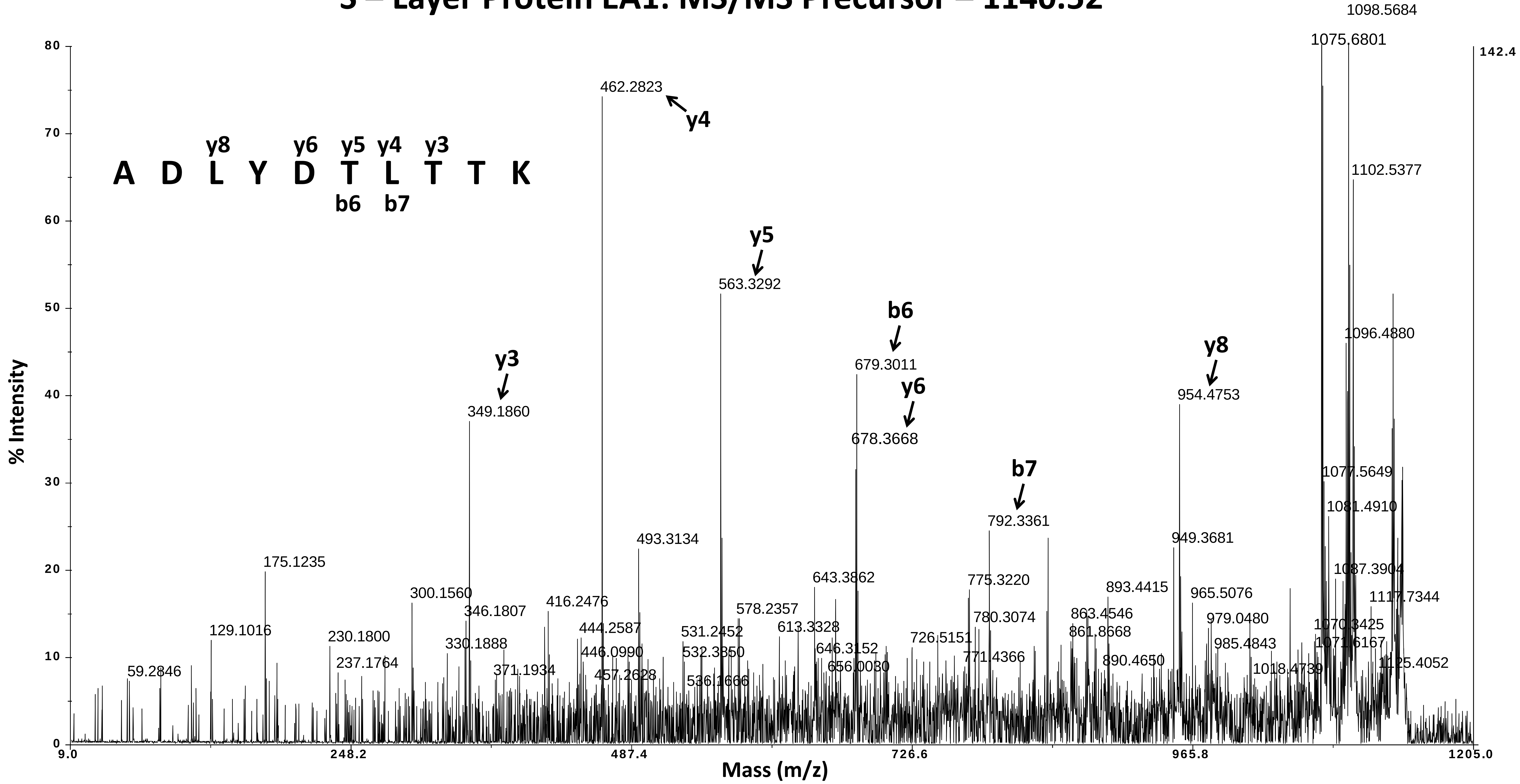

MS and MS/MS spectra of EF-Tu: MASCOT Score 179

Corresponding to verification results shown in supplementary table S4

Elongation Factor Tu: MS Spectrum

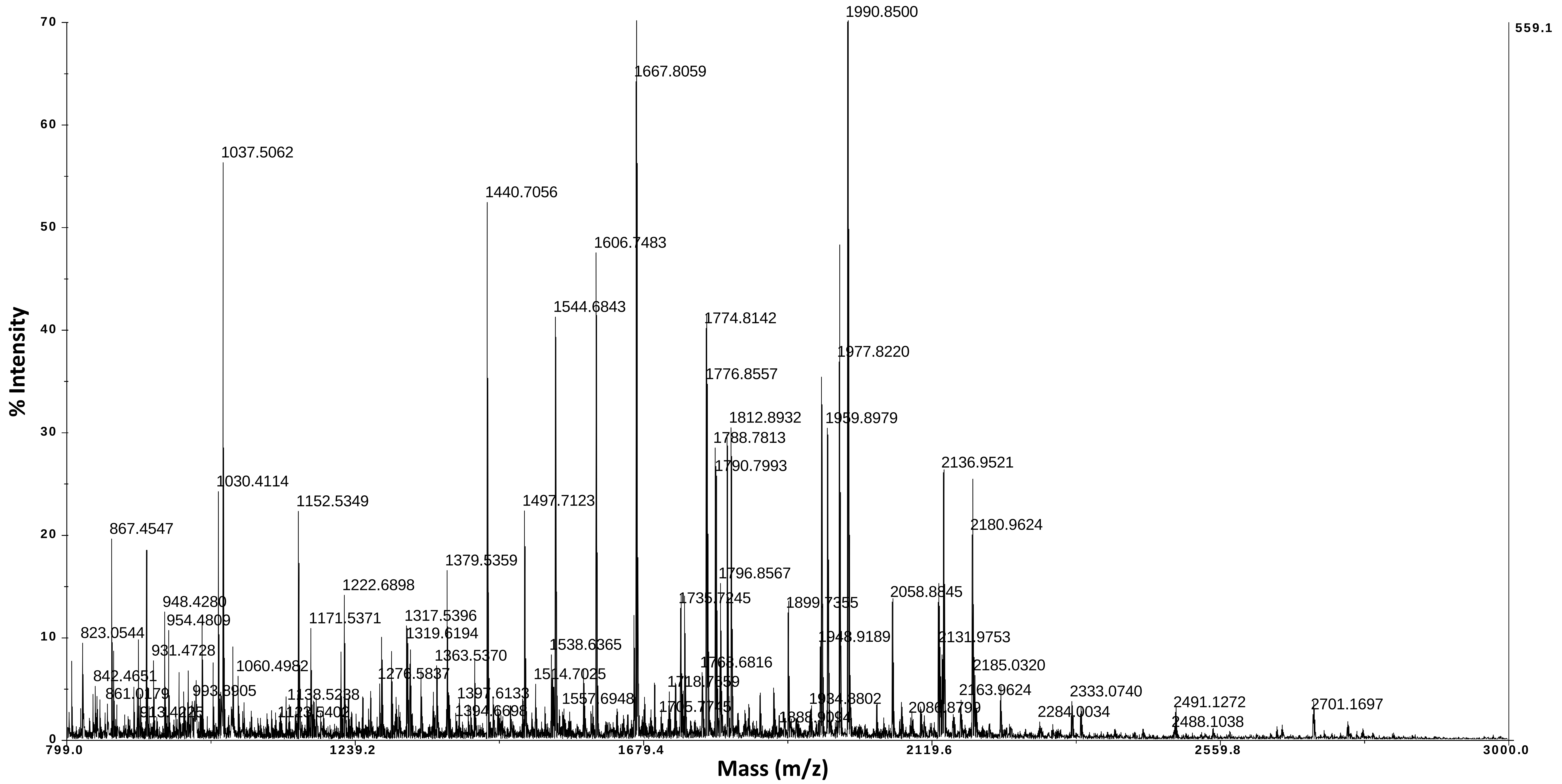

Elongation Factor Tu: MS/MS Precursor – 1768.68

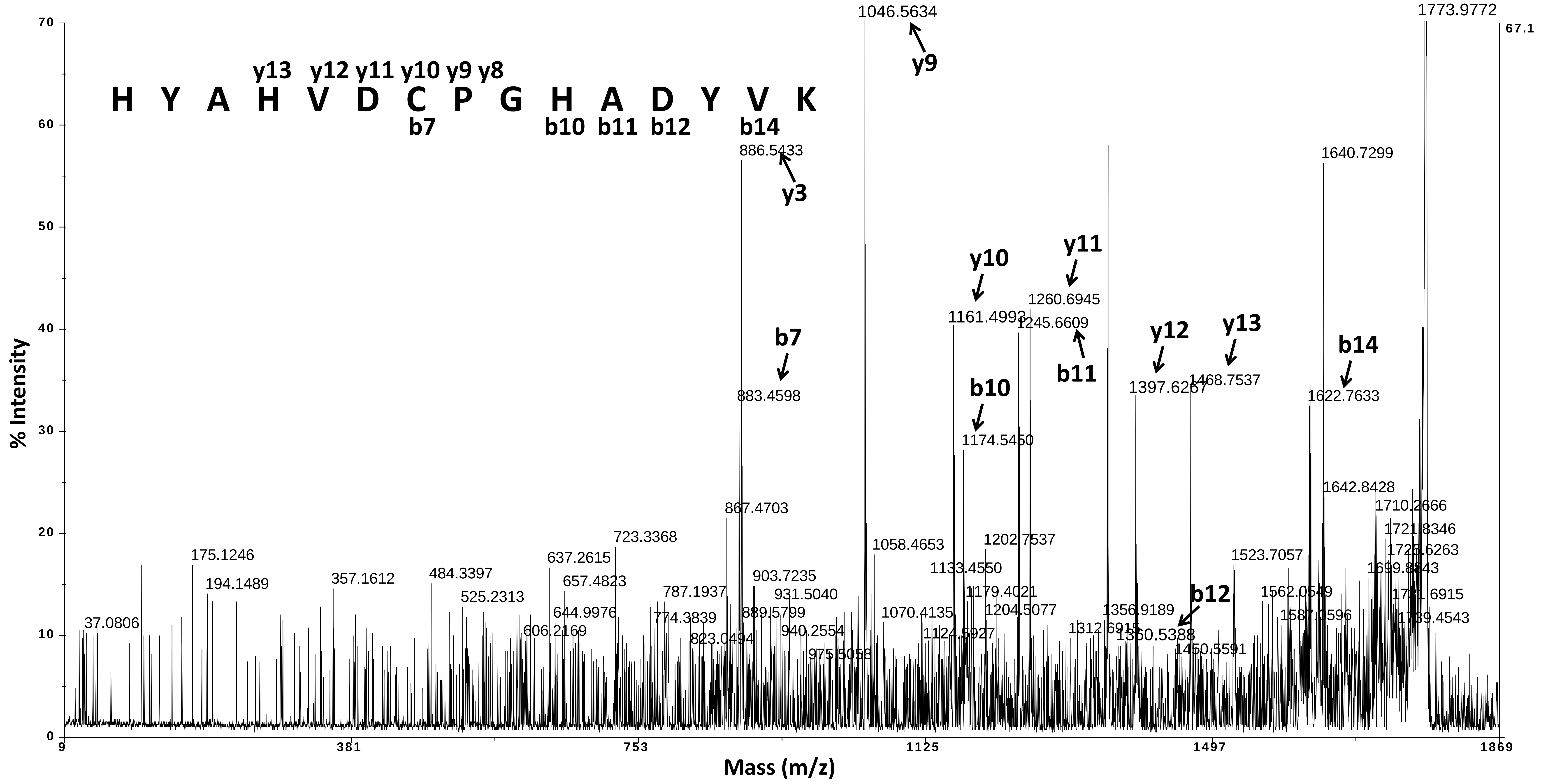

Elongation Factor Tu: MS/MS Precursor – 1363.53

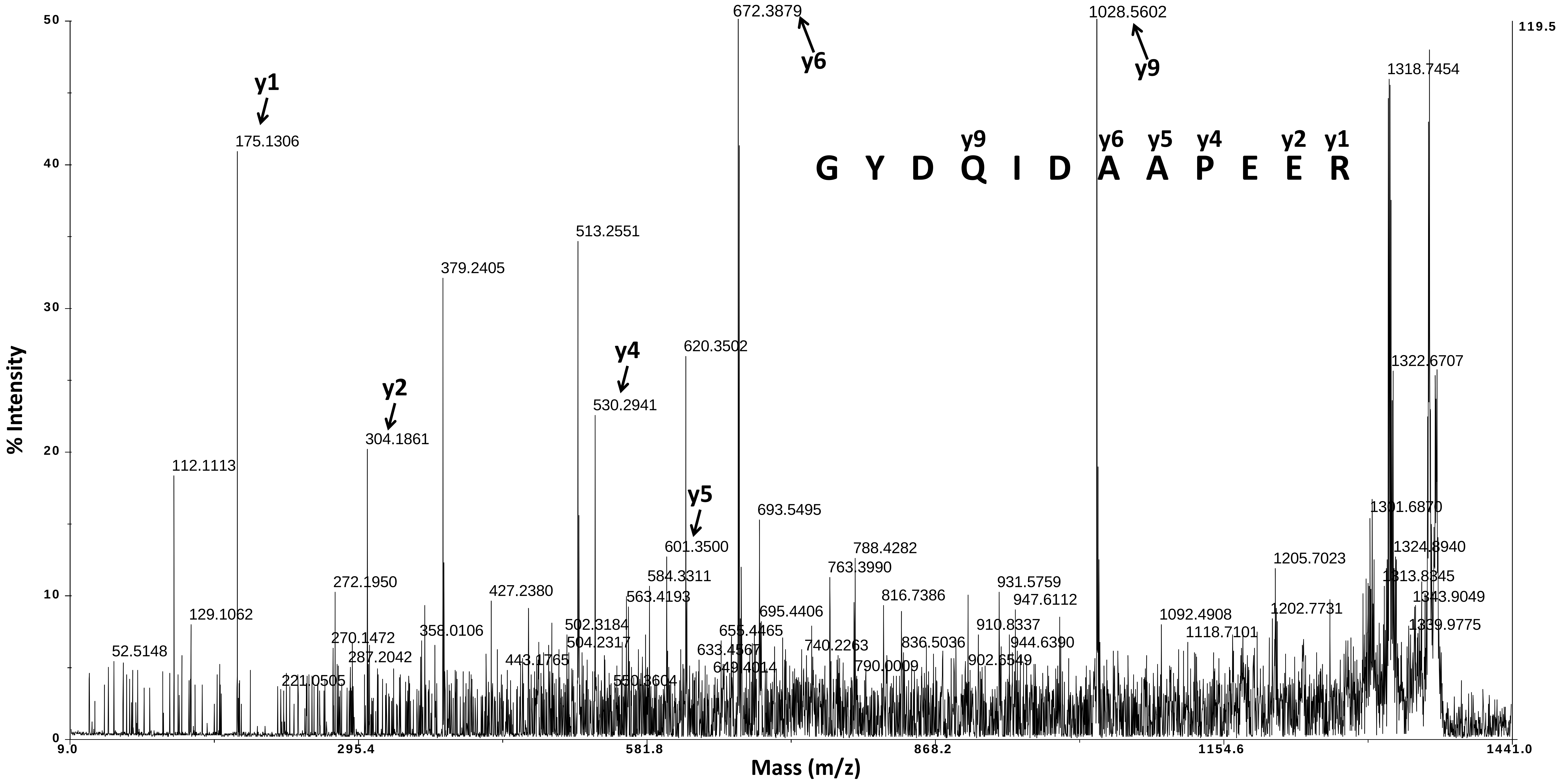

Elongation Factor Tu: MS/MS Precursor – 1959.9

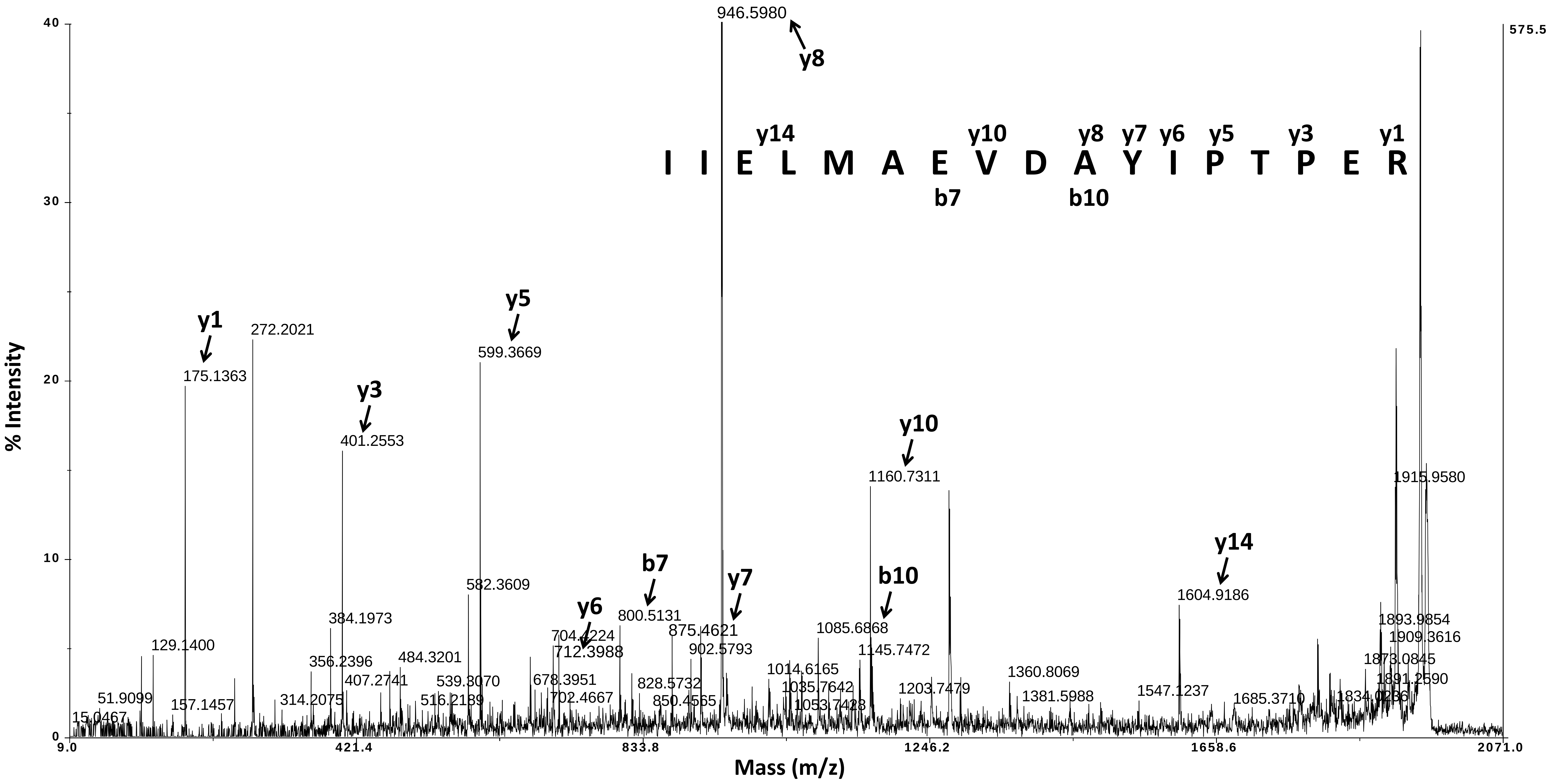

Supplement: Supplementary file 1 — Supplementary Information. [file 41598_2020_59156_MOESM1_ESM.pdf]
